# Decarboxylative sulfoximination of benzoic acids enabled by photoinduced ligand-to-copper charge transfer†

**DOI:** 10.1039/d2sc05442f

**Published:** 2022-11-14

**Authors:** Peng Xu, Wanqi Su, Tobias Ritter

**Affiliations:** Max-Planck-Institut für Kohlenforschung Kaiser-Wilhelm Platz 1 D-45470 Mülheim an der Ruhr Germany ritter@mpi-muelheim.mpg.de; Institute of Organic Chemistry, RWTH Aachen University Landoltweg 1 52074 Aachen Germany

## Abstract

Sulfoximines are synthetically important scaffolds and serve important roles in drug discovery. Currently, there is no solution to decarboxylative sulfoximination of benzoic acids; although thoroughly investigated, limited substrate scope and harsh reaction conditions still hold back traditional thermal aromatic decarboxylative functionalization. Herein, we realize the first decarboxylative sulfoximination of benzoic acids *via* photo-induced ligand to copper charge transfer (copper-LMCT)-enabled decarboxylative carbometalation. The transformation proceeds under mild reaction conditions, has a broad substrate scope, and can be applied to late-stage functionalization of complex small molecules.

## Abstract 2

We report the first decarboxylative sulfoximination of benzoic acids *via* photoinduced ligand-to-copper charge transfer. The transformation proceeds at ambient reaction temperature and demonstrates broad substrate scope.

## Graphical abstract

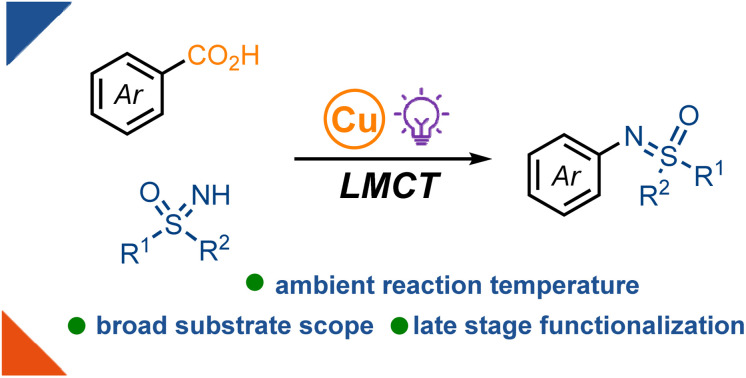

## Introduction

Coordination of substrates including alcohols,^1^ halides,^2^ azides,^3^ and alkyl carboxylates^4^ to abundant 3d metal salts like Fe, Ni, or Cu can form photoactive metal complexes. Promoted to their excited states upon irradiation can result in intramolecular ligand-to-metal charge transfer (LMCT) within the excited state complexes to generate reactive open-shell radical intermediates with reactivity hardly reachable in the ground state.^1–5^ Conventional metal-catalysed or -mediated thermal decarboxylative cross-coupling reactions normally require high reaction temperature and *ortho*-substituents.^6^ Radical aromatic decarboxylation proceeds about three orders of magnitude slower than from aliphatic carboxyl radicals,^7,8^ which generally leads to undesirable side reactions such as hydrogen atom abstraction for benzoyl radicals.^7^ As a consequence, many aromatic decarboxylative bond forming reactions, including sulfoximination, are still out of reach for conventional reaction chemistry. Here we report the first decarboxylative sulfoximination of benzoic acids enabled by photo-induced ligand to copper charge transfer. Photoactive copper(ii) carboxylates undergo a low-barrier radical CO_2_ extrusion upon irradiation, with the putative formed aryl radicals subsequently captured by copper complexes to generate CuAr(iii) species for C–N reductive elimination. The synthetic utility of this method was exemplified by late-stage decarboxylative sulfoximination of several complex small-molecule benzoic acids, which are abundantly available from nature.

**Fig. 1:**
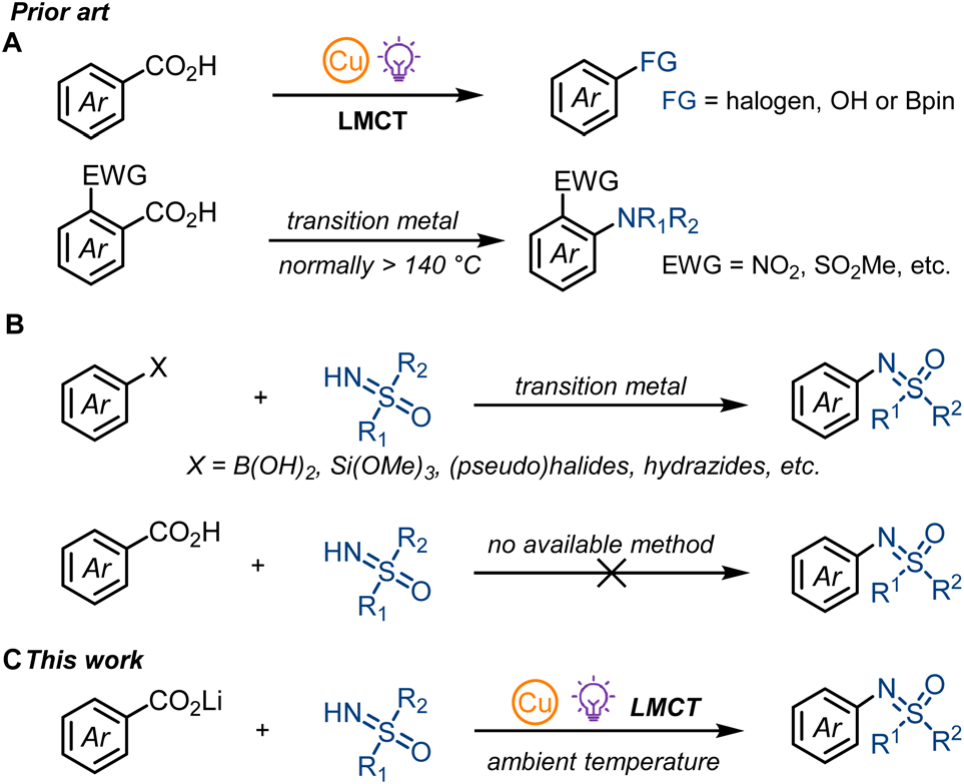
(A) Prior art of copper-LMCT enabled decarboxylation of benzoic acids and thermal aromatic decarboxylative C–N cross couplings. (B) Previous synthesis of *N*-arylated sulfoximines *via N*-arylation of NH-sulfoximines. (C) Aromatic decarboxylative sulfoximination *via* copper-LMCT.

Photonic excitation of copper(ii) complexes have been known as an effective platform for generating reactive radicals for decades. Kochi first studied the addition and C_sp^3^_–H abstraction reactivity of chlorine radical generated by the photo-irradiation of CuCl_2_ in different organic solvents.^2*a*^ Based on this initial finding, Wan and co-workers developed a vicinal dichlorination of alkenes catalyzed by CuCl_2_ under air,^2*b*^ and the Rovis group realized a copper catalyzed olefination of unactivated C_sp^3^_–H bonds.^2*c*^ In addition to chlorine radicals, copper-LMCT is also suitable for N- or C-centered radical generation. In 2018, Rehbein and Reiser found that copper-LMCT was effective for azide radical generation,^3^ and Wang and Xu suggested the formation of N-centered radical cations *via* intramolecular LMCT of quinolinyl-8-glycinate ester coordinated alkyl-Cu(iii) adducts.^9^ For C-centered radicals, Gong and co-workers proposed the generation of alkyl radical intermediates *via* photolysis of Cu(ii)-alkyl complexes.^10^ Although copper-LMCT in copper(ii) carboxylate complexes was first described by DeGraff and co-workers during their research on the photolysis of copper(ii)-malonate,^4*a*,*b*^ it was not until 2021 that our group applied this copper-LMCT reactivity to synthetic applications.^11^ We realized the first aromatic decarboxylative fluorination^11*a*^ and the decarboxylative hydroxylation^11*b*^ of benzoic acids. Concurrently, the MacMillan group explored the copper-LMCT reactivity in aromatic decarboxylative borylation^12*a*^ and halogenation,^12*b*^ and achieved copper catalysis for transformations with single electron oxidants such as 1-fluoro-2,4,6-trimethylpyridinium tetrafluoroborate (NFTPT). Stoichiometric copper is still required for nucleophiles such as fluoride.^11*a*^ Nearly at the same time, the Yoon group developed a copper-mediated oxidative decarboxylative functionalization of aliphatic carboxylic acids.^4*e*^ Most recently, Reiser and co-workers reported a copper-catalysed aliphatic decarboxylative oxygenation methodology, where oxygen was applied as the oxidant.^4*i*^ While we and others have developed the concept of photo-induced copper-LMCT-enabled aromatic radical decarboxylation to achieve previously unknown reactivity, the coupling counterparts are generally limited to halides, carboxylates or boronate esters but strong coordinating NH-nucleophiles have not been shown to react (Fig. 1A).^11,12^ Given that copper-LMCT relies on the coordination of carboxylates to copper(ii) species, strong coordination of NH-nucleophiles such as NH-sulfoximines will compete with carboxylates and form undesired copper species that can diminish the reaction efficiency. Though thermal aromatic decarboxylative C–N cross couplings under high reaction temperatures (normally more than 140 °C) have been explored by Jia, Gooβen, and Xie, electron-deficient *ortho*-substituted benzoic acids are required for efficient CO_2_ extrusion (Fig. 1A).^13^ Thus, a general aromatic decarboxylative C–N cross coupling still remains elusive.

The study of sulfoximines dates back as far as 1949 when methionine sulfoximine was first synthesized by Bentley and Whitehead.^14^ Today, sulfoximines play a remarkable role in synthetic chemistry^15^ and drug discovery;^16^ they have been applied as chiral auxiliaries,^17^ chiral ligands,^18^ asymmetric organocatalysts^19^ and building blocks.^15,20^*N*-arylated sulfoximines are mainly prepared by transition-metal-catalyzed direct *N*-arylation of NH-sulfoximines with aryl (pseudo)halides,^21^ arylboronic acids,^22^ aryl siloxanes,^23^ acyl peroxides,^24^ arylsulfinates,^25^ and aryl hydrazides^26^ but cannot be accessed from benzoic acids (Fig. 1B). Herein, we present the first decarboxylative sulfoximination of benzoic acids by applying a photo-induced carboxylate-to-copper charge transfer strategy (Fig. 1C). We found that lithium carboxylates with 2,6-di-*tert*-butylpyridine (DTBP) and LiOMe as additives was able to overcome the challenging low reaction efficiency associated with copper-LMCT-enabled aromatic decarboxylative sulfoximination.

## Results and discussion

Because of the enhanced N–H acidity, NH-sulfoximines can undergo facile deprotonation and readily coordinate with copper(ii) species.^16*c*^ In the copper-LMCT process, competing coordination of sulfoximines to copper(ii) might hinder the formation of key copper(ii) carboxylate intermediates and, in turn, decrease the reaction efficiency. We hypothesized that initial deprotonation of the benzoic acids to their carboxylate salts might facilitate the generation of photoactive copper(ii) carboxylates, while the careful screening of additives can hinder the formation of undesired sulfoximine-ligated copper(ii) species.

As shown in Table 1, we verified our assumption *via* the decarboxylative sulfoximination of lithium 4-fluorobenzoate (1). A series of reaction condition optimization (see ESI for more details†) reveals that purple light irradiation of a mixture of 1, NH-sulfoximine 2, Cu(OTf)_2_, LiOMe and 2,6-di-*tert*-butylpyridine (DTBP) in MeCN can afford *N*-arylated sulfoximine 3 in 66% yield, together with side product ester 3a in 8% yield (Table 1, entry 1). Compared with 4-fluorobenzoic acid, 4-fluorobenzoate salts underwent more efficient decarboxylation, and the lithium salts gave the best yield (entries 1–3). The result is consistent with our hypothesis that the use of benzoate salts can promote the formation of copper carboxylates and increase the reaction efficiency. Notably, the combination of bulky DTBP and LiOMe is crucial to obtain a high yield. Replacing DTBP/LiOMe with other inorganic or organic bases generally led to lower yields, and a large amount of side oxydecarboxylation product 3a was detected (entries 4–6). Strong coordinating bi- or tridentate pyridine-based ligands or bidentate PyBOX ligand were also tested, yet no better yields were observed (see ESI for more details†). We assume that weak ligation of bulky DTBP to copper might favour C–N reductive elimination over C–O reductive elimination or help to form photoactive copper(ii) carboxylates species. The role of LiOMe is not very clear; we propose that the addition of LiOMe might help decrease the concentration of free sulfoximines by forming poorly soluble sulfoximine lithium salts and in turn, accelerate the formation of copper(ii) carboxylate species. Low conversion of starting substrates 1 and 2 was observed when copper sources including Cu(OAc)_2_ were used instead of Cu(OTf)_2_ (entry 7). Interestingly, only MeCN as solvent was productive, and DCM only afforded 12% protodecarboxylation side-product fluorobenzene (entry 8). Control experiments confirmed the essential use of 390 nm LEDs irradiation for CO_2_ extrusion, and no decarboxylation was observed under thermal reaction conditions.

**Table 1:** Optimization of the reaction conditions^a^

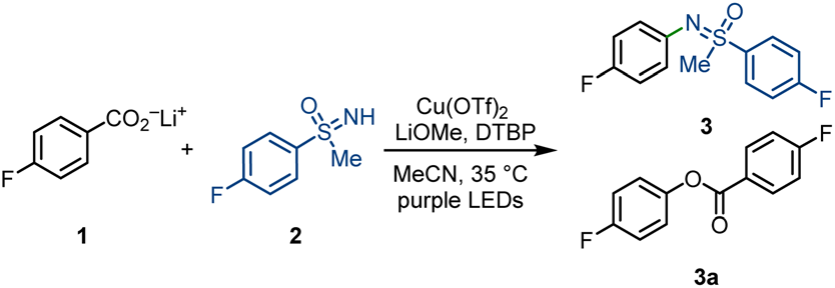		
Entry	Variation	Yield of 3/3a^b^ (%)
1	None	66/8
2	H^+^ instead of Li^+^	44/8
3	Na^+^ instead of Li^+^	60/12
4	Li_2_CO_3_ instead of LiOMe/DTBP	48/20
5	KF instead of LiOMe/DTBP	38/40
6	2,6-Difluoropyridine instead of LiOMe/DTBP	22/36
7	Cu(OAc)_2_ instead of Cu(OTf)_2_	0/0
8	DCM instead of MeCN	0/0^c^
9	No DTBP	0/0
10	No LiOMe	58/8

a Reaction conditions: 1 (0.05 mmol, 1.0 equiv.), 2 (2.5 equiv.), Cu(OTf)_2_ (2.5 equiv.), LiOMe (1.0 equiv.), 2,6-di-*tert*-butylpyridine (DTBP, 2.0 equiv.), MeCN (*c* = 25 mM), 18 h purple LEDs irradiation, 35 °C.

b Yields were determined by ^19^F NMR using 2-fluorotoluene (2.0 equiv.) as an internal standard.

c 12% yield of protodecarboxylation product fluorobenzene was observed.

**Fig. 2:**
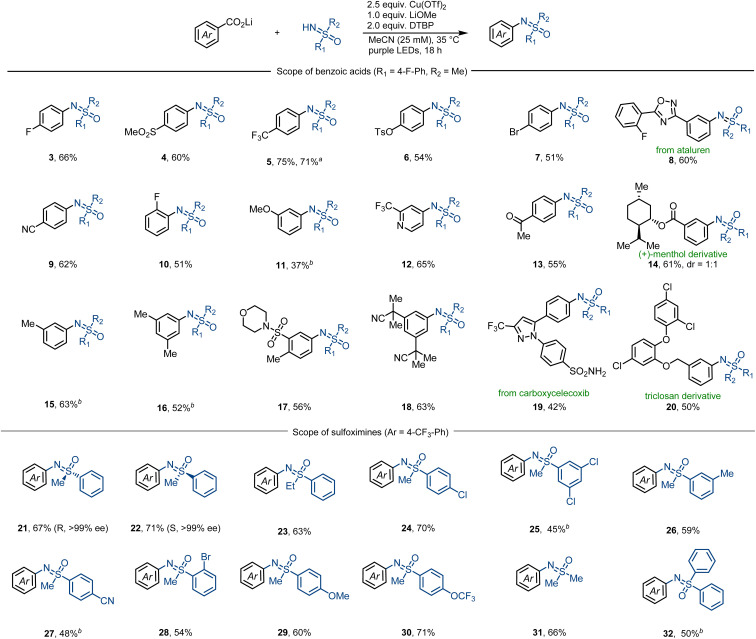
Substrate scope. Standard reaction condition: lithium benzoate (0.20 mmol, 1.0 equiv.), sulfoximine (2.5 equiv.), Cu(OTf)_2_ (2.5 equiv.), LiOMe (1.0 equiv.), DTBP (2.0 equiv.), MeCN (*c* = 25 mM), 18 h purple LEDs irradiation, 35 °C. ^*a*^Reaction was performed at 1.0 mmol scale. ^*b*^Reaction condition: lithium benzoate (0.20 mmol, 1.0 equiv.), sulfoximine (2.5 equiv.), Cu(MeCN)_4_BF_4_ (2.5 equiv.), 1-fluoro-2,4,6-trimethylpyridinium triflate (2.5 equiv.), DTBP (2.0 equiv.), MeCN (*c* = 25 mM), 18 h purple LEDs irradiation, 35 °C. DTBP = 2,6-di-*tert*-butylpyridine.

Subsequently, we next studied the substrate scope of the decarboxylative sulfoximination (Fig. 2). Electron-deficient (4, 5, 9, 17), electron-neutral (3, 7, 10) and electron-rich (6, 11, 16) benzoic acids underwent smooth decarboxylative sulfoximination to afford their corresponding *N*-arylated sulfoximines in moderate to good yields. Owing to the high oxidative potential, radical decarboxylation of electron-deficient benzoic acids is generally problematic,^27^ however, performed well under our present reaction condition. Ortho-fluoro-substituted benzoic acid (10) gave a moderate yield; yet, benzoic acids with large *ortho*-substituents failed to afford productive yields, possibly owing to the insufficient generation of copper(ii) carboxylates. Heteroaromatic carboxylic acids such as CF_3_-substituted isonicotinic acid can also perform efficient decarboxylation to afford the corresponding *N*-arylated sulfoximine 12. Functional groups including aryl halides (7, 20), ketone (13), heterocycles (8, 12, 19), nitriles (9, 18) and sulfonamides (17, 19) were well tolerated. α-O or –N (11, 14, 17, 20), benzylic (15–17, 20), and tertiary (14) C–H bonds that are sensitive to HAT do not prevent efficient decarboxylative sulfoximination. In addition, strong coordinating or oxidizable functional groups like amines inhibit the transformation. The utility of this decarboxylative sulfoximination was further displayed by the late-stage decarboxylative sulfoximination of several complex small molecules (8, 14, 19, 20). NH-Sulfoximines with electron-rich (29, 30) and electron-neutral (21–24) arenes afforded good yields; however, electron-deficient NH-sulfoximines (25, 27) gave lower yields, possibly due to their weaker N-nucleophilicity. Dialkyl (31) and diaryl (32) NH-sulfoximines furnished their corresponding *N*-arylated sulfoximines in moderate yields. Benefiting from the mild reaction conditions, *N*-arylation of enantiopure NH-sulfoximines (21, 22) proceeded in good yields, and no racemization was observed. In most cases, low conversion of the starting benzoate salts accounts for the observed low yield.

**Fig. 3:**
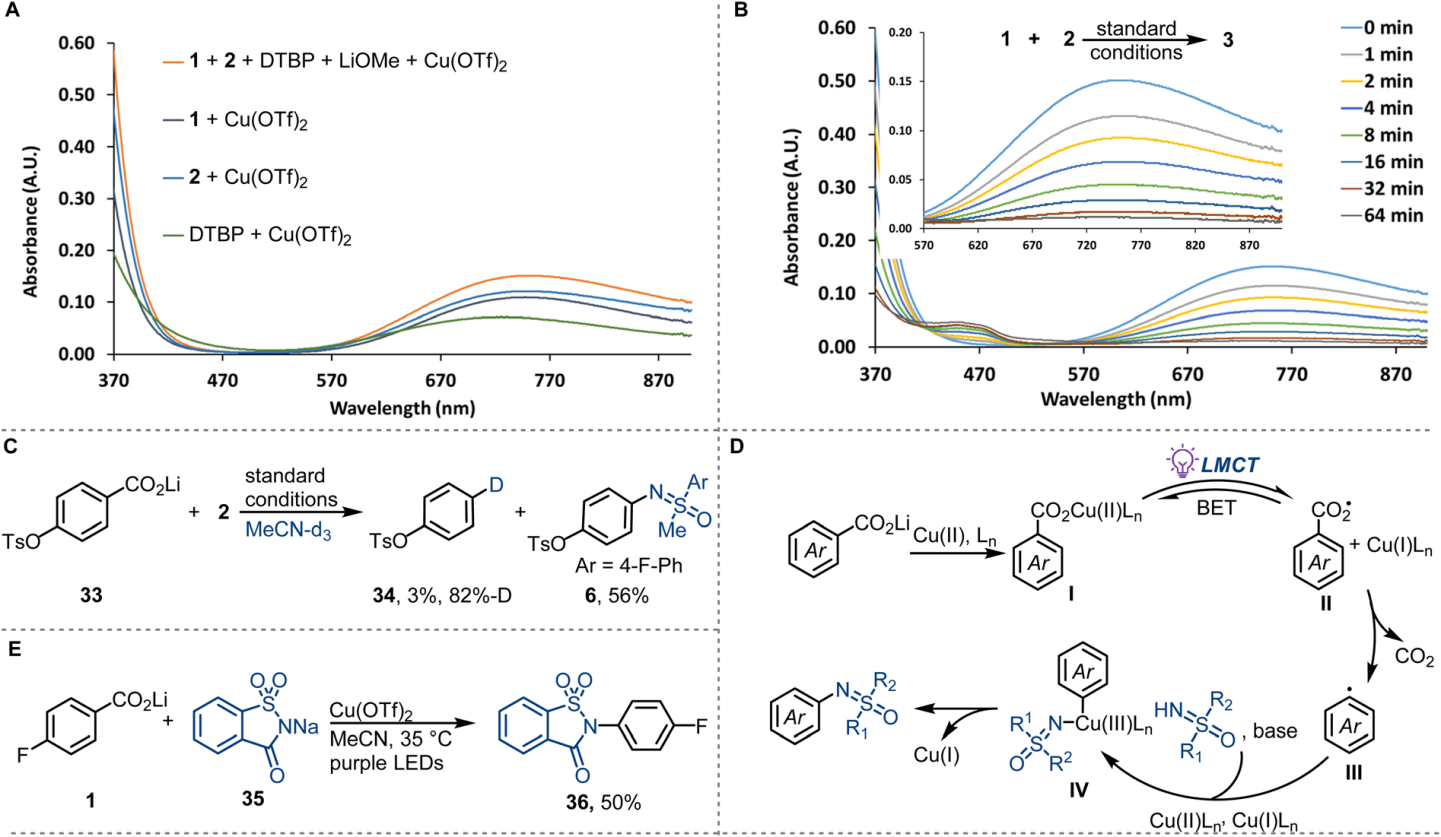
Mechanistic investigations and synthetic application. DTBP = 2,6-di-*tert*-butylpyridine. (A) UV-vis absorption spectra of reaction components. (B) UV-vis spectral changes observed upon photolysis of a mixture of 1 (1.0 mM), 2 (2.5 mM), Cu(OTf)_2_ (2.5 mM), LiOMe (1.0 mM), and DTBP (2.0 mM) in MeCN under purple LEDs irradiation (0–64 min). (C) Deuterodecarboxylation. (D) Proposed reaction mechanism. (E) Decarboxylative C–N cross-coupling with sodium saccharin as the N-nucleophile.

Preliminary investigations to study the reaction mechanism are consistent with the mechanism shown in Fig. 3. In the UV-vis absorption spectrum of the mixture of lithium 4-fluorobenzoate (1) and Cu(OTf)_2_, a strong absorbance (370–470 nm) attributed to the LMCT band of copper(ii) carboxylates was detected (Fig. 3A).^28^ The LMCT band overlaps with the purple LED emission spectrum, consistent with the excitation of copper(ii) carboxylates under the reaction conditions. The coordination of sulfoximines to copper(ii) and the coordination of 2,6-di-*tert*-butylpyridine (DTBP) to copper(ii) are in agreement with the observation of an absorbance (370–470 nm) of a mixture of sulfoximine 2 and Cu(OTf)_2_, and a mixture of DTBP and Cu(OTf)_2_ (Fig. 3A). All copper(ii)-containing mixtures display a broad d–d transitions absorbance at 550−900 nm, which decreased monotonously upon purple LED irradiation, consistent with the reduction of Cu(ii) to Cu(i) (Fig. 3B). The formation of Cu(i) was confirmed by the observation of a characteristic purple [Cu^I^(biq)_2_]^+^ complex when 2,2′-biquinoline (biq) was added to the irradiated reaction mixture (see ESI for more detail†).^29^ Additional experiments were then performed to explore the transformation of carboxylate ions upon irradiation. In radical trapping experiments, aryl carboxyl radical adduct phenyl 3-methoxybenzoate and aryl radical adduct 3-methoxy-1,1′-biphenyl were separated, which indicated the formation of aryl carboxyl and aryl radicals (see ESI for more details†). The presence of aryl carboxyl radical and aryl radical was further confirmed by 6-*endo-trig* intramolecular radical cyclisation (see ESI for more details†) and radical deuterodecarboxylation (Fig. 3C), respectively. *N*-Phenyl-sulfoximine, possibly formed by radical addition of sulfoximinyl radical to benzene, was also identified in the radical trapping experiments. We hypothesize that the sulfoximinyl radical is formed *via* a nitrogen to copper charge transfer in the copper(ii) sulfoximine complex, with consumption of the copper(ii) species. This result is consistent with competing coordination of sulfoximines to copper(ii) and may also explain the low reaction reactivity caused by the competing coordination. Based on the above mechanistic investigation, we propose a mechanism as depicted in Fig. 3D. Photo-induced carboxylate to copper(ii) charge transfer in copper(ii) carboxylates I affords aryl carboxyl radical intermediates II, which then undergo low-barrier radical decarboxylation to afford aryl radicals III. Subsequent copper-assisted aryl radical capture generates arylcopper(iii) intermediates IV that finally undergo C–N reductive elimination to afford *N*-arylated sulfoximines (Fig. 3D). Additionally, we found that other N-nucleophiles, such as *ortho*-sulphobenzamide, could also be coupled by the copper-LMCT approach (Fig. 3E).

## Conclusions

Copper-LMCT based radical aromatic decarboxylative carbometalation enabled the first decarboxylative sulfoximination of benzoic acids. The broad substrate scope and good functional group tolerance demonstrate the generality of the copper-LMCT concept in aromatic decarboxylative sulfoximination. Conceptually, the success of this transformation demonstrates the expansion of the copper-LMCT concept for aromatic decarboxylative cross-couplings to reactions with strongly coordinating nucleophiles.

## Data availability

Procedures and compound characterization are provided in the ESI.†

## Author contributions

P. X. initiated the project and performed experiments and analyzed the data. W. S. performed and analyzed experiments regarding the mechanism. T. R. directed the project.

## Conflicts of interest

The authors declare no competing interests.

## Supplementary Material

SC-013-D2SC05442F-s001
