# Supplementary material for: Decarboxylative sulfoximination of benzoic acids enabled by photoinduced ligand-to-copper charge transfer
Source: Chem Sci. 2022 Nov 14;13(45):13611–6. doi: 10.1039/d2sc05442f (PMC9682917; doi:10.1039/d2sc05442f)

Electronic Supplementary Material (ESI) for Chemical Science.
This journal is © The Royal Society of Chemistry 2022

SUPPORTING INFORMATION

Decarboxylative Sulfoximination of Benzoic Acids Enabled by Photoinduced Ligand-to-Copper Charge Transfer

Peng Xu[1], Wanqi Su[1,2], Tobias Ritter[1]*

[1]Max-Planck-Institut für Kohlenforschung, Kaiser-Wilhelm Platz 1, D-45470 Mülheim an der Ruhr, Germany

[2]Institute of Organic Chemistry, RWTH Aachen University, Landoltweg 1, 52074 Aachen, Germany

*E-mail: ritter@kofo.mpg.de

TABLE OF CONTENTS

TABLE OF CONTENTS 1

MATERIALS AND METHODS 7

EXPERIMENTAL DATA 8

General procedure of preparing lithium aryl carboxylate 8

General procedure for aromatic decarboxylative sulfoximination 8

Reaction condition optimization 9

Table S1 Counterion 9

Table S2 Additive optimization 9

Table S3 Cu(II) salt optimization 10

Table S4 Solvent optimization 10

Table S5 Control experiments 11

Photo-induced LMCT-enabled aromatic decarboxylative sulfoximination 12

(4-Fluorophenyl)((4-fluorophenyl)imino)(methyl)-λ^6-sulfanone (**3**) 12

(4-Fluorophenyl)(methyl)((4-(methylsulfonyl)phenyl)imino)-λ^6-sulfanone (**4**) 13

(4-Fluorophenyl)(methyl)((4-(trifluoromethyl)phenyl)imino)-λ^6-sulfanone (5) 14

4-(((4-Fluorophenyl)(methyl)(oxo)-λ^6-sulfanylidene)amino)phenyl 4-methylbenzenesulfonate (**6**) 15

((4-Bromophenyl)imino)(4-fluorophenyl)(methyl)-λ^6-sulfanone (**7**) 15

(4-Fluorophenyl)((3-(5-(2-fluorophenyl)-1,2,4-oxadiazol-3-yl)phenyl)imino)(methyl)-λ^6-sulfanone (**8**) 16

((4-Cyanophenyl)imino)(4-fluorophenyl)(methyl)-λ^6-sulfanone (**9**) 17

((2-Fluorophenyl)imino)(4-fluorophenyl)(methyl)-λ^6-sulfanone (**10**) 18

((3-Methoxyphenyl)imino)(4-fluorophenyl)(methyl)-λ^6-sulfanone (**11**) 18

(4-Fluorophenyl)(methyl)((2-(trifluoromethyl)pyridin-4-yl)imino)-λ^6-sulfanone (**12**) 19

((4-Cyanophenyl)imino)(4-fluorophenyl)(methyl)-λ^6-sulfanone (**13**) 20

(+)-Menthol derivative **14** 20

((3-Methylphenyl)imino)(4-fluorophenyl)(methyl)-λ^6-sulfanone (**15**) 21

((3,5-Dimethylphenyl)imino)(4-fluorophenyl)(methyl)-λ^6-sulfanone (**16**) 22

(4-Fluorophenyl)(methyl)((4-methyl-3-(morpholinosulfonyl)phenyl)imino)-λ^6-sulfanone (**17**) 23

2,2'-(5-(((4-Fluorophenyl)(methyl)(oxo)-λ^6-sulfanylidene)amino)-1,3-phenylene)bis(2-methylpropanenitrile) (**18**) 23

Celecoxib analogue **19** 24

Triclosan derivative **20** 25

(*R*)-Methyl(phenyl)((4-(trifluoromethyl)phenyl)imino)-λ^6-sulfanone (**21**) 26

(*S*)-Methyl(phenyl)((4-(trifluoromethyl)phenyl)imino)-λ^6-sulfanone (**22**) 28

Ethyl(phenyl)((4-(trifluoromethyl)phenyl)imino)-λ^6-sulfanone (**23**) 30
(4-Chlorophenyl)(methyl)((4-(trifluoromethyl)phenyl)imino)-λ^6-sulfanone (**24**) 31
(3,5-Dichlorophenyl)(methyl)((4-(trifluoromethyl)phenyl)imino)-λ^6-sulfanone (**25**) 32
(3-Methylphenyl)(methyl)((4-(trifluoromethyl)phenyl)imino)-λ^6-sulfanone (**26**) 33
(4-Cyanophenyl)(methyl)((4-(trifluoromethyl)phenyl)imino)-λ^6-sulfanone (**27**) 33
(2-Bromophenyl)(methyl)((4-(trifluoromethyl)phenyl)imino)-λ^6-sulfanone (**28**) 34
(4-Methoxyphenyl)(methyl)((4-(trifluoromethyl)phenyl)imino)-λ^6-sulfanone (**29**) 35
(4-Trifluoromethoxyphenyl)(methyl)((4-(trifluoromethyl)phenyl)imino)-λ^6-sulfanone (**30**) 36
Dimethyl((4-(trifluoromethyl)phenyl)imino)-λ^6-sulfanone (**31**) 36
Diphenyl((4-(trifluoromethyl)phenyl)imino)-λ^6-sulfanone (**32**) 37
1 mmol scale decarboxylative sulfoximination of lithium 4-(trifluoromethyl)benzoate 38
Tested *ortho*-substituted substrates 38
Reactions with catalytic amounts of $Cu(OTf)_2$ and oxidants 39

Mechanistic Studies 39

UV-vis Absorption Spectrum 39
Photolysis of the mixture of 1, 2, DTBP, LiOMe and $Cu(OTf)_2$ 40
Radical trapping experiments 42
Deuterodecarboxylation 44
Radical cyclisation experiment 45
Decarboxylative sulfoximination of 4-fluorobenzoic acid derived oxime ester 46
Example of decarboxylative amination 46

REFERENCES 47

SPECTROSCOPIC DATA 48

^{1}H NMR of (4-fluorophenyl)((4-fluorophenyl)imino)(methyl)-λ^6-sulfanone (**3**) 48

^{13}C NMR of (4-fluorophenyl)((4-fluorophenyl)imino)(methyl)-λ^6-sulfanone (**3**) 49

^{19}F NMR of (4-fluorophenyl)((4-fluorophenyl)imino)(methyl)-λ^6-sulfanone (**3**) 50

^{1}H NMR of (4-fluorophenyl)(methyl)((4-(methylsulfonyl)phenyl)imino)-λ^6-sulfanone (**4**) 51

^{13}C NMR of (4-fluorophenyl)(methyl)((4-(methylsulfonyl)phenyl)imino)-λ^6-sulfanone (**4**) 52

^{19}F NMR of (4-fluorophenyl)(methyl)((4-(methylsulfonyl)phenyl)imino)-λ^6-sulfanone (**4**) 53

^{1}H NMR of (4-fluorophenyl)(methyl)((4-(trifluoromethyl)phenyl)imino)-λ^6-sulfanone (**5**) 54

^{13}C NMR of (4-fluorophenyl)(methyl)((4-(trifluoromethyl)phenyl)imino)-λ^6-sulfanone (**5**) 55

^{19}F NMR of (4-fluorophenyl)(methyl)((4-(trifluoromethyl)phenyl)imino)-λ^6-sulfanone (**5**) 56

^{1}H NMR of 4-(((4-fluorophenyl)(methyl)(oxo)-λ^6-sulfanylidene)amino)phenyl 4-methylbenzenesulfonate (**6**)

.... 57

^{13}C NMR of 4-(((4-fluorophenyl)(methyl)(oxo)-λ^6-sulfanylidene)amino)phenyl 4-methylbenzenesulfonate (**6**) 58

^{19}F NMR of 4-(((4-fluorophenyl)(methyl)(oxo)-λ^6-sulfanylidene)amino)phenyl 4-methylbenzenesulfonate (**6**) 59

^1H NMR of ((4-bromophenyl)imino)(4-fluorophenyl)(methyl)-λ^6-sulfanone (**7**) 60

^{13}C NMR of ((4-bromophenyl)imino)(4-fluorophenyl)(methyl)-λ^6-sulfanone (**7**) 61

^{19}F NMR of ((4-bromophenyl)imino)(4-fluorophenyl)(methyl)-λ^6-sulfanone (**7**) 62

^1H NMR of (4-fluorophenyl)((3-(5-(2-fluorophenyl)-1,2,4-oxadiazol-3-yl)phenyl)imino)(methyl)-λ^6-sulfanone (**8**) 63

^{13}C NMR of (4-fluorophenyl)((3-(5-(2-fluorophenyl)-1,2,4-oxadiazol-3-yl)phenyl)imino)(methyl)-λ^6-sulfanone (**8**) 64

^{19}F NMR of (4-fluorophenyl)((3-(5-(2-fluorophenyl)-1,2,4-oxadiazol-3-yl)phenyl)imino)(methyl)-λ^6-sulfanone (**8**) 65

^1H NMR of ((4-cyanophenyl)imino)(4-fluorophenyl)(methyl)-λ^6-sulfanone (**9**) 66

^{13}C NMR of ((4-cyanophenyl)imino)(4-fluorophenyl)(methyl)-λ^6-sulfanone (**9**) 67

^{19}F NMR of ((4-cyanophenyl)imino)(4-fluorophenyl)(methyl)-λ^6-sulfanone (**9**) 68

^1H NMR of ((2-fluorophenyl)imino)(4-fluorophenyl)(methyl)-λ^6-sulfanone (**10**) 69

^{13}C NMR of ((2-fluorophenyl)imino)(4-fluorophenyl)(methyl)-λ^6-sulfanone (**10**) 70

^{19}F NMR of ((2-fluorophenyl)imino)(4-fluorophenyl)(methyl)-λ^6-sulfanone (**10**) 71

^1H NMR of ((3-Methoxyphenyl)imino)(4-fluorophenyl)(methyl)-λ^6-sulfanone (**11**) 72

^{13}C NMR of ((3-Methoxyphenyl)imino)(4-fluorophenyl)(methyl)-λ^6-sulfanone (**11**) 73

^{19}F NMR of ((3-Methoxyphenyl)imino)(4-fluorophenyl)(methyl)-λ^6-sulfanone (**11**) 74

^1H NMR of (4-fluorophenyl)(methyl)((2-(trifluoromethyl)pyridin-4-yl)imino)-λ^6-sulfanone (**12**) 75

^{13}C NMR of (4-fluorophenyl)(methyl)((2-(trifluoromethyl)pyridin-4-yl)imino)-λ^6-sulfanone (**12**) 76

^{19}F NMR of (4-fluorophenyl)(methyl)((2-(trifluoromethyl)pyridin-4-yl)imino)-λ^6-sulfanone (**12**) 77

^1H NMR of ((4-acetylphenyl)imino)(4-fluorophenyl)(methyl)-λ^6-sulfanone (**13**) 78

^{13}C NMR of ((4-acetylphenyl)imino)(4-fluorophenyl)(methyl)-λ^6-sulfanone (**13**) 79

^{19}F NMR of ((4-acetylphenyl)imino)(4-fluorophenyl)(methyl)-λ^6-sulfanone (**13**) 80

^1H NMR of (+)-menthol derivative **14** 81

^{13}C NMR of (+)-menthol derivative **14** ... 82

^{19}F NMR of (+)-menthol derivative **14** ... 83

^{1}H NMR of ((3-methylphenyl)imino)(4-fluorophenyl)(methyl)-λ^{6}-sulfanone (**15**) ... 84

^{13}C NMR of ((3-methylphenyl)imino)(4-fluorophenyl)(methyl)-λ^{6}-sulfanone (**15**) ... 85

^{19}F NMR of ((3-methylphenyl)imino)(4-fluorophenyl)(methyl)-λ^{6}-sulfanone (**15**) ... 86

^{1}H NMR of ((3,5-Dimethylphenyl)imino)(4-fluorophenyl)(methyl)-λ^{6}-sulfanone (**16**) ... 87

^{13}C NMR of ((3,5-Dimethylphenyl)imino)(4-fluorophenyl)(methyl)-λ^{6}-sulfanone (**16**) ... 88

^{19}F NMR of ((3,5-Dimethylphenyl)imino)(4-fluorophenyl)(methyl)-λ^{6}-sulfanone (**16**) ... 89

^{1}H NMR of (4-fluorophenyl)(methyl)((4-methyl-3-(morpholinosulfonyl)phenyl)imino)-λ^{6}-sulfanone (**17**) ... 90

^{13}C NMR of (4-fluorophenyl)(methyl)((4-methyl-3-(morpholinosulfonyl)phenyl)imino)-λ^{6}-sulfanone (**17**) ... 91

^{19}F NMR of (4-fluorophenyl)(methyl)((4-methyl-3-(morpholinosulfonyl)phenyl)imino)-λ^{6}-sulfanone (**17**) ... 92

^{1}H NMR of 2,2'-(5-(((4-fluorophenyl)(methyl)(oxo)-λ^{6}-sulfanylidene)amino)-1,3-phenylene)bis(2-methylpropanenitrile) (**18**) ... 93

^{13}C NMR of 2,2'-(5-(((4-fluorophenyl)(methyl)(oxo)-λ^{6}-sulfanylidene)amino)-1,3-phenylene)bis(2-methylpropanenitrile) (**18**) ... 94

^{19}F NMR of 2,2'-(5-(((4-fluorophenyl)(methyl)(oxo)-λ^{6}-sulfanylidene)amino)-1,3-phenylene)bis(2-methylpropanenitrile) (**18**) ... 95

^{1}H NMR of celecoxib analogue **19** ... 96

^{13}C NMR of celecoxib analogue **19** ... 97

^{19}F NMR of celecoxib analogue **19** ... 98

^{1}H NMR of triclosan derivative **20** ... 99

^{13}C NMR of triclosan derivative **20** ... 100

^{19}F NMR of triclosan derivative **20** ... 102

^{1}H NMR of (*R*)-methyl(phenyl)((4-(trifluoromethyl)phenyl)imino)-λ^{6}-sulfanone (**21**) ... 103

^{13}C NMR of (*R*)-methyl(phenyl)((4-(trifluoromethyl)phenyl)imino)-λ^{6}-sulfanone (**21**) ... 104

^{19}F NMR of (*R*)-methyl(phenyl)((4-(trifluoromethyl)phenyl)imino)-λ^{6}-sulfanone (**21**) ... 105

^{1}H NMR of (*S*)-methyl(phenyl)((4-(trifluoromethyl)phenyl)imino)-λ^{6}-sulfanone (**22**) ... 106

^{13}C NMR of (*S*)-methyl(phenyl)((4-(trifluoromethyl)phenyl)imino)-λ^{6}-sulfanone (**22**) ... 107

^{19}F NMR of (*S*)-methyl(phenyl)((4-(trifluoromethyl)phenyl)imino)-λ^{6}-sulfanone (**22**) ... 108

^{1}H NMR of ethyl(phenyl)((4-(trifluoromethyl)phenyl)imino)-λ^{6}-sulfanone (**23**) 109

^{13}C NMR of ethyl(phenyl)((4-(trifluoromethyl)phenyl)imino)-λ^{6}-sulfanone (**23**) 110

^{19}F NMR of ethyl(phenyl)((4-(trifluoromethyl)phenyl)imino)-λ^{6}-sulfanone (**23**)..111

^{1}H NMR of (4-chlorophenyl)(methyl)((4-(trifluoromethyl)phenyl)imino)-λ^{6}-sulfanone (**24**) 112

^{13}C NMR of (4-chlorophenyl)(methyl)((4-(trifluoromethyl)phenyl)imino)-λ^{6}-sulfanone (**24**).......................113

^{19}F NMR of (4-chlorophenyl)(methyl)((4-(trifluoromethyl)phenyl)imino)-λ^{6}-sulfanone (**24**) 114

^{1}H NMR of (3,5-dichlorophenyl)(methyl)((4-(trifluoromethyl)phenyl)imino)-λ^{6}-sulfanone (**25**) 115

^{13}C NMR of (3,5-dichlorophenyl)(methyl)((4-(trifluoromethyl)phenyl)imino)-λ^{6}-sulfanone (**25**)................116

^{19}F NMR of (3,5-dichlorophenyl)(methyl)((4-(trifluoromethyl)phenyl)imino)-λ^{6}-sulfanone (**25**) 117

^{1}H NMR of (3-methylphenyl)(methyl)((4-(trifluoromethyl)phenyl)imino)-λ^{6}-sulfanone (**26**) 118

^{13}C NMR of (3-methylphenyl)(methyl)((4-(trifluoromethyl)phenyl)imino)-λ^{6}-sulfanone (**26**)......................119

^{19}F NMR of (3-methylphenyl)(methyl)((4-(trifluoromethyl)phenyl)imino)-λ^{6}-sulfanone (**26**) 120

^{1}H NMR of (4-cyanophenyl)(methyl)((4-(trifluoromethyl)phenyl)imino)-λ^{6}-sulfanone (**27**) 121

^{13}C NMR of (4-cyanophenyl)(methyl)((4-(trifluoromethyl)phenyl)imino)-λ^{6}-sulfanone (**27**).......................122

^{19}F NMR of (4-cyanophenyl)(methyl)((4-(trifluoromethyl)phenyl)imino)-λ^{6}-sulfanone (**27**) 123

^{1}H NMR of (2-bromophenyl)(methyl)((4-(trifluoromethyl)phenyl)imino)-λ^{6}-sulfanone (**28**)........................124

^{13}C NMR of (2-bromophenyl)(methyl)((4-(trifluoromethyl)phenyl)imino)-λ^{6}-sulfanone (**28**) 125

^{19}F NMR of (2-bromophenyl)(methyl)((4-(trifluoromethyl)phenyl)imino)-λ^{6}-sulfanone (**28**).......................126

^{1}H NMR of (4-methoxyphenyl)(methyl)((4-(trifluoromethyl)phenyl)imino)-λ^{6}-sulfanone (**29**) 127

^{13}C NMR of (4-methoxyphenyl)(methyl)((4-(trifluoromethyl)phenyl)imino)-λ^{6}-sulfanone (**29**)...................128

^{19}F NMR of (4-methoxyphenyl)(methyl)((4-(trifluoromethyl)phenyl)imino)-λ^{6}-sulfanone (**29**) 129

^{1}H NMR of (4-trifluoromethoxyphenyl)(methyl)((4-(trifluoromethyl)phenyl)imino)-λ^{6}-sulfanone (**30**) 130

^{13}C NMR of (4-trifluoromethoxyphenyl)(methyl)((4-(trifluoromethyl)phenyl)imino)-λ^{6}-sulfanone (**30**).......131

^{19}F NMR of (4-trifluoromethoxyphenyl)(methyl)((4-(trifluoromethyl)phenyl)imino)-λ^{6}-sulfanone (**30**) 132

^{1}H NMR of dimethyl((4-(trifluoromethyl)phenyl)imino)-λ^{6}-sulfanone (**31**)..133

^{13}C NMR of dimethyl((4-(trifluoromethyl)phenyl)imino)-λ^{6}-sulfanone (**31**) 134

^{19}F NMR of dimethyl((4-(trifluoromethyl)phenyl)imino)-λ^{6}-sulfanone (**31**)..135

^{1}H NMR of diphenyl((4-(trifluoromethyl)phenyl)imino)-λ^{6}-sulfanone (**32**)...136

^{13}C NMR of diphenyl((4-(trifluoromethyl)phenyl)imino)-λ^{6}-sulfanone (**32**) 137

^{19}F NMR of diphenyl((4-(trifluoromethyl)phenyl)imino)-λ^6-sulfanone (**32**) 138

^{1}H NMR of phenyl-4-*d* 4-methylbenzenesulfonate (**34**) 139

D NMR of phenyl-4-*d* 4-methylbenzenesulfonate (**34**) 140

^{13}C NMR of phenyl-4-*d* 4-methylbenzenesulfonate (**34**) 141

^{1}H NMR of 2-(4-fluorophenyl)benzo[*d*]isothiazol-3(2*H*)-one 1,1-dioxide (**36**) 142

^{13}C NMR of 2-(4-fluorophenyl)benzo[*d*]isothiazol-3(2*H*)-one 1,1-dioxide (**36**) 143

^{19}F NMR of 2-(4-fluorophenyl)benzo[*d*]isothiazol-3(2*H*)-one 1,1-dioxide (**36**) 144

^{1}H NMR of phenyl 3-methoxybenzoate (**S1**) 145

^{13}C NMR of phenyl 3-methoxybenzoate (**S1**) 146

^{1}H NMR of 3-methoxy-1,1'-biphenyl (**S2**) 147

^{13}C NMR of 3-methoxy-1,1'-biphenyl (**S2**) 148

^{1}H NMR of (4-fluorophenyl)(methyl)(phenylimino)-λ^6-sulfanone (**S3**) 149

^{13}C NMR of (4-fluorophenyl)(methyl)(phenylimino)-λ^6-sulfanone (**S3**) 150

^{19}F NMR of (4-fluorophenyl)(methyl)(phenylimino)-λ^6-sulfanone (**S3**) 151

^{1}H NMR of 6*H*-benzo[c]chromen-6-one (**S4**) 152

^{13}C NMR of 6*H*-benzo[c]chromen-6-one (**S4**) 153

MATERIALS AND METHODS

All air- and moisture-insensitive reactions were carried out under an ambient atmosphere and monitored by thin-layer chromatography (TLC). All air- and moisture-sensitive manipulations were performed using standard *Schlenk* and glove-box techniques under an atmosphere of nitrogen. Concentration under reduced pressure was performed by rotary evaporation at 25–40 °C at an appropriate pressure, unless otherwise stated. Purified compounds were further dried under high vacuum (0.008–0.5 Torr). Yields refer to purified and spectroscopically pure compounds, unless otherwise stated.

Solvents

Anhydrous Acetonitrile, DCM, THF, and toluene were obtained from Phoenix Solvent Drying Systems. All deuterated solvents were purchased from Euriso-Top.

Chromatography

Thin layer chromatography (TLC) was performed using EMD TLC silica gel 60 F254 plates pre-coated with 250 μm thickness silica gel and visualized by fluorescence quenching under UV light or phosphomolybdic acid stain. Preparative TLC was performed using pre-coated TLC plates SIL G-100 UV_{254} (Layer: 1.00 mm silica gel 60 with fluorescent indicator UV_{254}).

Spectroscopy and Instruments

NMR spectra were recorded on a Bruker Ascend^{TM} 500 spectrometer operating at 500 MHz, 471 MHz, and 126 MHz, for ^{1}H, ^{19}F, and ^{13}C acquisitions, respectively; or a Bruker AV600 spectrometer operating at 600 MHz, 565 MHz, 92 MHz, and 151 MHz, for ^{1}H, ^{19}F, D and ^{13}C acquisitions, respectively. Chemical shifts are reported in ppm with the solvent residual peak as the internal standard. For ^{1}H NMR: $CDCl_3$, δ 7.26; CD_2Cl_2, δ 5.32. For ^{13}C NMR: $CDCl_3$, δ 77.16; CD_2Cl_2, δ 53.84.[1] Data is reported as follows: s = singlet, d = doublet, t = triplet, q = quartet, m = multiplet; coupling constants in Hz; integration.

Starting materials

All substrates and materials were used as received from commercial suppliers, unless otherwise stated. $Cu(OTf)_2$ purchased from TCI was dried in a 150 °C oven for 2 hours and stored in a glovebox. $Cu(MeCN)_4BF_4$ and 1-fluoro-2,4,6-trimethylpyridinium triflate were purchased from TCI and stored in a glovebox. *NH*-sulfoximines are purchased from the commercial suppliers or prepared according to the literature[2]. (+)-Menthol-derived benzoic acid[3], carboxycelecoxib[4] and triclosan-derived benzoic acid[5] were synthesized according to the literatures.

EXPERIMENTAL DATA

General procedure of preparing lithium aryl carboxylate

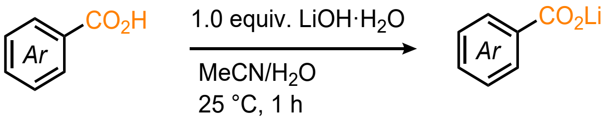

Under an ambient atmosphere, a 20 mL borosilicate vial equipped with a magnetic stir bar was charged with aryl carboxylic acid (1.00 mmol, 1.0 equiv.), lithium hydroxide monohydrate (42.0 mg, 1.00 mmol, 1.0 equiv), and MeCN/H_2O (10.0 mL, v/v = 1:1, *c* = 0.10 M). The reaction mixture was stirred at 25 °C for 1 h. The solvent was evaporated under reduced pressure to afford the lithium aryl carboxylate as a solid. The solid was further dried under high vacuum for 2 hours.

Note: For some substrates, the v/v ratio and the final volume of MeCN/H_2O were adjusted by adding additional MeCN or H_2O according to the solubility of the aryl carboxylic acid and the resulting lithium salt to generate a solution.

General procedure for aromatic decarboxylative sulfoximination

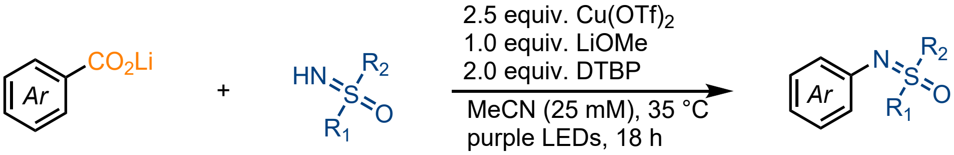

Under nitrogen atmosphere, a 16 mL borosilicate vial equipped with a magnetic stir bar was charged with lithium benzoate (0.200 mmol, 1.00 equiv.), $Cu(OTf)_2$ (0.500 mmol, 2.50 equiv.), LiOMe (0.200 mmol, 1.00 equiv.), 2,6-di-*tert*-butylpyridine (0.400 mmol, 2.00 equiv.) and sulfoximine (0.500 mmol, 2.50 equiv.). Anhydrous MeCN (8.0 mL, c = 25 mM) was then added into the vial. The vial was sealed with a Teflon cap and placed 5 cm away from two purple LEDs (Kessil PR160L-390 nm LEDs). The reaction mixture was irradiated for 18 h while maintaining the temperature at approximately 35 °C through cooling with a fan. After irradiation, the reaction mixture was evaporated under reduced pressure to remove all volatiles. DCM (15 mL) was added to the residue, and the precipitation was removed by filtered through a Büchner funnel. The filtrate was evaporated under reduced pressure, and the residue was purified by chromatography on silica gel. Where necessary, further purification was accomplished by preparative TLC.

NOTE: UV light is harmful to human health, and the operator should wear UV light safety glass when setting up the reaction. For the duration of the reaction, the irradiation setup was covered with aluminium foil to shield the UV light. For simplicity, all reaction components were stored and weighed in a N_2-filled glovebox, though the reactions can be performed outside of a glovebox by using Schlenk techniques to avoid moisture.

Reaction condition optimization

Table S1 Counterion

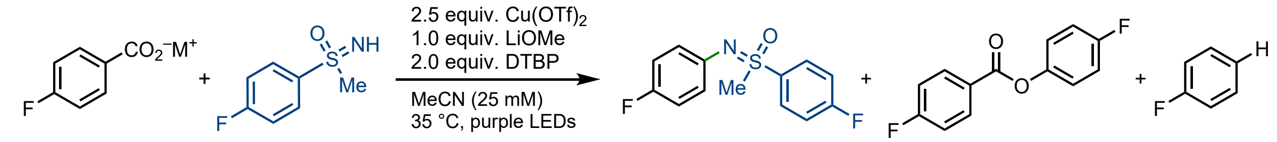

1 0.05 mmol, 1.00 equiv. **2** 2.5 equiv. **3** **3a** **3b**

Counterion M^+	Yield (**3**/**3a**/**3b**, %)[a]	Cu(I) source	Yield (**4**/**4a**/**4b**, %)[a]
H^+	44/8/6	TBA^+	54/4/8
Na^+	60/12/8	Li^+	**66/8/5**
Cs^+	62/8/8	K^+	46/8/6

DTBP: 2,6-di-*tert*-tutylpyridine. [a] ^{19}F NMR yield with 2-fluorotoluene(2.0 equiv.) as an internal standard.

Table S2 Additive optimization

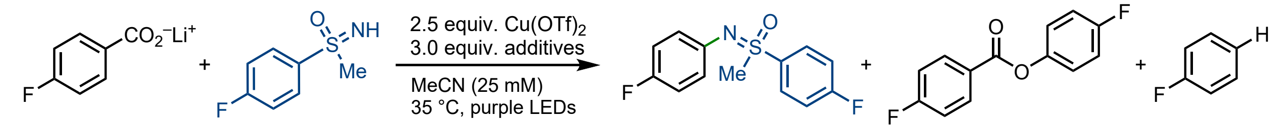

1 0.05 mmol, 1.00 equiv. **2** 2.5 equiv. **3** **3a** **3b**

additive	Yield (**3**/**3a**/**3b**, %)[a]	additive	Yield (**3**/**3a**/**3b**, %)[a]
2,6-di-*tert*-butylpyridine (DTBP)	58/8/8	Cs_2CO_3	0/0/0
2,6-difluoropyridine	22/36/8	LiOH	4/0/2
2,6-lutidine	22/0/4	Li_2CO_3	48/20/8
2-fluoro-6-trifluoromethylpyridine	32/40/8	LiO^{t}Bu	0/0/0
pyridine	54/12/12	LiOMe	0/0/0
quinuclidine	2/0/0	2.0 equiv. DTBP + 1.0	66/8/5

		equiv. LiOMe	
CsF	50/16/8	2.0 equiv. DTBP + 1.0 equiv. LiO^{t}Bu	44/4/8
KF	38/40/10	2.0 equiv. DTBP + 1.0 equiv CsF	30/12/6

[a] ^{19}F NMR yield with 2-fluorotoluene(2.0 equiv.) as an internal standard.

Table S3 Cu(II) salt optimization

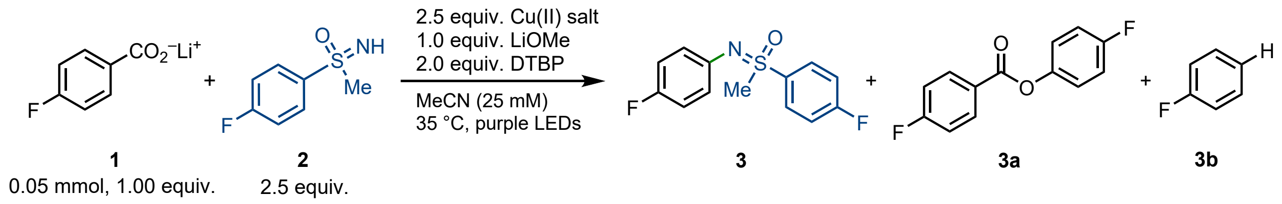

Cu(II) salt	Yield (**3**/**3a**/**3b**, %)[a]	Cu(II) salt	Yield (**3**/**3a**/**3b**, %)[a]
1.1 equiv. $Cu(OTf)_2$	14/0/6	$Cu(BF_4)_2{\cdot}nH_2O$	46/0/10
2.0 equiv. $Cu(OTf)_2$	56/4/4	CuF_2	0/0/0
**2.5 equiv. $Cu(OTf)_2$**	**66/8/5**	$CuSO_4$	0/0/0
$Cu(OAc)_2$	0/0/0	CuO	0/0/0
$Cu(ClO)_4{\cdot}6H_2O$	18/1/5	$CuCl_2$	6/0/0

DTBP: 2,6-di-*tert*-tutylpyridine. [a] Yield was determined by ^{19}F NMR using 2-fluorotoluene(2.0 equiv.) as an internal standard.

Table S4 Solvent optimization

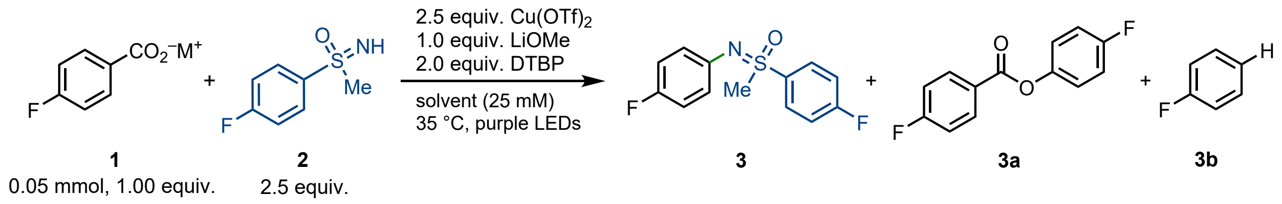

Solvent	Yield (**3**/**3a**/**3b**, %)[a]	Solvent	Yield (**3**/**3a**/**3b**, %)[a]

**MeCN**	**66/8/5**	DCM	0/0/12
DMSO	0/0/0	1,4-dioxane	0/0/0
acetone	0/0/0	toluene	0/0/0
EA	4/0/4	isobutyronitrile	16/8/16
acetone	0/0/0	pivalonitrile	8/4/1
DMF	0/0/0	propionitrile	42/8/20

DTBP: 2,6-di-*tert*-tutylpyridine. [a] ^{19}F NMR yield with 2-fluorotoluene(2.0 equiv.) as an internal standard.

Table S5 Control experiments

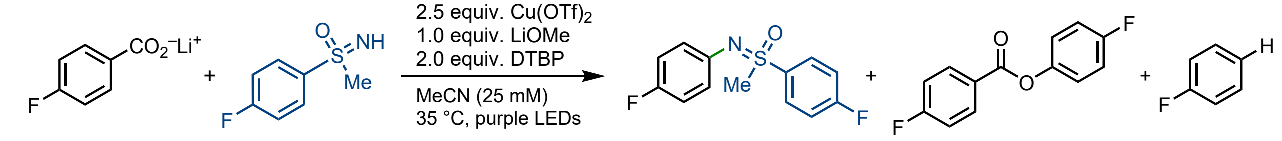

1 0.05 mmol, 1.00 equiv. **2** 2.5 equiv. **3** **3a** **3b**

derivation	Yield (**3**/**3a**/**3b**, %)[a]
none	66/8/5
2,2'-bipyridine instead of DTBP	26/8/6
4,4'-di-tert-butyl-2,2'-bipyridine instead of DTBP	30/0/7
2,2'-6',2''-terpyridine instead of DTBP	35/0/8
(R,R)-2,2'-(2,6-pyridinediyl)bis(4-isopropyl-2-oxazoline) instead of DTBP	45/0/9
100 °C heating instead of irradination	0/0/0
blue LEDs instead of purple LEDs	0/0/0

no $Cu(OTf)_2$	0/0/0
no LiOMe and no DTBP	32/44/12
2.0 equiv. TEMPO as additive	2/0/0
air atmosphere instead of nitrogen atmosphere	52/8/6
$Cu(OTf)_2$ (0.2 equiv.) and 1-fluoro-2,4,6-trimethylpyridinium triflate (2.5 equiv.) instead of $Cu(OTf)_2$ (2.5 equiv.)	2/0/2
$Cu(OTf)_2$ (0.2 equiv.) and $K_2S_2O_8$ (2.5 equiv.) instead of $Cu(OTf)_2$ (2.5 equiv.)	0/0/0
benzoate generated *in situ* by stirring a mixture of 4-fluorobenzoic acid, LiOMe (2.0 equiv.), DTBP (2.0 equiv.) and $Cu(OTf)_2$ for 1 h	54/4/8
benzoate generated *in situ* by stirring a mixture of 4-fluorobenzoic acid, LiOMe (1.0 equiv.), DTBP (2.0 equiv.) and $Cu(OTf)_2$ for 1 h	46/20/8
benzoate generated *in situ* by stirring a mixture of 4-fluorobenzoic acid, Li_2CO_3 (1.0 equiv.), DTBP (2.0 equiv.) and $Cu(OTf)_2$ for 1 h	56/12/10
benzoate generated *in situ* by stirring a mixture of 4-fluorobenzoic acid, DTBP (3.0 equiv.) and $Cu(OTf)_2$ for 1 h	52/16/8

DTBP: 2,6-di-*tert*-tutylpyridine. [a] ^{19}F NMR yield with 2-fluorotoluene(2.0 equiv.) as an internal standard.

Photo-induced LMCT-enabled aromatic decarboxylative sulfoximination

(4-Fluorophenyl)((4-fluorophenyl)imino)(methyl)-λ^6-sulfanone (3)

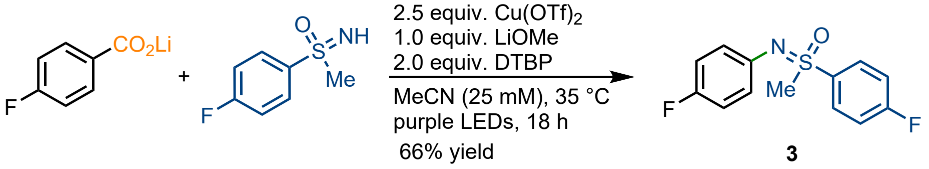

In a nitrogen-filled glovebox, a 16 mL borosilicate vial equipped with a magnetic stir bar was charged with lithium 4-fluorobenzoate (29.2 mg, 0.200 mmol, 1.00 equiv.), $Cu(OTf)_2$ (181 mg, 0.500 mmol, 2.50 equiv.), LiOMe (7.6 mg, 0.200 mmol, 1.00 equiv.), 2,6-di-*tert*-butylpyridine (76.5 mg, 0.400 mmol, 2.00 equiv.) and (4-fluorophenyl)(imino)(methyl)-λ^6-sulfanone (86.5 mg, 0.500 mmol, 2.50 equiv.). Anhydrous MeCN (8.0 mL, c = 25 mM) was then added into the vial. The vial was sealed with a Teflon cap and placed 5 cm away from two

purple LEDs (Kessil PR160L-390 nm LEDs). The reaction mixture was irradiated for 18 h while maintaining the temperature at approximately 35 °C through cooling with a fan. After irradiation, the reaction mixture was evaporated under reduced pressure to remove all volatiles. DCM (15 mL) was added to the residue, and the precipitation was removed by filtered through a Büchner funnel. The filtrate was evaporated under reduced pressure, and the residue was purified by chromatography on silica gel (EA/DCM = 1/50, v/v) to yield (4-fluorophenyl)((4-fluorophenyl)imino)(methyl)-λ6-sulfanone (**3**) (35.4 mg, 132 μmol, 66%) as a slightly yellow oil.

R*f* = 0.17 (DCM).

NMR Spectroscopy:

^{1}**H NMR** (500 MHz, $CDCl_3$, 298 K, δ): 8.03–7.90 (m, 2H), 7.19 (d, *J* = 8.9 Hz, 2H), 6.94 (dd, *J* = 9.0, 4.9 Hz, 2H), 6.80 (t, *J* = 8.8 Hz, 2H), 3.23 (s, 3H) ppm.

^{13}**C NMR** (126 MHz, $CDCl_3$, 298 K, δ): 165.6 (d, *J* = 256.2 Hz), 158.5 (d, *J* = 240.3 Hz), 140.5 (d, *J* = 2.9 Hz), 134.8 (d, *J* = 3.0 Hz), 131.5 (d, *J* = 9.5 Hz), 124.4 (d, *J* = 7.7 Hz), 116.9 (d, *J* = 22.7 Hz), 115.6 (d, *J* = 22.5 Hz), 45.92 ppm.

^{19}**F NMR** (471 MHz, $CDCl_3$, 298 K, δ): –104.3 (m), –122.0 (m) ppm.

HRMS-EI (m/z) calculated for $C_{13}H_{11}NOSF_2^+$ $[M]^+$, 267.0524; found, 267.0529; deviation: –1.94 ppm.

(4-Fluorophenyl)(methyl)((4-(methylsulfonyl)phenyl)imino)-λ6-sulfanone (4)

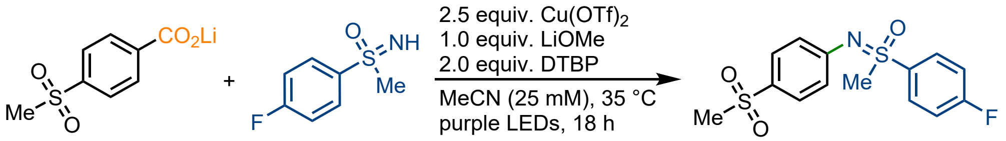

4

In a nitrogen-filled glovebox, a 16 mL borosilicate vial equipped with a magnetic stir bar was charged with lithium 4-(methylsulfonyl)benzoate (41.2 mg, 0.200 mmol, 1.00 equiv.), $Cu(OTf)_2$ (181 mg, 0.500 mmol, 2.50 equiv.), LiOMe (7.6 mg, 0.200 mmol, 1.00 equiv.), 2,6-di-*tert*-butylpyridine (76.5 mg, 0.400 mmol, 2.00 equiv.) and (4-fluorophenyl)(imino)(methyl)-λ6-sulfanone (86.5 mg, 0.500 mmol, 2.50 equiv.). Anhydrous MeCN (8.0 mL, c = 25 mM) was then added into the vial. The vial was sealed with a Teflon cap and placed 5 cm away from two purple LEDs (Kessil PR160L-390 nm LEDs). The reaction mixture was irradiated for 18 h while maintaining the temperature at approximately 35 °C through cooling with a fan. After irradiation, the reaction mixture was evaporated under reduced pressure to remove all volatiles. DCM (15 mL) was added to the residue, and the precipitation was removed by filtered through a Büchner funnel. The filtrate was evaporated under reduced pressure, and the residue was purified by chromatography on silica gel (EA/DCM = 1/50, v/v) to yield (4-fluorophenyl)(methyl)((4-(methylsulfonyl)phenyl)imino)-λ6-sulfanone (**4**) (39.5 mg, 121 μmol, 60%) as a slightly yellow oil.

R*f* = 0.20 (EA/DCM = 1/50, v/v).

NMR Spectroscopy:

^{1}H NMR (500 MHz, $CDCl_3$, 298 K, δ): 7.97–7.92 (m, 2H), 7.69–7.58 (m, 2H), 7.25–7.18 (m, 2H), 7.10–7.04 (m, 2H), 3.28 (s, 3H), 2.96 (s, 3H) ppm.

^{13}C NMR (126 MHz, $CDCl_3$, 298 K, δ): 166.0 (d, J = 256.9 Hz), 150.9, 134.3 (d, J = 3.0 Hz), 132.7, 131.4 (d, J = 9.6 Hz), 128.8, 123.1, 117.3 (d, J = 22.6 Hz), 46.7, 44.8 ppm.

^{19}F NMR (471 MHz, $CDCl_3$, 298 K, δ): –103.1 (m) ppm.

HRMS-EI (m/z) calculated for $C_{14}H_{14}NO_3S_2F^+$ $[M]^+$, 327.0394; found, 327.0394; deviation: –0.19 ppm.

(4-Fluorophenyl)(methyl)((4-(trifluoromethyl)phenyl)imino)-λ⁶-sulfanone (5)

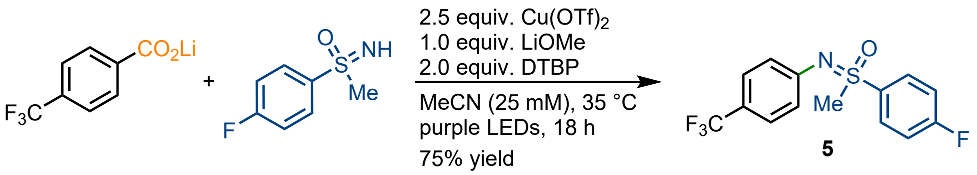

In a nitrogen-filled glovebox, a 16 mL borosilicate vial equipped with a magnetic stir bar was charged with lithium 4-(trifluoromethyl)benzoate (39.2 mg, 0.200 mmol, 1.00 equiv.), $Cu(OTf)_2$ (181 mg, 0.500 mmol, 2.50 equiv.), LiOMe (7.6 mg, 0.200 mmol, 1.00 equiv.), 2,6-di-*tert*-butylpyridine (76.5 mg, 0.400 mmol, 2.00 equiv.) and (4-fluorophenyl)(imino)(methyl)-λ^6-sulfanone (86.5 mg, 0.500 mmol, 2.50 equiv.). Anhydrous MeCN (8.0 mL, c = 25 mM) was then added into the vial. The vial was sealed with a Teflon cap and placed 5 cm away from two purple LEDs (Kessil PR160L-390 nm LEDs). The reaction mixture was irradiated for 18 h while maintaining the temperature at approximately 35 °C through cooling with a fan. After irradiation, the reaction mixture was evaporated under reduced pressure to remove all volatiles. DCM (15 mL) was added to the residue, and the precipitation was removed by filtered through a Büchner funnel. The filtrate was evaporated under reduced pressure, and the residue was purified by chromatography on silica gel (EA/DCM = 1/50, v/v) to yield (4-fluorophenyl)(methyl)((4-(trifluoromethyl)phenyl)imino)-λ^6-sulfanone (**5**) (47.3 mg, 149 μmol, 75%) as a slightly yellow oil.

R*f* = 0.36 (DCM).

NMR Spectroscopy:

^{1}H NMR (500 MHz, $CDCl_3$, 298 K, δ): 7.97–7.92 (m, 2H), 7.69–7.58 (m, 2H), 7.25–7.18 (m, 2H), 7.10–7.04 (m, 2H), 3.28 (s, 3H), 2.96 (s, 3H) ppm.

^{13}C NMR (126 MHz, $CDCl_3$, 298 K, δ): 165.9 (d, J = 256.4 Hz), 148.4, 134.7 (d, J = 3.1 Hz), 131.5 (d, J = 9.5 Hz), 126.4 (q, J = 3.7 Hz), 124.6 (q, J = 269.5 Hz), 123.7 (q, J = 32.6 Hz), 123.0, 117.2 (d, J = 22.8 Hz), 46.6 ppm.

^{19}F NMR (471 MHz, $CDCl_3$, 298 K, δ): –61.7 (s), –103.7 (m) ppm.

HRMS-ESI (m/z) calculated for $C_{14}H_{12}NOSF_4^+$ $[M+H]^+$, 318.0570; found, 318.0572; deviation: –0.58 ppm.

4-(((4-Fluorophenyl)(methyl)(oxo)-λ^6-sulfanylidene)amino)phenyl 4-methylbenzenesulfonate (6)

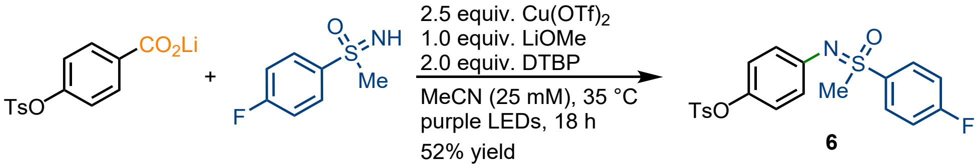

In a nitrogen-filled glovebox, a 16 mL borosilicate vial equipped with a magnetic stir bar was charged with lithium 4-(tosyloxy)benzoate (59.6 mg, 0.200 mmol, 1.00 equiv.), $Cu(OTf)_2$ (181 mg, 0.500 mmol, 2.50 equiv.), LiOMe (7.6 mg, 0.200 mmol, 1.00 equiv.), 2,6-di-*tert*-butylpyridine (76.5 mg, 0.400 mmol, 2.00 equiv.) and (4-fluorophenyl)(imino)(methyl)-λ^6-sulfanone (86.5 mg, 0.500 mmol, 2.50 equiv.). Anhydrous MeCN (8.0 mL, c = 25 mM) was then added into the vial. The vial was sealed with a Teflon cap and placed 5 cm away from two purple LEDs (Kessil PR160L-390 nm LEDs). The reaction mixture was irradiated for 18 h while maintaining the temperature at approximately 35 °C through cooling with a fan. After irradiation, the reaction mixture was evaporated under reduced pressure to remove all volatiles. DCM (15 mL) was added to the residue, and the precipitation was removed by filtered through a Büchner funnel. The filtrate was evaporated under reduced pressure, and the residue was purified by chromatography on silica gel (EA/DCM = 1/50, v/v) to yield 4-(((4-fluorophenyl)(methyl)(oxo)-λ^6-sulfanylidene)amino)phenyl 4-methylbenzenesulfonate (**6**) (43.5 mg, 104 μmol, 52%) as a colorless solid.

R*f* = 0.18 (DCM).

NMR Spectroscopy:

^{1}H NMR (500 MHz, $CDCl_3$, 298 K, δ): 7.97–7.92 (m, 2H), 7.69–7.58 (m, 2H), 7.25–7.18 (m, 2H), 7.10–7.04 (m, 2H), 3.28 (s, 3H), 2.96 (s, 3H) ppm.

^{13}C NMR (126 MHz, $CDCl_3$, 298 K, δ): 165.7 (d, *J* = 256.3 Hz), 144.8 (d, *J* = 122.7 Hz), 144.0, 134.9 (d, *J* = 3.1 Hz), 132.3, 131.5 (d, *J* = 9.5 Hz), 129.7, 128.6, 123.9, 123.0, 117.0 (d, *J* = 22.6 Hz), 46.3, 21.8 ppm.

^{19}F NMR (471 MHz, $CDCl_3$, 298 K, δ): –104.0 (m) ppm.

HRMS-ESI (m/z) calculated for $C_{14}H_{12}NOSF_4^+$ $[M+H]^+$, 318.0570; found, 318.0572; deviation: –0.58 ppm.

((4-Bromophenyl)imino)(4-fluorophenyl)(methyl)-λ^6-sulfanone (7)

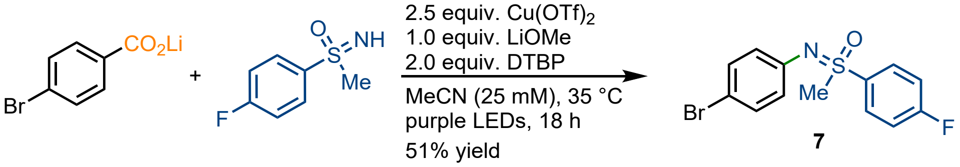

In a nitrogen-filled glovebox, a 16 mL borosilicate vial equipped with a magnetic stir bar was charged with lithium 4-bromobenzoate (41.4 mg, 0.200 mmol, 1.00 equiv.), $Cu(OTf)_2$ (181 mg, 0.500 mmol, 2.50 equiv.), LiOMe (7.6 mg, 0.200 mmol, 1.00 equiv.), 2,6-di-*tert*-butylpyridine (76.5 mg, 0.400 mmol, 2.00 equiv.) and (4-fluorophenyl)(imino)(methyl)-λ^6-sulfanone (86.5 mg, 0.500 mmol, 2.50 equiv.). Anhydrous MeCN (8.0 mL, c = 25 mM) was then added into the vial. The vial was sealed with a Teflon cap and placed 5 cm away from two

purple LEDs (Kessil PR160L-390 nm LEDs). The reaction mixture was irradiated for 18 h while maintaining the temperature at approximately 35 °C through cooling with a fan. After irradiation, the reaction mixture was evaporated under reduced pressure to remove all volatiles. DCM (15 mL) was added to the residue, and the precipitation was removed by filtered through a Büchner funnel. The filtrate was evaporated under reduced pressure, and the residue was purified by chromatography on silica gel (EA/DCM = 1/50, v/v) to yield ((4-bromophenyl)imino)(4-fluorophenyl)(methyl)-λ^6-sulfanone (**7**) (33.6 mg, 102 μmol, 51%) as a colorless oil.

R*f* = 0.45 (EA/DCM = 1/50, v/v).

NMR Spectroscopy:

^{1}H NMR (500 MHz, $CDCl_3$, 298 K, δ): 8.04–7.80 (m, 2H), 7.24–7.15 (m, 4H), 6.96–6.81 (m, 2H), 3.23 (s, 3H) ppm.

^{13}C NMR (126 MHz, $CDCl_3$, 298 K, δ): 165.8 (d, *J* = 256.3 Hz), 144.0, 134.9 (d, *J* = 3.1 Hz), 132.1, 131.5 (d, *J* = 9.6 Hz), 125.0, 117.1 (d, *J* = 22.6 Hz), 114.8, 46.3 ppm.

^{19}F NMR (471 MHz, $CDCl_3$, 298 K, δ): –104.0 (m) ppm.

HRMS-EI (m/z) calculated for $C_{13}H_{11}NOBrSF^+$ $[M]^+$, 326.9723; found, 326.9728; deviation: –1.44 ppm.

(4-Fluorophenyl)((3-(5-(2-fluorophenyl)-1,2,4-oxadiazol-3-yl)phenyl)imino)(methyl)-λ^6-sulfanone (8)

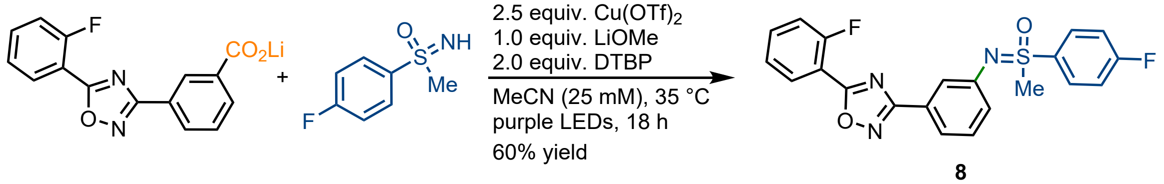

8

In a nitrogen-filled glovebox, a 16 mL borosilicate vial equipped with a magnetic stir bar was charged with lithium salt of ataluren (58.0 mg, 0.200 mmol, 1.00 equiv.), $Cu(OTf)_2$ (181 mg, 0.500 mmol, 2.50 equiv.), LiOMe (7.6 mg, 0.200 mmol, 1.00 equiv.), 2,6-di-*tert*-butylpyridine (76.5 mg, 0.400 mmol, 2.00 equiv.) and (4-fluorophenyl)(imino)(methyl)-λ^6-sulfanone (86.5 mg, 0.500 mmol, 2.50 equiv.). Anhydrous MeCN (8.0 mL, c = 25 mM) was then added into the vial. The vial was sealed with a Teflon cap and placed 5 cm away from two purple LEDs (Kessil PR160L-390 nm LEDs). The reaction mixture was irradiated for 18 h while maintaining the temperature at approximately 35 °C through cooling with a fan. After irradiation, the reaction mixture was evaporated under reduced pressure to remove all volatiles. DCM (15 mL) was added to the residue, and the precipitation was removed by filtered through a Büchner funnel. The filtrate was evaporated under reduced pressure, and the residue was purified by chromatography on silica gel (EA/DCM = 1/20, v/v) to afford a mixture. The mixture was further purified by preparative TLC (EA/DCM = 1/20, v/v) to yield (4-fluorophenyl)((3-(5-(2-fluorophenyl)-1,2,4-oxadiazol-3-yl)phenyl)imino)(methyl)-λ^6-sulfanone (**8**) (49.6 mg, 121 μmol, 60%) as a colorless solid.

R*f* = 0.33 (EA/DCM = 1/19, v/v).

NMR Spectroscopy:

^{1}**H NMR** (500 MHz, $CDCl_3$, 298 K, δ): 8.15–8.07 (m, 1H), 7.98–7.90 (m, 2H), 7.76 (s, 1H), 7.63 (d, *J* = 6.3 Hz, 1H), 7.51 (ddd, *J* = 13.4, 7.0, 1.8 Hz, 1H), 7.33–7.00 (m, 6H), 3.22 (s, 3H) ppm.

^{13}**C NMR** (126 MHz, $CDCl_3$, 298 K, δ): 172.7 (d, *J* = 4.3 Hz), 168.8, 165.8 (d, *J* = 256.2 Hz), 160.9 (d, *J* = 260.3 Hz), 145.4, 135.0, 134.6 (d, *J* = 8.8 Hz), 131.6 (d, *J* = 9.5 Hz), 131.1, 129.7, 127.7, 126.0, 124.8 (d, *J* = 3.7 Hz), 122.7, 121.3, 117.2 (d, *J* = 20.9 Hz), 117.1 (d, *J* = 22.6 Hz), 113.0 (d, *J* = 11.4 Hz), 46.3 ppm.

^{19}**F NMR** (471 MHz, $CDCl_3$, 298 K, δ): –104.2 (m), -108.4 (m) ppm.

HRMS-ESI (m/z) calculated for $C_{21}H_{15}N_3O_2SF_2Na^+$ $[M+Na]^+$, 434.0745; found, 434.0750; deviation: –0.98 ppm.

((4-Cyanophenyl)imino)(4-fluorophenyl)(methyl)-λ⁶-sulfanone (9)

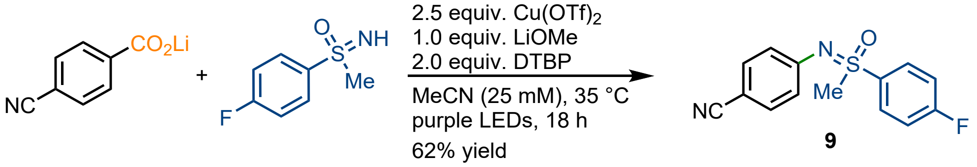

In a nitrogen-filled glovebox, a 16 mL borosilicate vial equipped with a magnetic stir bar was charged with lithium 4-cyanobenzoate (30.6 mg, 0.200 mmol, 1.00 equiv.), $Cu(OTf)_2$ (181 mg, 0.500 mmol, 2.50 equiv.), LiOMe (7.6 mg, 0.200 mmol, 1.00 equiv.), 2,6-di-*tert*-butylpyridine (76.5 mg, 0.400 mmol, 2.00 equiv.) and (4-fluorophenyl)(imino)(methyl)-λ^6-sulfanone (86.5 mg, 0.500 mmol, 2.50 equiv.). Anhydrous MeCN (8.0 mL, c = 25 mM) was then added into the vial. The vial was sealed with a Teflon cap and placed 5 cm away from two purple LEDs (Kessil PR160L-390 nm LEDs). The reaction mixture was irradiated for 18 h while maintaining the temperature at approximately 35 °C through cooling with a fan. After irradiation, the reaction mixture was evaporated under reduced pressure to remove all volatiles. DCM (15 mL) was added to the residue, and the precipitation was removed by filtered through a Büchner funnel. The filtrate was evaporated under reduced pressure, and the residue was purified by chromatography on silica gel (EA/DCM = 1/50, v/v) to yield ((4-cyanophenyl)imino)(4-fluorophenyl)(methyl)-λ^6-sulfanone (**9**) (34.1 mg, 124 µmol, 62%) as a colorless oil.

R*f* = 0.40 (EA/DCM = 1/50, v/v).

NMR Spectroscopy:

^{1}**H NMR** (500 MHz, $CDCl_3$, 298 K, δ): 7.99–7.91 (m, 2H), 7.43–7.34 (m, 2H), 7.26–7.18 (m, 2H), 7.02–6.98 (m, 2H), 3.28 (s, 3H) ppm.

^{13}**C NMR** (126 MHz, $CDCl_3$, 298 K, δ): 166.0 (d, *J* = 257.3 Hz), 149.9, 134.4 (d, *J* = 3.2 Hz), 133.4, 131.4 (d, *J* = 9.7 Hz), 123.3, 119.7, 117.4 (d, *J* = 22.7 Hz), 104.5, 46.8 ppm.

^{19}**F NMR** (471 MHz, $CDCl_3$, 298 K, δ): –103.1 (m) ppm.

HRMS-EI (m/z) calculated for $C_{14}H_{11}N_2OSF^+$ $[M]^+$, 274.0571; found, 274.0574; deviation: –1.23 ppm.

((2-Fluorophenyl)imino)(4-fluorophenyl)(methyl)-λ⁶-sulfanone (10)

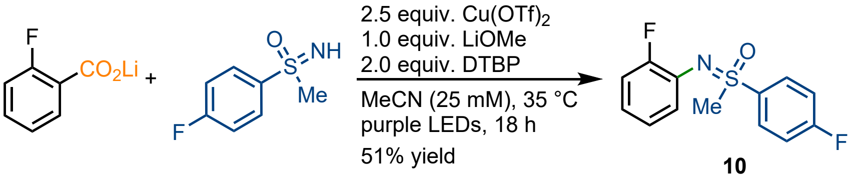

10

In a nitrogen-filled glovebox, a 16 mL borosilicate vial equipped with a magnetic stir bar was charged with lithium 2-fluorobenzoate (29.2 mg, 0.200 mmol, 1.00 equiv.), $Cu(OTf)_2$ (181 mg, 0.500 mmol, 2.50 equiv.), LiOMe (7.6 mg, 0.200 mmol, 1.00 equiv.), 2,6-di-*tert*-butylpyridine (76.5 mg, 0.400 mmol, 2.00 equiv.) and (4-fluorophenyl)(imino)(methyl)-λ^6-sulfanone (86.5 mg, 0.500 mmol, 2.50 equiv.). Anhydrous MeCN (8.0 mL, c = 25 mM) was then added into the vial. The vial was sealed with a Teflon cap and placed 5 cm away from two purple LEDs (Kessil PR160L-390 nm LEDs). The reaction mixture was irradiated for 18 h while maintaining the temperature at approximately 35 °C through cooling with a fan. After irradiation, the reaction mixture was evaporated under reduced pressure to remove all volatiles. DCM (15 mL) was added to the residue, and the precipitation was removed by filtered through a Büchner funnel. The filtrate was evaporated under reduced pressure, and the residue was purified by chromatography on silica gel (EA/DCM = 1/50, v/v) to yield ((2-fluorophenyl)imino)(4-fluorophenyl)(methyl)-λ^6-sulfanone (**10**) (27.4 mg, 103 µmol, 51%) as a colorless oil.

R*f* = 0.37 (EA/DCM = 1/50, v/v).

NMR Spectroscopy:

^{1}H NMR (500 MHz, $CDCl_3$, 298 K, δ): 8.05–7.98 (m, 2H), 7.24–7.17 (m, 2H), 7.14–7.08 (m, 1H), 7.02–6.94 (m, 1H), 6.90–6.81 (m, 2H), 3.27 (s, 3H) ppm.

^{13}C NMR (126 MHz, $CDCl_3$, 298 K, δ): 165.8 (d, *J* = 256.2 Hz), 156.6 (d, *J* = 243.9 Hz), 135.3 (d, *J* = 3.0 Hz), 132.5 (d, *J* = 11.9 Hz), 131.5 (d, *J* = 9.6 Hz), 125.3 (d, *J* = 2.2 Hz), 124.3 (d, *J* = 4.0 Hz), 123.1 (d, *J* = 7.2 Hz), 117.0 (d, *J* = 22.6 Hz), 116.0 (d, *J* = 20.3 Hz), 46.3 ppm.

^{19}F NMR (471 MHz, $CDCl_3$, 298 K, δ): –104.3 (m), –125.6 (m) ppm.

HRMS-EI (m/z) calculated for $C_{13}H_{11}NOSF_2^+$ $[M]^+$, 267.0524; found, 267.0529; deviation: –1.97 ppm.

((3-Methoxyphenyl)imino)(4-fluorophenyl)(methyl)-λ⁶-sulfanone (11)

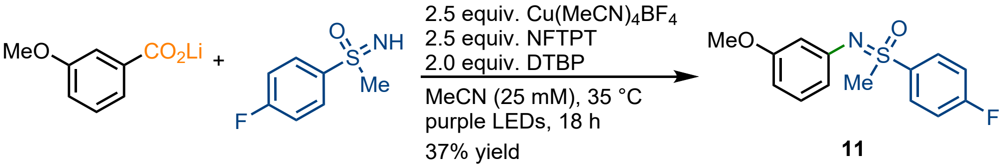

11

In a nitrogen-filled glovebox, a 16 mL borosilicate vial equipped with a magnetic stir bar was charged with lithium 3-methoxybenzoate (29.2 mg, 0.200 mmol, 1.00 equiv.), $Cu(MeCN)_4BF_4$ (157 mg, 0.500 mmol, 2.50 equiv.), 1-fluoro-2,4,6-trimethylpyridinium triflate (145 mg, 0.500 mmol, 2.50 equiv.), 2,6-di-*tert*-butylpyridine (76.5 mg, 0.400 mmol, 2.00 equiv.) and (4-fluorophenyl)(imino)(methyl)-λ^6-sulfanone (86.5 mg, 0.500 mmol,

2.50 equiv.). Anhydrous MeCN (8.0 mL, c = 25 mM) was then added into the vial. The vial was sealed with a Teflon cap and placed 5 cm away from two purple LEDs (Kessil PR160L-390 nm LEDs). The reaction mixture was irradiated for 18 h while maintaining the temperature at approximately 35 °C through cooling with a fan. After irradiation, the reaction mixture was evaporated under reduced pressure to remove all volatiles. The residue was purified by chromatography on silica gel (EA/DCM = 1/50, v/v) to yield ((3-methoxyphenyl)imino)(4-fluorophenyl)(methyl)-λ^6-sulfanone (**11**) (20.7 mg, 74 μmol, 37%) as a slightly yellow oil.

R*f* = 0.15 (EA/DCM = 1/50, v/v).

NMR Spectroscopy:

^{1}**H NMR** (500 MHz, $CDCl_3$, 298 K, δ): 8.00 (dd, *J* = 8.9, 5.0 Hz, 2H), 7.22–7.17 (m, 2H), 7.02 (t, *J* = 8.4 Hz, 1H), 6.62–6.58 (m, 2H), 6.46 (ddd, *J* = 8.3, 2.4, 1.0 Hz, 1H), 3.71 (s, 3H), 3.25 (s, 3H) ppm.

^{13}**C NMR** (126 MHz, $CDCl_3$, 298 K, δ): 165.8 (d, *J* = 256.2 Hz), 160.4, 145.7, 135.1 (d, *J* = 3.0 Hz), 131.6 (d, *J* = 9.5 Hz), 129.8, 117.0 (d, *J* = 22.7 Hz), 115.8, 109.2, 108.2, 55.3, 46.2 ppm.

^{19}**F NMR** (471 MHz, $CDCl_3$, 298 K, δ): –104.4 (m) ppm.

HRMS-EI (m/z) calculated for $C_{14}H_{14}NO_2SF^+$ $[M]^+$, 279.0724; found, 279.0725; deviation: –0.50 ppm.

(4-Fluorophenyl)(methyl)((2-(trifluoromethyl)pyridin-4-yl)imino)-λ^6-sulfanone (12)

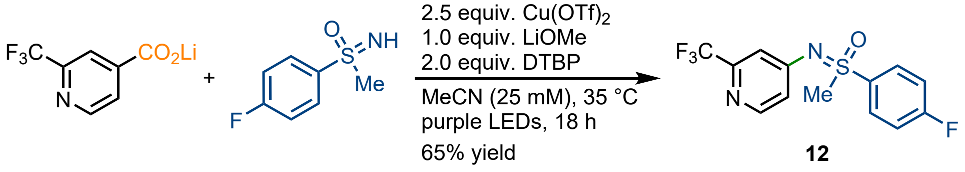

In a nitrogen-filled glovebox, a 16 mL borosilicate vial equipped with a magnetic stir bar was charged with lithium 2-(trifluoromethyl)isonicotinate (39.4 mg, 0.200 mmol, 1.00 equiv.), $Cu(OTf)_2$ (181 mg, 0.500 mmol, 2.50 equiv.), LiOMe (7.6 mg, 0.200 mmol, 1.00 equiv.), 2,6-di-*tert*-butylpyridine (76.5 mg, 0.400 mmol, 2.00 equiv.) and (4-fluorophenyl)(imino)(methyl)-λ^6-sulfanone (86.5 mg, 0.500 mmol, 2.50 equiv.). Anhydrous MeCN (8.0 mL, c = 25 mM) was then added into the vial. The vial was sealed with a Teflon cap and placed 5 cm away from two purple LEDs (Kessil PR160L-390 nm LEDs). The reaction mixture was irradiated for 18 h while maintaining the temperature at approximately 35 °C through cooling with a fan. After irradiation, the reaction mixture was evaporated under reduced pressure to remove all volatiles. The residue was purified by chromatography on silica gel (EA/DCM = 1/50, v/v) to yield (4-fluorophenyl)(methyl)((2-(trifluoromethyl)pyridin-4-yl)imino)-λ^6-sulfanone (**12**) (41.2 mg, 129 μmol, 65%) as a colorless oil.

R*f* = 0.33 (EA/DCM = 1/50, v/v).

NMR Spectroscopy:

^{1}**H NMR** (500 MHz, $CDCl_3$, 298 K, δ): 8.30 (d, *J* = 5.5 Hz, 1H), 7.99–7.92 (m, 2H), 7.28–7.23 (m, 2H), 7.21 (d, *J* = 2.1 Hz, 1H), 6.93 (dd, *J* = 5.5, 2.1 Hz, 1H), 3.31 (s, 3H) ppm.

^{13}**C NMR** (126 MHz, $CDCl_3$, 298 K, δ): 166.2 (d, *J* = 258.0 Hz), 154.3, 150.5, 149.0 (q, *J* = 33.7 Hz), 133.8

(d, J = 3.0 Hz), 131.3 (d, J = 9.7 Hz), 121.7 (q, J = 274.5 Hz), 119.4, 117.6 (d, J = 22.7 Hz), 115.1 (q, J = 2.9 Hz), 46.8 ppm.

^{19}F NMR (471 MHz, $CDCl_3$, 298 K, δ): –68.3 (s), –102.3 (m) ppm.

HRMS-EI (m/z) calculated for $C_{13}H_{10}NOSF_4^+$ $[M]^+$, 318.0444; found, 318.0449; deviation: –1.48 ppm.

((4-Cyanophenyl)imino)(4-fluorophenyl)(methyl)-λ^6-sulfanone (13)

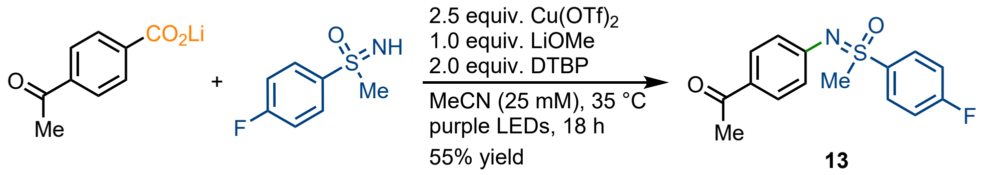

13

In a nitrogen-filled glovebox, a 16 mL borosilicate vial equipped with a magnetic stir bar was charged with lithium 4-acetylbenzoate (34.0 mg, 0.200 mmol, 1.00 equiv.), $Cu(OTf)_2$ (181 mg, 0.500 mmol, 2.50 equiv.), LiOMe (7.6 mg, 0.200 mmol, 1.00 equiv.), 2,6-di-*tert*-butylpyridine (76.5 mg, 0.400 mmol, 2.00 equiv.) and (4-fluorophenyl)(imino)(methyl)-λ^6-sulfanone (86.5 mg, 0.500 mmol, 2.50 equiv.). Anhydrous MeCN (8.0 mL, c = 25 mM) was then added into the vial. The vial was sealed with a Teflon cap and placed 5 cm away from two purple LEDs (Kessil PR160L-390 nm LEDs). The reaction mixture was irradiated for 18 h while maintaining the temperature at approximately 35 °C through cooling with a fan. After irradiation, the reaction mixture was evaporated under reduced pressure to remove all volatiles. DCM (15 mL) was added to the residue, and the precipitation was removed by filtered through a Büchner funnel. The filtrate was evaporated under reduced pressure, and the residue was purified by chromatography on silica gel (EA/DCM = 1/50, v/v) to yield ((4-acetylphenyl)imino)(4-fluorophenyl)(methyl)-λ^6-sulfanone (**13**) (32.3 mg, 111 μmol, 55%) as a colorless oil.

R*f* = 0.17 (EA/DCM = 1/50, v/v).

NMR Spectroscopy:

^{1}H NMR (500 MHz, $CDCl_3$, 298 K, δ): 7.98–7.93 (m, 2H), 7.76–7.71 (m, 2H), 7.24–7.17 (m, 2H), 7.03–6.97 (m, 2H), 3.28 (s, 3H), 2.47 (s, 3H) ppm.

^{13}C NMR (126 MHz, $CDCl_3$, 298 K, δ): 197.1, 165.9 (d, J = 256.4 Hz), 150.2, 134.7 (d, J = 3.1 Hz), 131.5 (d, J = 9.6 Hz), 131.0, 130.0, 122.7, 117.2 (d, J = 22.6 Hz), 46.7, 26.4 ppm.

^{19}F NMR (471 MHz, $CDCl_3$, 298 K, δ): –103.6 (m) ppm.

HRMS-EI (m/z) calculated for $C_{14}H_{11}N_2OSF^+$ $[M]^+$, 274.0571; found, 274.0574; deviation: –1.23 ppm.

(+)-Menthol derivative 14

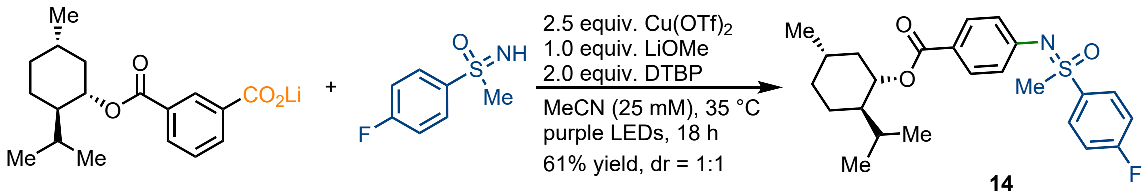

14

In a nitrogen-filled glovebox, a 16 mL borosilicate vial equipped with a magnetic stir bar was charged with lithium salt of (+)-menthol derived benzoic acid (62.1 mg, 0.200 mmol, 1.00 equiv.), $Cu(OTf)_2$ (181 mg, 0.500 mmol, 2.50 equiv.), LiOMe (7.6 mg, 0.200 mmol, 1.00 equiv.), 2,6-di-*tert*-butylpyridine (76.5 mg, 0.400 mmol, 2.00 equiv.) and (4-fluorophenyl)(imino)(methyl)-λ^6-sulfanone (86.5 mg, 0.500 mmol, 2.50 equiv.). Anhydrous MeCN (8.0 mL, c = 25 mM) was then added into the vial. The vial was sealed with a Teflon cap and placed 5 cm away from two purple LEDs (Kessil PR160L-390 nm LEDs). The reaction mixture was irradiated for 18 h while maintaining the temperature at approximately 35 °C through cooling with a fan. After irradiation, the reaction mixture was evaporated under reduced pressure to remove all volatiles. DCM (15 mL) was added to the residue, and the precipitation was removed by filtered through a Büchner funnel. The filtrate was evaporated under reduced pressure, and the residue was purified by chromatography on silica gel (EA/DCM = 1/50, v/v) to yield (+)-menthol derivative **14** (a mixture of two diastereomers, dr = 1:1, 52.4 mg, 121 μmol, 61%) as a slightly yellow oil.

R*f* = 0.16 (EA/DCM = 1/50, v/v).

NMR Spectroscopy:

^{1}H NMR (500 MHz, $CDCl_3$, 298 K, δ): 8.02–7.93 (m, 2H), 7.64 (d, *J* = 6.4 Hz, 1H), 7.59–7.51 (m, 1H), 7.23–7.12 (m, 4H), 4.86 (t, *J* = 10.8 Hz, 1H), 3.26 (t, *J* = 2.1 Hz, 3H), 2.07 (d, *J* = 12.1 Hz, 1H), 1.97–1.84 (m, 1H), 1.70 (d, *J* = 12.1 Hz, 2H), 1.57–1.47 (m, 2H), 1.14–0.98 (m, 2H), 0.93–0.83 (m, 7H), 0.77 (d, *J* = 8.5 Hz, 1.5H), 0.73 (d, *J* = 8.5 Hz, 1.5 H) ppm.

^{13}C NMR (126 MHz, $CDCl_3$, 298 K, δ): 166.1, 116.1, 165.8 (d, *J* = 256.3 Hz), 144.9, 144.8, 135.0 (d, *J* = 3.0 Hz), 134.8 (d, *J* = 3.0 Hz), 131.9, 131.9, 131.6 (d, *J* = 9.5 Hz), 131.6 (d, *J* = 9.5 Hz), 129.0, 129.0, 127.3, 127.3, 124.7, 124.3, 123.2, 123.2, 117.1 (d, *J* = 22.5), 117.1 (d, *J* = 22.5), 74.8, 74.8, 47.3, 47.3, 46.4, 46.2 , 41.0, 34.4, 34.4, 31.5, 26.5, 26.5, 23.7, 23.7, 22.1, 20.9, 20.9, 16.6, 116.6 ppm.

^{19}F NMR (471 MHz, $CDCl_3$, 298 K, δ): –104.1 (m), –104.2 (m) ppm.

HRMS-EI (m/z) calculated for $C_{24}H_{30}NO_3SF^+$ $[M]^+$, 431.1925; found, 431.1933; deviation: –1.84 ppm.

((3-Methylphenyl)imino)(4-fluorophenyl)(methyl)-λ^6-sulfanone (15)

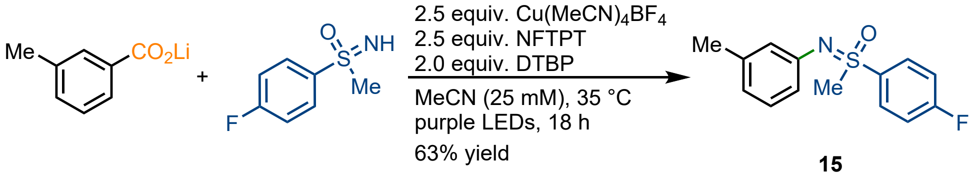

15

In a nitrogen-filled glovebox, a 16 mL borosilicate vial equipped with a magnetic stir bar was charged with lithium 3-methylbenzoate (28.4 mg, 0.200 mmol, 1.00 equiv.), $Cu(MeCN)_4BF_4$ (157 mg, 0.500 mmol, 2.50 equiv.), 1-fluoro-2,4,6-trimethylpyridinium triflate (145 mg, 0.500 mmol, 2.50 equiv.), 2,6-di-*tert*-butylpyridine (76.5 mg, 0.400 mmol, 2.00 equiv.) and (4-fluorophenyl)(imino)(methyl)-λ^6-sulfanone (86.5 mg, 0.500 mmol, 2.50 equiv.). Anhydrous MeCN (8.0 mL, c = 25 mM) was then added into the vial. The vial was sealed with a Teflon cap and placed 5 cm away from two purple LEDs (Kessil PR160L-390 nm LEDs). The reaction mixture

was irradiated for 18 h while maintaining the temperature at approximately 35 °C through cooling with a fan. After irradiation, the reaction mixture was evaporated under reduced pressure to remove all volatiles. The residue was purified by chromatography on silica gel (EA/DCM = 1/50, v/v) to yield ((3-methylphenyl)imino)(4-fluorophenyl)(methyl)-λ^6-sulfanone (**15**) (33.2 mg, 126 μmol, 63%) as a slightly yellow oil.

R*f* = 0.25 (EA/DCM = 1/50, v/v).

NMR Spectroscopy:

^{1}**H NMR** (500 MHz, $CDCl_3$, 298 K, δ): 8.03–7.96 (m, 2H), 7.24–7.15 (m, 2H), 7.02 (t, *J* = 7.7 Hz, 1H), 6.86 (d, *J* = 1.8 Hz, 1H), 6.81 (dd, *J* = 8.0, 2.2 Hz, 1H), 6.72 (d, *J* = 7.5 Hz, 1H), 3.26 (s, 3H), 2.22 (s, 3H) ppm.

^{13}**C NMR** (126 MHz, $CDCl_3$, 298 K, δ): 165.8 (d, *J* = 255.7 Hz), 144.2, 139.0, 135.2 (d, *J* = 3.2 Hz), 131.6 (d, *J* = 9.6 Hz), 129.0, 124.2, 123.2 , 120.4, 117.0 (d, *J* = 22.7 Hz), 46.2, 21.5 ppm.

^{19}**F NMR** (471 MHz, $CDCl_3$, 298 K, δ): –104.6 (m) ppm.

HRMS-EI (m/z) calculated for $C_{14}H_{14}NOSF^+$ $[M]^+$, 263.0775; found, 263.0778; deviation: –1.27 ppm.

((3,5-Dimethylphenyl)imino)(4-fluorophenyl)(methyl)-λ^6-sulfanone (16)

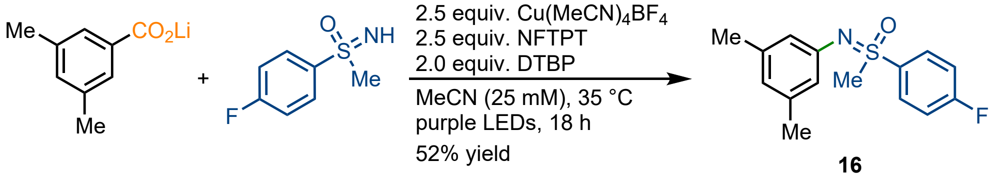

In a nitrogen-filled glovebox, a 16 mL borosilicate vial equipped with a magnetic stir bar was charged with lithium 3,5-dimethylbenzoate (31.2 mg, 0.200 mmol, 1.00 equiv.), $Cu(MeCN)_4BF_4$ (157 mg, 0.500 mmol, 2.50 equiv.), 1-fluoro-2,4,6-trimethylpyridinium triflate (145 mg, 0.500 mmol, 2.50 equiv.), 2,6-di-*tert*-butylpyridine (76.5 mg, 0.400 mmol, 2.00 equiv.) and (4-fluorophenyl)(imino)(methyl)-λ^6-sulfanone (86.5 mg, 0.500 mmol, 2.50 equiv.). Anhydrous MeCN (8.0 mL, c = 25 mM) was then added into the vial. The vial was sealed with a Teflon cap and placed 5 cm away from two purple LEDs (Kessil PR160L-390 nm LEDs). The reaction mixture was irradiated for 18 h while maintaining the temperature at approximately 35 °C through cooling with a fan. After irradiation, the reaction mixture was evaporated under reduced pressure to remove all volatiles. The residue was purified by chromatography on silica gel (EA/DCM = 1/50, v/v) to yield ((3,5-dimethylphenyl)imino)(4-fluorophenyl)(methyl)-λ^6-sulfanone (**16**) (28.8 mg, 104 μmol, 52%) as a slightly yellow oil.

R*f* = 0.23 (EA/DCM = 1/50, v/v).

NMR Spectroscopy:

^{1}**H NMR** (500 MHz, $CDCl_3$, 298 K, δ): 8.03–7.96 (m, 2H), 7.23–7.17 (m, 2H), 6.66 (dt, *J* = 1.5, 0.8 Hz, 2H), 6.56 (dp, *J* = 1.6, 0.7 Hz, 1H), 3.24 (s, 3H), 2.18 (s, 6H) ppm.

^{13}**C NMR** (126 MHz, $CDCl_3$, 298 K, δ): 165.7 (d, *J* = 255.7 Hz), 143.9, 138.7, 135.2, 131.6 (d, *J* = 9.5 Hz),

124.3, 121.2, 117.0 (d, J = 22.6 Hz), 46.1, 21.4 ppm.

^{19}F NMR (471 MHz, $CDCl_3$, 298 K, δ): –104.6 (m) ppm.

HRMS-ESI (m/z) calculated for $C_{15}H_{16}NOSFNa^+$ $[M+Na]^+$, 300.0829; found, 300.0827; deviation: 0.58 ppm.

(4-Fluorophenyl)(methyl)((4-methyl-3-(morpholinosulfonyl)phenyl)imino)-λ^6-sulfanone (17)

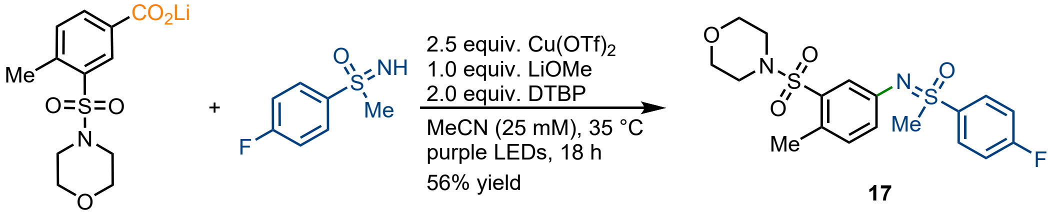

In a nitrogen-filled glovebox, a 16 mL borosilicate vial equipped with a magnetic stir bar was charged with lithium 4-methyl-3-(morpholinosulfonyl)benzoate (58.2 mg, 0.200 mmol, 1.00 equiv.), $Cu(OTf)_2$ (181 mg, 0.500 mmol, 2.50 equiv.), LiOMe (7.6 mg, 0.200 mmol, 1.00 equiv.), 2,6-di-*tert*-butylpyridine (76.5 mg, 0.400 mmol, 2.00 equiv.) and (4-fluorophenyl)(imino)(methyl)-λ^6-sulfanone (86.5 mg, 0.500 mmol, 2.50 equiv.). Anhydrous MeCN (8.0 mL, c = 25 mM) was then added into the vial. The vial was sealed with a Teflon cap and placed 5 cm away from two purple LEDs (Kessil PR160L-390 nm LEDs). The reaction mixture was irradiated for 18 h while maintaining the temperature at approximately 35 °C through cooling with a fan. After irradiation, the reaction mixture was evaporated under reduced pressure to remove all volatiles. DCM (15 mL) was added to the residue, and the precipitation was removed by filtered through a Büchner funnel. The filtrate was evaporated under reduced pressure, and the residue was purified by chromatography on silica gel (EA/DCM = 1/5, v/v) to yield (4-fluorophenyl)(methyl)((4-methyl-3-(morpholinosulfonyl)phenyl)imino)-λ^6-sulfanone (**17**) (46.5 mg, 113 μmol, 56%) as a colorless solid.

R*f* = 0.30 (EA/DCM = 1/4, v/v).

NMR Spectroscopy:

^{1}H NMR (500 MHz, $CDCl_3$, 298 K, δ): 7.99–7.93 (m, 2H), 7.46 (d, J = 2.2 Hz, 1H), 7.21 (t, J = 8.5 Hz, 2H), 7.10–7.03 (m, 2H), 3.67 (t, J = 4.7 Hz, 4H), 3.27 (s, 3H), 3.03 (td, J = 4.3, 2.5 Hz, 4H), 2.47 (s, 3H) ppm.

^{13}C NMR (126 MHz, $CDCl_3$, 298 K, δ): 165.9 (d, J = 256.8 Hz), 135.3, 134.6, 133.7, 131.6 (d, J = 9.5 Hz), 130.9, 127.5, 124.9, 117.2 (d, J = 22.7 Hz), 66.4, 46.3, 45.5, 20.2 ppm.

^{19}F NMR (471 MHz, $CDCl_3$, 298 K, δ): –103.6 (m) ppm.

HRMS-ESI (m/z) calculated for $C_{18}H_{21}N_2O_4S_2FNa^+$ $[M+Na]^+$, 435.0819; found, 435.0823; deviation: –0.90 ppm.

2,2'-(5-(((4-Fluorophenyl)(methyl)(oxo)-λ^6-sulfanylidene)amino)-1,3-phenylene)bis(2-methylpropanenitrile) (18)

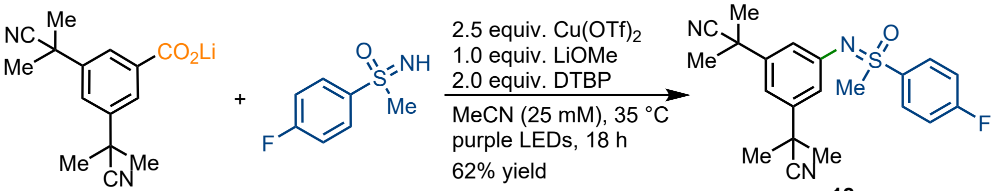

In a nitrogen-filled glovebox, a 16 mL borosilicate vial equipped with a magnetic stir bar was charged with lithium 3,5-bis(2-cyanopropan-2-yl)benzoate (52.4 mg, 0.200 mmol, 1.00 equiv.), $Cu(OTf)_2$ (181 mg, 0.500 mmol, 2.50 equiv.), LiOMe (7.6 mg, 0.200 mmol, 1.00 equiv.), 2,6-di-*tert*-butylpyridine (76.5 mg, 0.400 mmol, 2.00 equiv.) and (4-fluorophenyl)(imino)(methyl)-λ^6-sulfanone (86.5 mg, 0.500 mmol, 2.50 equiv.). Anhydrous MeCN (8.0 mL, c = 25 mM) was then added into the vial. The vial was sealed with a Teflon cap and placed 5 cm away from two purple LEDs (Kessil PR160L-390 nm LEDs). The reaction mixture was irradiated for 18 h while maintaining the temperature at approximately 35 °C through cooling with a fan. After irradiation, the reaction mixture was evaporated under reduced pressure to remove all volatiles. DCM (15 mL) was added to the residue, and the precipitation was removed by filtered through a Büchner funnel. The filtrate was evaporated under reduced pressure, and the residue was purified by chromatography on silica gel (EA/DCM = 1/50 to 1/30, v/v) to yield 2,2'-(5-(((4-fluorophenyl)(methyl)(oxo)-λ^6-sulfanylidene)amino)-1,3-phenylene)bis(2-methylpropanenitrile) (**18**) (47.5 mg, 124 μmol, 62%) as a colorless oil.

R*f* = 0.17 (EA/DCM = 1/50, v/v).

NMR Spectroscopy:

^{1}H NMR (500 MHz, $CDCl_3$, 298 K, δ): 7.99 (dd, J = 8.9, 5.0 Hz, 2H), 7.23 (t, *J* = 8.5 Hz, 2H), 7.06 (t, *J* = 1.8 Hz, 1H), 7.01 (d, *J* = 1.8 Hz, 2H), 3.29 (s, 3H), 1.64 (s, 6H), 1.61 (s, 6H) ppm.

^{13}C NMR (126 MHz, $CDCl_3$, 298 K, δ): 165.9 (d, *J* = 256.4 Hz), 146.1, 143.2, 134.7, 131.5 (d, *J* = 9.5 Hz), 124.4, 119.5, 117.2 (d, *J* = 22.7 Hz), 115.3, 46.3, 37.2, 29.2, 29.0 ppm.

^{19}F NMR (471 MHz, $CDCl_3$, 298 K, δ): –103.7 (m) ppm.

HRMS-ESI (m/z) calculated for $C_{21}H_{22}N_3OSFNa^+$ $[M+Na]^+$, 406.1360; found, 406.1361; deviation: –0.39 ppm.

Celecoxib analogue 19

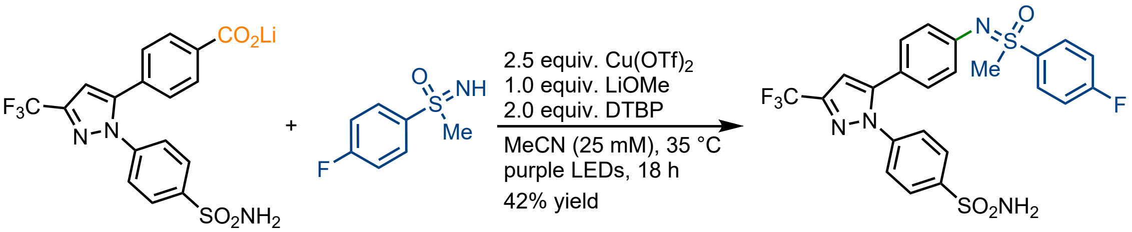

19

In a nitrogen-filled glovebox, a 16 mL borosilicate vial equipped with a magnetic stir bar was charged with

lithium salt of carboxycelecoxib (83.5 mg, 0.200 mmol, 1.00 equiv.), $Cu(OTf)_2$ (181 mg, 0.500 mmol, 2.50 equiv.), LiOMe (7.6 mg, 0.200 mmol, 1.00 equiv.), 2,6-di-*tert*-butylpyridine (76.5 mg, 0.400 mmol, 2.00 equiv.) and (4-fluorophenyl)(imino)(methyl)-λ^6-sulfanone (86.5 mg, 0.500 mmol, 2.50 equiv.). Anhydrous MeCN (8.0 mL, c = 25 mM) was then added into the vial. The vial was sealed with a Teflon cap and placed 5 cm away from two purple LEDs (Kessil PR160L-390 nm LEDs). The reaction mixture was irradiated for 18 h while maintaining the temperature at approximately 35 °C through cooling with a fan. After irradiation, the reaction mixture was evaporated under reduced pressure to remove all volatiles. DCM (15 mL) was added to the residue, and the precipitation was removed by filtered through a Büchner funnel. The filtrate was evaporated under reduced pressure, and the residue was purified by chromatography on silica gel (EA/DCM = 1/2, v/v) to yield a mixture. The mixture was further purified by preparative TLC (EA/DCM = 1/2, v/v) to yield celecoxib analogue **19** (45.1 mg, 84 μmol, 42%) as a colorless oil.

R*f* = 0.40 (EA/DCM = 1/2, v/v).

NMR Spectroscopy:

^{1}H NMR (600 MHz, CD_2Cl_2, 298 K, δ): 7.95 (dd, *J* = 8.9, 5.0 Hz, 2H), 7.87–7.82 (m, 2H), 7.45–7.39 (m, 2H), 7.28–7.21 (m, 2H), 7.01–6.96 (m, 2H), 6.94–6.89 (m, 2H), 6.67 (d, *J* = 0.6 Hz, 1H), 5.15 (s, 2H), 3.25 (s, 3H) ppm.

^{13}C NMR (151 MHz, CD_2Cl_2, 298 K, δ): 166.1 (d, *J* = 255.2 Hz), 147.1, 145.8, 144.0 (q, *J* = 38.2 Hz), 143.0, 141.9, 135.2 (d, *J* = 2.9 Hz), 131.8 (d, *J* = 9.6 Hz), 130.1, 127.7, 125.9, 123.7, 122.7 (q, *J* = 269.0 Hz), 121.9, 117.3 (d, *J* = 22.8 Hz), 106.2 (q, *J* = 1.9 Hz), 46.6. ppm.

^{19}F NMR (565 MHz, CD_2Cl_2, 298 K, δ):–62.8 (s), –105.0 (m) ppm.

HRMS-ESI (m/z) calculated for $C_{23}H_{18}N_4O_3S_2F_4Na^+$ $[M+Na]^+$, 561.0649; found, 561.0648; deviation: 0.16 ppm.

Triclosan derivative 20

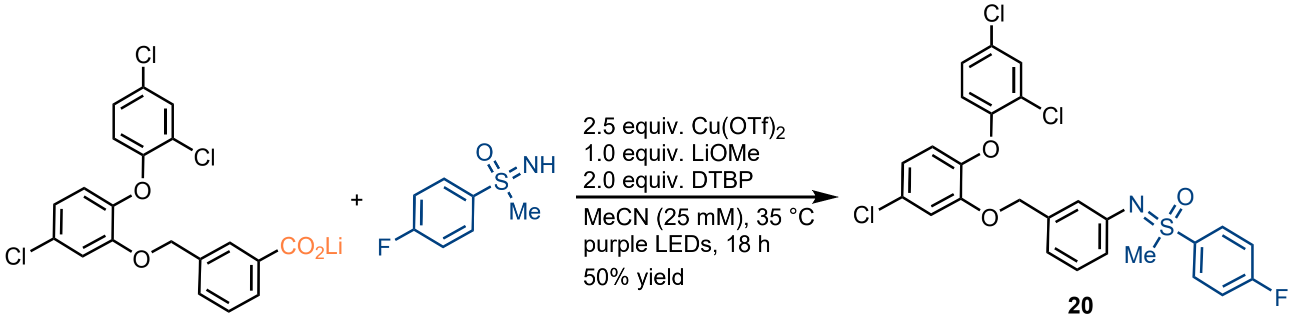

In a nitrogen-filled glovebox, a 16 mL borosilicate vial equipped with a magnetic stir bar was charged with lithium salt of triclosan-derived benzoic acid (52.4 mg, 0.200 mmol, 1.00 equiv.), $Cu(OTf)_2$ (181 mg, 0.500 mmol, 2.50 equiv.), LiOMe (7.6 mg, 0.200 mmol, 1.00 equiv.), 2,6-di-*tert*-butylpyridine (76.5 mg, 0.400 mmol, 2.00 equiv.) and (4-fluorophenyl)(imino)(methyl)-λ^6-sulfanone (86.5 mg, 0.500 mmol, 2.50 equiv.). Anhydrous MeCN (8.0 mL, c = 25 mM) was then added into the vial. The vial was sealed with a Teflon cap and placed 5 cm away from two purple LEDs (Kessil PR160L-390 nm LEDs). The reaction mixture was irradiated for 18 h

while maintaining the temperature at approximately 35 °C through cooling with a fan. After irradiation, the reaction mixture was evaporated under reduced pressure to remove all volatiles. DCM (15 mL) was added to the residue, and the precipitation was removed by filtered through a Büchner funnel. The filtrate was evaporated under reduced pressure, and the residue was purified by chromatography on silica gel (EA/DCM = 1/50, v/v) to yield triclosan derivative **20** (55.3 mg, 100 μmol, 50%) as a colorless solid.

R*f* = 0.27 (DCM).

NMR Spectroscopy:

^{1}**H NMR** (500 MHz, $CDCl_3$, 298 K, δ): 7.93 (dd, *J* = 8.9, 5.0 Hz, 2H), 7.41 (d, *J* = 2.6 Hz, 1H), 7.20–7.14 (m, 2H), 7.12 (dd, *J* = 8.9, 2.6 Hz, 1H), 7.06 (t, *J* = 7.7 Hz, 1H), 6.90 (d, *J* = 15.3 Hz, 5H), 6.72 (d, *J* = 7.8 Hz, 1H), 6.67 (d, *J* = 8.7 Hz, 1H), 4.95 (s, 2H), 3.23 (s, 3H) ppm.

^{13}**C NMR** (126 MHz, $CDCl_3$, 298 K, δ): 165.7 (d, *J* = 255.8 Hz), 152.4, 150.6, 145.2, 143.5, 137.0, 135.1 (d, *J* = 3.3 Hz), 131.5 (d, *J* = 9.5 Hz), 130.5, 130.3, 129.4, 128.0, 127.9, 124.7, 122.9, 122.0, 122.0, 121.5, 120.6, 118.4, 117.0 (d, *J* = 22.7 Hz), 115.7, 71.0, 46.3 ppm.

^{19}**F NMR** (471 MHz, $CDCl_3$, 298 K, δ): –104.3 (m) ppm.

HRMS-ESI (m/z) calculated for $C_{26}H_{19}Cl_3FNO_3SNa^+$ $[M+Na]^+$, 572.0027; found, 572.0031; deviation: –0.65 ppm.

(*R*)-Methyl(phenyl)((4-(trifluoromethyl)phenyl)imino)-λ^6-sulfanone (21)

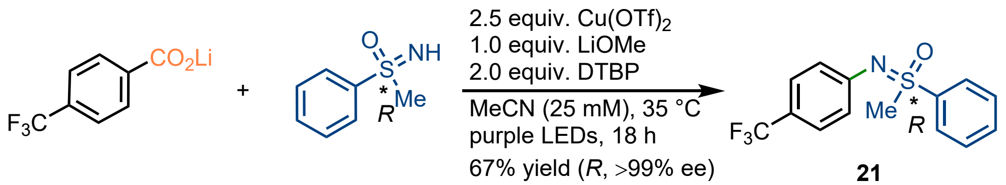

21

In a nitrogen-filled glovebox, a 16 mL borosilicate vial equipped with a magnetic stir bar was charged with lithium 4-(trifluoromethyl)benzoate (39.2 mg, 0.200 mmol, 1.00 equiv.), $Cu(OTf)_2$ (181 mg, 0.500 mmol, 2.50 equiv.), LiOMe (7.6 mg, 0.200 mmol, 1.00 equiv.), 2,6-di-*tert*-butylpyridine (76.5 mg, 0.400 mmol, 2.00 equiv.) and (*R*)-imino(methyl)(phenyl)-λ^6-sulfanone (77.6 mg, 0.500 mmol, 2.50 equiv.). Anhydrous MeCN (8.0 mL, c = 25 mM) was then added into the vial. The vial was sealed with a Teflon cap and placed 5 cm away from two purple LEDs (Kessil PR160L-390 nm LEDs). The reaction mixture was irradiated for 18 h while maintaining the temperature at approximately 35 °C through cooling with a fan. After irradiation, the reaction mixture was evaporated under reduced pressure to remove all volatiles. DCM (15 mL) was added to the residue, and the precipitation was removed by filtered through a Büchner funnel. The filtrate was evaporated under reduced pressure, and the residue was purified by chromatography on silica gel (EA/DCM = 1/50, v/v) to yield (*R*)-methyl(phenyl)((4-(trifluoromethyl)phenyl)imino)-λ^6-sulfanone (**21**) (40.2 mg, 134 μmol, 67%) as a colorless oil.

R*f* = 0.50 (EA/DCM = 1/50, v/v).

ee > 99% (determined by comparison with a racemic sample)

HPLC Daicel Chiralpak AD-3, n-heptane/IPA = 95/5, 1 mL/min, 25 °C, 220 nm

Racemic sample:

<Chromatogram>

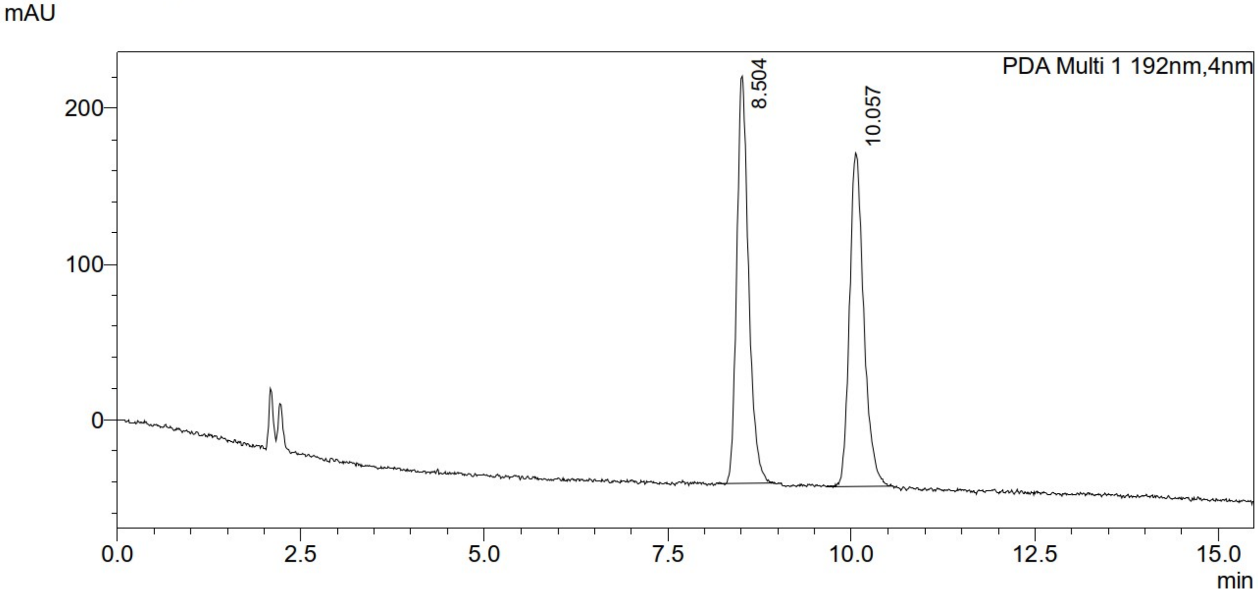

<Peak Table>

PDA Ch1 192nm

Peak#	Ret. Time	Area	Height	Area%
1	8.504	2949405	261359	50.379
2	10.057	2905042	214217	49.621
Total		5854447	475576	100.000

(*R*)-Methyl(phenyl)((4-(trifluoromethyl)phenyl)imino)-λ^6-sulfanone (**21**)

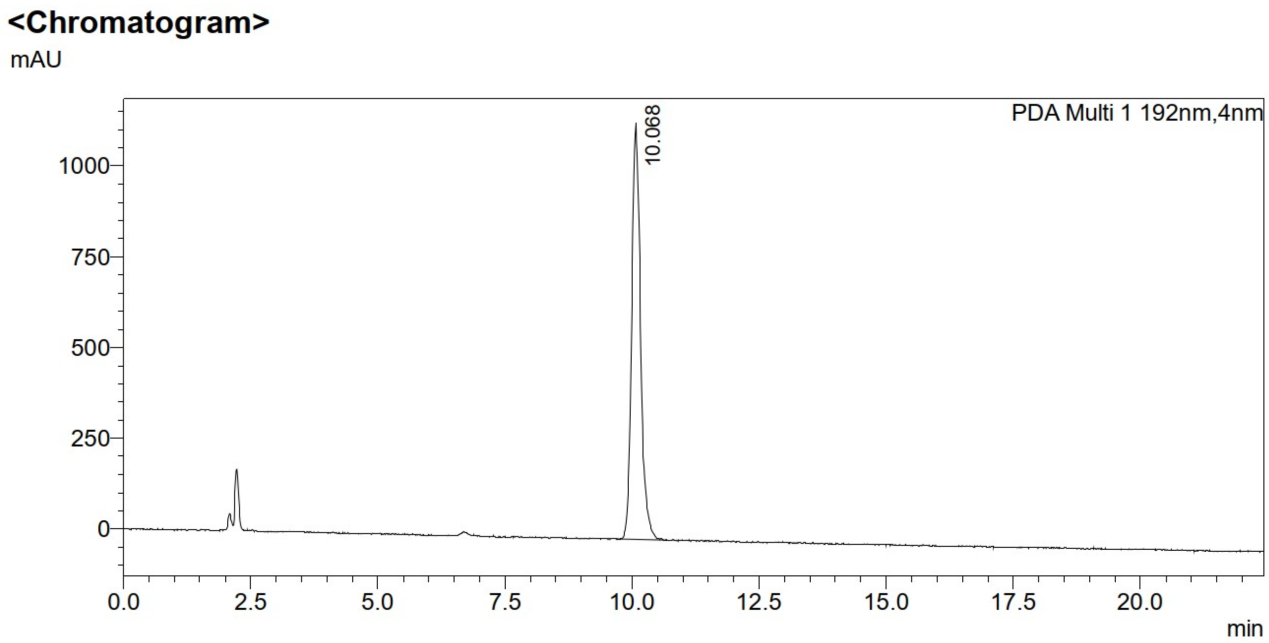

<Peak Table>

PDA Ch1 192nm

Peak#	Ret. Time	Area%	Height%	Area
1	10.068	100.000	100.000	13891000
Total		100.000	100.000	13891000

NMR Spectroscopy:

^{1}H NMR (500 MHz, $CDCl_3$, 298 K, δ): 8.01–7.87 (m, 2H), 7.65–7.58 (m, 1H), 7.58–7.49 (m, 2H), 7.38–7.31 (m, 2H), 7.09–7.00 (m, 2H), 3.26 (s, 3H) ppm.

^{13}C NMR (126 MHz, $CDCl_3$, 298 K, δ): 148.8, 138.9, 133.7, 129.9, 128.6, 126.3 (q, *J* = 4.0 Hz), 124.7 (q, *J* = 269.4 Hz), 123.3 (q, *J* = 32.2 Hz), 122.9, 46.4 ppm.

^{19}F NMR (471 MHz, $CDCl_3$, 298 K, δ): –61.7 (s) ppm.

HRMS-ESI (m/z) calculated for $C_{14}H_{12}NOSF_3Na^+$ $[M+Na]^+$, 322.0484; found, 322.0485; deviation: –0.31 ppm.

(*S*)-Methyl(phenyl)((4-(trifluoromethyl)phenyl)imino)-λ^6-sulfanone (22)

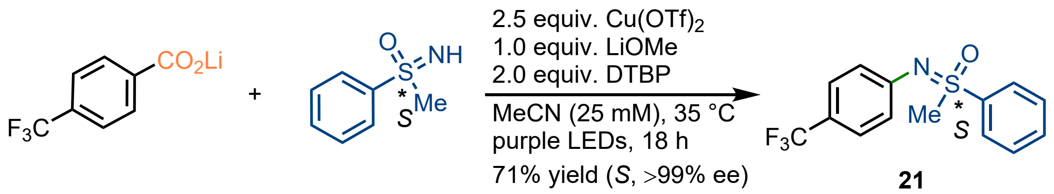

In a nitrogen-filled glovebox, a 16 mL borosilicate vial equipped with a magnetic stir bar was charged with lithium 4-(trifluoromethyl)benzoate (39.2 mg, 0.200 mmol, 1.00 equiv.), $Cu(OTf)_2$ (181 mg, 0.500 mmol, 2.50 equiv.), LiOMe (7.6 mg, 0.200 mmol, 1.00 equiv.), 2,6-di-*tert*-butylpyridine (76.5 mg, 0.400 mmol, 2.00 equiv.) and (*S*)-imino(methyl)(phenyl)-λ^6-sulfanone (77.6 mg, 0.500 mmol, 2.50 equiv.). Anhydrous MeCN (8.0 mL, c = 25 mM) was then added into the vial. The vial was sealed with a Teflon cap and placed 5 cm away from two

purple LEDs (Kessil PR160L-390 nm LEDs). The reaction mixture was irradiated for 18 h while maintaining the temperature at approximately 35 °C through cooling with a fan. After irradiation, the reaction mixture was evaporated under reduced pressure to remove all volatiles. DCM (15 mL) was added to the residue, and the precipitation was removed by filtered through a Büchner funnel. The filtrate was evaporated under reduced pressure, and the residue was purified by chromatography on silica gel (EA/DCM = 1/50, v/v) to yield (*S*)-methyl(phenyl)((4-(trifluoromethyl)phenyl)imino)-λ^6-sulfanone (**22**) (42.3 mg, 141 μmol, 71%) as a colorless oil.

R*f* = 0.50 (EA/DCM = 1/50, v/v).

ee > 99% (determined by comparison with a racemic sample)

HPLC Daicel Chiralpak AD-3, n-heptane/IPA = 95/5, 1 mL/min, 25 °C, 220 nm

Racemic sample:

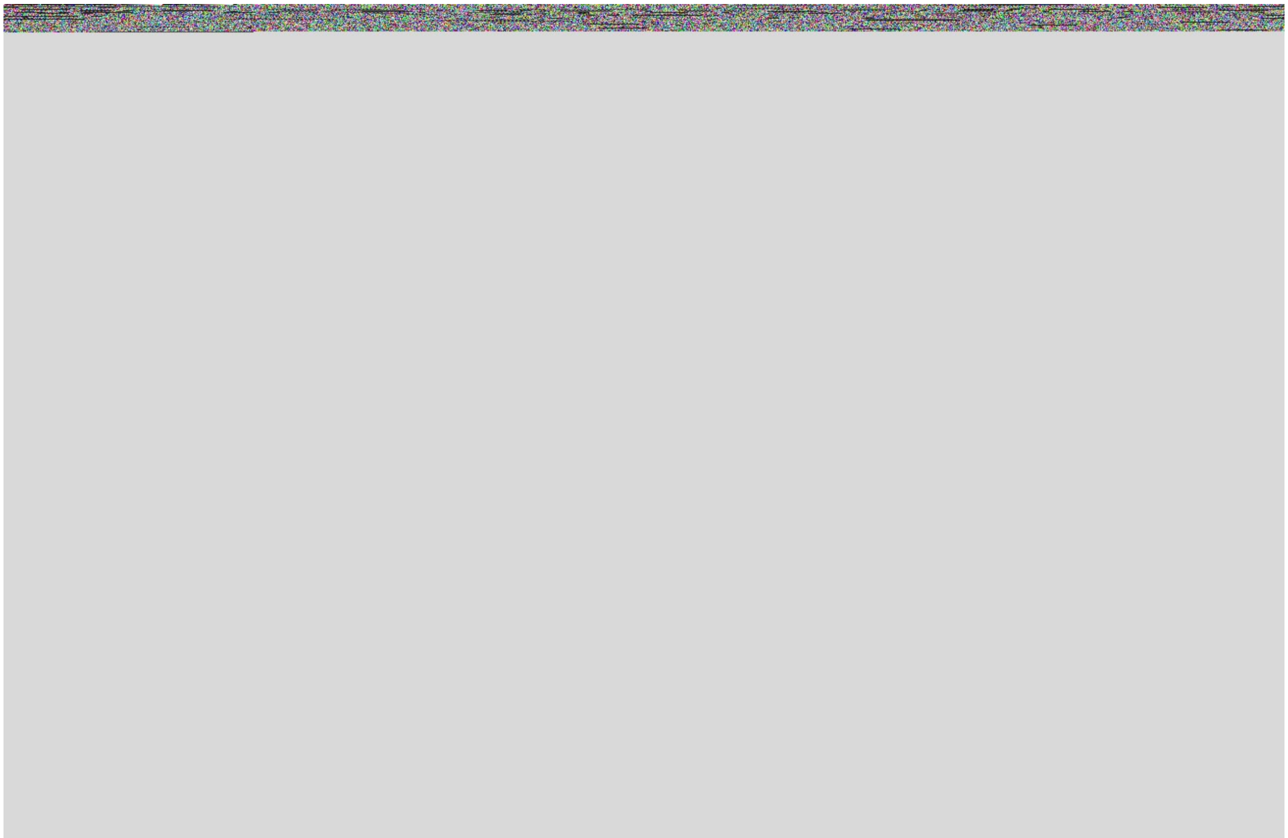

(*S*)-Methyl(phenyl)((4-(trifluoromethyl)phenyl)imino)-λ^6-sulfanone (**22**)

<Chromatogram>

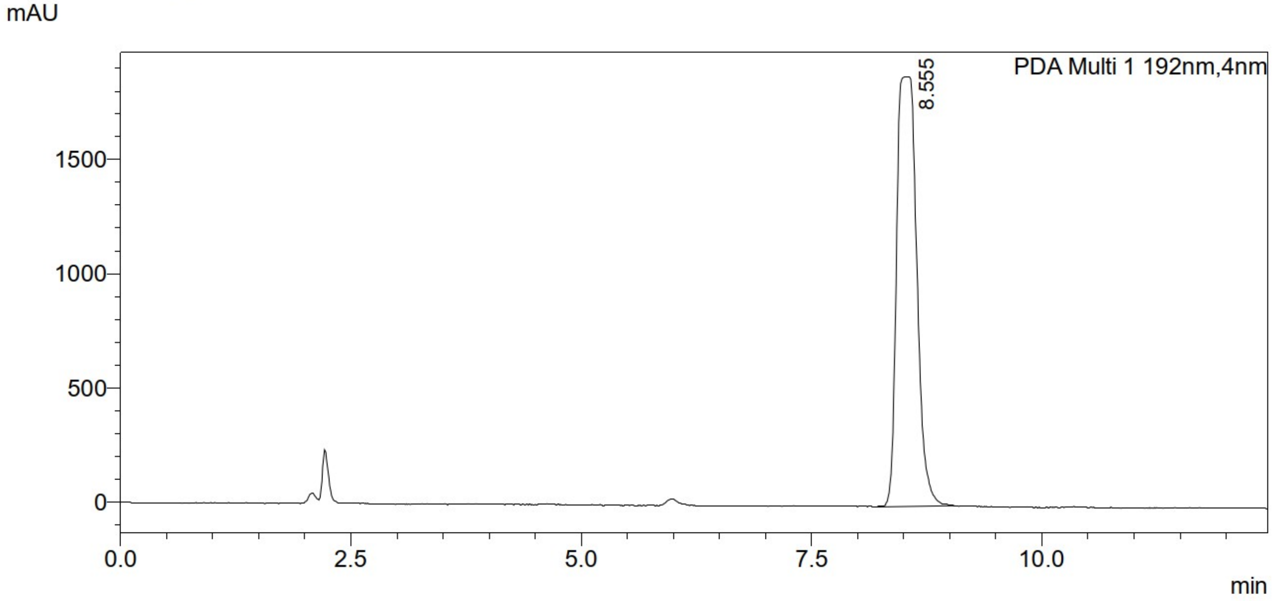

<Peak Table>

PDA Ch1 192nm

Peak#	Ret. Time	Area	Height	Area%
1	8.555	28445706	1880775	100.000
Total		28445706	1880775	100.000

NMR Spectroscopy:

^{1}H NMR (500 MHz, $CDCl_3$, 298 K, δ): 7.98–7.92 (m, 2H), 7.63–7.58 (m, 1H), 7.56–7.51 (m, 2H), 7.37–7.31 (m, 2H), 7.07–7.02 (m, 2H), 3.26 (s, 3H) ppm.

^{13}C NMR (126 MHz, $CDCl_3$, 298 K, δ): 148.8, 138.9, 133.7, 129.9, 128.6, 126.3 (q, *J* = 3.8 Hz), 124.7 (q, *J* = 269.5 Hz), 123.3 (q, *J* = 32.3 Hz), 122.9, 46.4 ppm.

^{19}F NMR (471 MHz, $CDCl_3$, 298 K, δ): –61.7 (s) ppm.

HRMS-ESI (m/z) calculated for $C_{14}H_{12}NOSF_3Na^+$ $[M+Na]^+$, 322.0484; found, 322.0483; deviation: 0.28 ppm.

Ethyl(phenyl)((4-(trifluoromethyl)phenyl)imino)-λ^6-sulfanone (23)

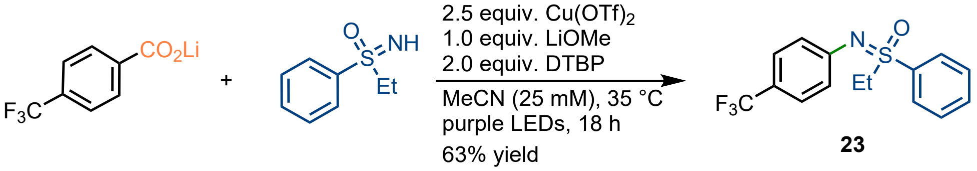

In a nitrogen-filled glovebox, a 16 mL borosilicate vial equipped with a magnetic stir bar was charged with lithium 4-(trifluoromethyl)benzoate (39.2 mg, 0.200 mmol, 1.00 equiv.), $Cu(OTf)_2$ (181 mg, 0.500 mmol, 2.50 equiv.), LiOMe (7.6 mg, 0.200 mmol, 1.00 equiv.), 2,6-di-*tert*-butylpyridine (76.5 mg, 0.400 mmol, 2.00 equiv.) and ethyl(imino)(phenyl)-λ^6-sulfanone (84.6 mg, 0.500 mmol, 2.50 equiv.). Anhydrous MeCN (8.0 mL, c = 25 mM) was then added into the vial. The vial was sealed with a Teflon cap and placed 5 cm away from two purple

LEDs (Kessil PR160L-390 nm LEDs). The reaction mixture was irradiated for 18 h while maintaining the temperature at approximately 35 °C through cooling with a fan. After irradiation, the reaction mixture was evaporated under reduced pressure to remove all volatiles. DCM (15 mL) was added to the residue, and the precipitation was removed by filtered through a Büchner funnel. The filtrate was evaporated under reduced pressure, and the residue was purified by chromatography on silica gel (EA/DCM = 1/50, v/v) to yield ethyl(phenyl)((4-(trifluoromethyl)phenyl)imino)-λ^6-sulfanone (**23**) (39.4 mg, 126 μmol, 63%) as a colorless oil.

R*f* = 0.42 (EA/DCM = 1/50, v/v).

NMR Spectroscopy:

^{1}H NMR (500 MHz, $CDCl_3$, 298 K, δ): 7.92–7.86 (m, 2H), 7.61 (tt, *J* = 6.8, 1.4 Hz, 1H), 7.56–7.50 (m, 2H), 7.34 (d, *J* = 8.6 Hz, 2H), 7.06 (d, *J* = 8.5 Hz, 2H), 3.43–3.29 (m, 2H), 1.32 (td, *J* = 7.4, 2.0 Hz, 3H) ppm.

^{13}C NMR (126 MHz, $CDCl_3$, 298 K, δ): 149.0, 136.9, 133.7, 129.8, 129.4, 126.2 (q, *J* = 3.9 Hz), 124.8 (q, *J* = 270.8 Hz), 123.1 (q, *J* = 32.2 Hz), 122.9, 52.4, 7.5 ppm.

^{19}F NMR (471 MHz, $CDCl_3$, 298 K, δ): –61.6 (s) ppm.

HRMS-EI (m/z) calculated for $C_{15}H_{14}NOSF_3^+$ $[M]^+$, 313.0743; found, 313.0749; deviation: –1.94 ppm.

(4-Chlorophenyl)(methyl)((4-(trifluoromethyl)phenyl)imino)-λ^6-sulfanone (24)

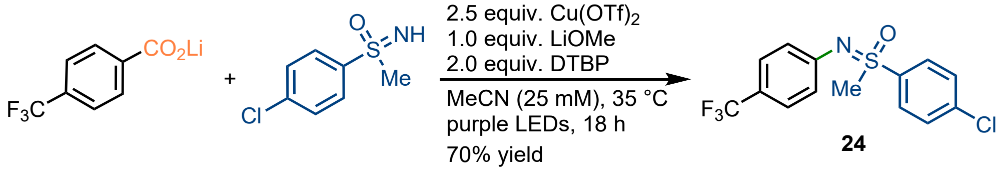

In a nitrogen-filled glovebox, a 16 mL borosilicate vial equipped with a magnetic stir bar was charged with lithium 4-(trifluoromethyl)benzoate (39.2 mg, 0.200 mmol, 1.00 equiv.), $Cu(OTf)_2$ (181 mg, 0.500 mmol, 2.50 equiv.), LiOMe (7.6 mg, 0.200 mmol, 1.00 equiv.), 2,6-di-*tert*-butylpyridine (76.5 mg, 0.400 mmol, 2.00 equiv.) and (4-chlorophenyl)(imino)(methyl)-λ^6-sulfanone (94.8 mg, 0.500 mmol, 2.50 equiv.). Anhydrous MeCN (8.0 mL, c = 25 mM) was then added into the vial. The vial was sealed with a Teflon cap and placed 5 cm away from two purple LEDs (Kessil PR160L-390 nm LEDs). The reaction mixture was irradiated for 18 h while maintaining the temperature at approximately 35 °C through cooling with a fan. After irradiation, the reaction mixture was evaporated under reduced pressure to remove all volatiles. DCM (15 mL) was added to the residue, and the precipitation was removed by filtered through a Büchner funnel. The filtrate was evaporated under reduced pressure, and the residue was purified by chromatography on silica gel (EA/DCM = 1/50, v/v) to yield (4-chlorophenyl)(methyl)((4-(trifluoromethyl)phenyl)imino)-λ^6-sulfanone (**24**) (46.7 mg, 140 μmol, 70%) as a colorless oil.

R*f* = 0.35 (DCM).

NMR Spectroscopy:

^{1}H NMR (500 MHz, $CDCl_3$, 298 K, δ): 7.93–7.83 (m, 2H), 7.56–7.47 (m, 2H), 7.36 (d, *J* = 8.3 Hz, 2H), 7.03

(d, J = 8.3 Hz, 2H), 3.27 (s, 3H) ppm.

^{13}C NMR (126 MHz, $CDCl_3$, 298 K, δ): 148.3, 140.6, 137.4, 130.2, 130.1, 126.4 (q, J = 3.7 Hz), 123.8 (q, J = 32.4 Hz), 124.6 (q, J = 269.5 Hz), 123.0, 46.5 ppm.

^{19}F NMR (471 MHz, $CDCl_3$, 298 K, δ): –61.7 (s) ppm.

HRMS-EI (m/z) calculated for $C_{14}H_{11}NOSF_3Cl^+$ $[M]^+$, 333.0197; found, 333.0201; deviation: –1.20 ppm.

(3,5-Dichlorophenyl)(methyl)((4-(trifluoromethyl)phenyl)imino)-λ^6-sulfanone (25)

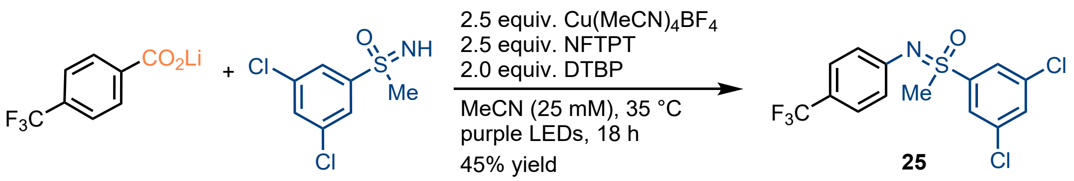

In a nitrogen-filled glovebox, a 16 mL borosilicate vial equipped with a magnetic stir bar was charged with lithium 4-(trifluoromethyl)benzoate (39.2 mg, 0.200 mmol, 1.00 equiv.), $Cu(MeCN)_4BF_4$ (157 mg, 0.500 mmol, 2.50 equiv.), 1-fluoro-2,4,6-trimethylpyridinium triflate (145 mg, 0.500 mmol, 2.50 equiv.), 2,6-di-*tert*-butylpyridine (76.5 mg, 0.400 mmol, 2.00 equiv.) and (3,5-dichlorophenyl)(imino)(methyl)-λ^6-sulfanone (112 mg, 0.500 mmol, 2.50 equiv.). Anhydrous MeCN (8.0 mL, c = 25 mM) was then added into the vial. The vial was sealed with a Teflon cap and placed 5 cm away from two purple LEDs (Kessil PR160L-390 nm LEDs). The reaction mixture was irradiated for 18 h while maintaining the temperature at approximately 35 °C through cooling with a fan. After irradiation, the reaction mixture was evaporated under reduced pressure to remove all volatiles. The residue was purified by chromatography on silica gel (DCM) to yield (3,5-dichlorophenyl)(methyl)((4-(trifluoromethyl)phenyl)imino)-λ^6-sulfanone (**25**) (33.1 mg, 90 μmol, 45%) as a colorless oil.

R*f* = 0.52 (DCM).

NMR Spectroscopy:

^{1}H NMR (500 MHz, $CDCl_3$, 298 K, δ): 7.76 (d, J = 1.9 Hz, 2H), 7.52 (t, J = 1.9 Hz, 1H), 7.35–7.31 (m, 2H), 7.01–6.97 (m, 2H), 3.21 (s, 3H) ppm.

^{13}C NMR (126 MHz, $CDCl_3$, 298 K, δ): 147.7, 142.4, 136.9, 133.9, 127.0, 126.5 (q, J = 3.7 Hz), 124.6 (q, J = 269.5 Hz),124.2 (q, J = 32.7 Hz), 123.1, 46.3 ppm.

^{19}F NMR (471 MHz, $CDCl_3$, 298 K, δ): –61.8 (s) ppm.

HRMS-ESI (m/z) calculated for $C_{14}H_{11}NOSCl_2F_3^+$ $[M+H]^+$, 367.9885; found, 367.9888; deviation: –0.92 ppm.

(3-Methylphenyl)(methyl)((4-(trifluoromethyl)phenyl)imino)-λ^6-sulfanone (26)

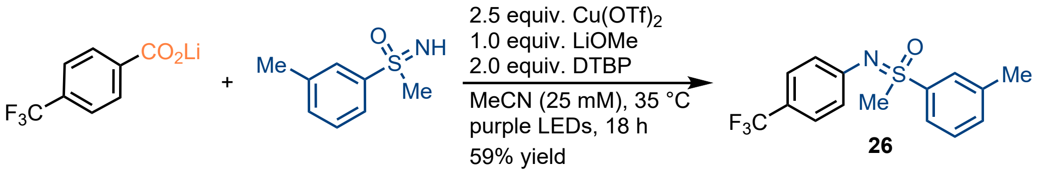

In a nitrogen-filled glovebox, a 16 mL borosilicate vial equipped with a magnetic stir bar was charged with lithium 4-(trifluoromethyl)benzoate (39.2 mg, 0.200 mmol, 1.00 equiv.), $Cu(OTf)_2$ (181 mg, 0.500 mmol, 2.50 equiv.), LiOMe (7.6 mg, 0.200 mmol, 1.00 equiv.), 2,6-di-*tert*-butylpyridine (76.5 mg, 0.400 mmol, 2.00 equiv.) and (3-methylphenyl)(imino)(methyl)-λ^6-sulfanone (84.6 mg, 0.500 mmol, 2.50 equiv.). Anhydrous MeCN (8.0 mL, c = 25 mM) was then added into the vial. The vial was sealed with a Teflon cap and placed 5 cm away from two purple LEDs (Kessil PR160L-390 nm LEDs). The reaction mixture was irradiated for 18 h while maintaining the temperature at approximately 35 °C through cooling with a fan. After irradiation, the reaction mixture was evaporated under reduced pressure to remove all volatiles. DCM (15 mL) was added to the residue, and the precipitation was removed by filtered through a Büchner funnel. The filtrate was evaporated under reduced pressure, and the residue was purified by chromatography on silica gel (EA/DCM = 1/50, v/v) to yield (3-methylphenyl)(methyl)((4-(trifluoromethyl)phenyl)imino)-λ^6-sulfanone (**26**) (36.9 mg, 118 µmol, 59%) as a colorless oil.

R*f* = 0.50 (EA/DCM = 1/25, v/v).

NMR Spectroscopy:

^{1}H NMR (500 MHz, $CDCl_3$, 298 K, δ): 7.79–7.76 (m, 1H), 7.73 (ddd, *J* = 5.4, 3.6, 2.0 Hz, 1H), 7.43–7.39 (m, 2H), 7.35 (d, *J* = 8.4 Hz, 2H), 7.05 (d, *J* = 8.3 Hz, 2H), 3.25 (s, 3H), 2.42 (s, 3H) ppm.

^{13}C NMR (126 MHz, $CDCl_3$, 298 K, δ): 148.9, 140.3, 138.8, 134.6, 129.7, 128.9, 126.3 (q, *J* = 3.8 Hz), 125.6, 124.7 (q, *J* = 269.3 Hz), 123.3 (q, *J* = 32.4 Hz), 122.9, 46.5, 21.5 ppm.

^{19}F NMR (471 MHz, $CDCl_3$, 298 K, δ): –61.7 (s) ppm.

HRMS-EI (m/z) calculated for $C_{15}H_{14}NOSF_3^+$ $[M]^+$, 313.0743; found, 313.0749; deviation: –1.94 ppm.

(4-Cyanophenyl)(methyl)((4-(trifluoromethyl)phenyl)imino)-λ^6-sulfanone (27)

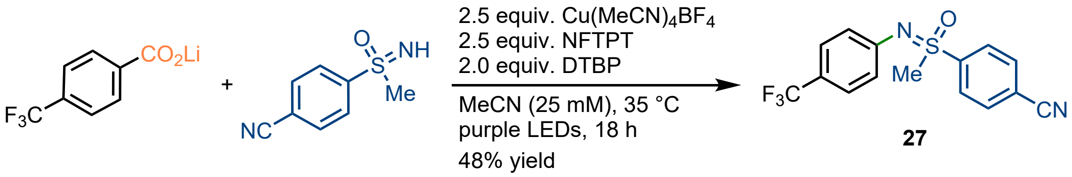

In a nitrogen-filled glovebox, a 16 mL borosilicate vial equipped with a magnetic stir bar was charged with lithium 4-(trifluoromethyl)benzoate (39.2 mg, 0.200 mmol, 1.00 equiv.), $Cu(MeCN)_4BF_4$ (157 mg, 0.500 mmol, 2.50 equiv.), 1-fluoro-2,4,6-trimethylpyridinium triflate (145 mg, 0.500 mmol, 2.50 equiv.), 2,6-di-*tert*-butylpyridine (76.5 mg, 0.400 mmol, 2.00 equiv.) and (4-cyanophenyl)(imino)(methyl)-λ^6-sulfanone (90.1 mg,

0.500 mmol, 2.50 equiv.). Anhydrous MeCN (8.0 mL, c = 25 mM) was then added into the vial. The vial was sealed with a Teflon cap and placed 5 cm away from two purple LEDs (Kessil PR160L-390 nm LEDs). The reaction mixture was irradiated for 18 h while maintaining the temperature at approximately 35 °C through cooling with a fan. After irradiation, the reaction mixture was evaporated under reduced pressure to remove all volatiles. The residue was purified by chromatography on silica gel (DCM) to yield (4-cyanophenyl)(methyl)((4-(trifluoromethyl)phenyl)imino)-λ^6-sulfanone (**27**) (31.2 mg, 96 µmol, 48%) as a colorless solid.

R*f* = 0.33 (DCM).

NMR Spectroscopy:

^{1}**H NMR** (500 MHz, $CDCl_3$, 298 K, δ): 8.11–8.05 (m, 2H), 7.88–7.82 (m, 2H), 7.39–7.34 (m, 2H), 7.07–7.00 (m, 2H), 3.30 (s, 3H) ppm.

^{13}**C NMR** (126 MHz, $CDCl_3$, 298 K, δ): 147.8, 143.6, 133.6, 129.4, 126.5 (q, *J* = 3.8 Hz), 124.5 (q, *J* = 269.5 Hz), 124.2 (q, *J* = 32.4 Hz),123.0, 117.6, 117.2, 46.1 ppm.

^{19}**F NMR** (471 MHz, $CDCl_3$, 298 K, δ): –61.8 (s) ppm.

HRMS-ESI (m/z) calculated for $C_{15}H_{12}N_2OSF_3^+$ $[M+H]^+$, 325.0617; found, 325.0618; deviation: –0.44 ppm.

(2-Bromophenyl)(methyl)((4-(trifluoromethyl)phenyl)imino)-λ^6-sulfanone (28)

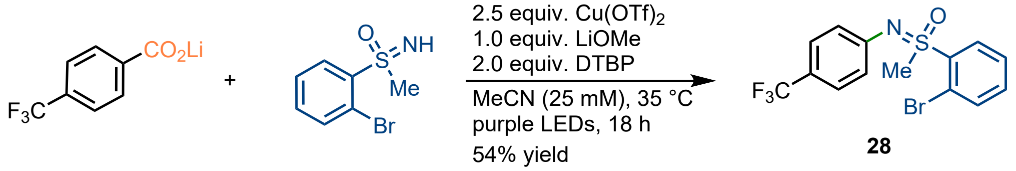

28

In a nitrogen-filled glovebox, a 16 mL borosilicate vial equipped with a magnetic stir bar was charged with lithium 4-(trifluoromethyl)benzoate (39.2 mg, 0.200 mmol, 1.00 equiv.), $Cu(OTf)_2$ (181 mg, 0.500 mmol, 2.50 equiv.), LiOMe (7.6 mg, 0.200 mmol, 1.00 equiv.), 2,6-di-*tert*-butylpyridine (76.5 mg, 0.400 mmol, 2.00 equiv.) and (2-bromophenyl)(imino)(methyl)-λ^6-sulfanone (117 mg, 0.500 mmol, 2.50 equiv.). Anhydrous MeCN (8.0 mL, c = 25 mM) was then added into the vial. The vial was sealed with a Teflon cap and placed 5 cm away from two purple LEDs (Kessil PR160L-390 nm LEDs). The reaction mixture was irradiated for 18 h while maintaining the temperature at approximately 35 °C through cooling with a fan. After irradiation, the reaction mixture was evaporated under reduced pressure to remove all volatiles. DCM (15 mL) was added to the residue, and the precipitation was removed by filtered through a Büchner funnel. The filtrate was evaporated under reduced pressure, and the residue was purified by chromatography on silica gel (EA/DCM = 1/50, v/v) to yield (2-bromophenyl)(methyl)((4-(trifluoromethyl)phenyl)imino)-λ^6-sulfanone (**28**) (41.1 mg, 109 µmol, 54%) as a yellow oil.

R*f* = 0.56 (EA/DCM = 1/25, v/v).

NMR Spectroscopy:

^{1}**H NMR** (500 MHz, $CDCl_3$, 298 K, δ): 8.29 (dd, *J* = 7.9, 1.7 Hz, 1H), 7.69 (dd, *J* = 7.9, 1.3 Hz, 1H), 7.51

(td, J = 7.6, 1.3 Hz, 1H), 7.41 (td, J = 7.6, 1.7 Hz, 1H), 7.36–7.30 (m, 2H), 7.08–7.00 (m, 2H), 3.49 (s, 3H) ppm.

^{13}C NMR (126 MHz, $CDCl_3$, 298 K, δ): 148.2, 137.9, 136.1, 134.7, 133.4, 128.3, 126.2 (q, J = 3.7 Hz), 124.7 (q, J = 269.4 Hz), 123.8 (d, J = 32.3 Hz), 122.9, 120.5, 43.6. ppm.

^{19}F NMR (471 MHz, $CDCl_3$, 298 K, δ): –61.7 (s) ppm.

HRMS-EI (m/z) calculated for $C_{15}H_{14}NOSF_3^+$ [M]$^+$, 313.0743; found, 313.0749; deviation: –1.94 ppm.

(4-Methoxyphenyl)(methyl)((4-(trifluoromethyl)phenyl)imino)-λ^6-sulfanone (29)

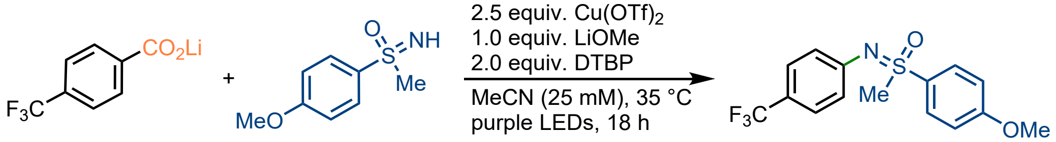

29

In a nitrogen-filled glovebox, a 16 mL borosilicate vial equipped with a magnetic stir bar was charged with lithium 4-(trifluoromethyl)benzoate (39.2 mg, 0.200 mmol, 1.00 equiv.), $Cu(OTf)_2$ (181 mg, 0.500 mmol, 2.50 equiv.), LiOMe (7.6 mg, 0.200 mmol, 1.00 equiv.), 2,6-di-*tert*-butylpyridine (76.5 mg, 0.400 mmol, 2.00 equiv.) and (4-methoxyphenyl)(imino)(methyl)-λ^6-sulfanone (92.6 mg, 0.500 mmol, 2.50 equiv.). Anhydrous MeCN (8.0 mL, c = 25 mM) was then added into the vial. The vial was sealed with a Teflon cap and placed 5 cm away from two purple LEDs (Kessil PR160L-390 nm LEDs). The reaction mixture was irradiated for 18 h while maintaining the temperature at approximately 35 °C through cooling with a fan. After irradiation, the reaction mixture was evaporated under reduced pressure to remove all volatiles. DCM (15 mL) was added to the residue, and the precipitation was removed by filtered through a Büchner funnel. The filtrate was evaporated under reduced pressure, and the residue was purified by chromatography on silica gel (EA/DCM = 1/25, v/v) to yield (4-methoxyphenyl)(methyl)((4-(trifluoromethyl)phenyl)imino)-λ^6-sulfanone (**29**) (39.6 mg, 120 µmol, 60%) as a yellow oil.

R*f* = 0.26 (EA/DCM = 1/25, v/v).

NMR Spectroscopy:

^{1}H NMR (500 MHz, $CDCl_3$, 298 K, δ): 7.89–7.82 (m, 2H), 7.35 (d, J = 8.5 Hz, 2H), 7.03 (d, J = 8.2 Hz, 2H), 7.01–6.96 (m, 2H), 3.85 (s, 3H), 3.25 (s, 3H) ppm.

^{13}C NMR (126 MHz, $CDCl_3$, 298 K, δ): 163.8, 149.0, 130.8, 129.9, 126.2 (q, J = 3.8 Hz), 124.7 (q, J = 271.1 Hz), 123.3 (q, J = 32.1 Hz), 122.9, 115.1, 55.8, 46.9 ppm.

^{19}F NMR (471 MHz, $CDCl_3$, 298 K, δ): –61.6 (s) ppm.

HRMS-EI (m/z) calculated for $C_{15}H_{14}NO_2SF_3^+$ [M]$^+$, 329.0692; found, 329.0697; deviation: –1.65 ppm.

(4-Trifluoromethoxyphenyl)(methyl)((4-(trifluoromethyl)phenyl)imino)-λ⁶-sulfanone (30)

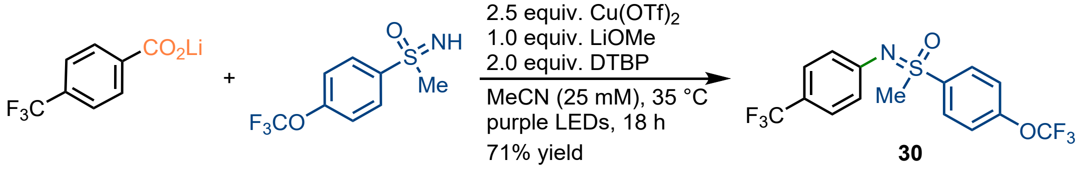

30

In a nitrogen-filled glovebox, a 16 mL borosilicate vial equipped with a magnetic stir bar was charged with lithium 4-(trifluoromethyl)benzoate (39.2 mg, 0.200 mmol, 1.00 equiv.), $Cu(OTf)_2$ (181 mg, 0.500 mmol, 2.50 equiv.), LiOMe (7.6 mg, 0.200 mmol, 1.00 equiv.), 2,6-di-*tert*-butylpyridine (76.5 mg, 0.400 mmol, 2.00 equiv.) and imino(methyl)(4-(trifluoromethoxy)phenyl)-λ^6-sulfanone (120 mg, 0.500 mmol, 2.50 equiv.). Anhydrous MeCN (8.0 mL, c = 25 mM) was then added into the vial. The vial was sealed with a Teflon cap and placed 5 cm away from two purple LEDs (Kessil PR160L-390 nm LEDs). The reaction mixture was irradiated for 18 h while maintaining the temperature at approximately 35 °C through cooling with a fan. After irradiation, the reaction mixture was evaporated under reduced pressure to remove all volatiles. DCM (15 mL) was added to the residue, and the precipitation was removed by filtered through a Büchner funnel. The filtrate was evaporated under reduced pressure, and the residue was purified by chromatography on silica gel (EA/DCM = 1/50, v/v) to yield methyl(4-(trifluoromethoxy)phenyl)((4-(trifluoromethyl)phenyl)imino)-λ^6-sulfanone (**30**) (54.1 mg, 141 μmol, 71%) as a yellow oil.

R*f* = 0.56 (EA/DCM = 1/25, v/v).

NMR Spectroscopy:

^{1}H NMR (500 MHz, $CDCl_3$, 298 K, δ): 8.03–7.98 (m, 2H), 7.39–7.34 (m, 4H), 7.08–7.03 (m, 2H), 3.28 (s, 3H) ppm.

^{13}C NMR (126 MHz, $CDCl_3$, 298 K, δ): 153.1, 148.3, 137.2, 130.9, 126.4 (q, J = 3.8 Hz), 124.6 (q, J = 269.6 Hz), 123.8 (q, J = 32.7 Hz), 123.0, 121.5, 120.3 (q, J = 259.9 Hz), 46.5 ppm.

^{19}F NMR (471 MHz, $CDCl_3$, 298 K, δ): –57.7 (s), –61.8 (s) ppm.

HRMS-EI (m/z) calculated for $C_{15}H_{11}NO_2SF_6^+$ $[M]^+$, 383.0409; found, 383.0417; deviation: –1.95 ppm.

Dimethyl((4-(trifluoromethyl)phenyl)imino)-λ⁶-sulfanone (31)

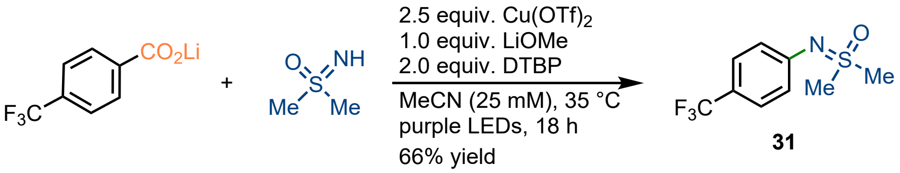

31

In a nitrogen-filled glovebox, a 16 mL borosilicate vial equipped with a magnetic stir bar was charged with lithium 4-(trifluoromethyl)benzoate (39.2 mg, 0.200 mmol, 1.00 equiv.), $Cu(OTf)_2$ (181 mg, 0.500 mmol, 2.50 equiv.), LiOMe (7.6 mg, 0.200 mmol, 1.00 equiv.), 2,6-di-*tert*-butylpyridine (76.5 mg, 0.400 mmol, 2.00 equiv.) and iminodimethyl-λ^6-sulfanone (46.6 mg, 0.500 mmol, 2.50 equiv.). Anhydrous MeCN (8.0 mL, c = 25 mM) was then added into the vial. The vial was sealed with a Teflon cap and placed 5 cm away from two purple

LEDs (Kessil PR160L-390 nm LEDs). The reaction mixture was irradiated for 18 h while maintaining the temperature at approximately 35 °C through cooling with a fan. After irradiation, the reaction mixture was evaporated under reduced pressure to remove all volatiles. DCM (15 mL) was added to the residue, and the precipitation was removed by filtered through a Büchner funnel. The filtrate was evaporated under reduced pressure, and the residue was purified by chromatography on silica gel (EA/DCM = 1/50, v/v) to yield dimethyl((4-(trifluoromethyl)phenyl)imino)-λ^6-sulfanone (**31**) (31.4 mg, 132 μmol, 66%) as a colorless solid.

R*f* = 0.25 (EA/DCM = 1/50, v/v).

NMR Spectroscopy:

^{1}H NMR (500 MHz, $CDCl_3$, 298 K, δ): 7.46 (d, *J* = 8.3 Hz, 2H), 7.13 (d, *J* = 8.3 Hz, 2H), 3.17 (s, 6H) ppm.

^{13}C NMR (126 MHz, $CDCl_3$, 298 K, δ): 149.0, 126.5 (q, *J* = 3.9 Hz), 124.7 (q, *J* = 269.4 Hz), 123.9 (d, *J* = 32.2 Hz), 122.9, 42.4 ppm.

^{19}F NMR (471 MHz, $CDCl_3$, 298 K, δ): –61.7 (s) ppm.

HRMS-EI (m/z) calculated for $C_9H_{10}NOSF_3^+$ $[M]^+$, 237.0430; found, 237.0431; deviation: –0.37 ppm.

Diphenyl((4-(trifluoromethyl)phenyl)imino)-λ^6-sulfanone (32)

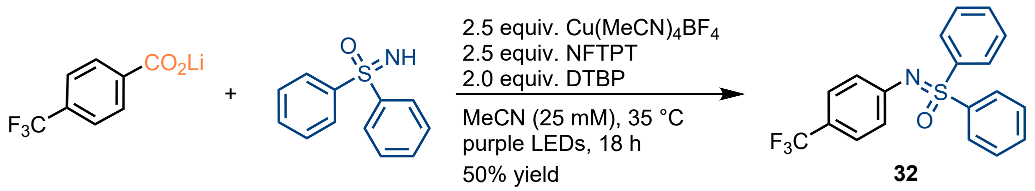

In a nitrogen-filled glovebox, a 16 mL borosilicate vial equipped with a magnetic stir bar was charged with lithium 4-(trifluoromethyl)benzoate (39.2 mg, 0.200 mmol, 1.00 equiv.), $Cu(MeCN)_4BF_4$ (157 mg, 0.500 mmol, 2.50 equiv.), 1-fluoro-2,4,6-trimethylpyridinium triflate (145 mg, 0.500 mmol, 2.50 equiv.), 2,6-di-*tert*-butylpyridine (76.5 mg, 0.400 mmol, 2.00 equiv.) and iminodiphenyl-λ^6-sulfanone (109 mg, 0.500 mmol, 2.50 equiv.). Anhydrous MeCN (8.0 mL, c = 25 mM) was then added into the vial. The vial was sealed with a Teflon cap and placed 5 cm away from two purple LEDs (Kessil PR160L-390 nm LEDs). The reaction mixture was irradiated for 18 h while maintaining the temperature at approximately 35 °C through cooling with a fan. After irradiation, the reaction mixture was evaporated under reduced pressure to remove all volatiles. The residue was purified by chromatography on silica gel (DCM) to yield diphenyl((4-(trifluoromethyl)phenyl)imino)-λ^6-sulfanone (**32**) (36.3 mg, 100 μmol, 50%) as a colorless solid.

R*f* = 0.63 (DCM).

NMR Spectroscopy:

^{1}H NMR (500 MHz, $CDCl_3$, 298 K, δ): 8.10–8.00 (m, 4H), 7.57–7.46 (m, 6H), 7.41–7.36 (m, 2H), 7.20 (d, *J* = 8.4 Hz, 2H) ppm.

^{13}C NMR (126 MHz, $CDCl_3$, 298 K, δ): 148.5, 140.5, 133.1, 129.6, 128.5, 126.3 (q, *J* = 3.8 Hz), 124.8 (q,

J = 269.4 Hz), 123.5, 123.2 (q, *J* = 32.3 Hz) ppm.

^{19}F NMR (471 MHz, $CDCl_3$, 298 K, δ): –61.6 (s) ppm.

HRMS-ESI (m/z) calculated for $C_{19}H_{14}F_3NOSNa^+$ $[M+Na]^+$, 384.0640; found, 384.0642; deviation: –0.28 ppm.

1 mmol scale decarboxylative sulfoximination of lithium 4-(trifluoromethyl)benzoate

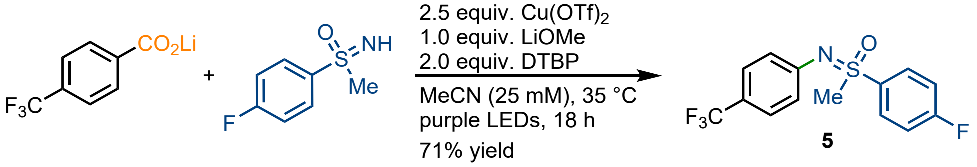

In a nitrogen-filled glovebox, a 100 mL borosilicate vial equipped with a magnetic stir bar was charged with lithium 4-(trifluoromethyl)benzoate (196 mg, 1.00 mmol, 1.00 equiv.), $Cu(OTf)_2$ (904 mg, 2.50 mmol, 2.50 equiv.), LiOMe (38.0 mg, 1.00 mmol, 1.00 equiv.), 2,6-di-*tert*-butylpyridine (383 mg, 2.00 mmol, 2.00 equiv.) and (4-fluorophenyl)(imino)(methyl)-λ^6-sulfanone (433 mg, 2.50 mmol, 2.50 equiv.). Anhydrous MeCN (40.0 mL, c = 25 mM) was then added into the vial. The vial was sealed with a Teflon cap and placed 5 cm away from two purple LEDs (Kessil PR160L-390 nm LEDs). The reaction mixture was irradiated for 18 h while maintaining the temperature at approximately 35 °C through cooling with a fan. After irradiation, the reaction mixture was evaporated under reduced pressure to remove all volatiles. DCM (30 mL) was added to the residue, and the precipitation was removed by filtered through a Büchner funnel. The filtrate was evaporated under reduced pressure, and the residue was purified by chromatography on silica gel (EA/DCM = 1/50, v/v) to yield (4-fluorophenyl)(methyl)((4-(trifluoromethyl)phenyl)imino)-λ^6-sulfanone (**5**) (225 mg, 0.709 mmol, 71%) as a slightly yellow oil.

Tested *ortho*-substituted substrates

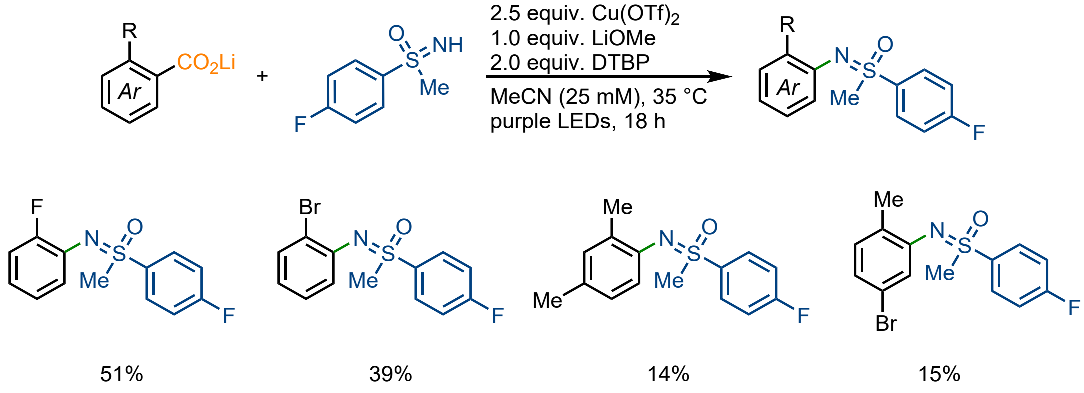

Reactions with catalytic amounts of $Cu(OTf)_2$ and oxidants

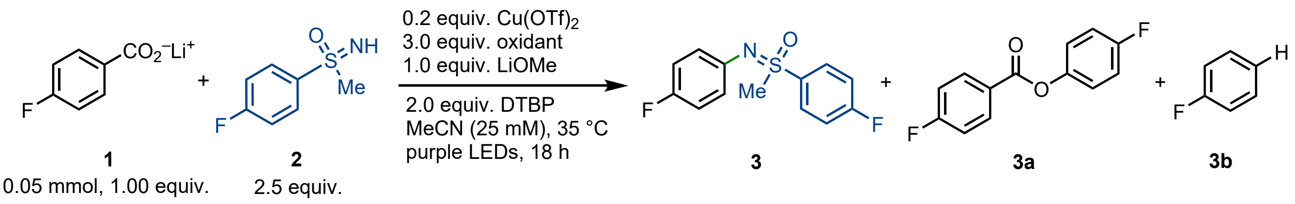

oxidant	Yield (**3**/**3a**/**3b**, %)[a]
$K_2S_2O_8$	0/0/10
N-Fluorobenzenesulfonimide	0/0/68
1-Fluoro-2,4,6-trimethylpyridinium tetrafluoroborate	0/0/0
Di-*tert*-butyl peroxide	0/0/0

[a] ^{19}F NMR yield with 2-fluorotoluene(2.0 equiv.) as an internal standard.

Mechanistic Studies

UV-vis Absorption Spectrum

All UV-vis measurements were recorded on a Shimadzu UV-vis Spectrophotometer UV-2600 with temperature controller using a screw-top quartz cuvette (Hellma fluorescence quartz cuvette, 10 × 10 mm, 3.5 mL). Samples were prepared in a glovebox and then taken out of the glovebox. UV-vis spectra of lithium 4-fluorobenzoate (**1**, 1 mM), (4-fluorophenyl)(imino)(methyl)-λ^6-sulfanone (**2**, 2.5 mM), (4-fluorophenyl)((4-fluorophenyl)imino)(methyl)-λ^6-sulfanone (**3**, 1 mM), $Cu(OTf)_2$ (2.5 mM), LiOMe (1.0 mM), 2,6-di-*tert*-butylpyridine (DTBP, 2.0 mM), the mixture of **1** (1 mM) + $Cu(OTf)_2$ (2.5 mM), the mixture of **2** (2.5 mM) + $Cu(OTf)_2$ (2.5 mM), the mixture of **3** (1 mM) + $Cu(OTf)_2$ (2.5 mM), the mixture of DTBP (2.0 mM) + $Cu(OTf)_2$ (2.5 mM), the mixture of **2** (2.5 mM) + DTBP (2 mM) + LiOMe (1mM) + $Cu(OTf)_2$ (2.5 mM), the mixture of **1** (1 mM) + **2** (2.5 mM) + DTBP (2 mM) + LiOMe (1mM) + $Cu(OTf)_2$ (2.5 mM) were recorded respectively, using MeCN as the solvent.

Note: Lithium 4-fluorobenzoate (**1**) and LiOMe are poorly dissolved in MeCN. All lithium 4-fluorobenzoate-containing or LiOMe-containing samples were filtered through a 0.22 μm syringe filter before measurement, so the actual concentration of lithium 4-fluorobenzoate (**1**) or LiOMe in these samples is less than 1.0 mM.

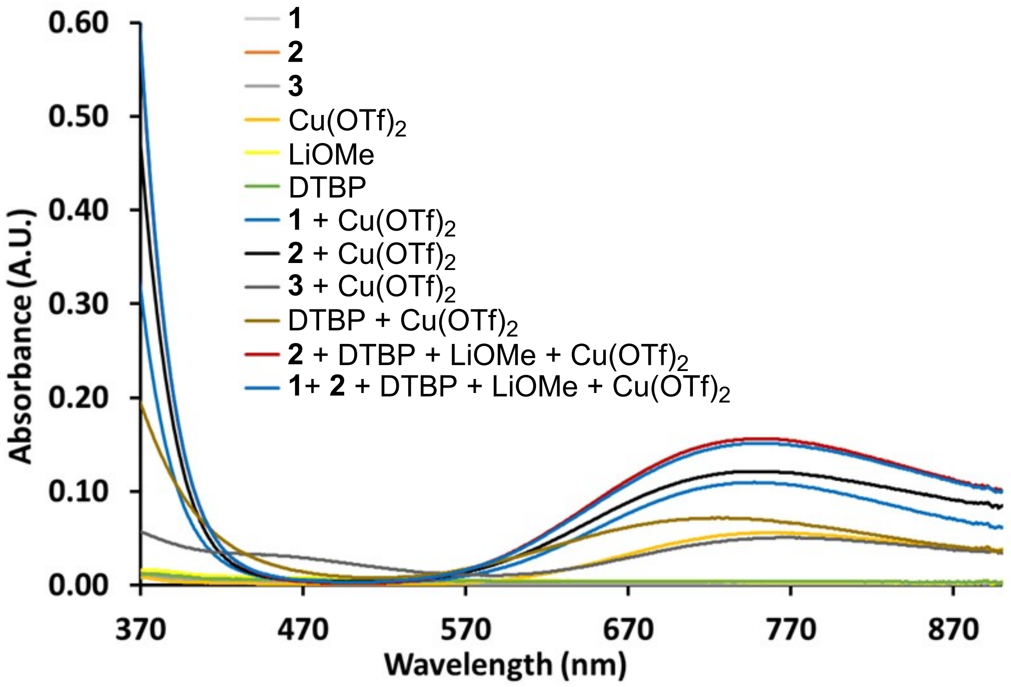

Figure S1. UV-vis spectra analysis of the reaction components.

Photolysis of the mixture of 1, 2, DTBP, LiOMe and $Cu(OTf)_2$

In a nitrogen-filled glovebox, a mixture of **1** (1 mM) + **2** (2.5 mM) + DTBP (2 mM) + LiOMe (1mM) + $Cu(OTf)_2$ (2.5 mM) in 3.0 mL MeCN was transferred to a screw-top quartz cuvette (Hellma fluorescence quartz cuvette, 10 × 10 mm, 3.5 mL). The quartz cuvette was sealed and taken out of the glovebox. The absorption spectra were recorded on a Shimadzu UV-vis Spectrophotometer UV-2600 after the cuvette was irradiated by two Kessil PR160L-390 nm LEDs (5 cm away from two Kessil PR160L-390 nm LEDs, and the temperature was maintained at approximately 35 °C through cooling with a fan) for various time (0.0 min, 1 min, 2 min, 4 min, 8 min, 16 min, 32 min and 64 min).

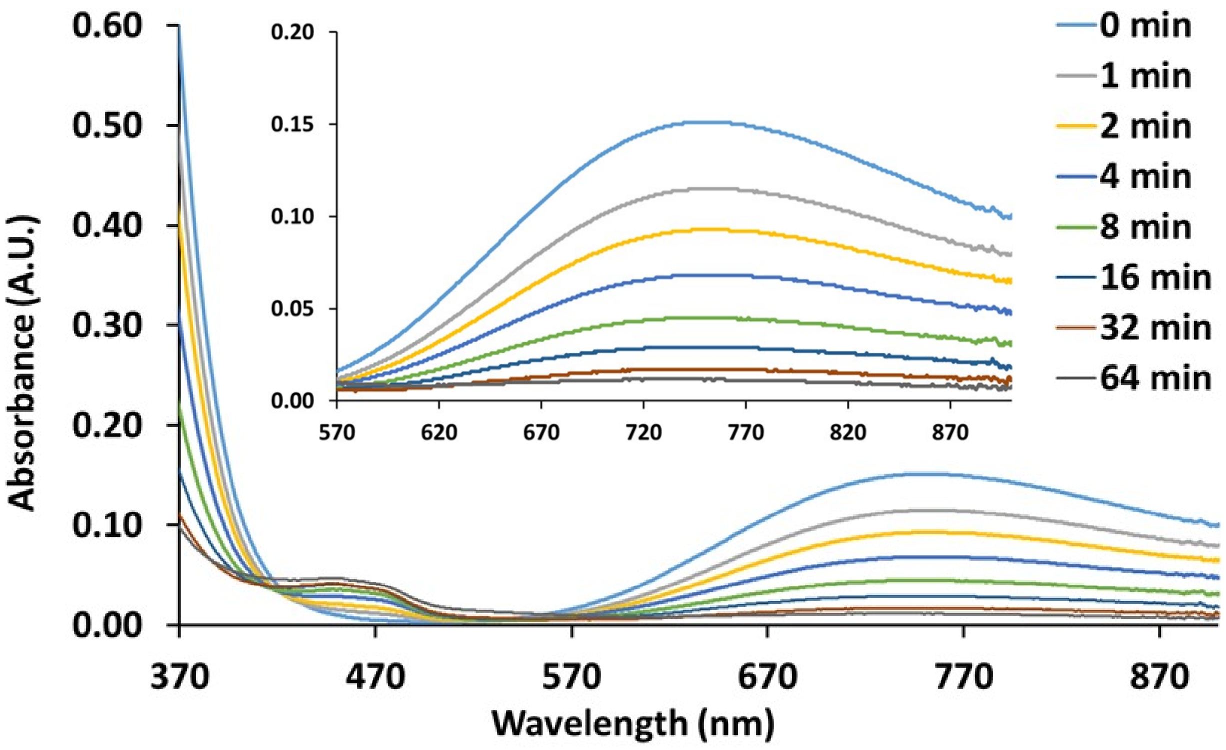

Figure S2. UV-vis spectra of the photolyzed mixture of **1**, **2**, DTBP, LiOMe and $Cu(OTf)_2$ in CH_3CN.

After photolysis, 0.5 mL reaction mixture was taken out and diluted with CD_3CN, and the formation of (4-fluorophenyl)((4-fluorophenyl)imino)(methyl)-λ^6-sulfanone (**3**) was confirmed by ^{19}F NMR. Upon addition of 2,2′-biquinoline (3.8 mg) to the rest of the reaction mixture, the colourless solution turned to dark purple colour, which originates from the formation of a purple $[Cu^I(biq)_2]^+$ complex. 0.25 mL of the above purple reaction mixture was taken out and further diluted to 3.0 mL with MeCN. The absorption spectrum was recorded on a UV-vis spectrophotometer, and a significant absorbance (λ_{max} = 546 nm) was observed.

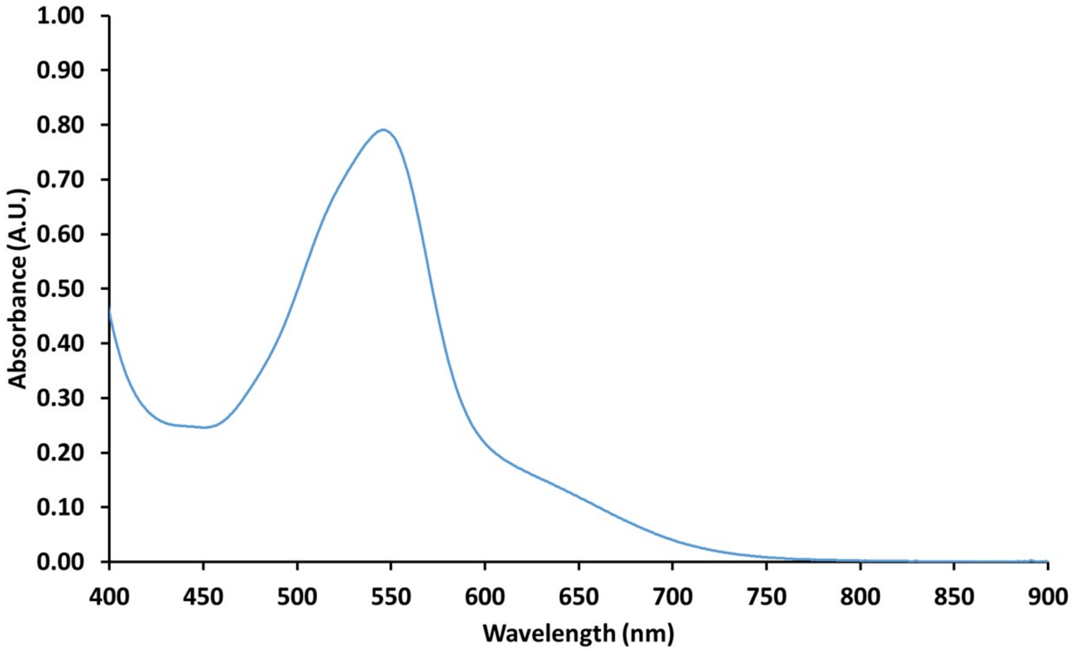

Figure S3. UV-vis spectra of $[Cu^{I}(biq)_2]^+$ complex.

Radical trapping experiments

Reaction with lithium 3-methoxybenzoate (33)

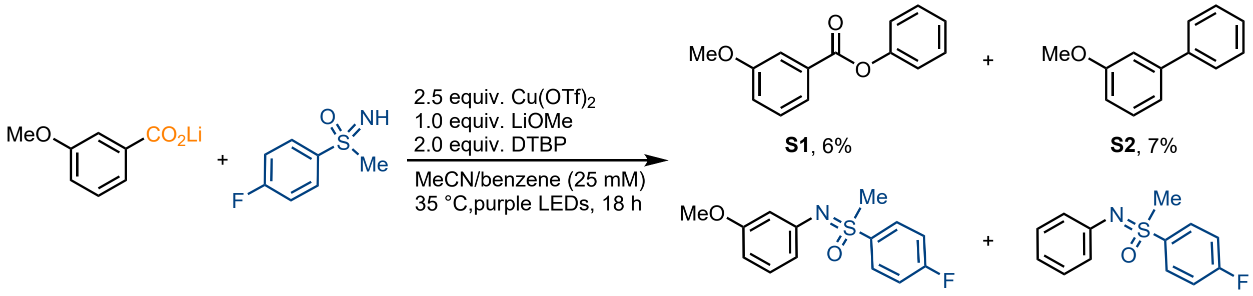

In a nitrogen-filled glovebox, a 16 mL borosilicate vial equipped with a magnetic stir bar was charged with lithium 3-methoxybenzoate (29.2 mg, 0.200 mmol, 1.00 equiv.), $Cu(OTf)_2$ (181 mg, 0.500 mmol, 2.50 equiv.), LiOMe (7.6 mg, 0.200 mmol, 1.00 equiv.), 2,6-di-*tert*-butylpyridine (76.5 mg, 0.400 mmol, 2.00 equiv.) and (4-fluorophenyl)(imino)(methyl)-λ^6-sulfanone (86.5 mg, 0.500 mmol, 2.50 equiv.). Anhydrous MeCN (4.0 mL) and benzene (4.0 mL) were then added into the vial. The vial was sealed with a Teflon cap, taken out of the glovebox and placed 5 cm away from two purple LEDs (Kessil PR160L-390 nm LEDs). The reaction mixture was irradiated for 18 h while maintaining the temperature at approximately 35 °C through cooling with a fan. After irradiation, the reaction mixture was evaporated under reduced pressure to remove all volatiles. The residue was purified by chromatography on silica gel (EA/hexanes = 1/50, v/v to EA/DCM = 1/50, v/v) to yield 3-methoxy-1,1′-biphenyl (**S2**) (2.4 mg, 13 μmol, 7%) as a colorless oil, phenyl 3-methoxybenzoate (**S1**) (2.8 mg, 12 μmol, 6%) as a colorless solid, ((3-methoxyphenyl)imino)(4-fluorophenyl)(methyl)-λ^6-sulfanone (**11**) (13.0

mg, 47 µmol, 23%) as a colorless oil and (4-fluorophenyl)(methyl)(phenylimino)-λ^6-sulfanone (**S3**) (5.7 mg, 23 µmol, 5%) as a colorless oil.

Phenyl 3-methoxybenzoate (**S1**):

R*f* =0.24 (EA/hexanes, 1/50, v/v).

NMR Spectroscopy:

^{1}**H NMR** (600 MHz, $CDCl_3$, 298 K, δ): 7.81 (dd, *J* = 7.6, 2.6 Hz, 1H), 7.72–7.70 (m, 1H), 7.45–7.41 (m, 3H), 7.30–7.26 (m, 1H), 7.23–7.20 (m, 2H), 7.20–7.17 (m, 1H), 3.89 (s, 3H) ppm.

^{13}**C NMR** (150 MHz, $CDCl_3$, 298 K, δ): 165.2, 160.0, 151.1, 131.0, 129.8, 129.7, 126.1, 122.8, 121.9, 120.3, 114.6, 55.7 ppm.

HRMS-ESI (m/z) calculated for $C_{14}H_{12}O_3Na^+$ $[M+Na]^+$, 251.0679; found, 251.0680; deviation: -0.62 ppm.

3-Methoxy-1,1′-biphenyl (**S2**):

R*f* =0.37 (EA/hexanes, 1/50, v/v).

NMR Spectroscopy:

^{1}**H NMR** (600 MHz, $CDCl_3$, 298 K, δ): 7.60–7.58 (m, 2H), 7.45–7.42 (m, 2H), 7.37–7.34 (m, 2H), 7.20–7.17 (m, 1H), 7.13 (t, *J* = 2.3 Hz, 1H), 6.90 (ddd, *J* = 8.2, 2.6, 0.9 Hz, 1H), 3.87 (s, 3H).

^{13}**C NMR** (150 MHz, $CDCl_3$, 298 K, δ): 13C NMR (151 MHz, CDCl3) δ 160.1, 142.9, 141.3, 129.9, 128.9, 127.6, 127.4, 119.8, 113.1, 112.8, 55.5 ppm.

HRMS-EI (m/z) calculated for $C_{13}H_{12}O^+$ $[M]^+$, 184.0883; found, 184.0882; deviation: 0.41 ppm.

(4-Fluorophenyl)(methyl)(phenylimino)-λ^6-sulfanone (**S3**):

R*f* = 0.40 (DCM).

NMR Spectroscopy:

^{1}**H NMR** (500 MHz, $CDCl_3$, 298 K, δ): 8.02–7.95 (m, 2H), 7.23–7.17 (m, 2H), 7.16–7.11 (m, 2H), 7.02–6.97 (m, 2H), 6.89 (tt, *J* = 7.2, 1.2 Hz, 1H), 3.24 (s, 3H) ppm.

^{13}**C NMR** (126 MHz, $CDCl_3$, 298 K, δ): 165.7 (d, *J* = 255.7 Hz), 144.7, 135.4, 131.6 (d, *J* = 9.6 Hz), 129.2, 123.5, 122.1, 117.0 (d, *J* = 22.5 Hz), 46.3 ppm.

^{19}**F NMR** (471 MHz, $CDCl_3$, 298 K, δ): –104.6 (m) ppm.

HRMS-ESI (m/z) calculated for $C_{13}H_{12}NOSF^+$ $[M+H]^+$, 249.0618; found, 249.0619; deviation: –0.18 ppm.

Reaction without lithium 3-methoxybenzoate

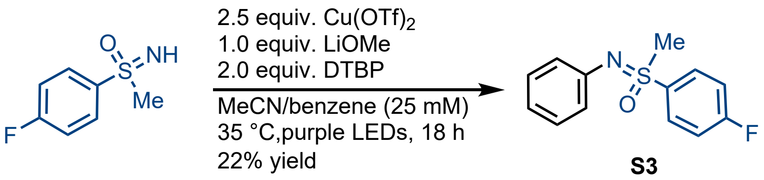

In a nitrogen-filled glovebox, a 16 mL borosilicate vial equipped with a magnetic stir bar was charged with $Cu(OTf)_2$ (181 mg, 0.500 mmol, 2.50 equiv.), LiOMe (7.6 mg, 0.200 mmol, 1.00 equiv.), 2,6-di-*tert*-butylpyridine (76.5 mg, 0.400 mmol, 2.00 equiv.) and (4-fluorophenyl)(imino)(methyl)-λ^6-sulfanone (86.5 mg, 0.500 mmol, 2.50 equiv.). Anhydrous MeCN (4.0 mL) and benzene (4.0 mL) were then added into the vial. The vial was sealed with a Teflon cap, taken out of the glovebox and placed 5 cm away from two purple LEDs (Kessil PR160L-390 nm LEDs). The reaction mixture was irradiated for 18 h while maintaining the temperature at approximately 35 °C through cooling with a fan. After irradiation, the reaction mixture was evaporated under reduced pressure to remove all volatiles. DCM (15 mL) was added to the residue, and the precipitation was removed by filtered through a Büchner funnel. The filtrate was evaporated under reduced pressure, and the residue was purified by chromatography on silica gel (EA/DCM = 1/50, v/v) to yield (4-fluorophenyl)(methyl)(phenylimino)-λ^6-sulfanone (**S3**) (27.7 mg, 111 μmol, 22%) as a colorless oil.

Deuterodecarboxylation

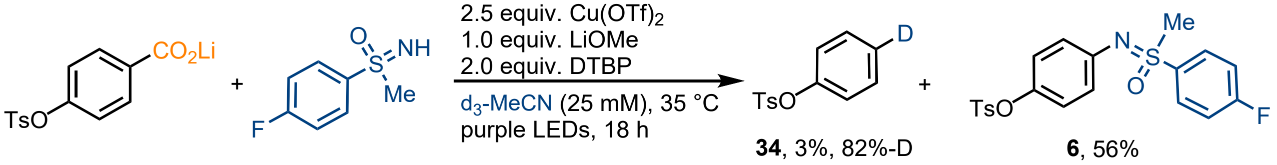

In a nitrogen-filled glovebox, a 16 mL borosilicate vial equipped with a magnetic stir bar was charged with lithium 4-(tosyloxy)benzoate (**33**) (59.6 mg, 0.200 mmol, 1.00 equiv.), $Cu(OTf)_2$ (181 mg, 0.500 mmol, 2.50 equiv.), LiOMe (7.6 mg, 0.200 mmol, 1.00 equiv.), 2,6-di-*tert*-butylpyridine (76.5 mg, 0.400 mmol, 2.00 equiv.) and (4-fluorophenyl)(imino)(methyl)-λ^6-sulfanone (86.5 mg, 0.500 mmol, 2.50 equiv.). Anhydrous d_3-MeCN (8.0 mL, c = 25 mM) was then added into the vial. The vial was sealed with a Teflon cap and placed 5 cm away from two purple LEDs (Kessil PR160L-390 nm LEDs). The reaction mixture was irradiated for 18 h while maintaining the temperature at approximately 35 °C through cooling with a fan. After irradiation, the reaction mixture was evaporated under reduced pressure to remove all volatiles. DCM (15 mL) was added to the residue, and the precipitation was removed by filtered through a Büchner funnel. The filtrate was evaporated under reduced pressure, and the residue was purified by chromatography on silica gel (EA/hexane = 1:50, v/v to EA/DCM = 1/50, v/v) to yield 4-(((4-fluorophenyl)(methyl)(oxo)-λ^6-sulfanylidene)amino)phenyl 4-methylbenzenesulfonate (**6**) (47.2 mg, 113 μmol, 56%) as a colorless solid and a mixture containing phenyl-4-*d* 4-methylbenzenesulfonate (**34**). The mixture was further purified by preparative TLC (DCM) to generate **34** (1.3 mg, 5 μmol, 3%) as a colorless solid. The deuterium ratio (82%) was determined by GC-MS.

Phenyl-4-*d* 4-methylbenzenesulfonate (**34,** 82%-D)

R*f* =0.73 (DCM).

NMR Spectroscopy:

^{1}H NMR (600 MHz, CD_2Cl_2, 298 K, δ): 7.71–7.67 (m, 2H), 7.37–7.33 (m, 2H), 7.32 (dd, *J* = 7.7, 4.9 Hz, 2H), 7.29 – 7.24 (m, 0.28H), 7.01–6.96 (m, 2H), 2.46 (s, 3H) ppm. Note:Since the deuterium ratio is 82%, the data reported here contains peaks belong to undeutered phenyl 4-methylbenzenesulfonate.

D NMR (92 MHz, CH_2Cl_2, 298 K, δ): 7.20 (s) ppm.

^{13}C NMR (151 MHz, $CDCl_3$, 298 K, δ): 149.8, 145.8, 132.4, 129.9, 129.7, 129.6, 128.5, 127.2, 122.5, 21.6. ppm. Note: The data contains peaks belong to undeutered phenyl 4-methylbenzenesulfonate.

HRMS-EI (m/z) calculated for $C_{13}H_{11}O_3SD^+$ $[M]^+$, 249.0564; found, 249.0568; deviation: –1.59 ppm.

Radical cyclisation experiment

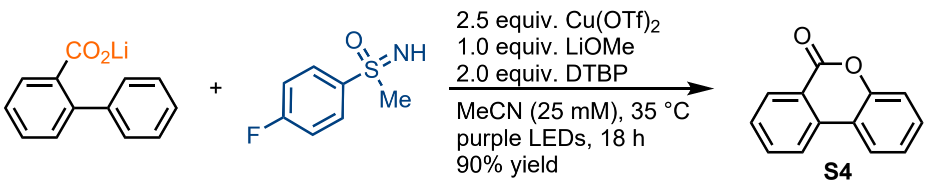

S4

In a nitrogen-filled glovebox, a 16 mL borosilicate vial equipped with a magnetic stir bar was charged with lithium [1,1'-biphenyl]-2-carboxylate (40.8 mg, 0.200 mmol, 1.00 equiv.), $Cu(OTf)_2$ (181 mg, 0.500 mmol, 2.50 equiv.), LiOMe (7.6 mg, 0.200 mmol, 1.00 equiv.), 2,6-di-*tert*-butylpyridine (76.5 mg, 0.400 mmol, 2.00 equiv.) and (4-fluorophenyl)(imino)(methyl)-λ^6-sulfanone (86.5 mg, 0.500 mmol, 2.50 equiv.). Anhydrous MeCN (4.0 mL) and benzene (4.0 mL) were then added into the vial. The vial was sealed with a Teflon cap, taken out of the glovebox and placed 5 cm away from two purple LEDs (Kessil PR160L-390 nm LEDs). The reaction mixture was irradiated for 18 h while maintaining the temperature at approximately 35 °C through cooling with a fan. After irradiation, the reaction mixture was evaporated under reduced pressure to remove all volatiles. DCM (15 mL) was added to the residue, and the precipitation was removed by filtered through a Büchner funnel. The filtrate was evaporated under reduced pressure, and the residue was purified by chromatography on silica gel (EA/pentane = 1/9, v/v) to afford 6*H*-benzo[*c*]chromen-6-one (**S4**) (35.3 mg, 180 μmol, 90%) as a colorless solid.

R*f* =0.31 (EA/pentane, 1/9, v/v).

NMR Spectroscopy:

^{1}H NMR (500 MHz, $CDCl_3$, 298 K, δ): 8.36 (dd, *J* = 7.9, 1.4 Hz, 1H), 8.07 (dd, *J* = 8.1, 1.1 Hz, 1H), 8.01 (dd, *J* = 7.9, 1.6 Hz, 1H), 7.79 (ddd, *J* = 8.4, 7.2, 1.4 Hz, 1H), 7.55 (ddd, *J* = 8.1, 7.2, 1.1 Hz, 1H), 7.45 (ddd, *J* = 8.4, 7.1, 1.5 Hz, 1H), 7.36–7.27 (m, 2H) ppm.

^{13}C NMR (126 MHz, $CDCl_3$, 298 K, δ): 161.2, 151.3, 134.9, 134.8, 130.6, 130.5, 128.9, 124.6, 122.8, 121.8, 121.29, 118.1, 117.8 ppm.

HRMS-EI (m/z) calculated for $C_{13}H_8O_2^+$ $[M]^+$, 196.0519; found, 196.0518; deviation: 0.20 ppm.

Decarboxylative sulfoximination of 4-fluorobenzoic acid derived oxime ester

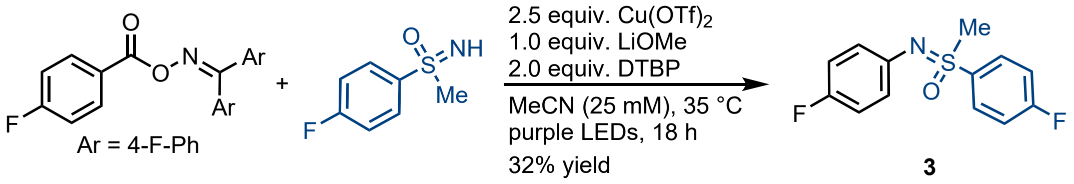

In a nitrogen-filled glovebox, a 16 mL borosilicate vial equipped with a magnetic stir bar was charged with 4-fluorobenzoic acid derived oxime ester (71.1 mg, 0.200 mmol, 1.00 equiv.), $Cu(OTf)_2$ (181 mg, 0.500 mmol, 2.50 equiv.), LiOMe (7.6 mg, 0.200 mmol, 1.00 equiv.), 2,6-di-*tert*-butylpyridine (76.5 mg, 0.400 mmol, 2.00 equiv.) and (4-fluorophenyl)(imino)(methyl)-λ^6-sulfanone (86.5 mg, 0.500 mmol, 2.50 equiv.). Anhydrous MeCN (8.0 mL, c = 25 mM) was then added into the vial. The vial was sealed with a Teflon cap and placed 5 cm away from two purple LEDs (Kessil PR160L-390 nm LEDs). The reaction mixture was irradiated for 18 h while maintaining the temperature at approximately 35 °C through cooling with a fan. After irradiation, the reaction mixture was evaporated under reduced pressure to remove all volatiles. DCM (15 mL) was added to the residue, and the precipitation was removed by filtered through a Büchner funnel. The filtrate was evaporated under reduced pressure, and the residue was purified by chromatography on silica gel (EA/DCM = 1/50, v/v) to yield (4-fluorophenyl)((4-fluorophenyl)imino)(methyl)-λ^6-sulfanone (**3**) (17.0 mg, 64 µmol, 32%) as a slightly yellow oil.

Example of decarboxylative amination

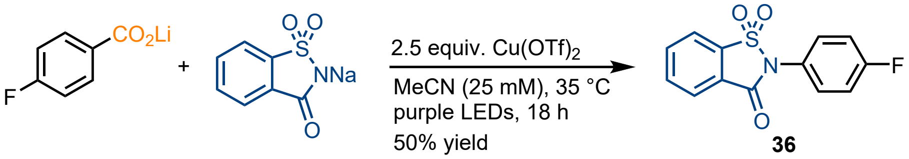

In a nitrogen-filled glovebox, a 16 mL borosilicate vial equipped with a magnetic stir bar was charged with lithium 4-fluorobenzoate (29.2 mg, 0.200 mmol, 1.00 equiv.), $Cu(OTf)_2$ (181 mg, 0.500 mmol, 2.50 equiv.), and sodium saccharin (**35**) (82.1 mg, 0.400 mmol, 2.00 equiv.). Anhydrous MeCN (8.0 mL, c = 25 mM) was then added into the vial. The vial was sealed with a Teflon cap and placed 5 cm away from two purple LEDs (Kessil PR160L-390 nm LEDs). The reaction mixture was irradiated for 18 h while maintaining the temperature at approximately 35 °C through cooling with a fan. After irradiation, the reaction mixture was evaporated under reduced pressure to remove all volatiles. The residue was purified by chromatography on silica gel (EA/hexanes = 1/5, v/v) to afford a mixture, then the mixture was further purified by preparative TLC (DCM) to yield 2-(4-fluorophenyl)benzo[*d*]isothiazol-3(2*H*)-one 1,1-dioxide (**36**) (27.9 mg, 101 µmol, 50%) as a white solid.

R*f* = 0.7 (DCM).

NMR Spectroscopy:

^{1}H NMR (500 MHz, $CDCl_3$, 298 K, δ): 8.21–8.10 (m, 1H), 8.00 (dt, *J* = 7.8, 0.9 Hz, 1H), 7.93 (td, *J* = 7.6, 1.3 Hz, 1H), 7.89 (dd, *J* = 7.5, 1.3 Hz, 1H), 7.59–7.46 (m, 2H), 7.27–7.21 (m, 2H) ppm.

^{13}C NMR (126 MHz, $CDCl_3$, 298 K, δ): 164.6, 162.6, 158.5, 137.6, 135.3, 134.7, 131.1, 131.1, 127.2, 125.8, 124.5, 124.5, 121.5, 117.3, 117.2 ppm.

^{19}F NMR (471 MHz, $CDCl_3$, 298 K, δ): –109.8 (m) ppm.

HRMS-EI (m/z) calculated for $C_{13}H_8NOSF^+$ $[M]^+$, 277.0203; found, 277.0209; deviation: –1.86 ppm.

REFERENCES

1. Fulmer, G. R.; Miller, A. J. M.; Sherden, N. H.; Gottlieb, H. E.; Nudelman, A.; Stoltz, B. M.; Bercaw, J. E.; Goldberg, K. I. NMR Chemical Shifts of Trace Impurities: Common Laboratory Solvents, Organics, and Gases in Deuterated Solvents Relevant to the Organometallic Chemist. *Organometallics* **2010**, *29*, 2176–2179.
2. Wang, B.; Han, X.; Li, J.; Li, C.; Liu, H. Sulfoximines-Assisted Rh(III)-Catalyzed C–H Activation and Intramolecular Annulation for the Synthesis of Fused Isochromeno-1,2-Benzothiazines Scaffolds under Room Temperature *Molecules* **2020***, 25,* 2515.
3. Xu, P.; López-Rojas, P.; Ritter, T. Radical Decarboxylative Carbometalation of Benzoic Acids: A Solution to Aromatic Decarboxylative Fluorination, *J. Am. Chem. Soc.* **2021**, *143*, 5349-5354.
4. Lill, A.; Scholich, K.; Stark, H. Synthesis of Novel Dansyl-Labeled Celecoxib Derivatives. *Tetrahedron Lett*. **2013**, *54*, 6682–6686.
5. Su, W.; Xu, P.; Ritter, T. Decarboxylative Hydroxylation of Benzoic acids, *Angew. Chem. Int. Ed.* under revision.

SPECTROSCOPIC DATA

^{1}H NMR of (4-fluorophenyl)((4-fluorophenyl)imino)(methyl)-λ^6-sulfanone (3)

$CDCl_3$, 298 K

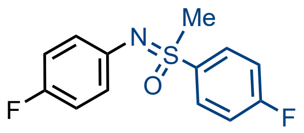

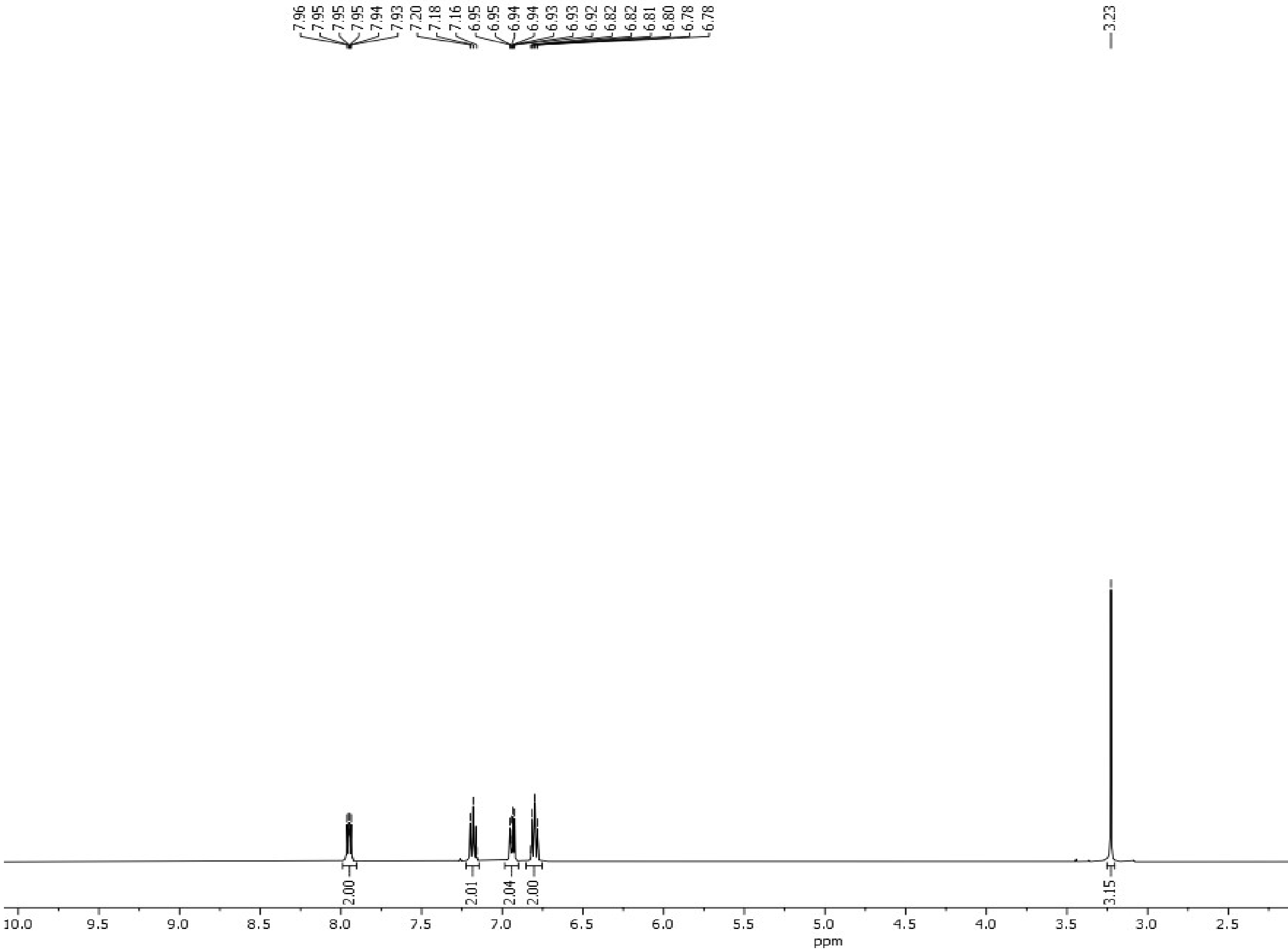

^{13}C NMR of (4-fluorophenyl)((4-fluorophenyl)imino)(methyl)-λ^6-sulfanone (3)

$CDCl_3$, 298 K

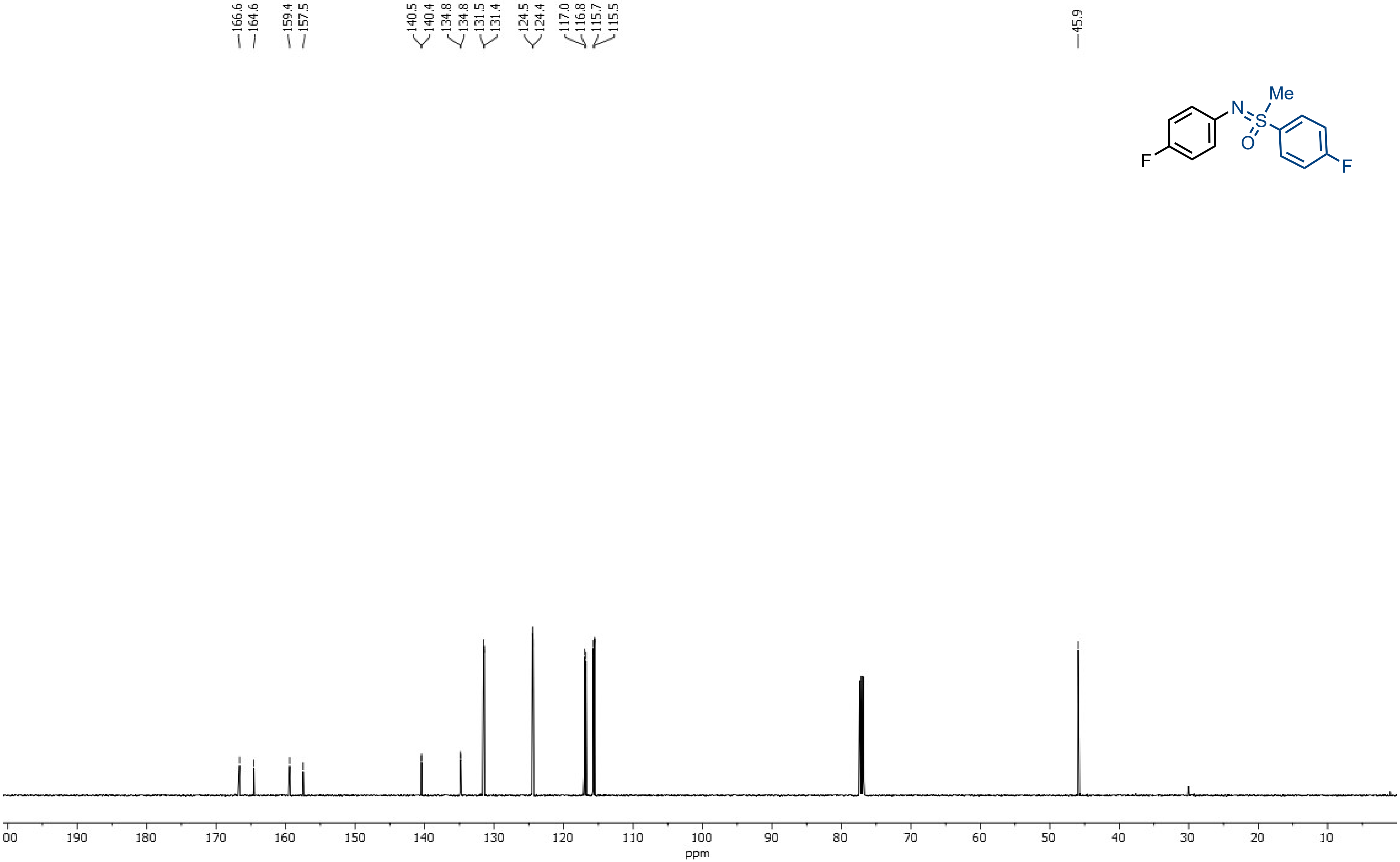

^{19}F NMR of (4-fluorophenyl)((4-fluorophenyl)imino)(methyl)-λ^6-sulfanone (3)

$CDCl_3$, 298 K

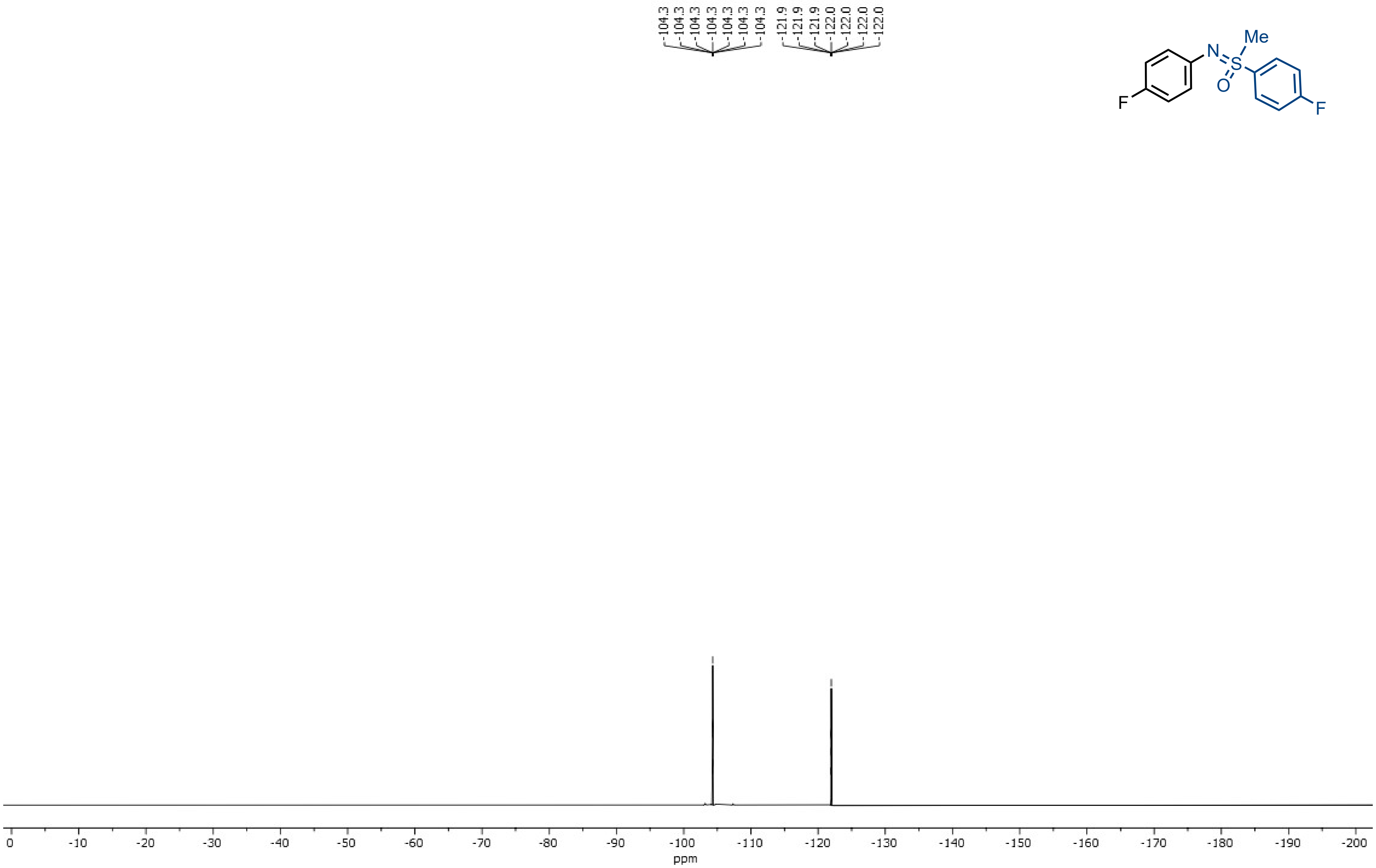

^{1}H NMR of (4-fluorophenyl)(methyl)((4-(methylsulfonyl)phenyl)imino)-λ[6]-sulfanone (4)

CDCl$_3$, 298 K

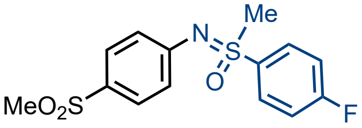

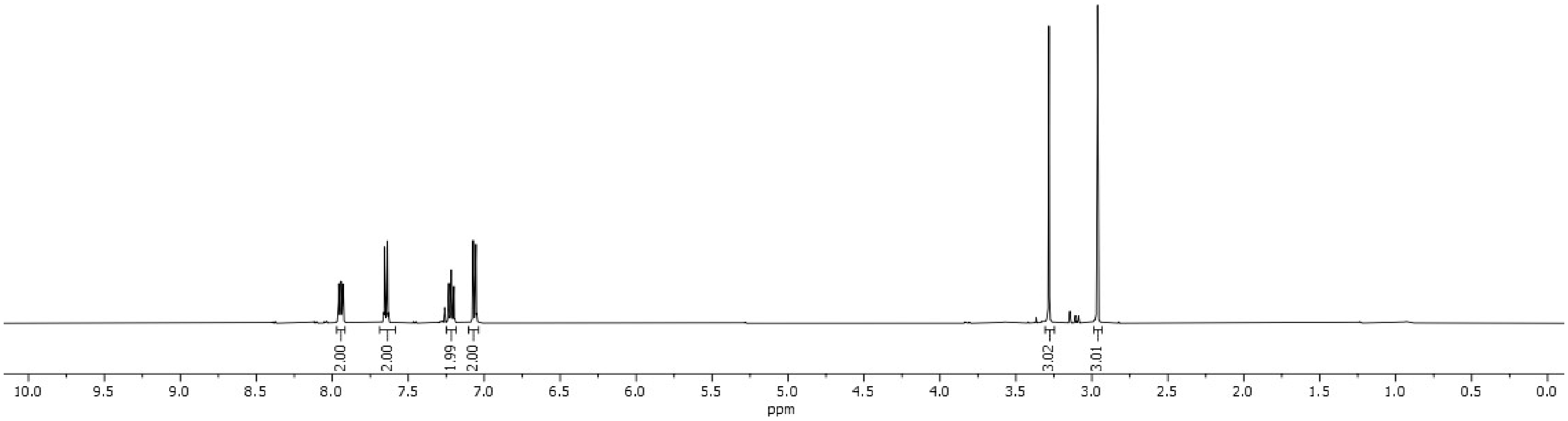

^{13}C NMR of (4-fluorophenyl)(methyl)((4-(methylsulfonyl)phenyl)imino)-λ^6-sulfanone (4)

$CDCl_3$, 298 K

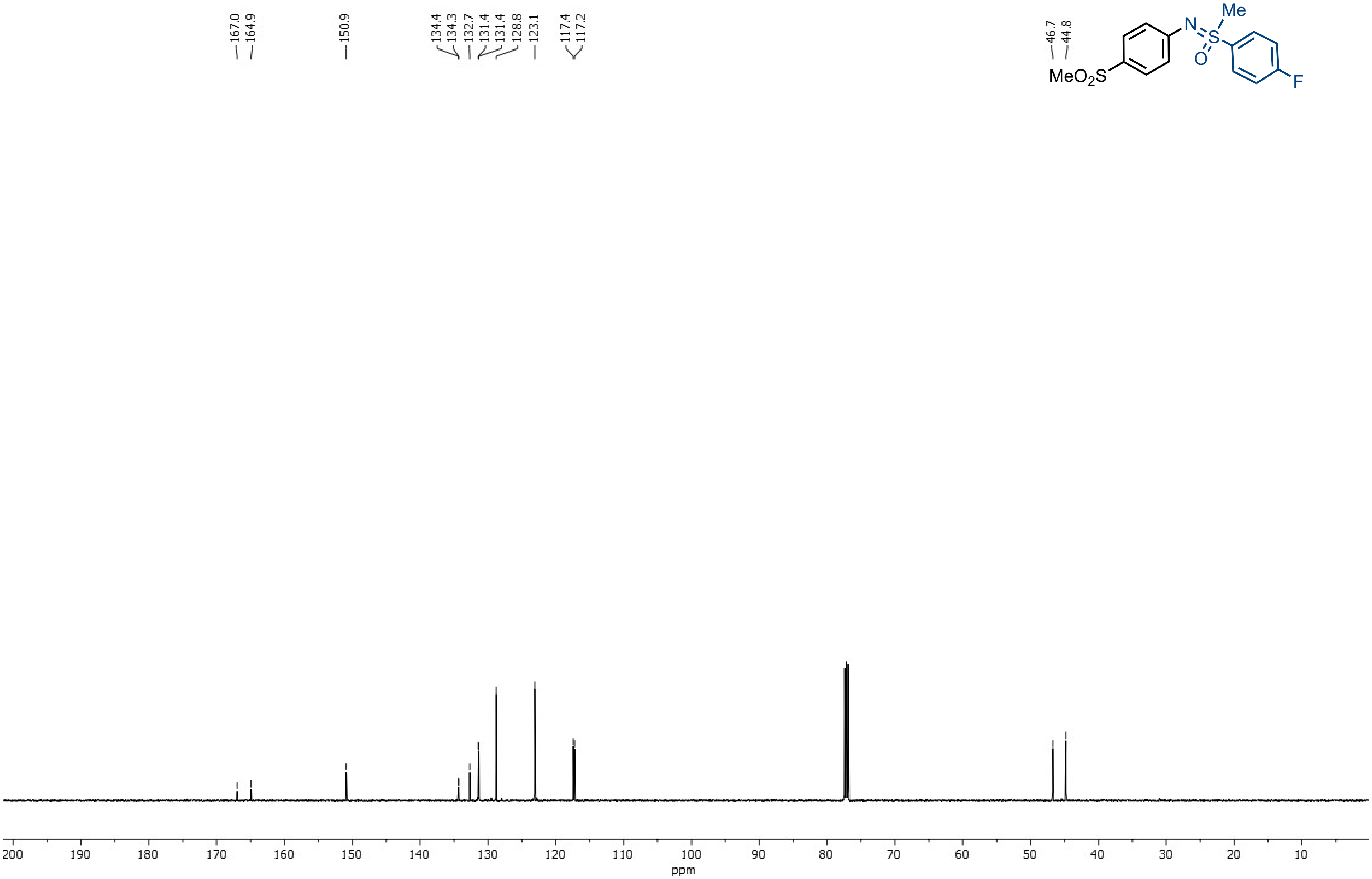

^{19}F NMR of (4-fluorophenyl)(methyl)((4-(methylsulfonyl)phenyl)imino)-λ^6-sulfanone (4)

$CDCl_3$, 298 K

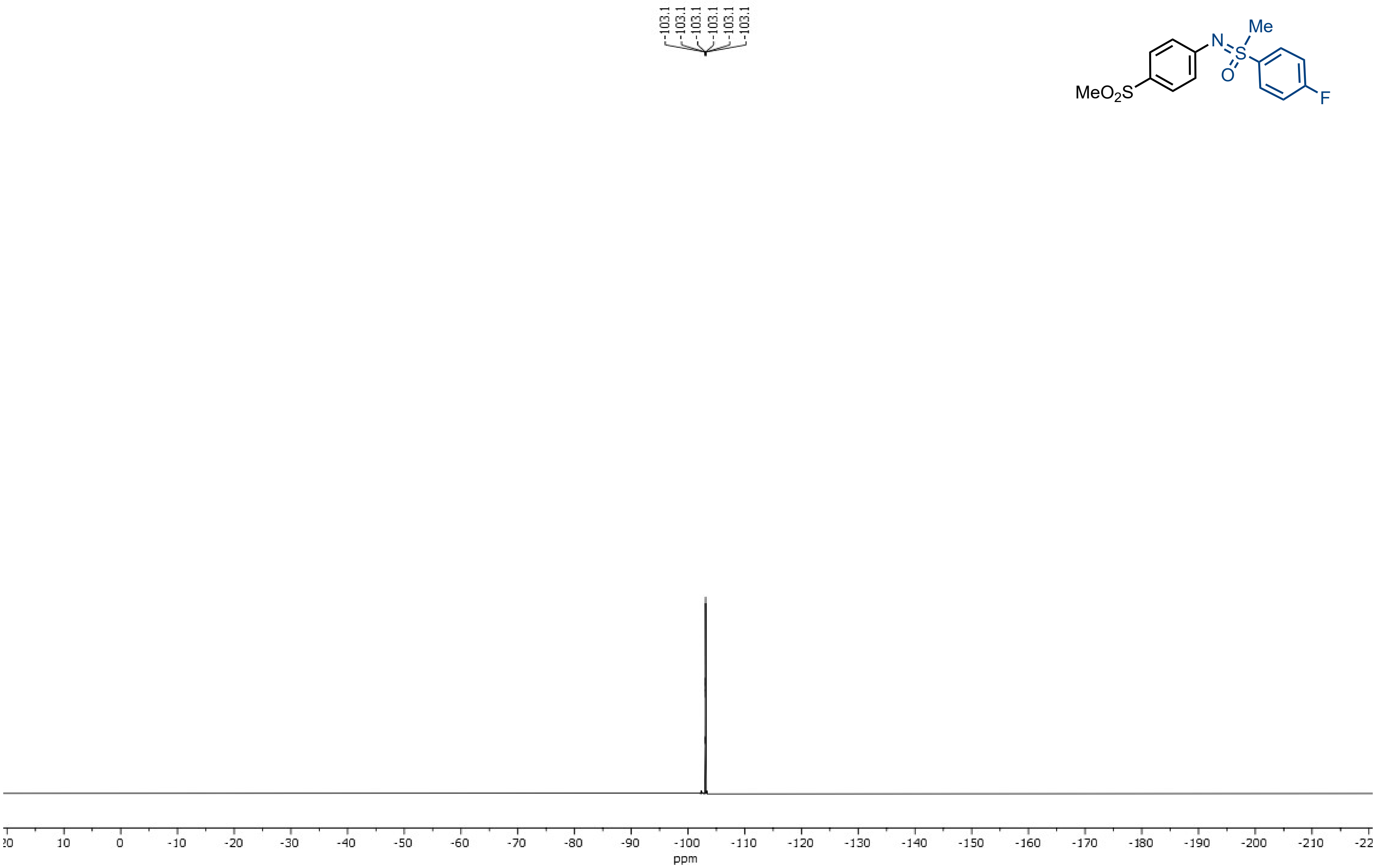

^{1}H NMR of (4-fluorophenyl)(methyl)((4-(trifluoromethyl)phenyl)imino)-λ[6]-sulfanone (5)

$CDCl_3$, 298 K

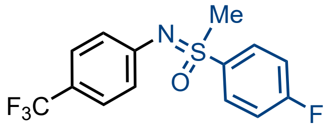

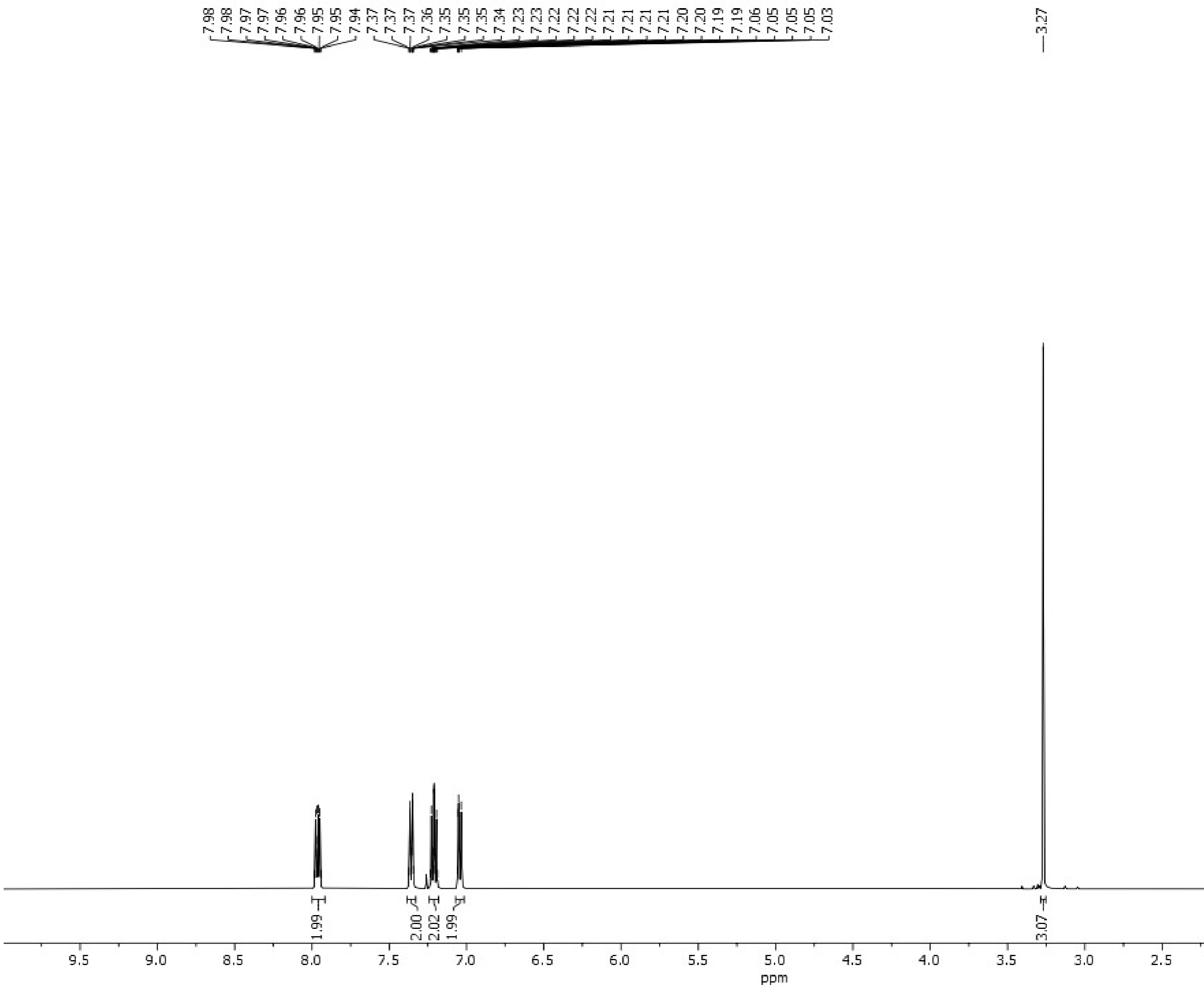

^{13}C NMR of (4-fluorophenyl)(methyl)((4-(trifluoromethyl)phenyl)imino)-λ^6-sulfanone (5)

$CDCl_3$, 298 K

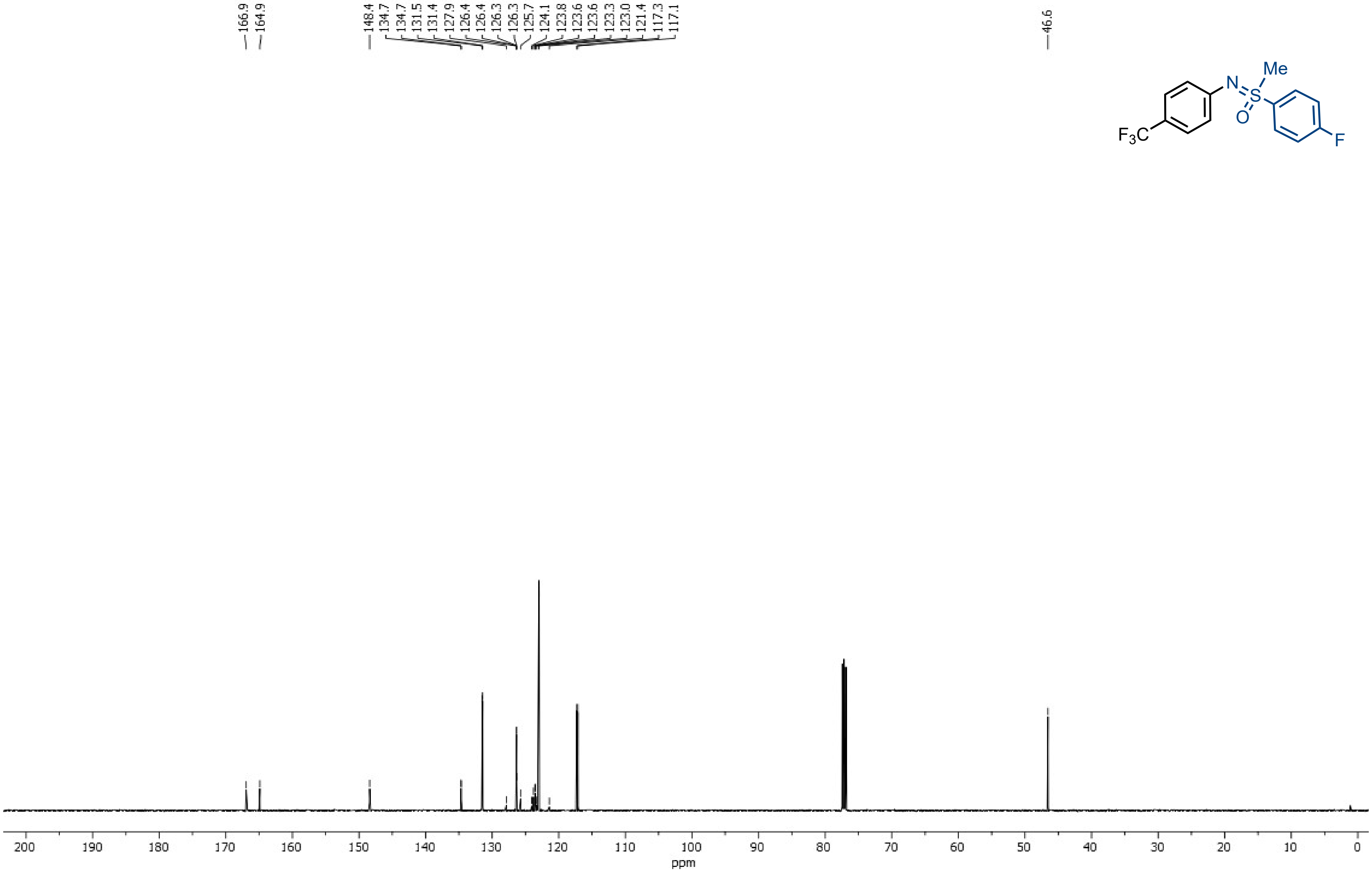

^{19}F NMR of (4-fluorophenyl)(methyl)((4-(trifluoromethyl)phenyl)imino)-λ^{6}-sulfanone (5)

$CDCl_3$, 298 K

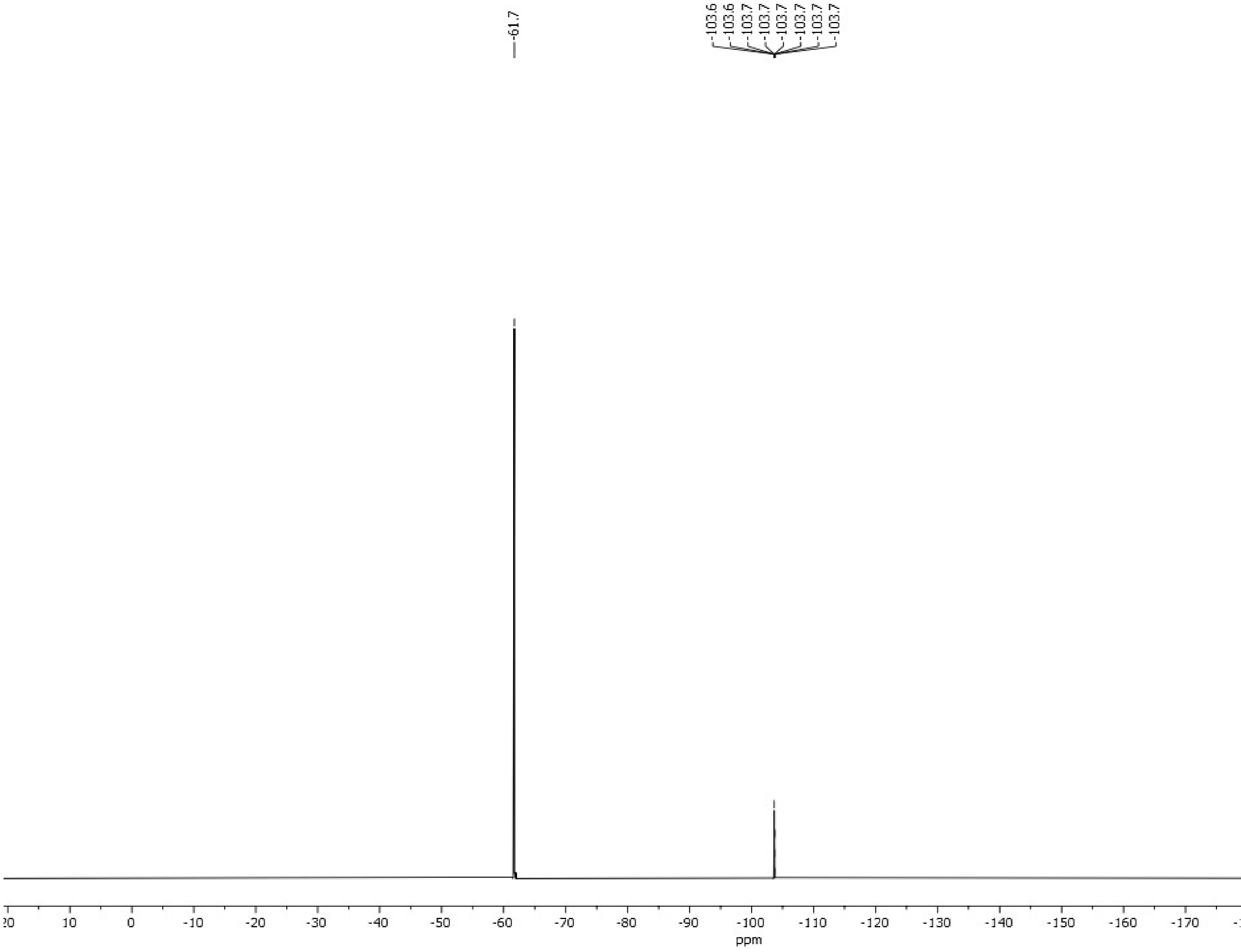

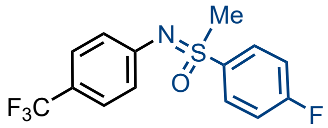

^{1}H NMR of 4-(((4-fluorophenyl)(methyl)(oxo)-λ^6-sulfanylidene)amino)phenyl 4-methylbenzenesulfonate (6)

CDCl$_3$, 298 K

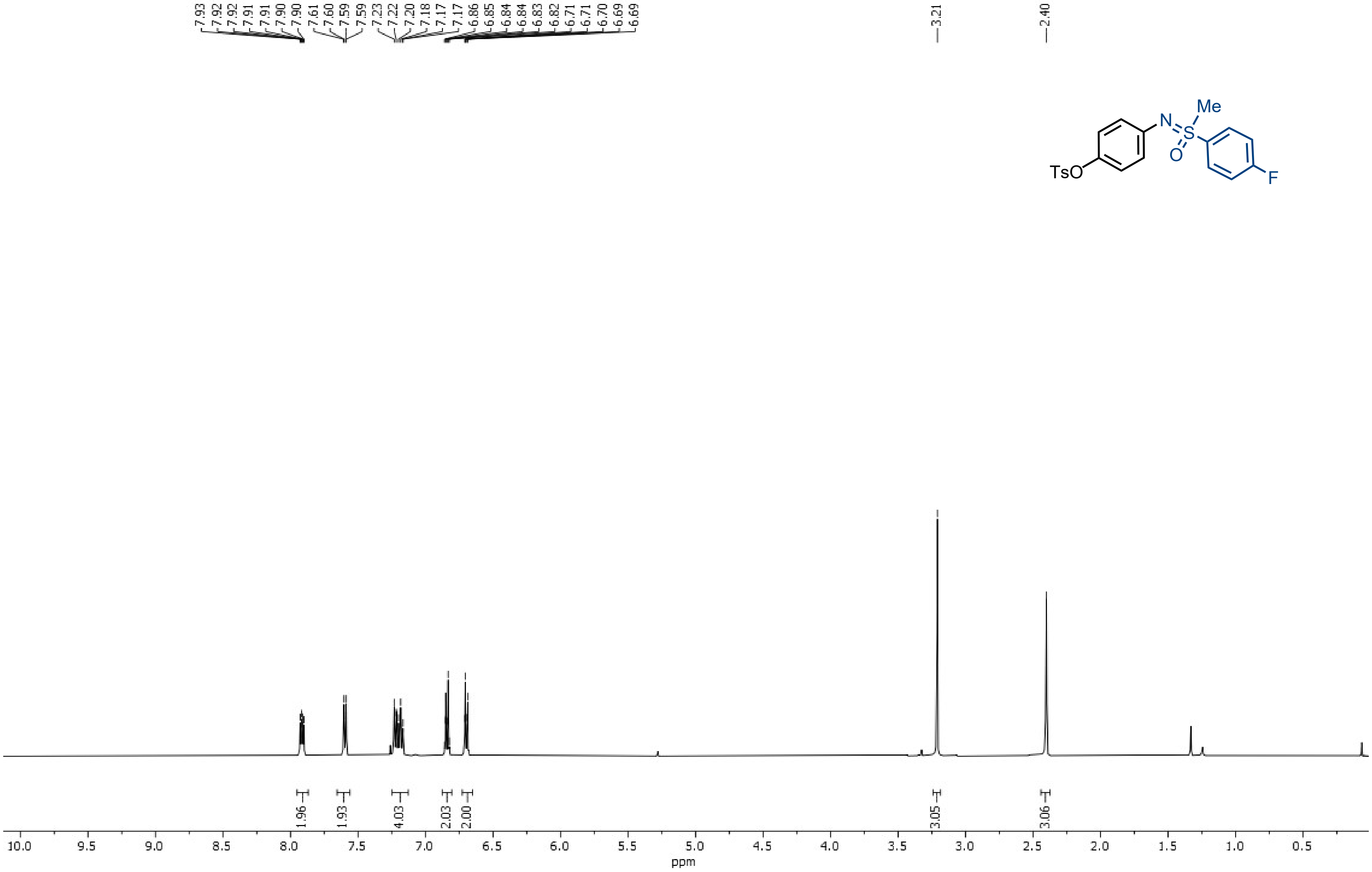

^{13}C NMR of 4-(((4-fluorophenyl)(methyl)(oxo)-λ^6-sulfanylidene)amino)phenyl 4-methylbenzenesulfonate (6)

$CDCl_3$, 298 K

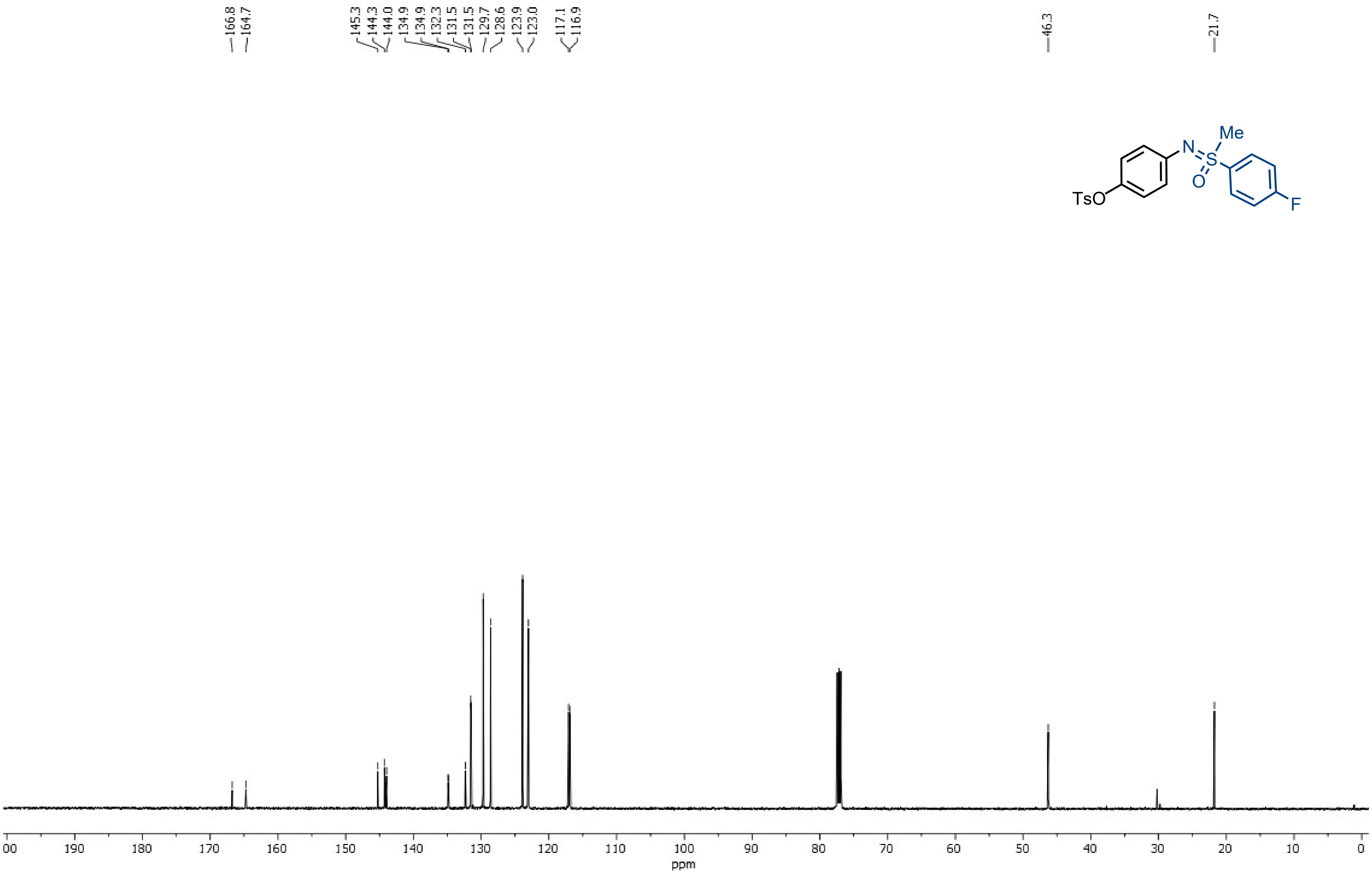

^{19}F NMR of 4-(((4-fluorophenyl)(methyl)(oxo)-λ^6-sulfanylidene)amino)phenyl 4-methylbenzenesulfonate (6)

$CDCl_3$, 298 K

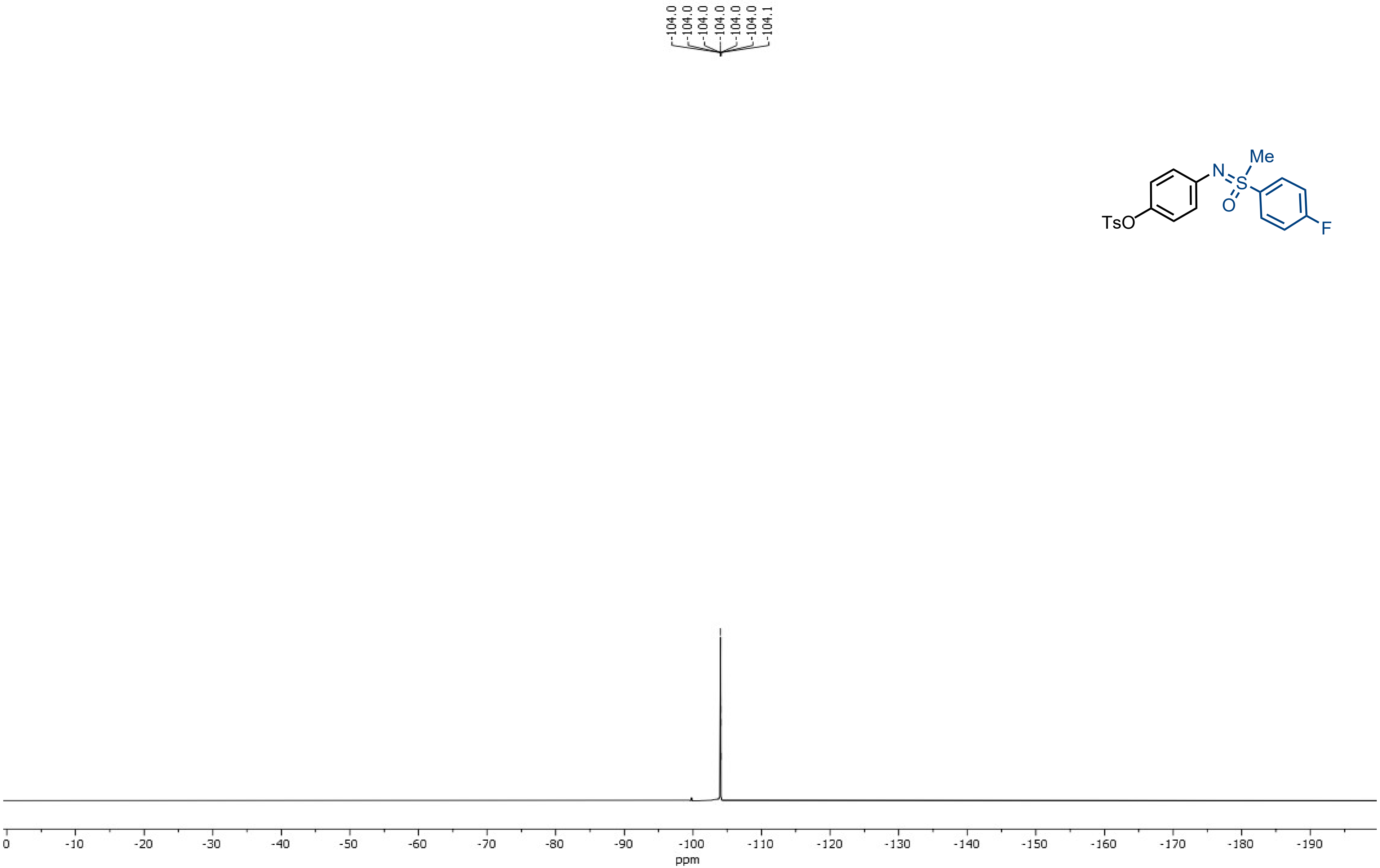

^{1}H NMR of ((4-bromophenyl)imino)(4-fluorophenyl)(methyl)-λ[6]-sulfanone (7)

$CDCl_3$, 298 K

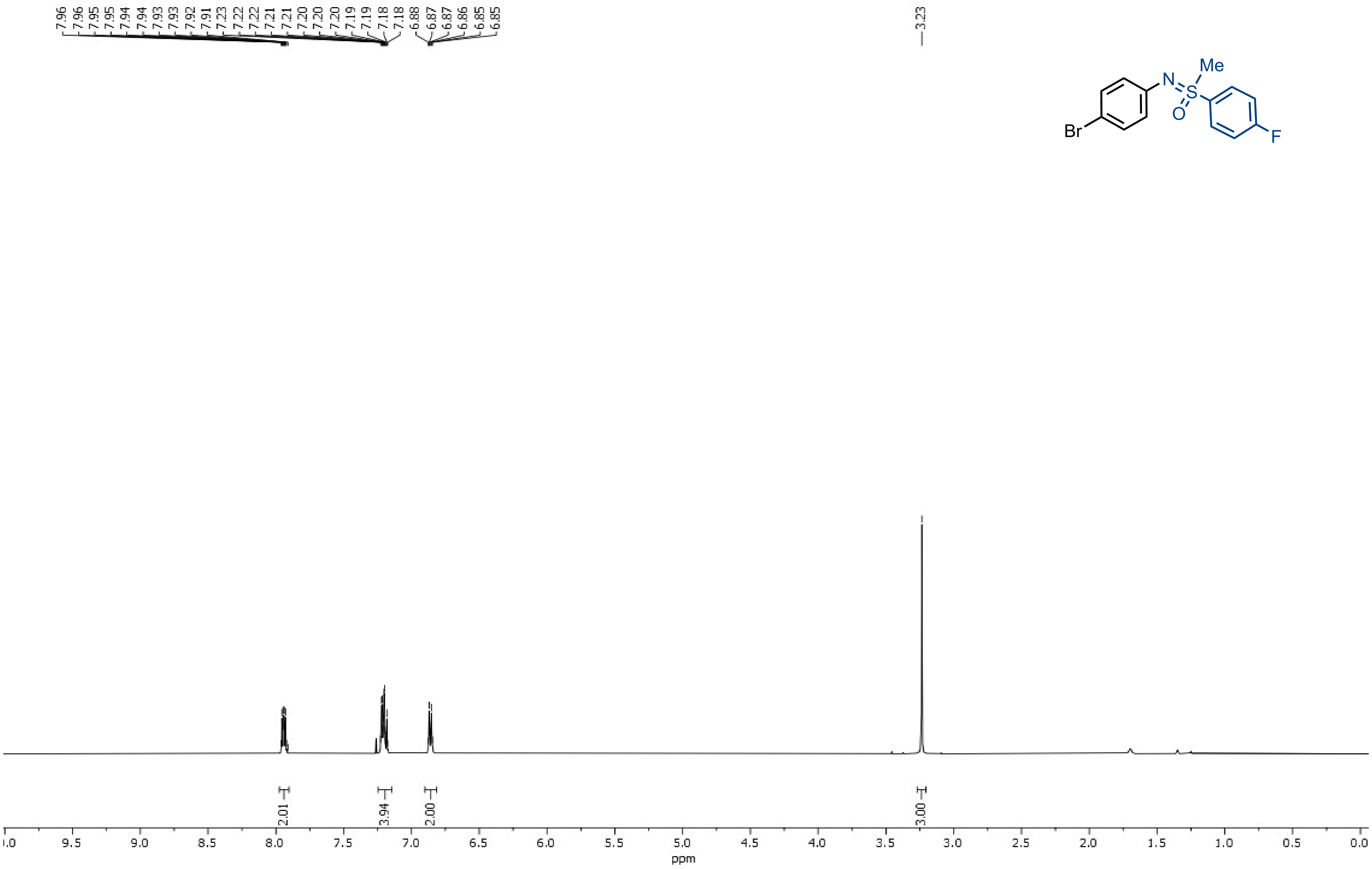

^{13}C NMR of ((4-bromophenyl)imino)(4-fluorophenyl)(methyl)-λ^6-sulfanone (7)

$CDCl_3$, 298 K

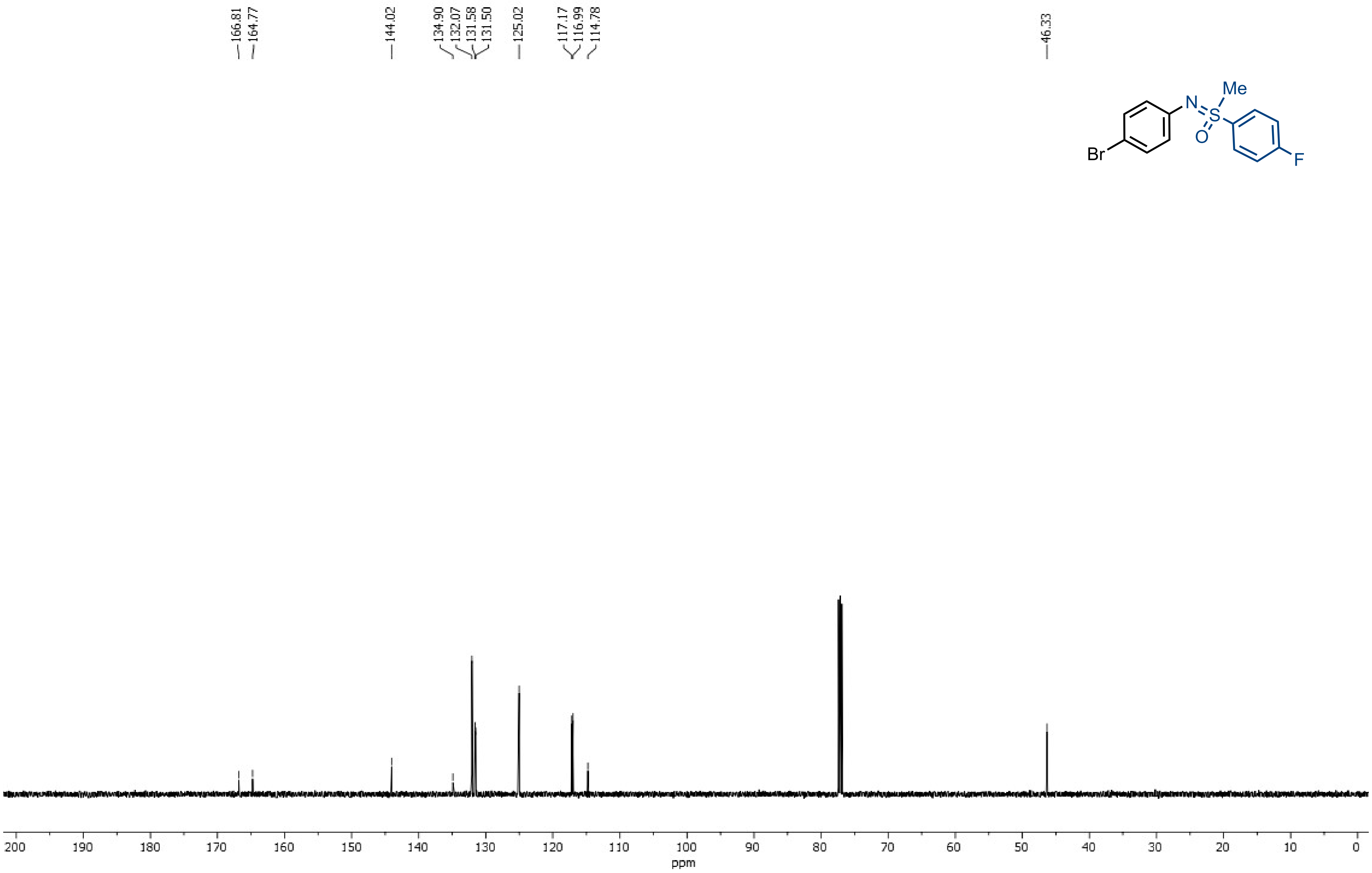

^{19}F NMR of ((4-bromophenyl)imino)(4-fluorophenyl)(methyl)-λ^6-sulfanone (7)

$CDCl_3$, 298 K

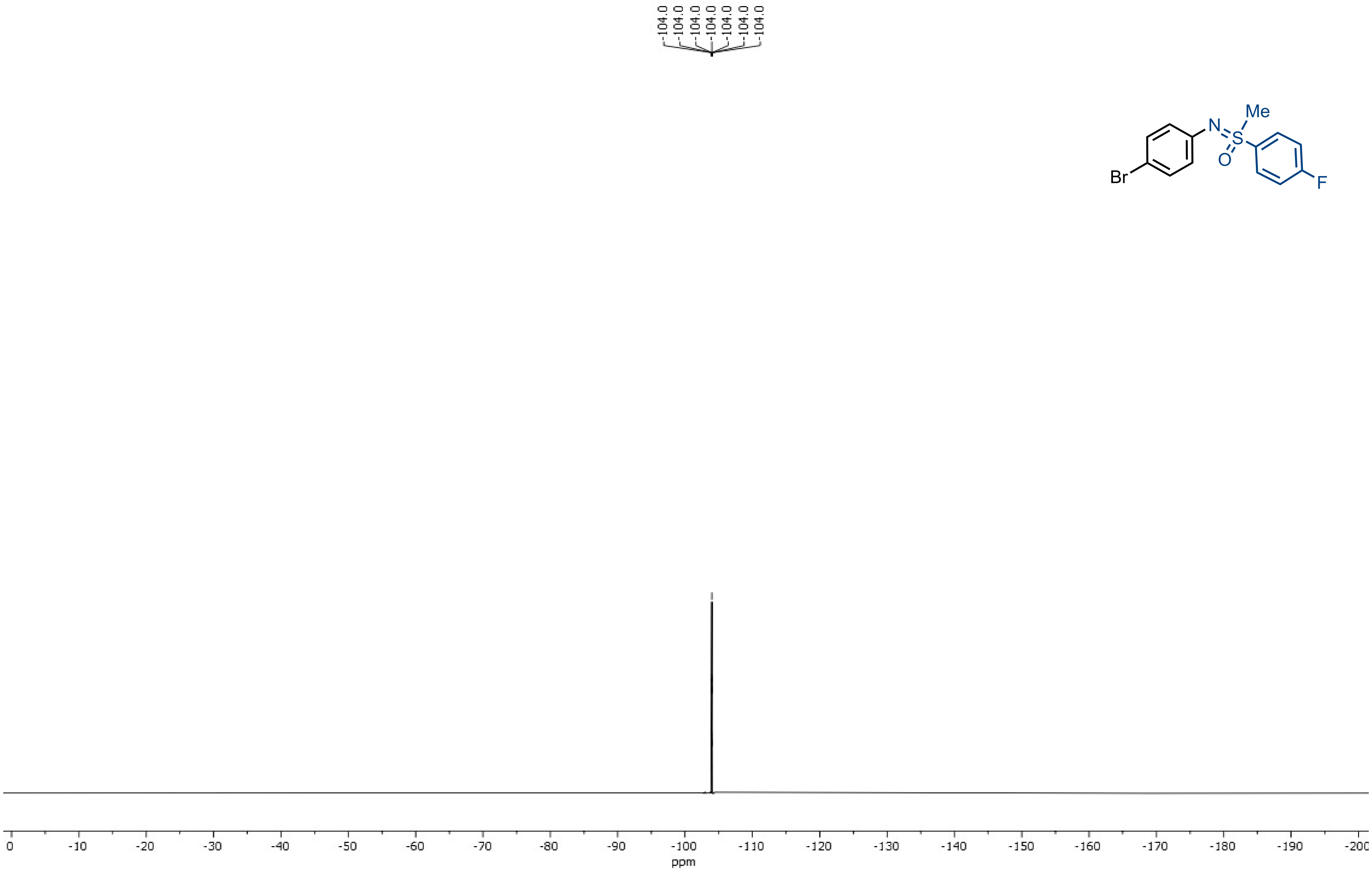

^{1}H NMR of (4-fluorophenyl)((3-(5-(2-fluorophenyl)-1,2,4-oxadiazol-3-yl)phenyl)imino)(methyl)-λ^6-sulfanone (8)

$CDCl_3$, 298 K

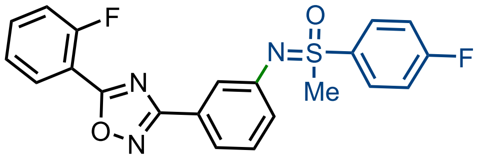

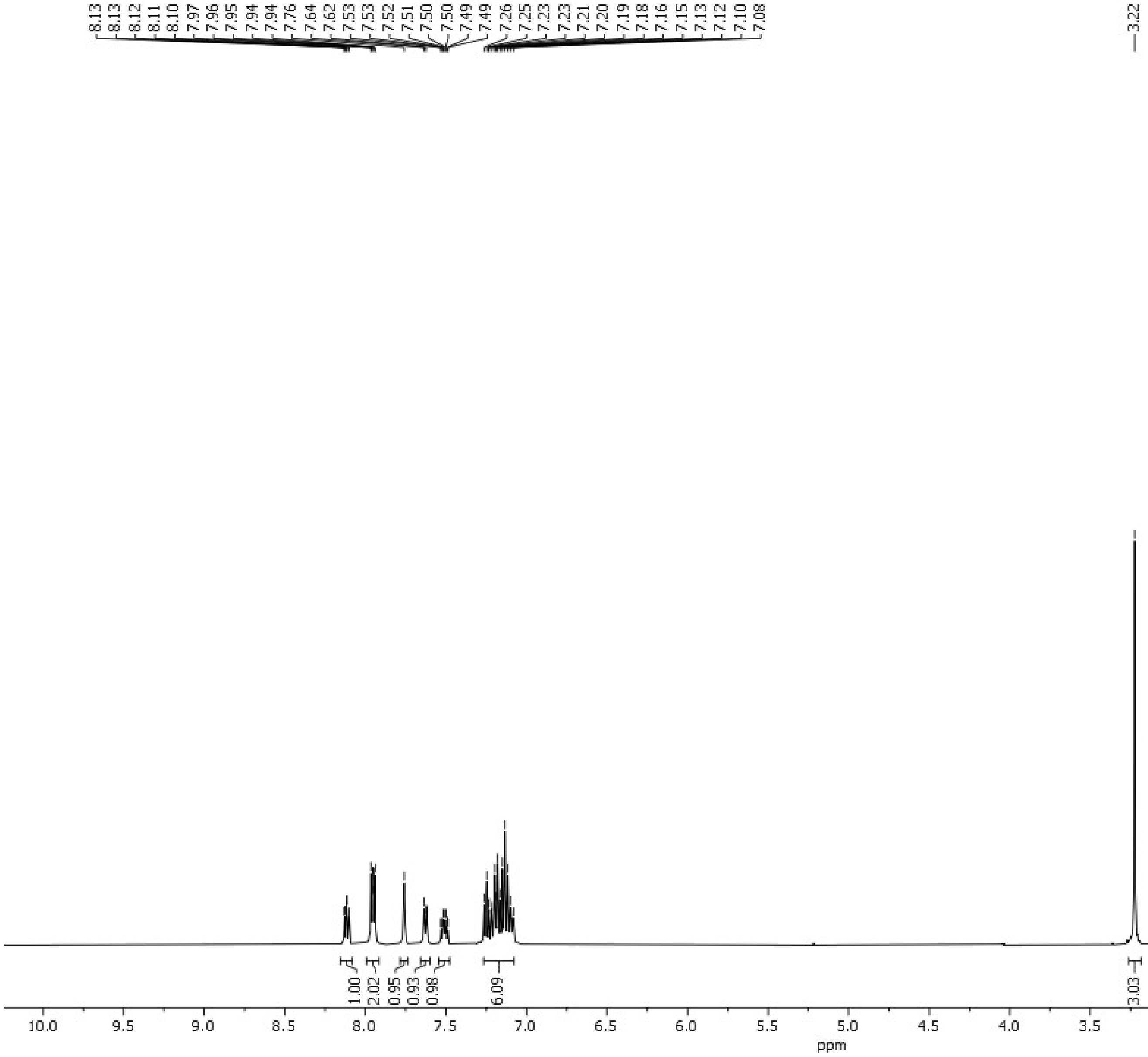

^{13}C NMR of (4-fluorophenyl)((3-(5-(2-fluorophenyl)-1,2,4-oxadiazol-3-yl)phenyl)imino)(methyl)-λ^{6}-sulfanone (8)

$CDCl_3$, 298 K

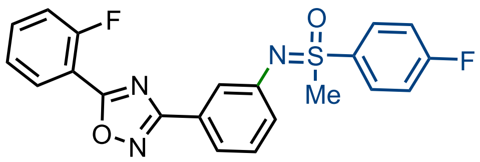

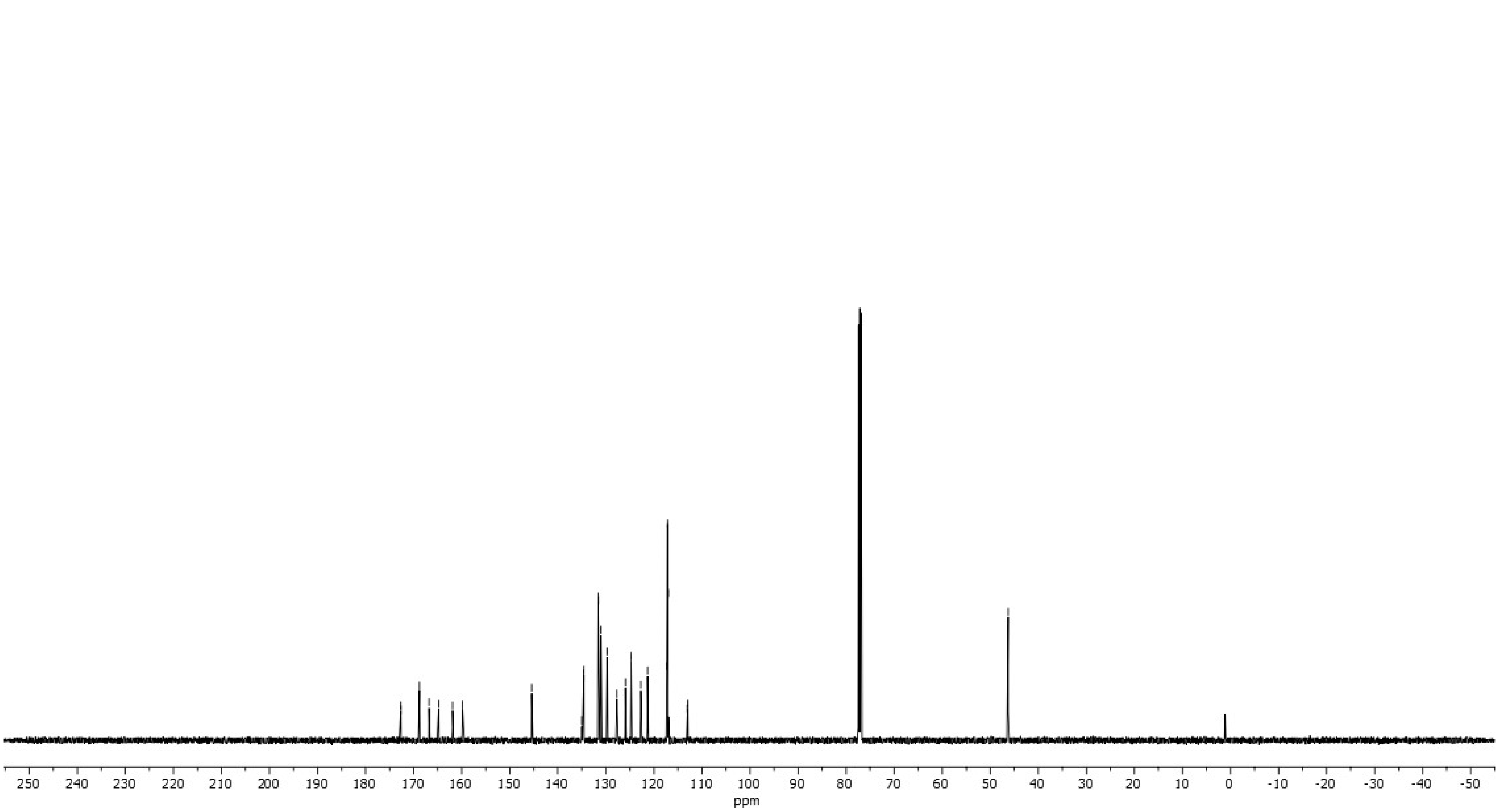

^{19}F NMR of (4-fluorophenyl)((3-(5-(2-fluorophenyl)-1,2,4-oxadiazol-3-yl)phenyl)imino)(methyl)-λ^6-sulfanone (8)

$CDCl_3$, 298 K

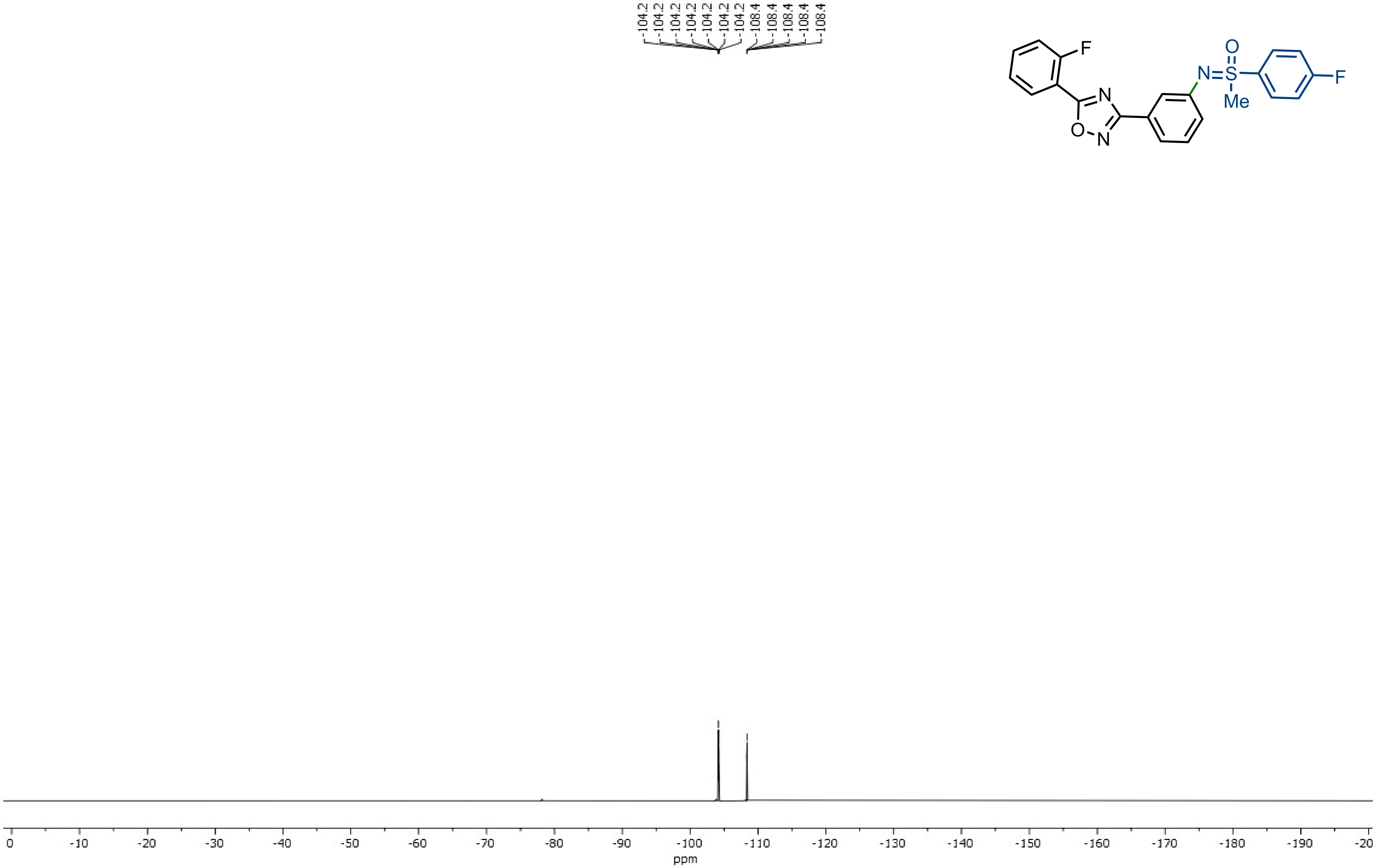

^{1}H NMR of ((4-cyanophenyl)imino)(4-fluorophenyl)(methyl)-λ^6-sulfanone (9)

$CDCl_3$, 298 K

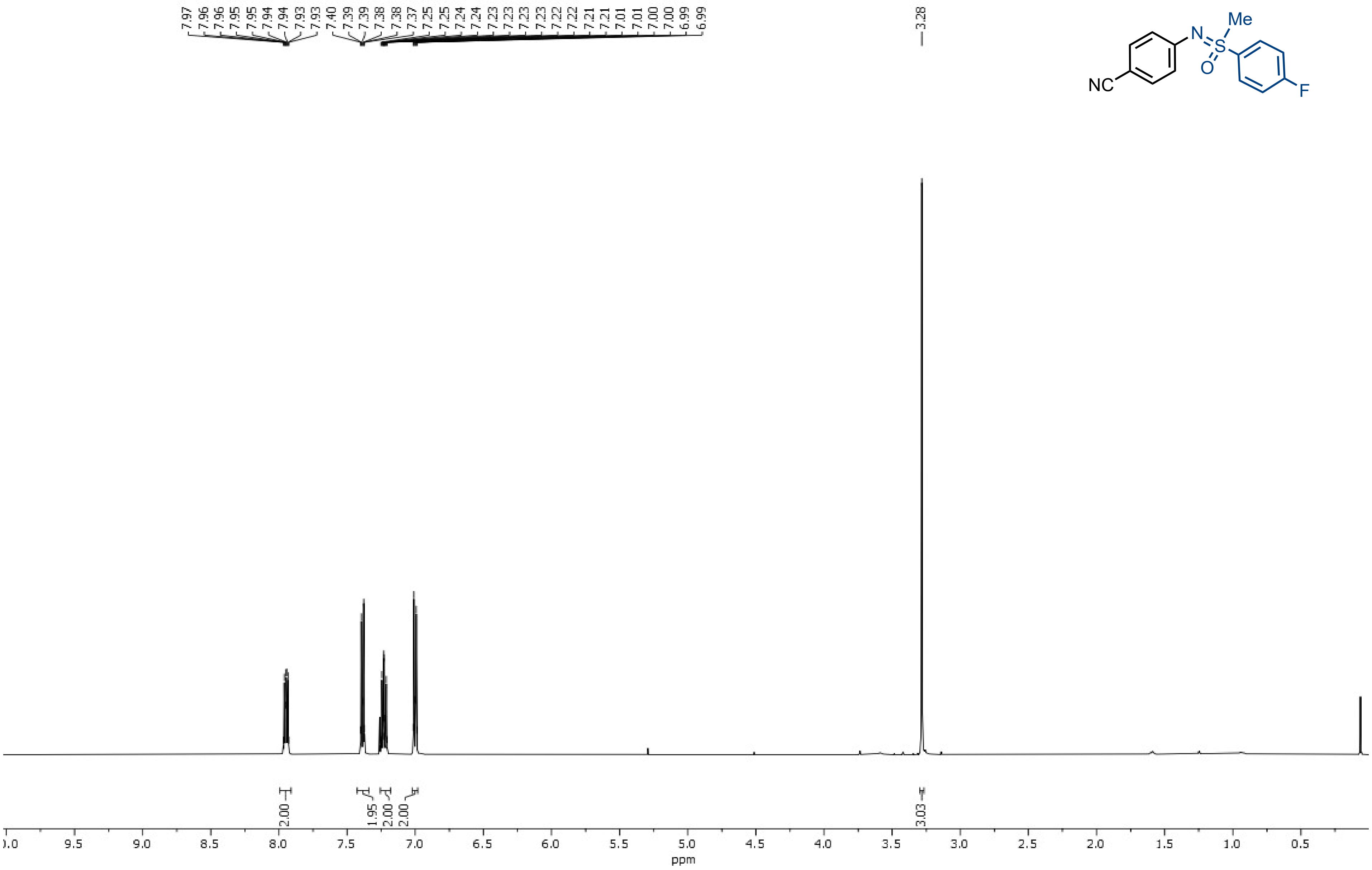

^{13}C NMR of ((4-cyanophenyl)imino)(4-fluorophenyl)(methyl)-λ^6-sulfanone (9)

$CDCl_3$, 298 K

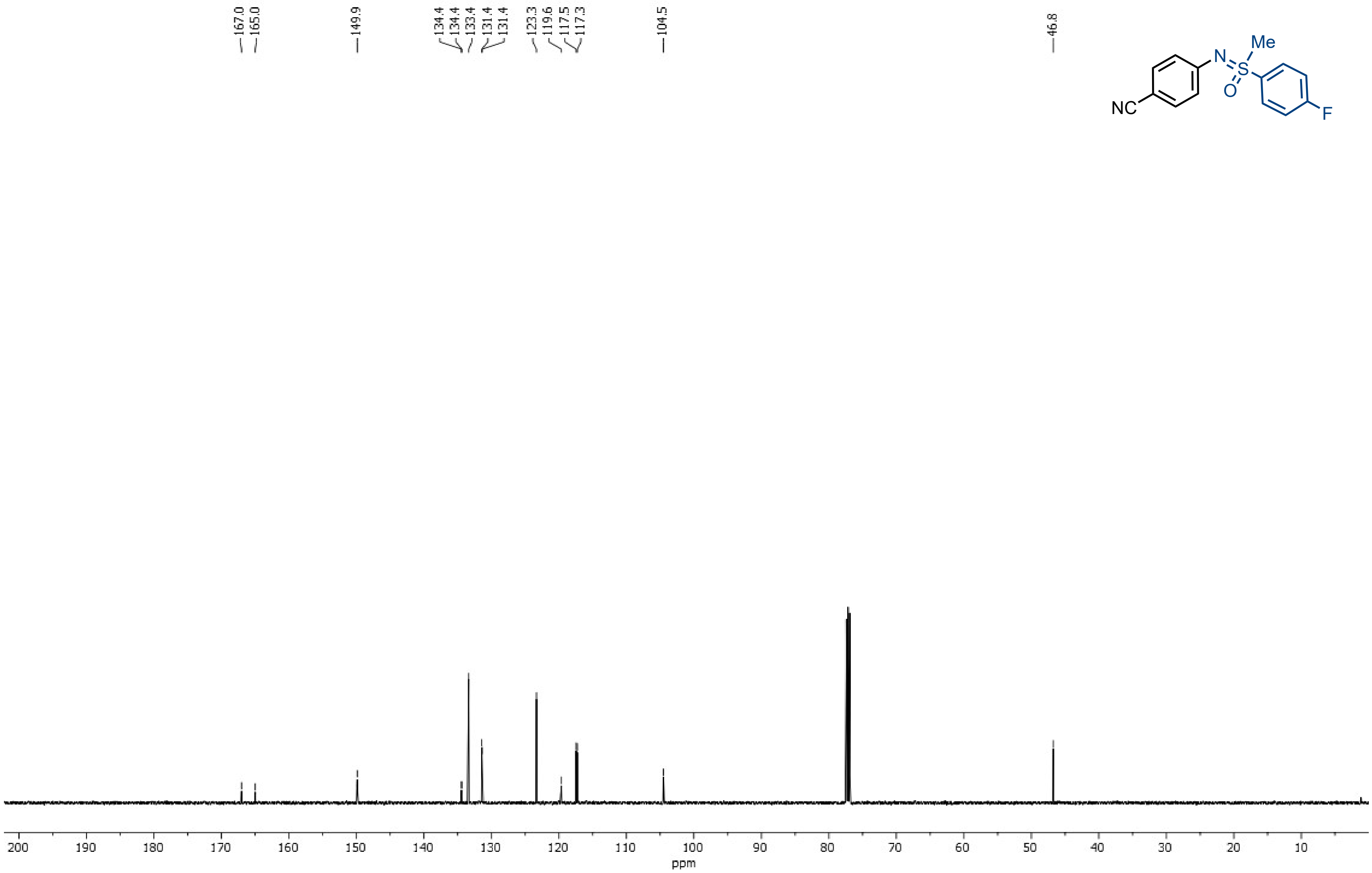

^{19}F NMR of ((4-cyanophenyl)imino)(4-fluorophenyl)(methyl)-λ^{6}-sulfanone (9)

$CDCl_3$, 298 K

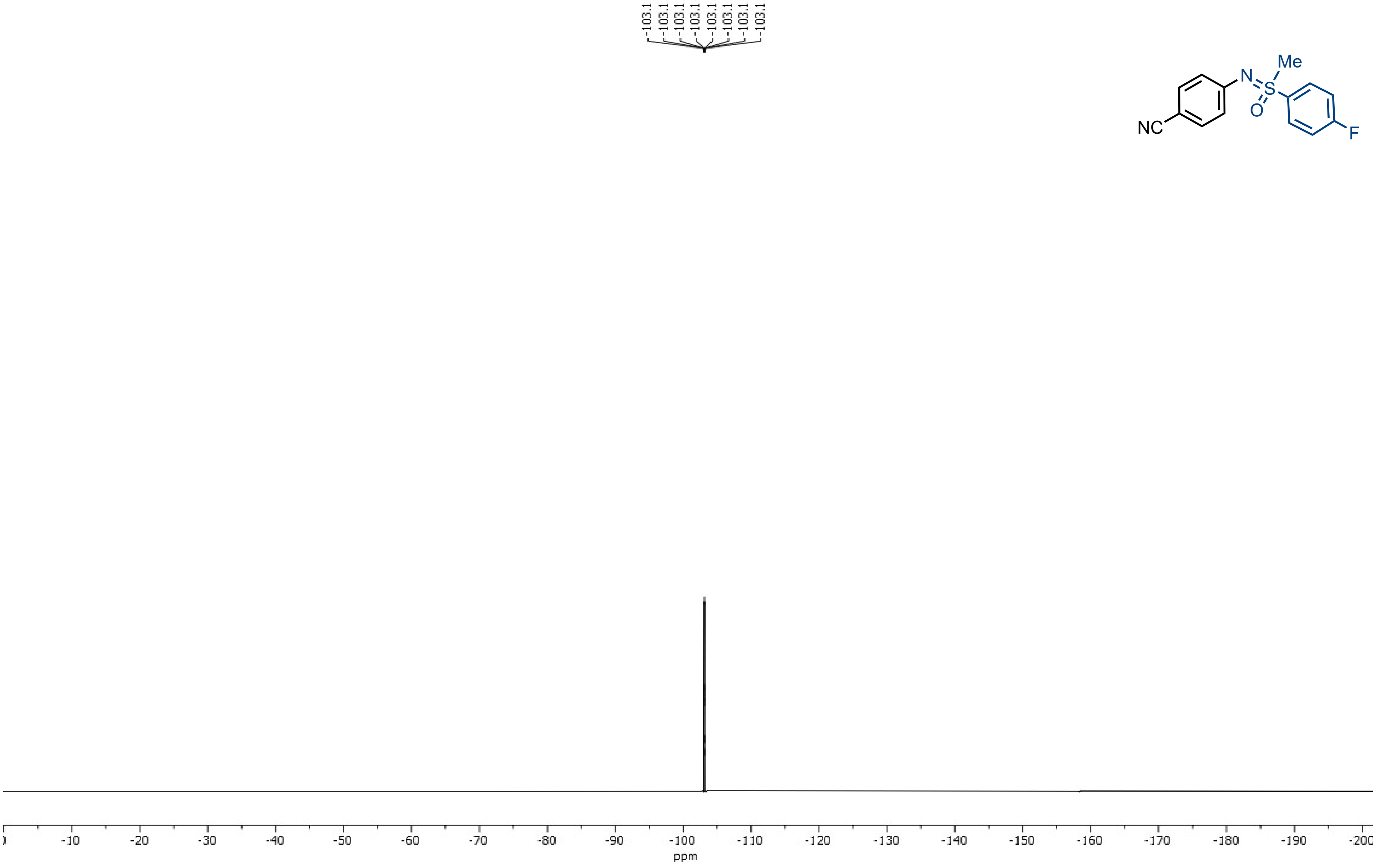

^{1}H NMR of ((2-fluorophenyl)imino)(4-fluorophenyl)(methyl)-λ^{6}-sulfanone (10)

$CDCl_3$, 298 K

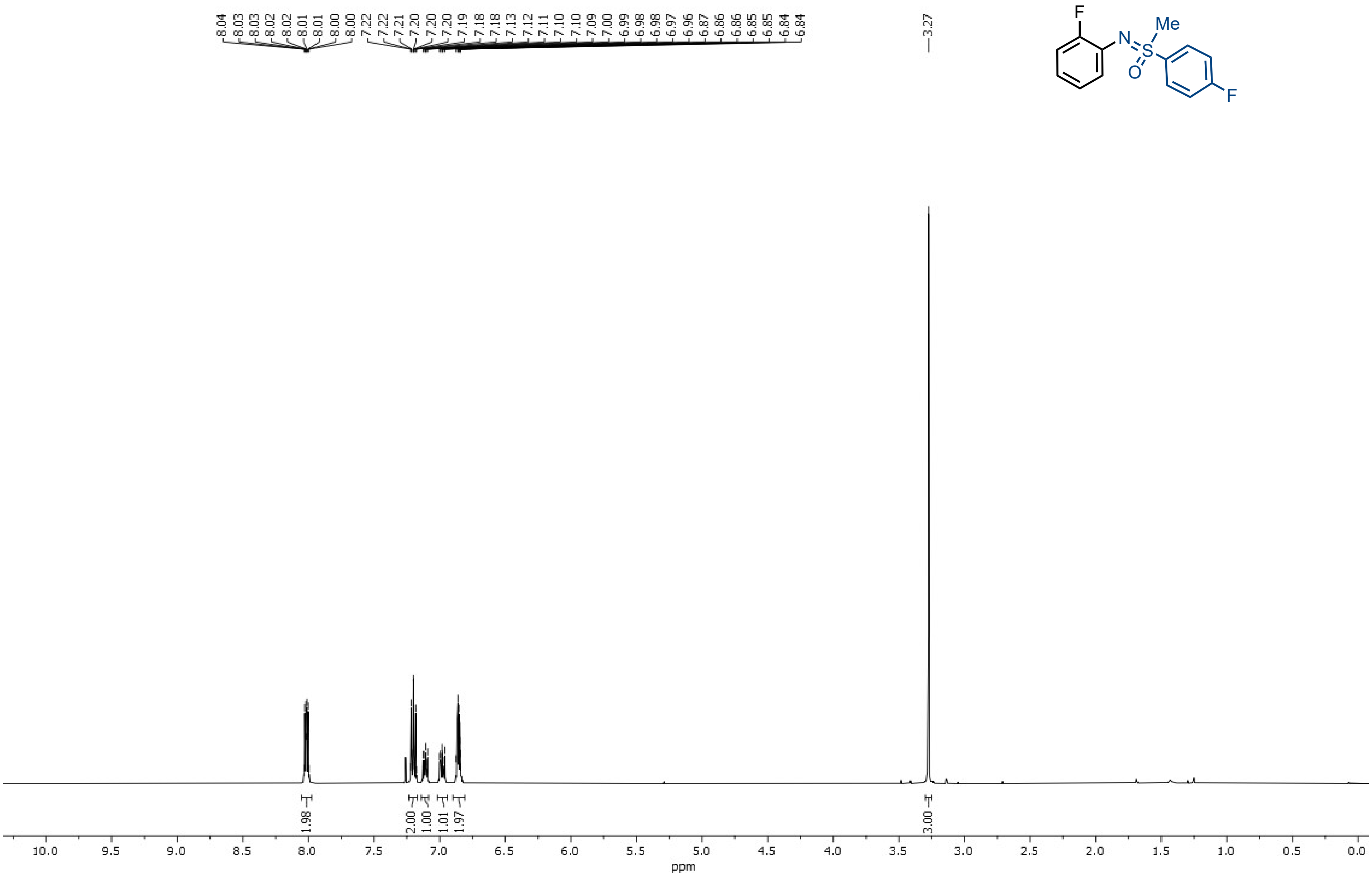

^{13}C NMR of ((2-fluorophenyl)imino)(4-fluorophenyl)(methyl)-λ^6-sulfanone (10)

$CDCl_3$, 298 K

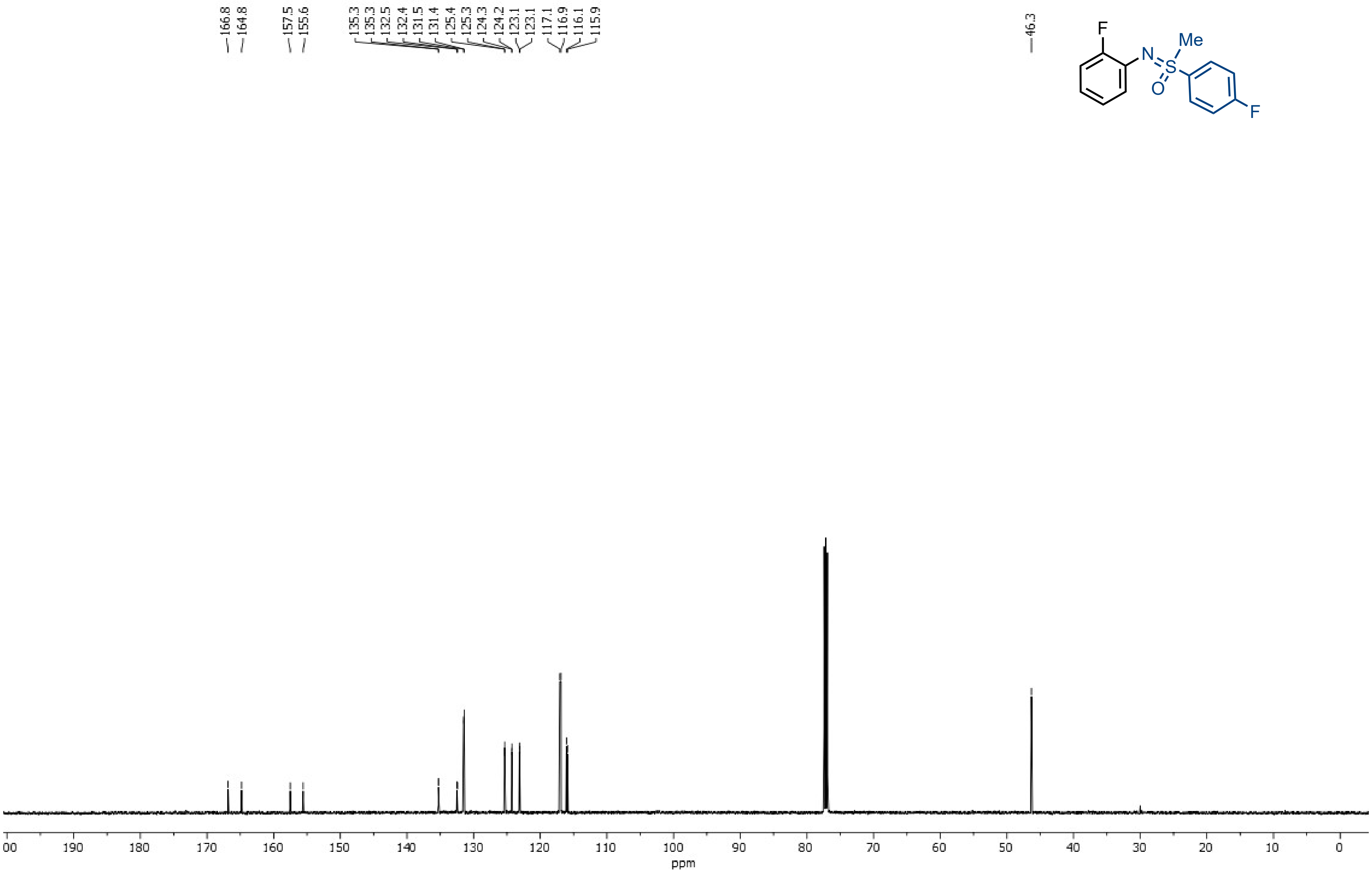

^{19}F NMR of ((2-fluorophenyl)imino)(4-fluorophenyl)(methyl)-λ^{6}-sulfanone (10)

CDCl$_3$, 298 K

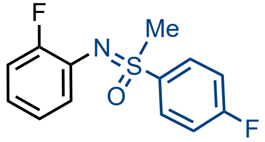

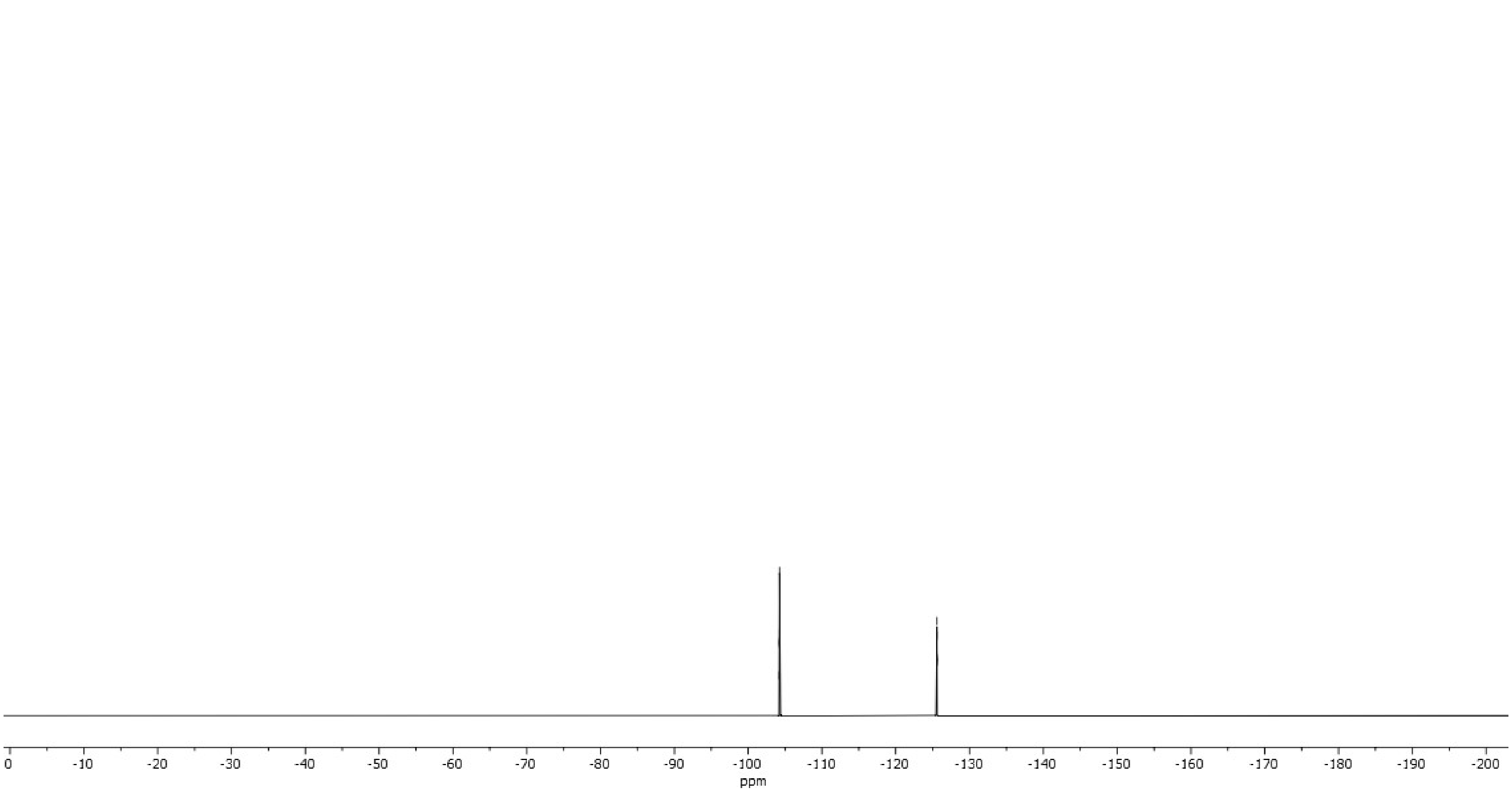

^{1}H NMR of ((3-Methoxyphenyl)imino)(4-fluorophenyl)(methyl)-λ^6-sulfanone (11)

$CDCl_3$, 298 K

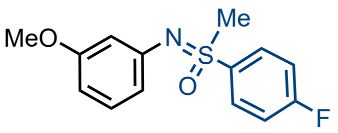

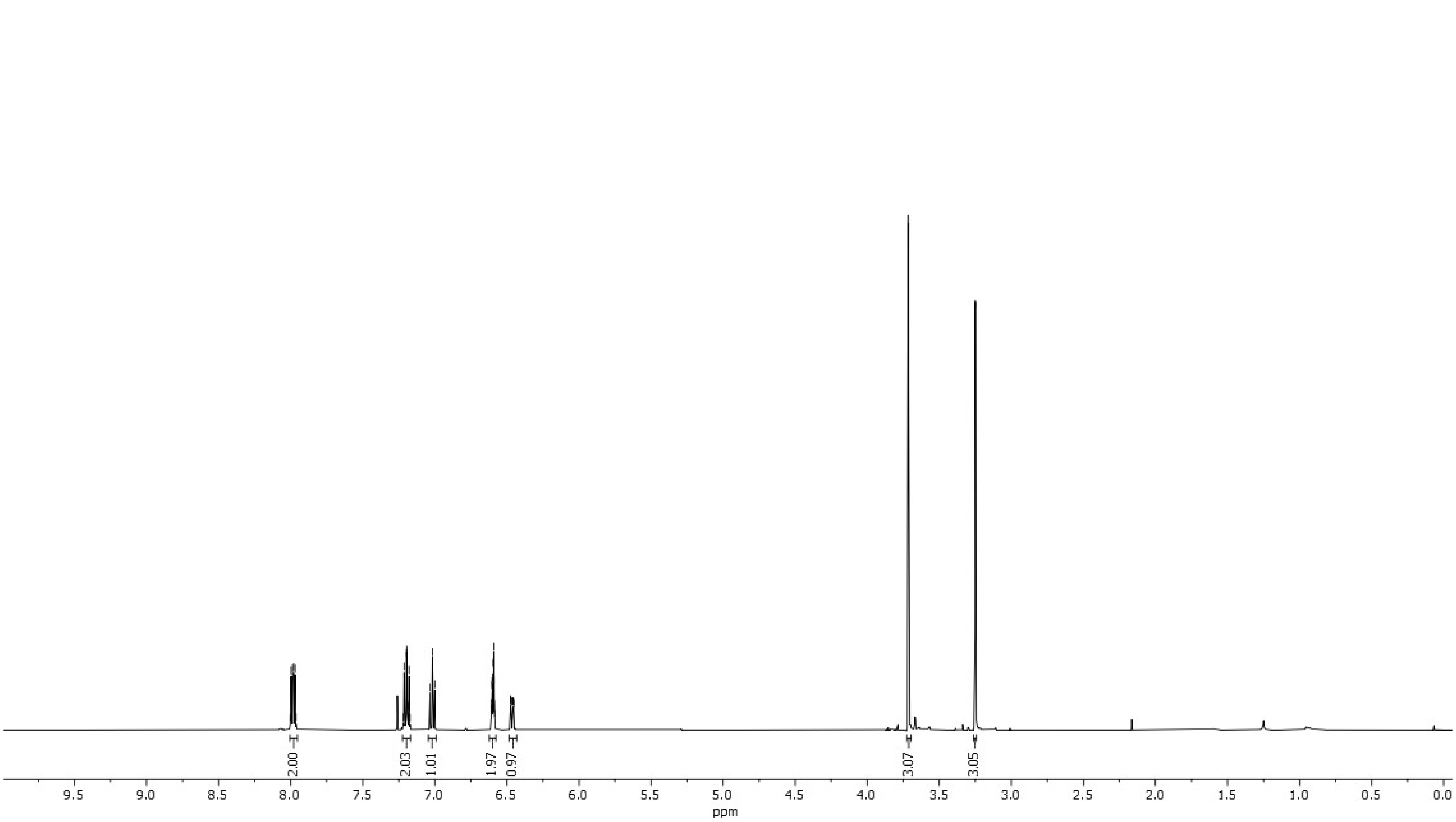

^{13}C NMR of ((3-Methoxyphenyl)imino)(4-fluorophenyl)(methyl)-λ^6-sulfanone (11)

$CDCl_3$, 298 K

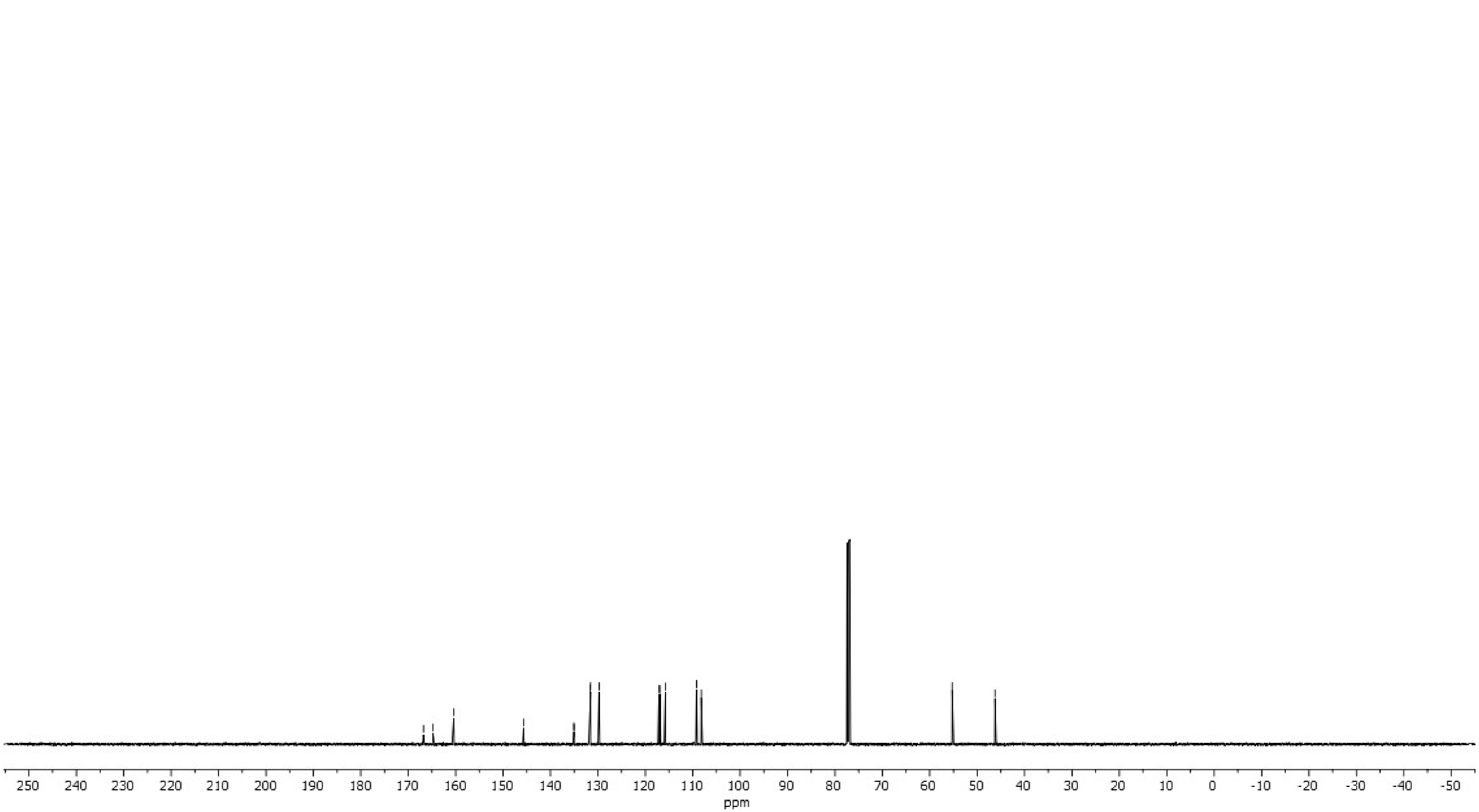

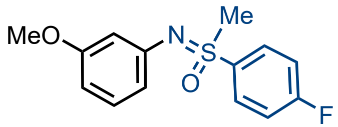

^{19}F NMR of ((3-Methoxyphenyl)imino)(4-fluorophenyl)(methyl)-λ^6-sulfanone (11)

$CDCl_3$, 298 K

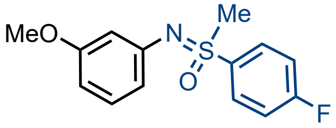

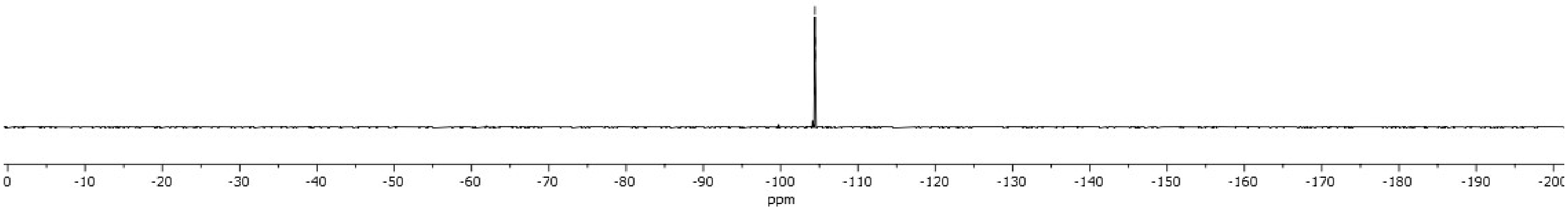

^{1}H NMR of (4-fluorophenyl)(methyl)((2-(trifluoromethyl)pyridin-4-yl)imino)-λ^6-sulfanone (12)

$CDCl_3$, 298 K

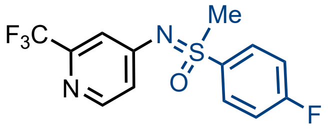

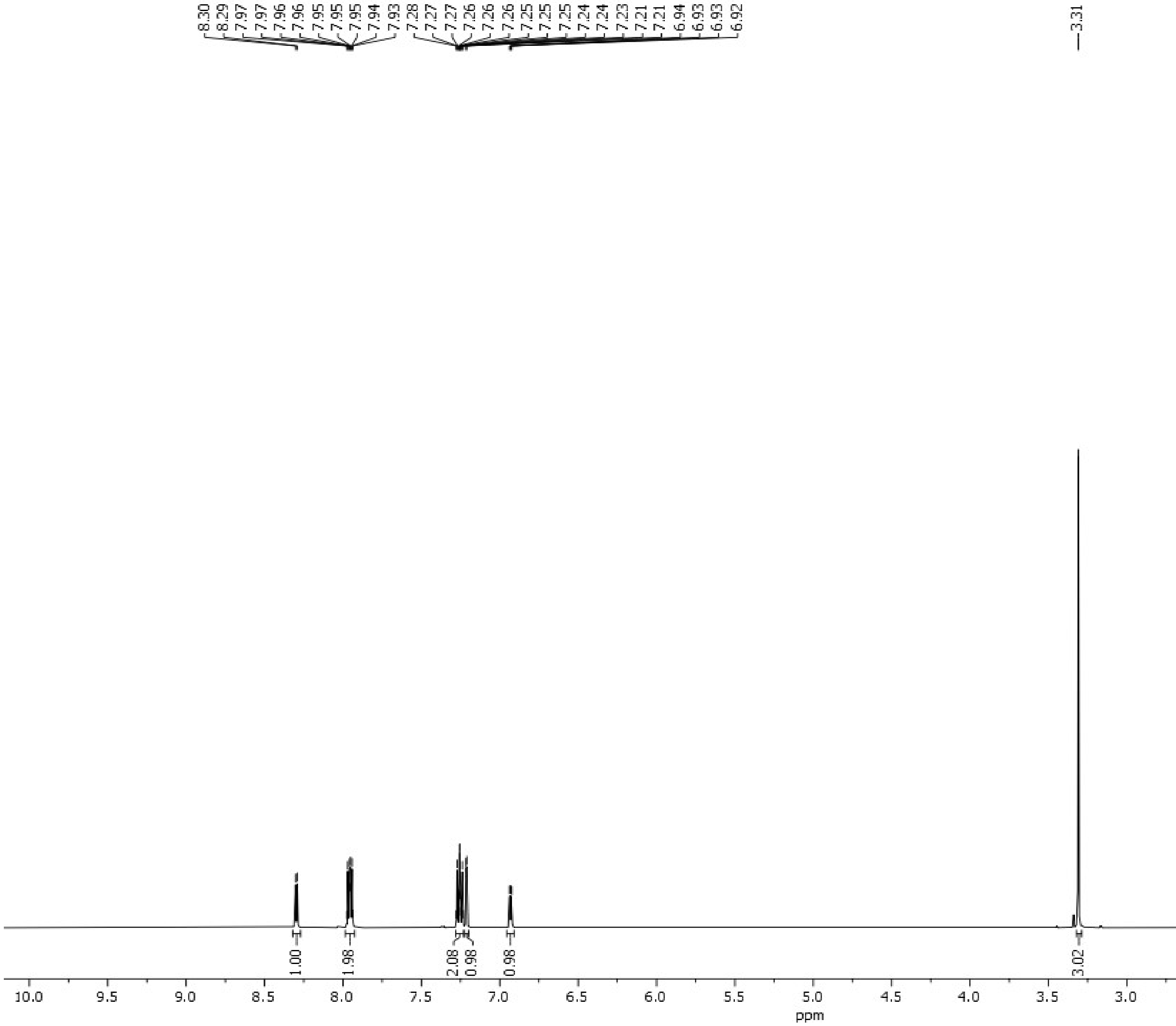

^{13}C NMR of (4-fluorophenyl)(methyl)((2-(trifluoromethyl)pyridin-4-yl)imino)-λ^6-sulfanone (12)

$CDCl_3$, 298 K

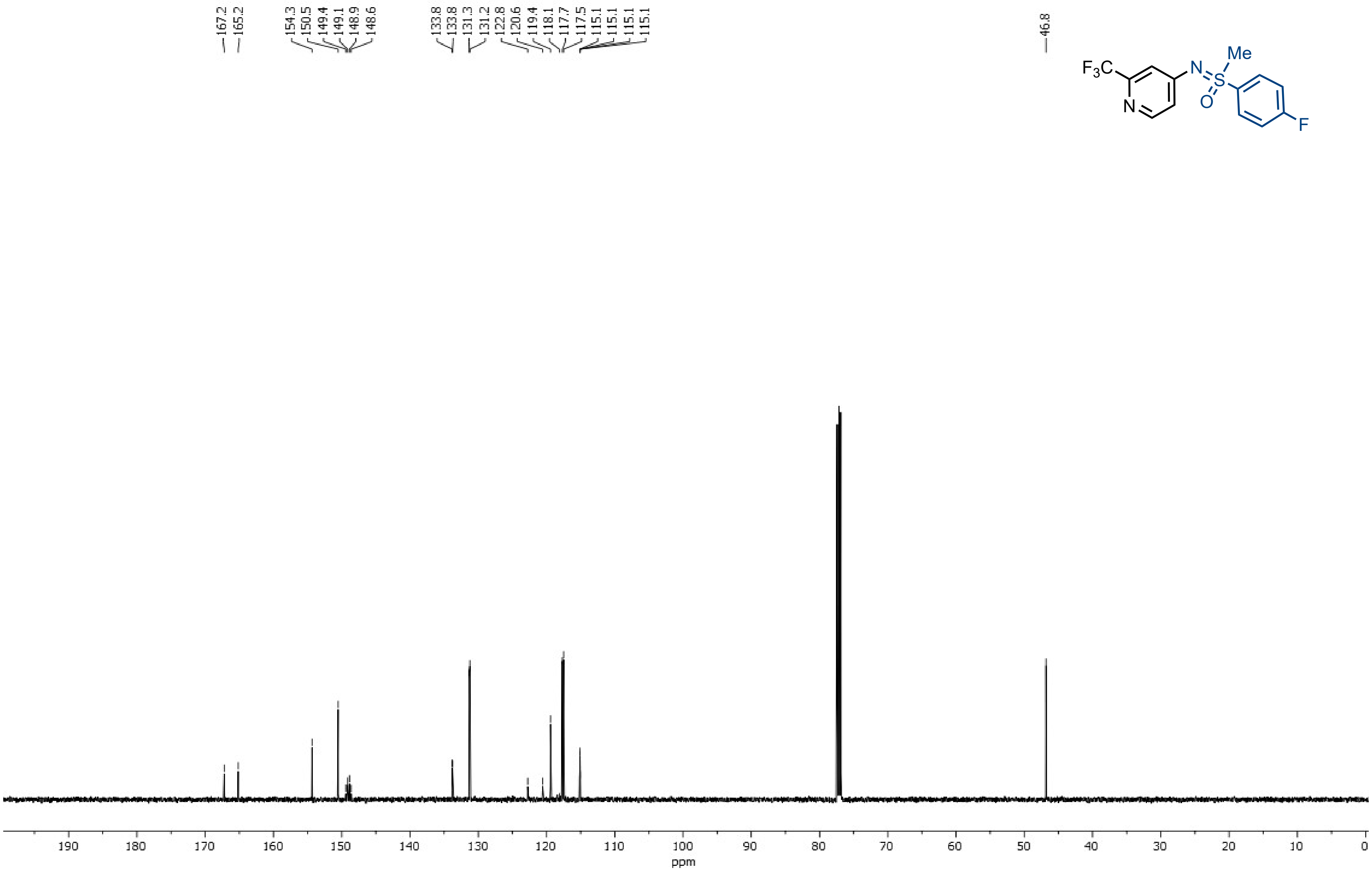

^{19}F NMR of (4-fluorophenyl)(methyl)((2-(trifluoromethyl)pyridin-4-yl)imino)-λ^6-sulfanone (12)

$CDCl_3$, 298 K

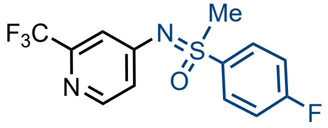

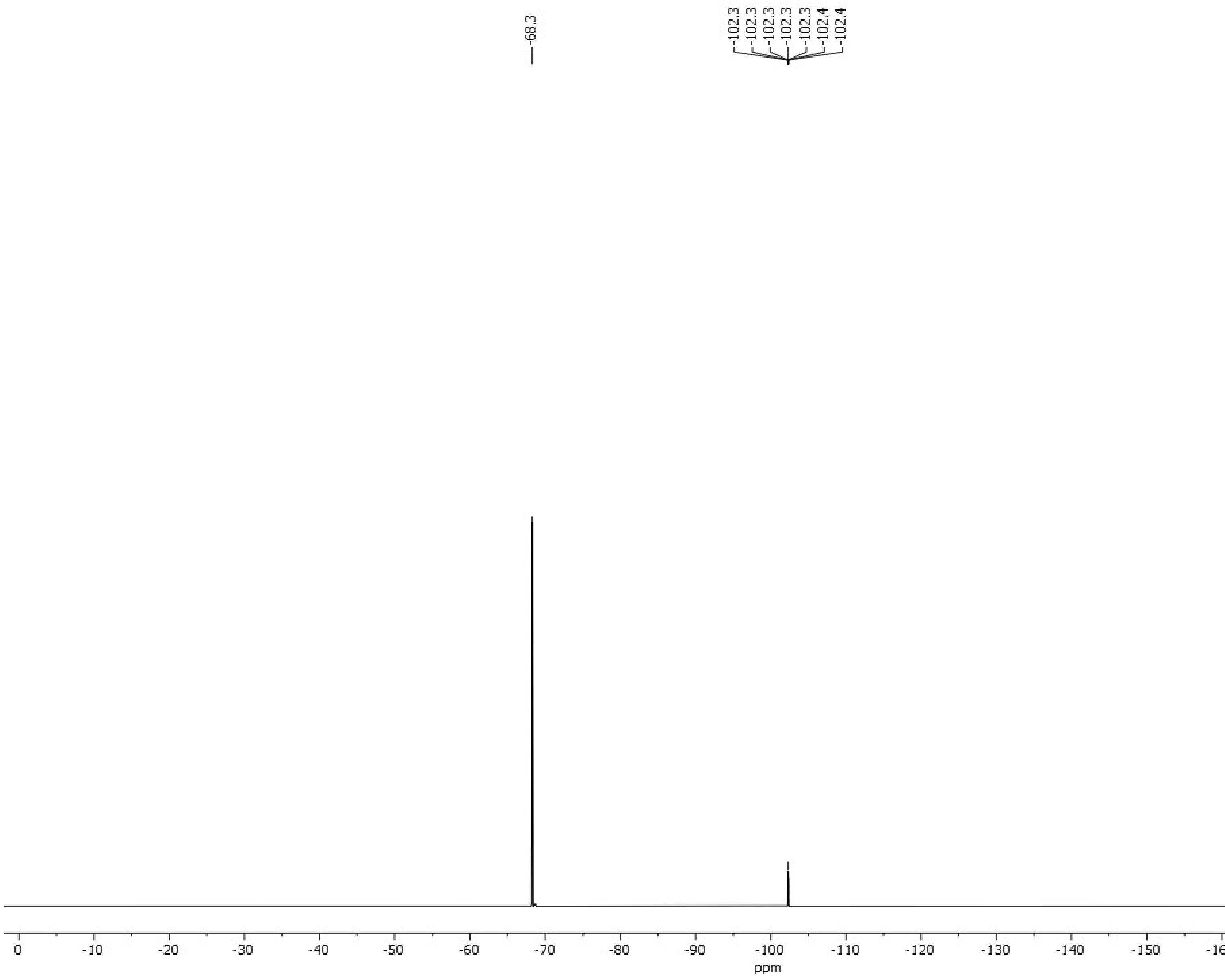

^{1}H NMR of ((4-acetylphenyl)imino)(4-fluorophenyl)(methyl)-λ^6-sulfanone (13)

$CDCl_3$, 298 K

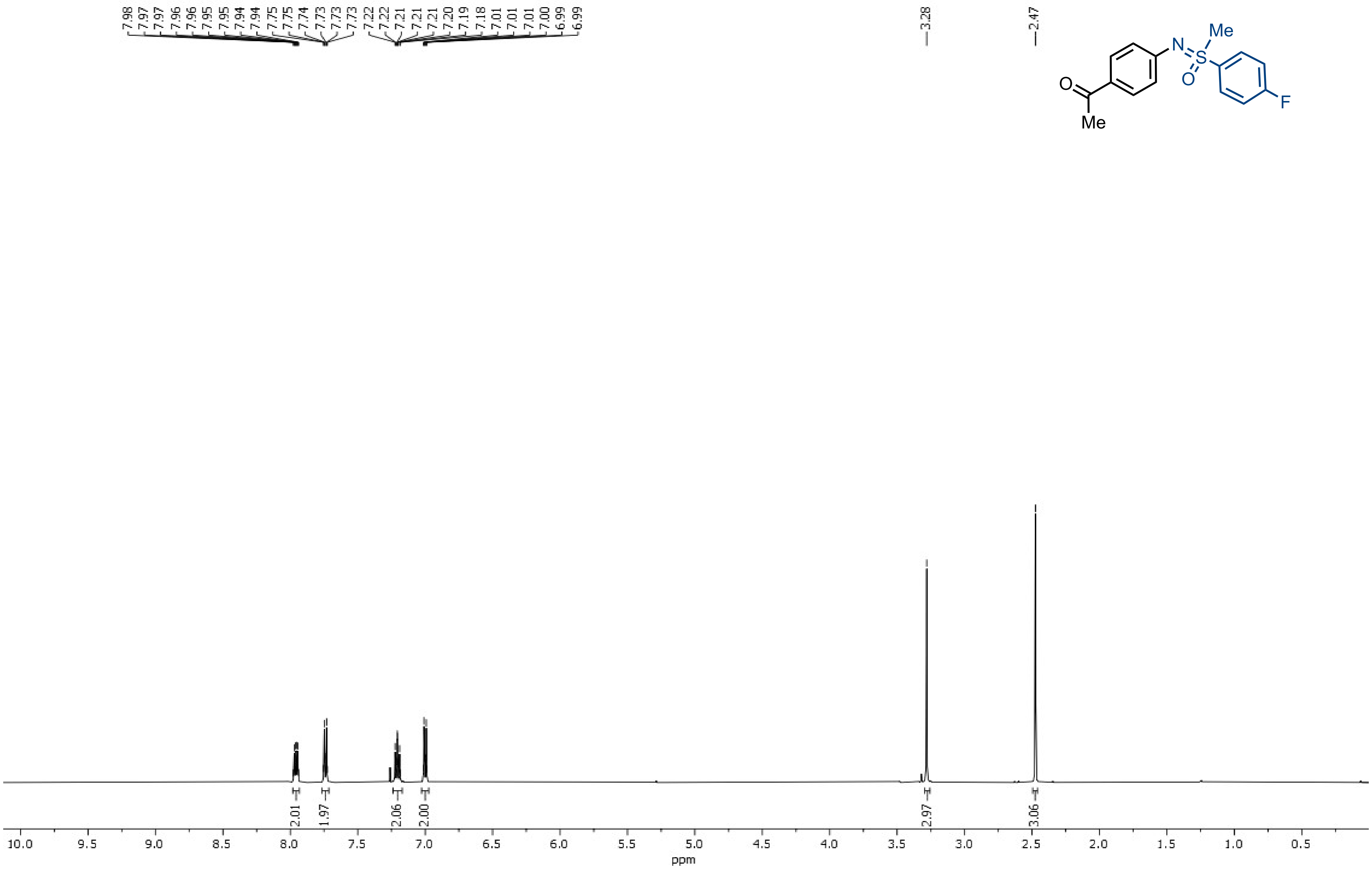

^{13}C NMR of ((4-acetylphenyl)imino)(4-fluorophenyl)(methyl)-λ^6-sulfanone (13)

$CDCl_3$, 298 K

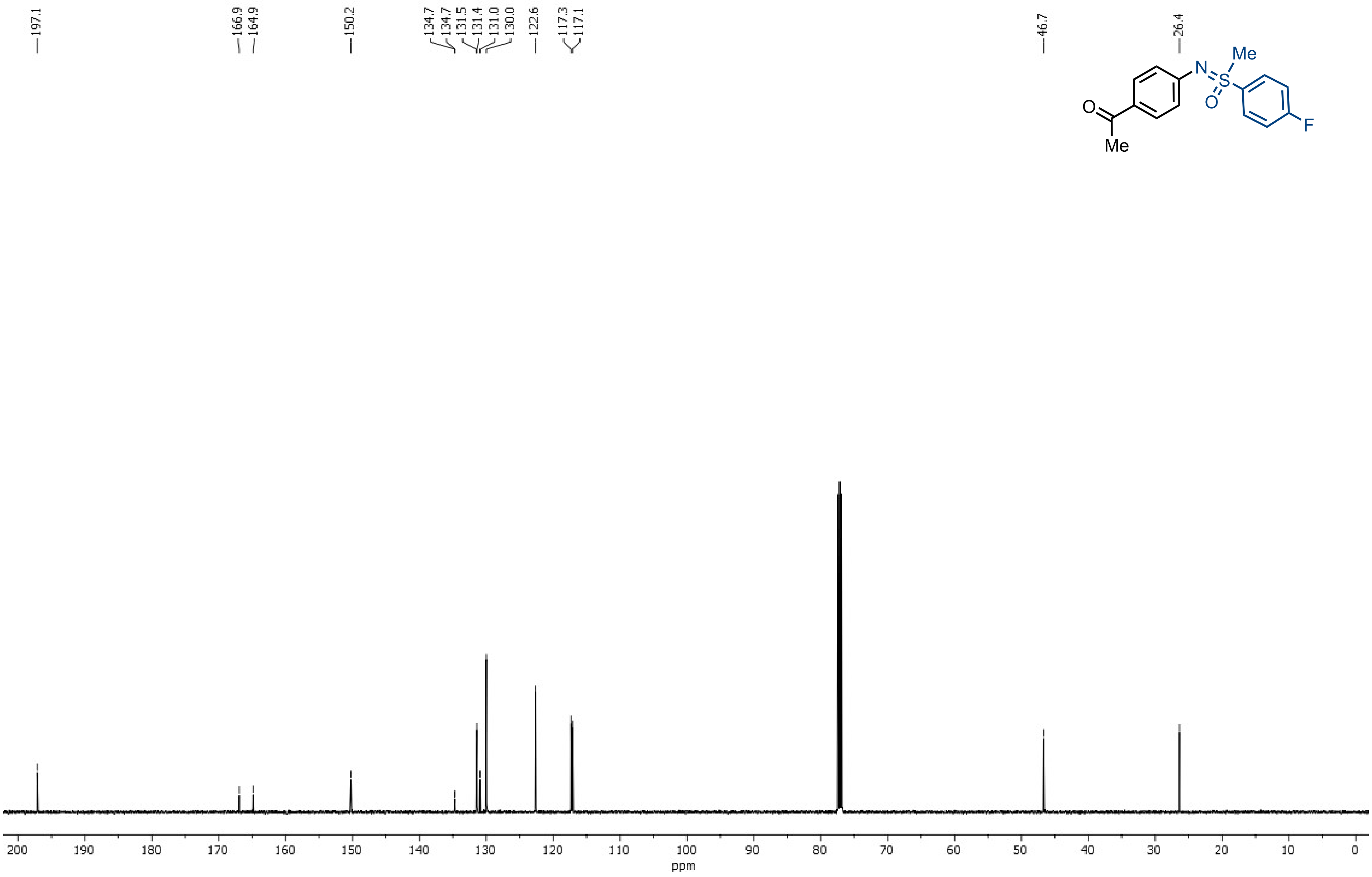

^{19}F NMR of ((4-acetylphenyl)imino)(4-fluorophenyl)(methyl)-λ^6-sulfanone (13)

$CDCl_3$, 298 K

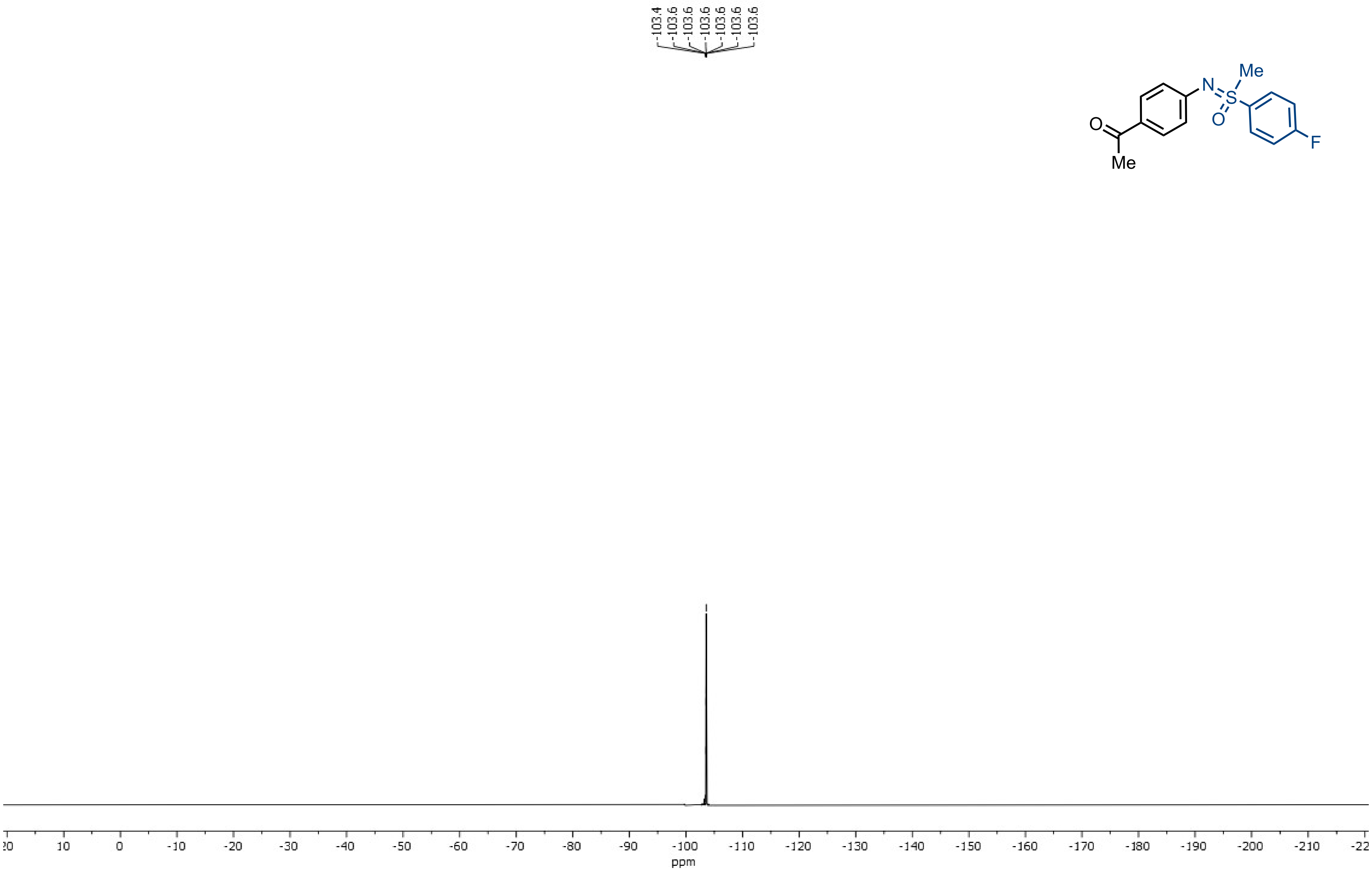

^{1}H NMR of (+)-menthol derivative 14

$CDCl_3$, 298 K

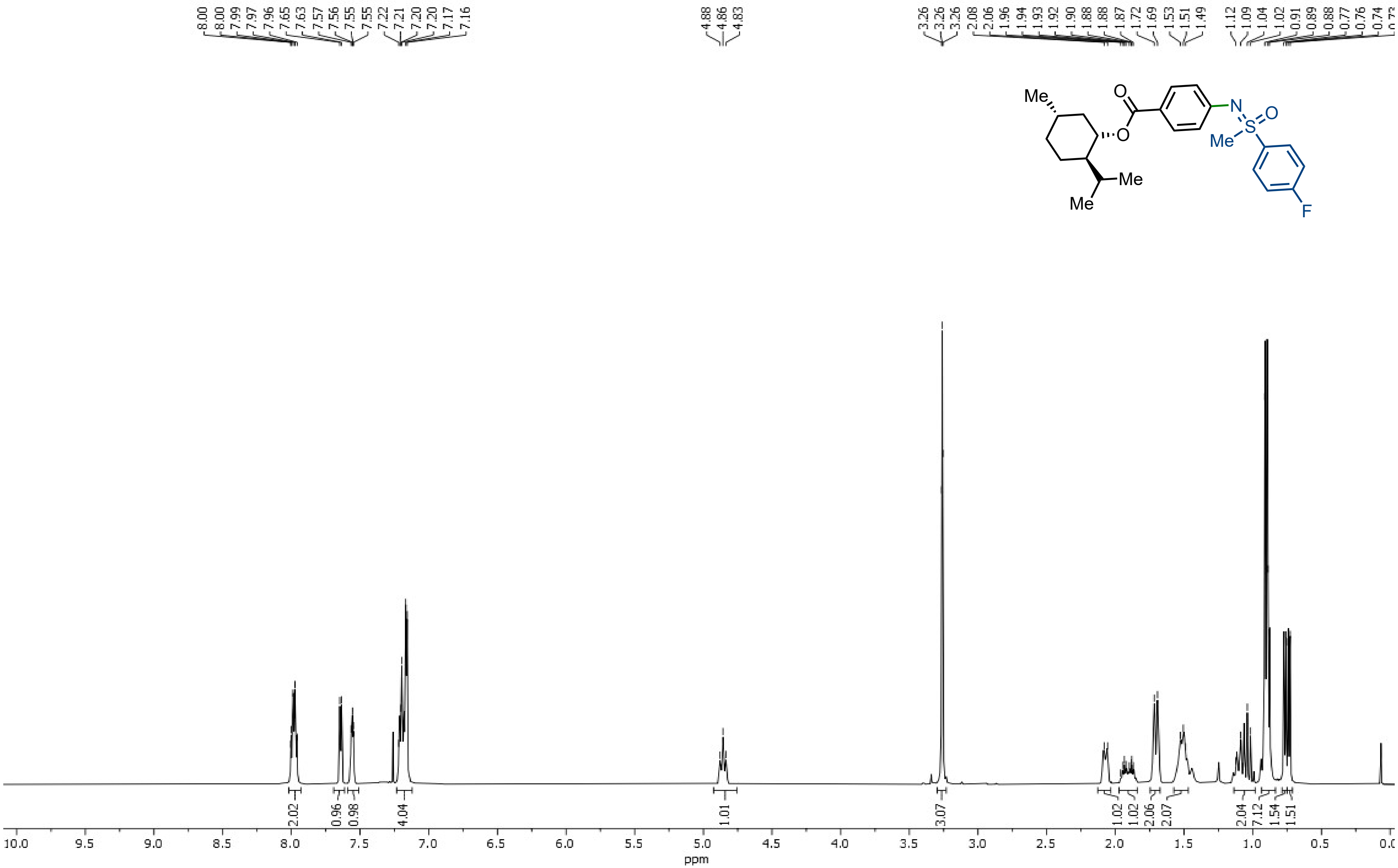

^{13}C NMR of (+)-menthol derivative 14

CDCl$_3$, 298 K

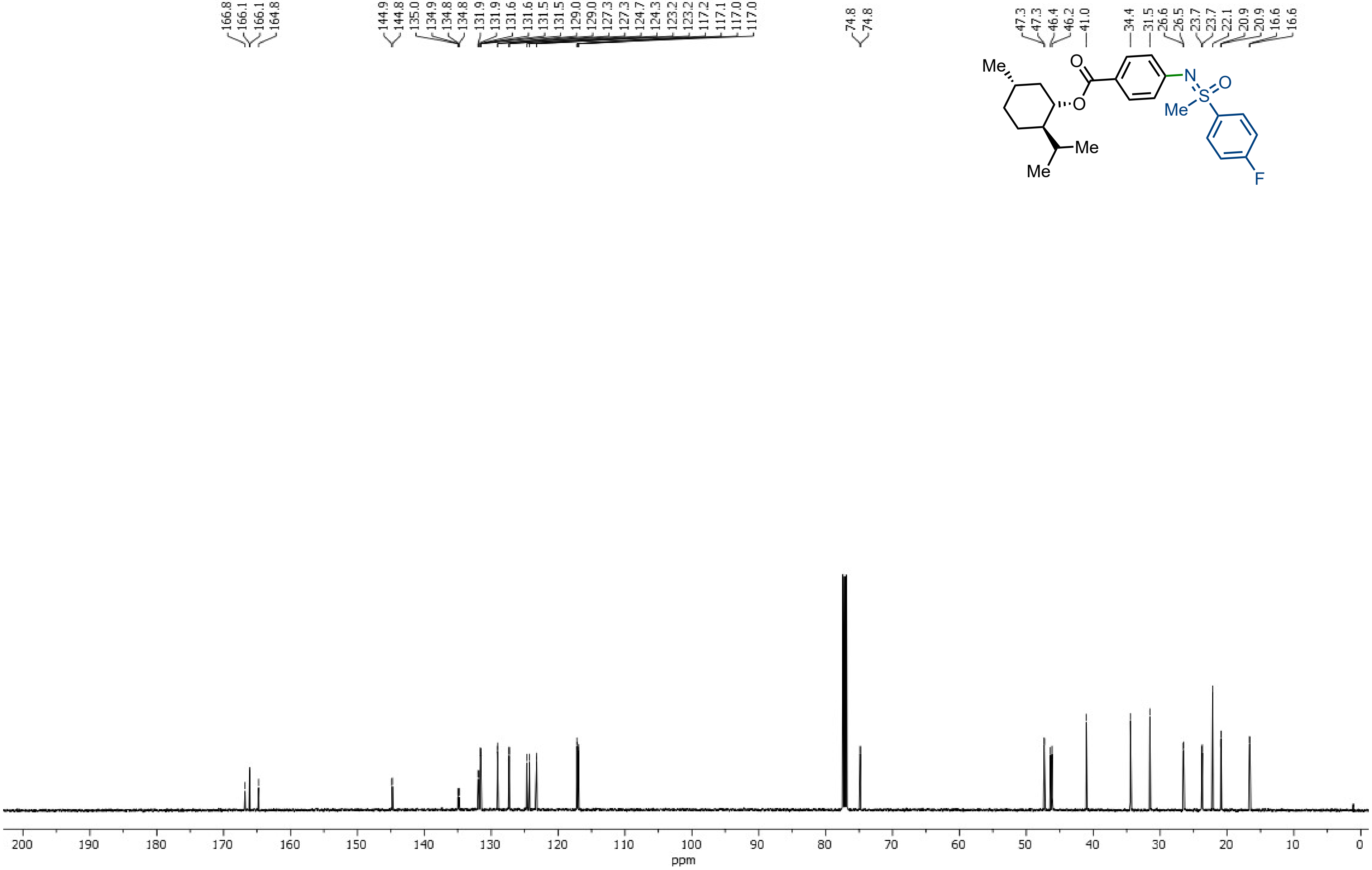

^{19}F NMR of (+)-menthol derivative 14

$CDCl_3$, 298 K

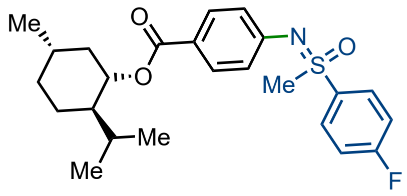

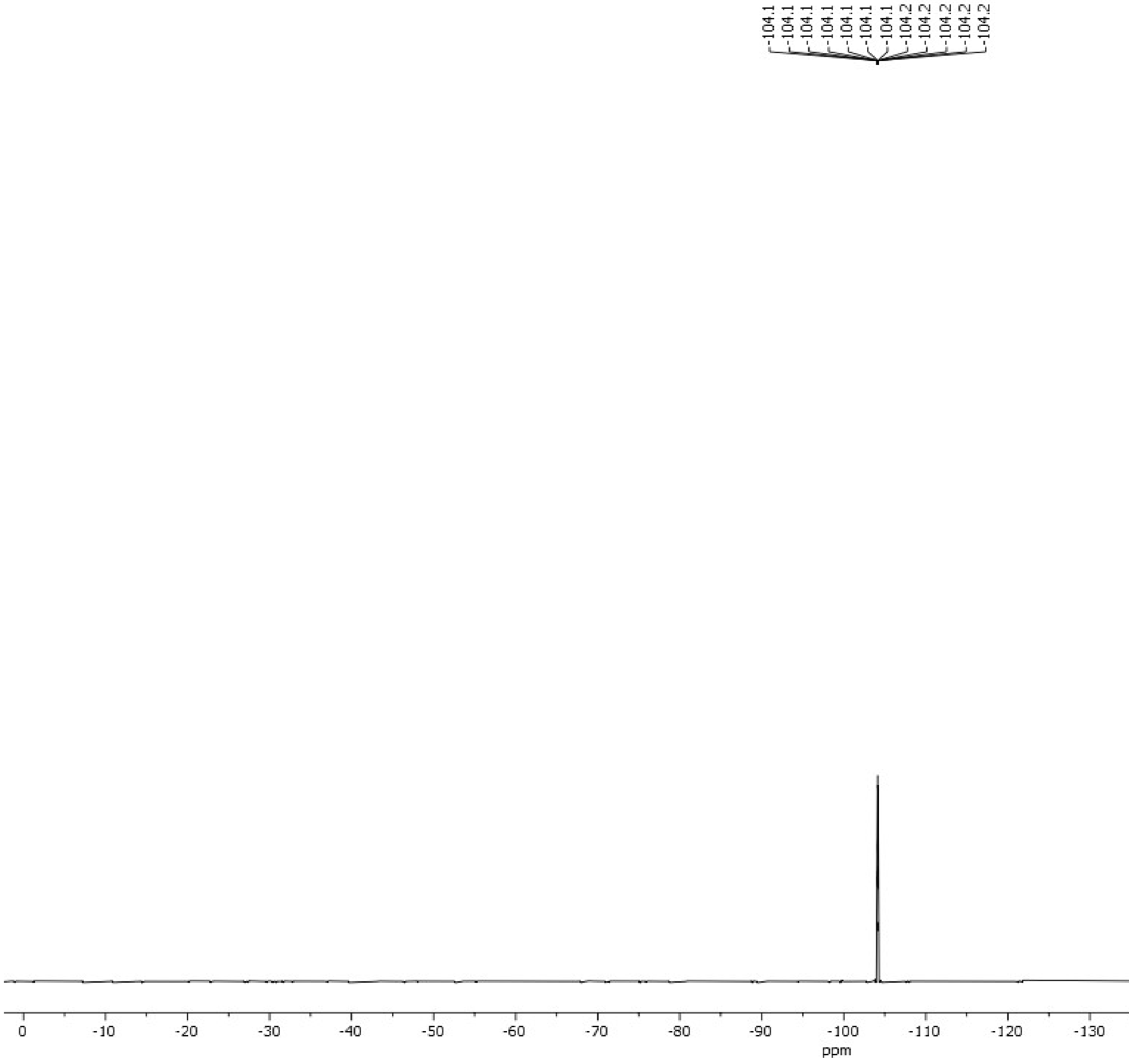

^{1}H NMR of ((3-methylphenyl)imino)(4-fluorophenyl)(methyl)-λ^6-sulfanone (15)

$CDCl_3$, 298 K

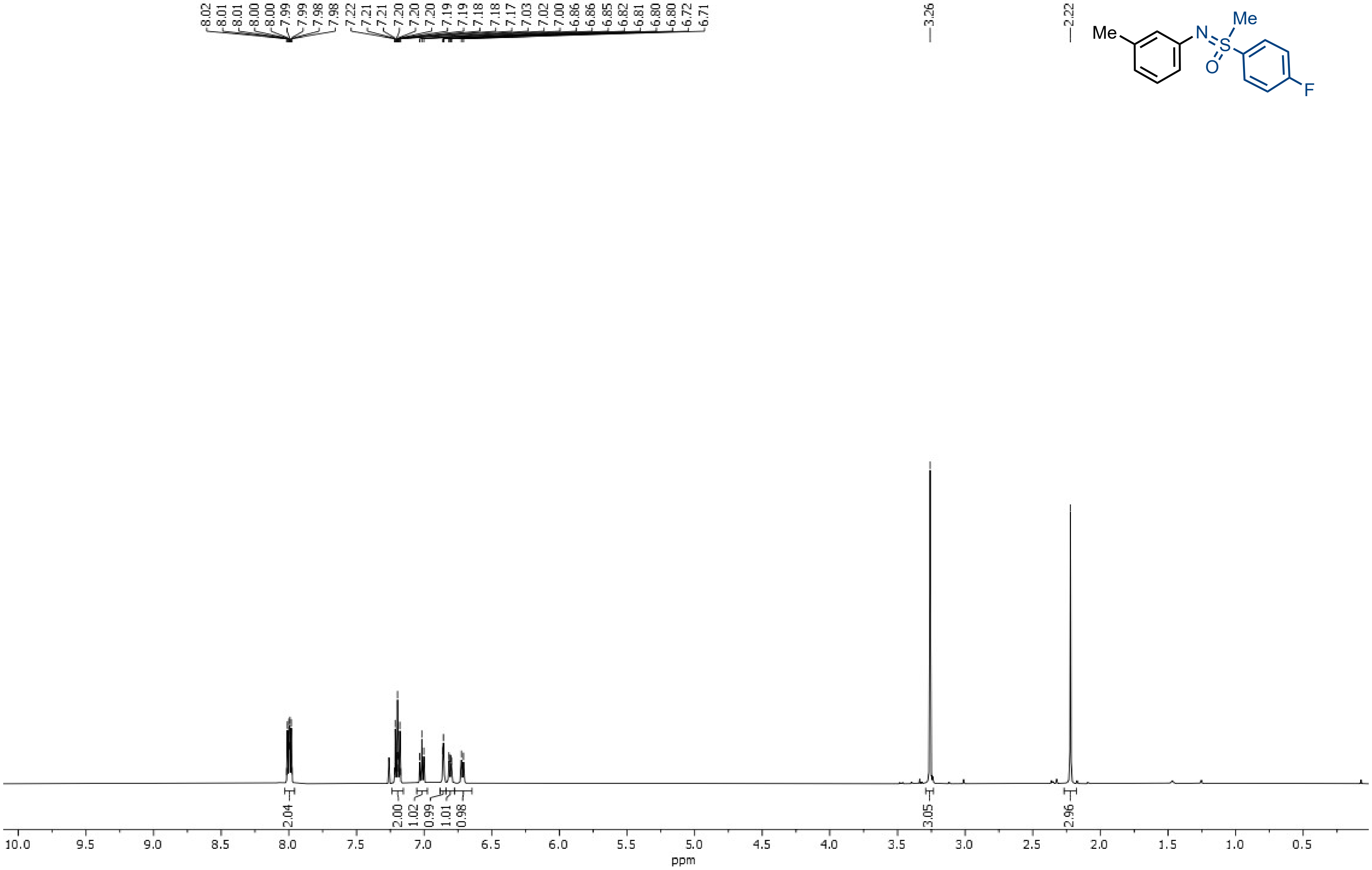

^{13}C NMR of ((3-methylphenyl)imino)(4-fluorophenyl)(methyl)-λ^6-sulfanone (15)

$CDCl_3$, 298 K

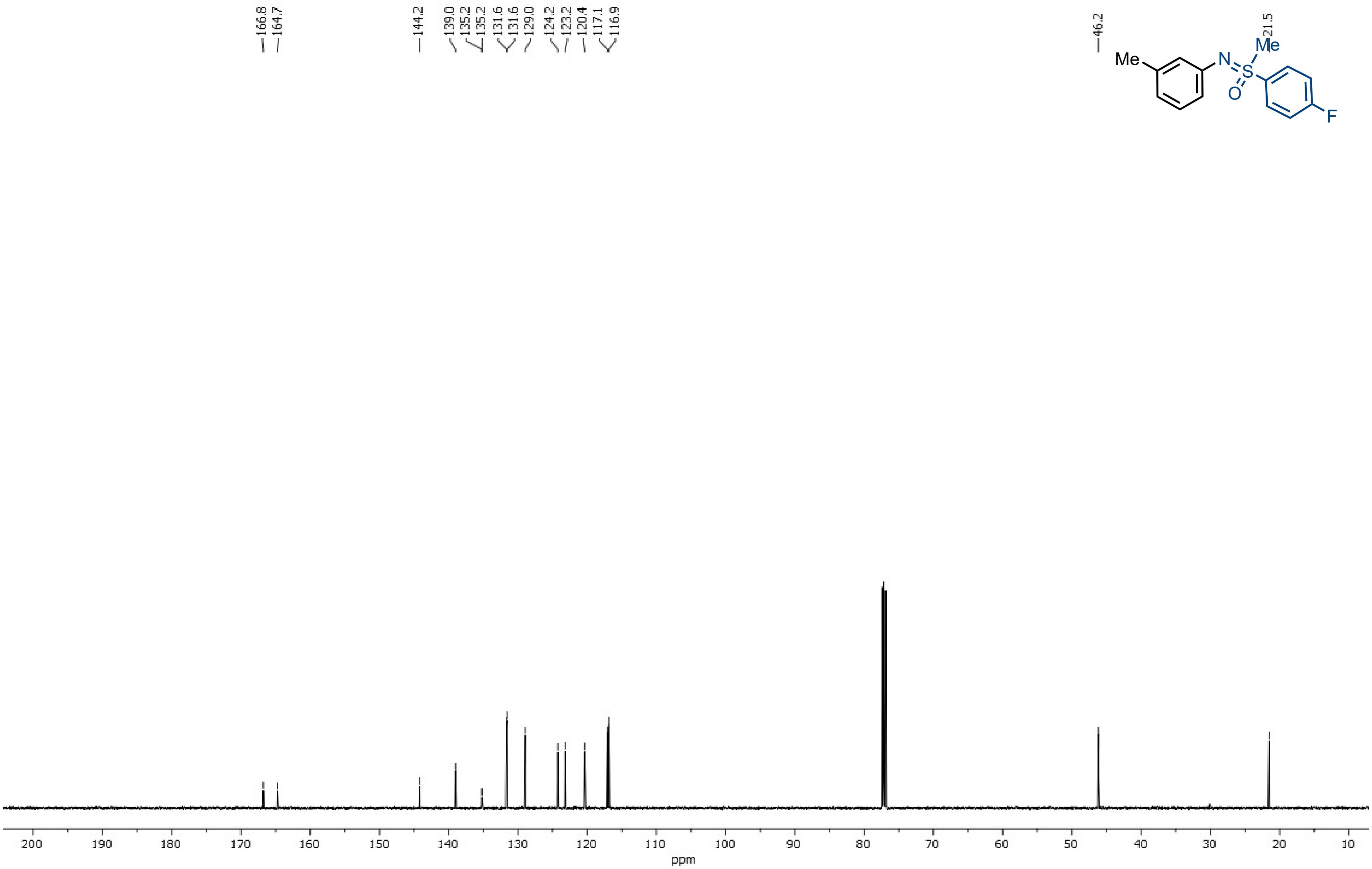

^{19}F NMR of ((3-methylphenyl)imino)(4-fluorophenyl)(methyl)-λ^{6}-sulfanone (15)

$CDCl_3$, 298 K

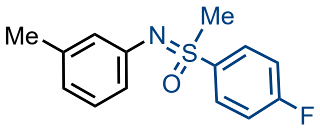

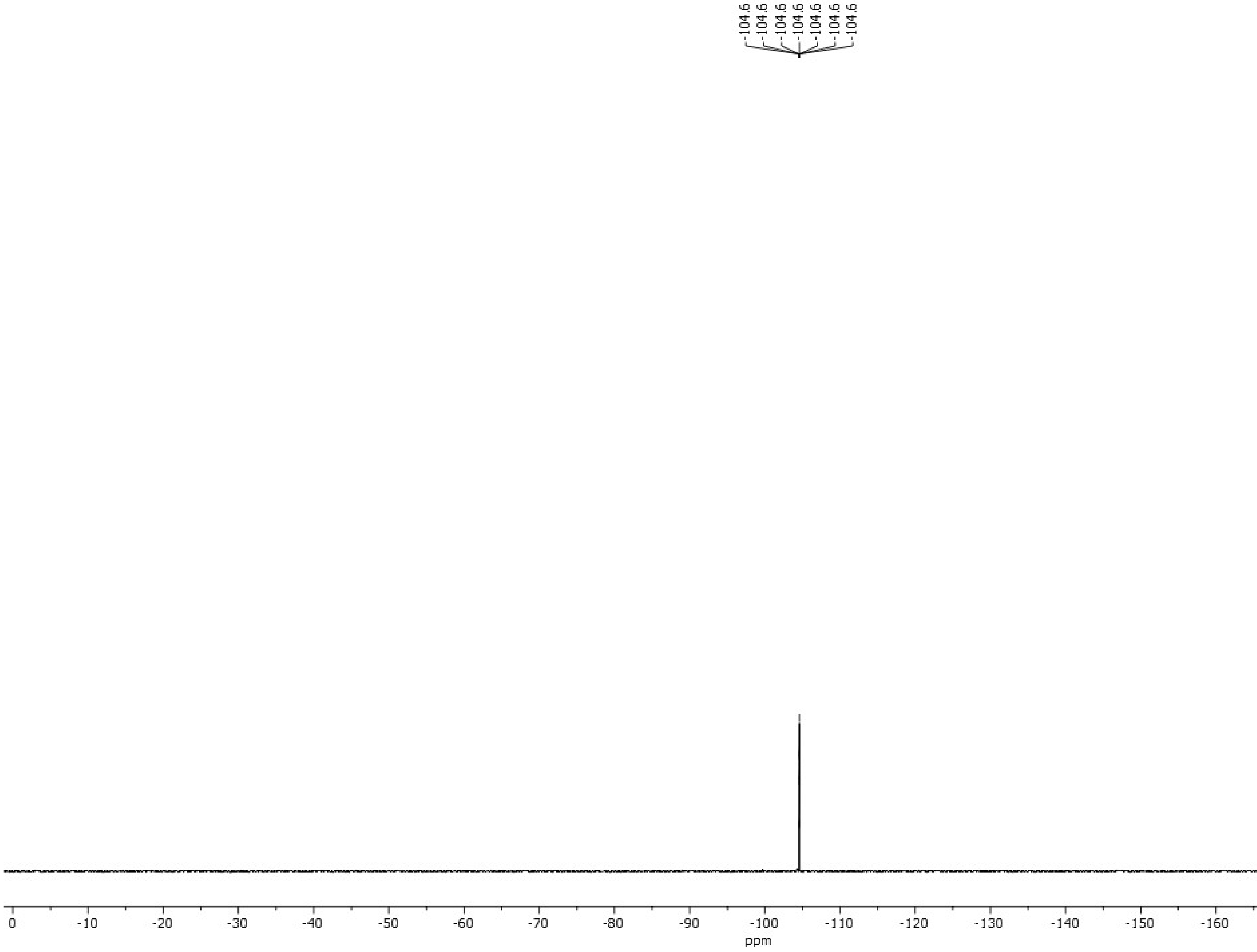

^{1}H NMR of ((3,5-Dimethylphenyl)imino)(4-fluorophenyl)(methyl)-λ^6-sulfanone (16)

$CDCl_3$, 298 K

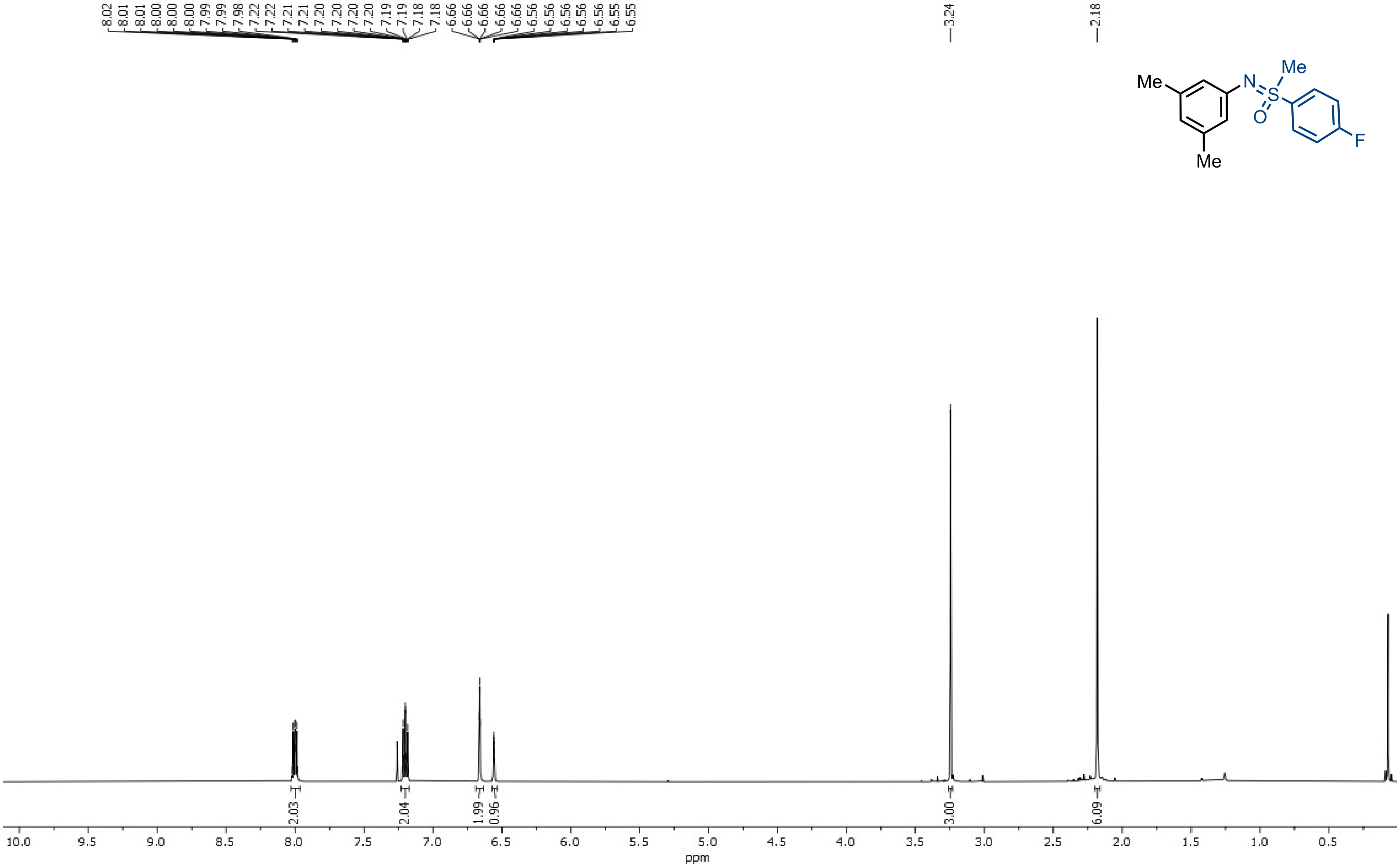

^{13}C NMR of ((3,5-Dimethylphenyl)imino)(4-fluorophenyl)(methyl)-λ^6-sulfanone (16)

$CDCl_3$, 298 K

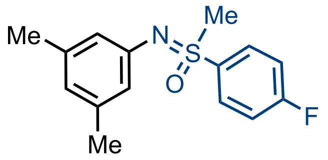

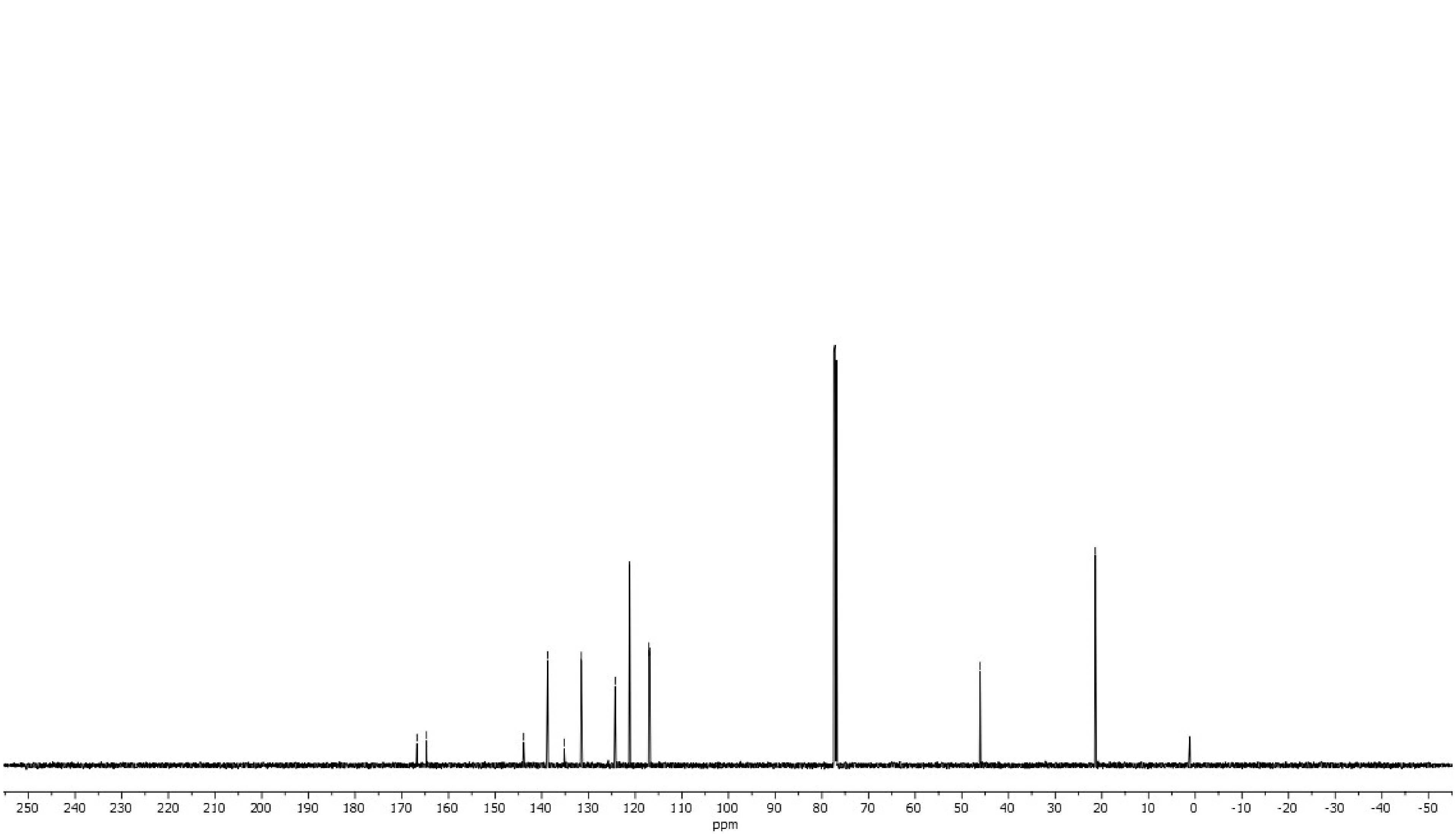

^{19}F NMR of ((3,5-Dimethylphenyl)imino)(4-fluorophenyl)(methyl)-λ^{6}-sulfanone (16)

$CDCl_3$, 298 K

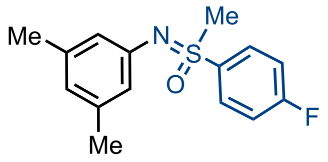

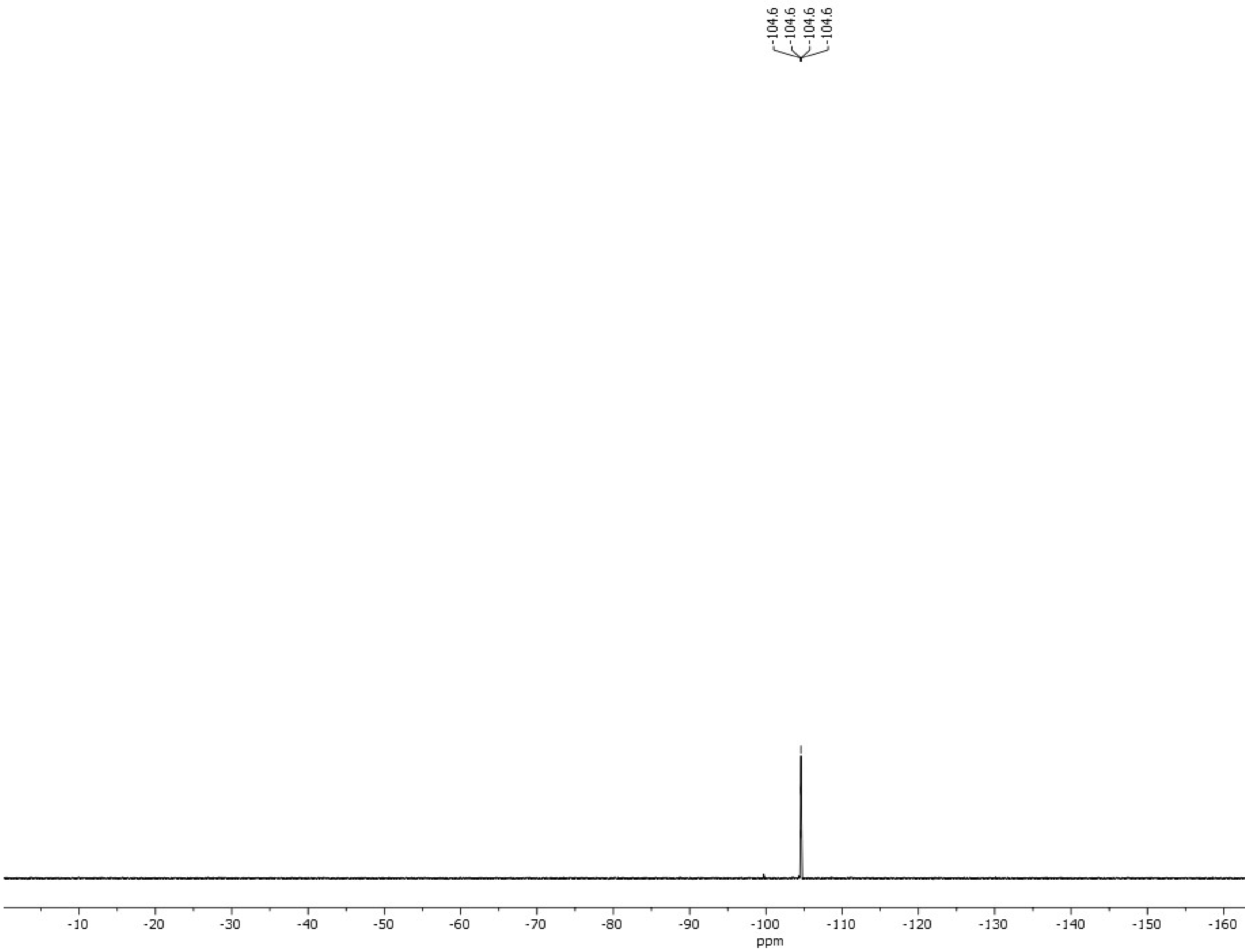

^{1}H NMR of (4-fluorophenyl)(methyl)((4-methyl-3-(morpholinosulfonyl)phenyl)imino)-λ^6-sulfanone (17)

CDCl3, 298 K

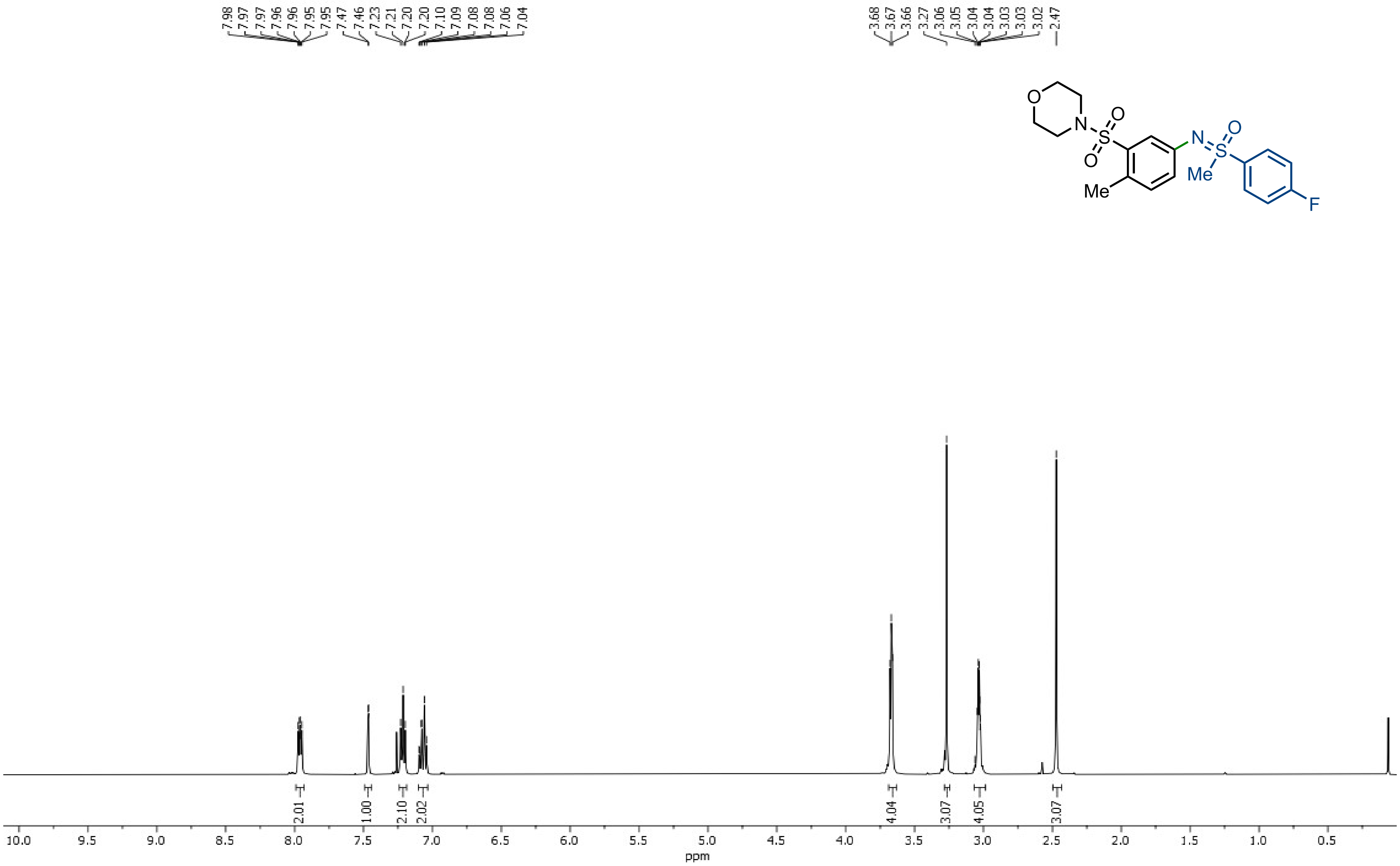

^{13}C NMR of (4-fluorophenyl)(methyl)((4-methyl-3-(morpholinosulfonyl)phenyl)imino)-λ^6-sulfanone (17)

$CDCl_3$, 298 K

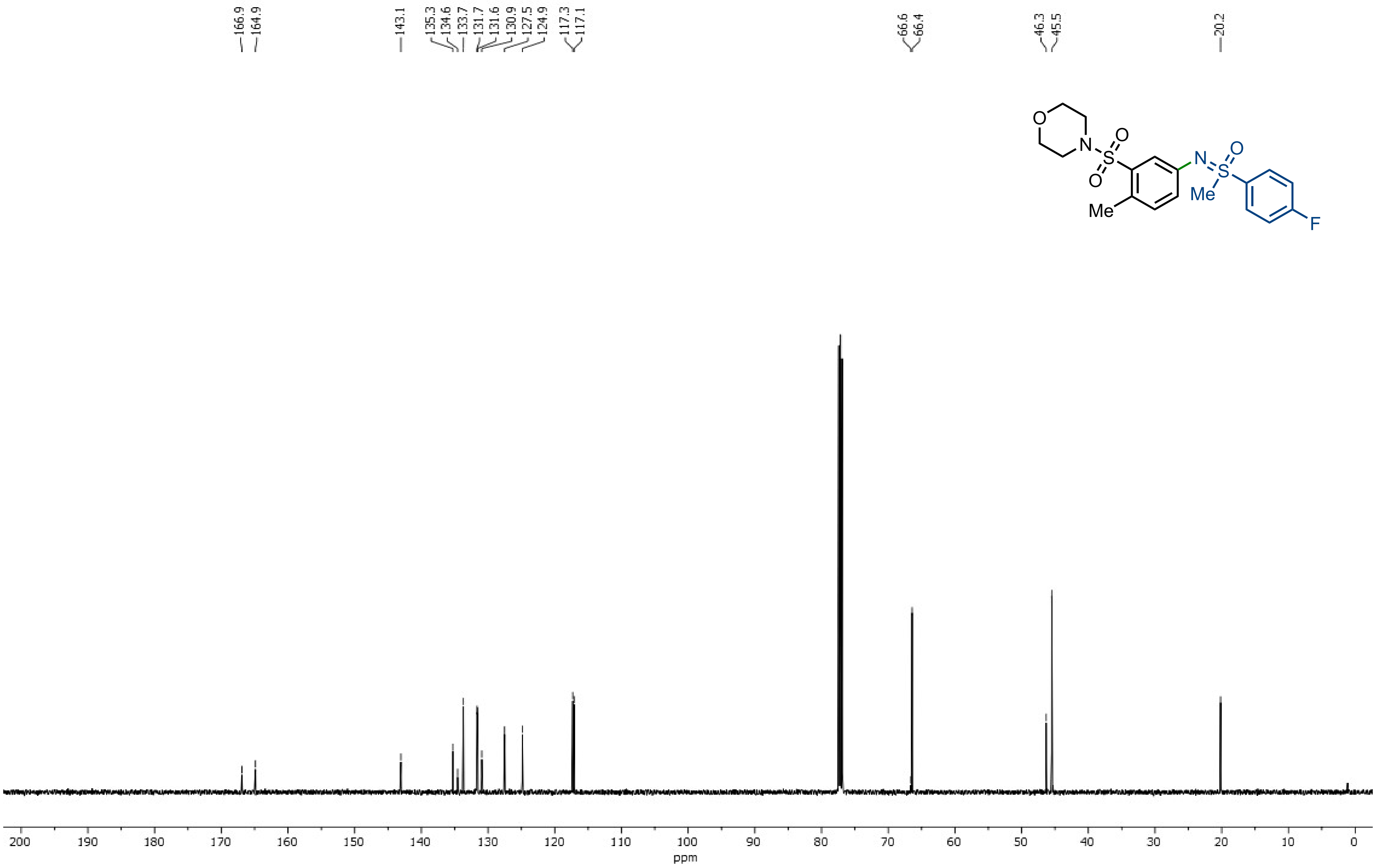

^{19}F NMR of (4-fluorophenyl)(methyl)((4-methyl-3-(morpholinosulfonyl)phenyl)imino)-λ^6-sulfanone (17)

$CDCl_3$, 298 K

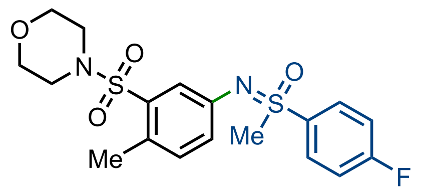

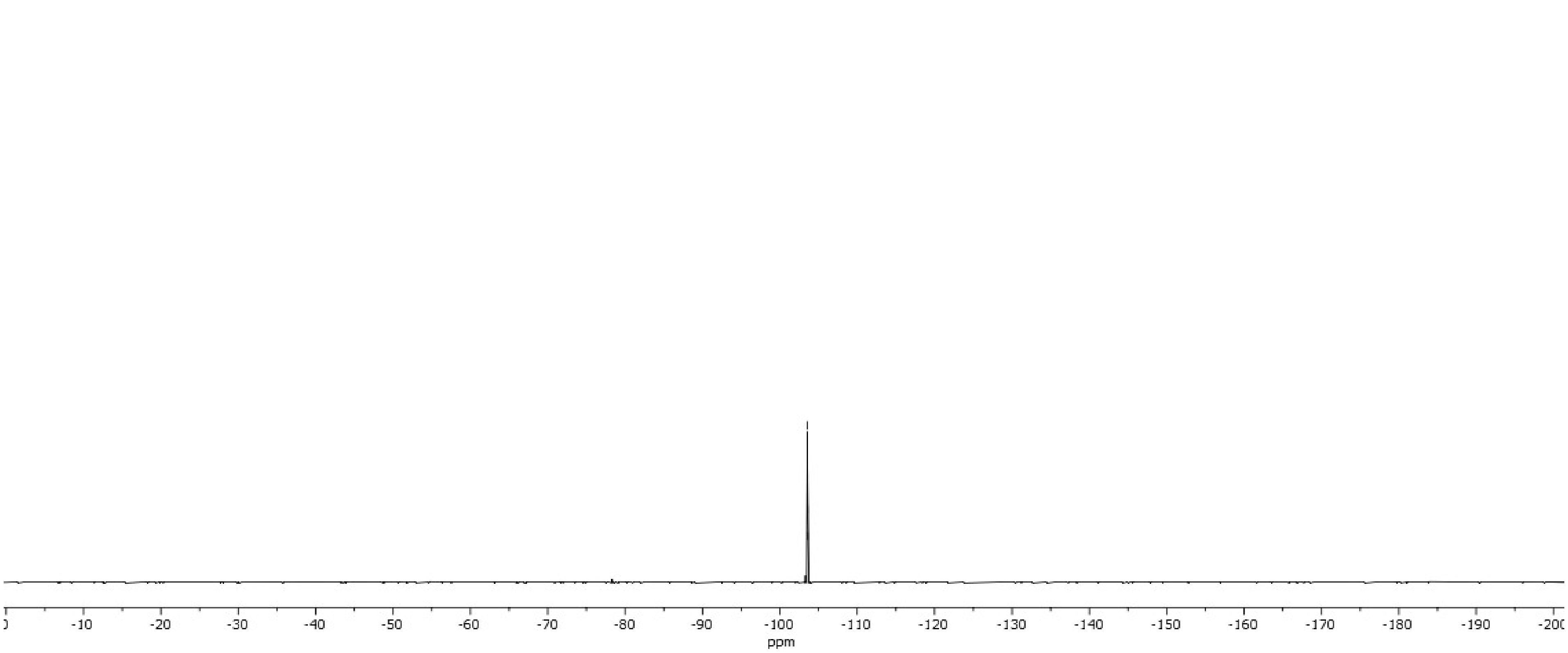

^{1}H NMR of 2,2'-(5-(((4-fluorophenyl)(methyl)(oxo)-λ[6]-sulfanylidene)amino)-1,3-phenylene)bis(2-methylpropanenitrile) (18)

CDCl3, 298 K

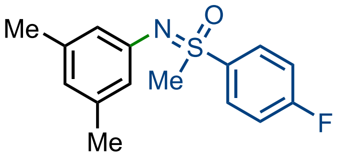

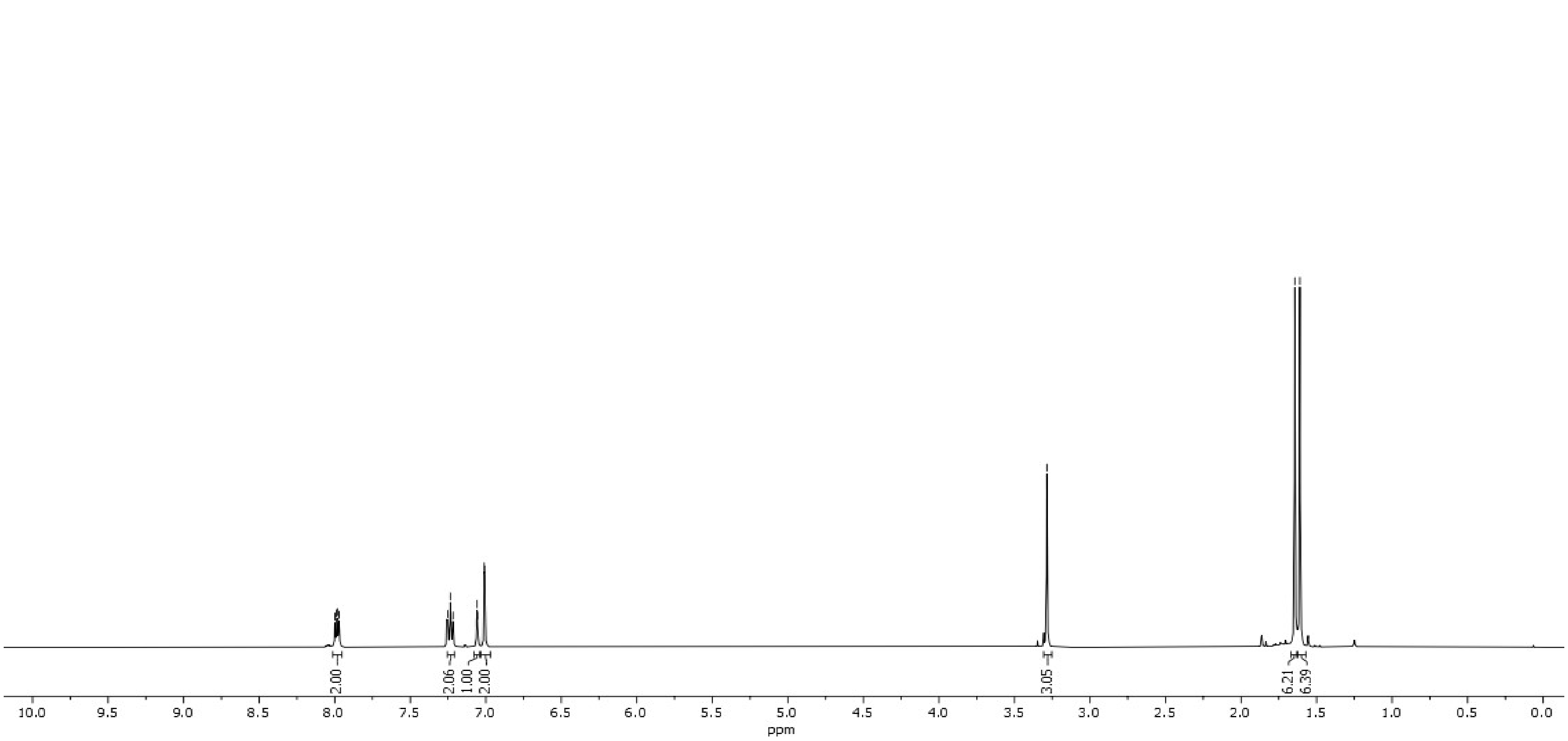

^{13}C NMR of 2,2'-(5-(((4-fluorophenyl)(methyl)(oxo)-λ^6-sulfanylidene)amino)-1,3-phenylene)bis(2-methylpropanenitrile) (18)

$CDCl_3$, 298 K

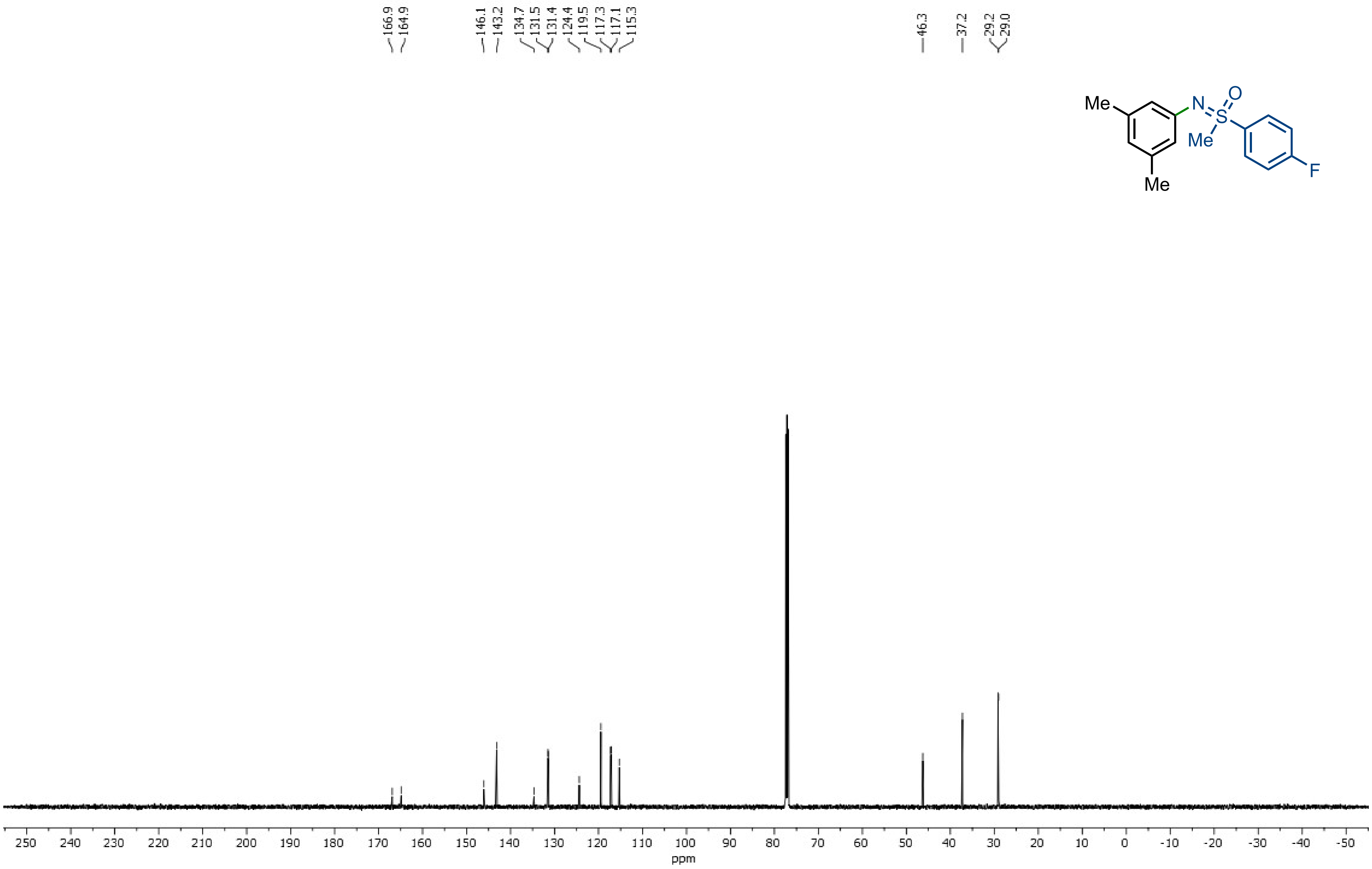

^{19}F NMR of 2,2'-(5-(((4-fluorophenyl)(methyl)(oxo)-λ^{6}-sulfanylidene)amino)-1,3-phenylene)bis(2-methylpropanenitrile) (18)

$CDCl_3$, 298 K

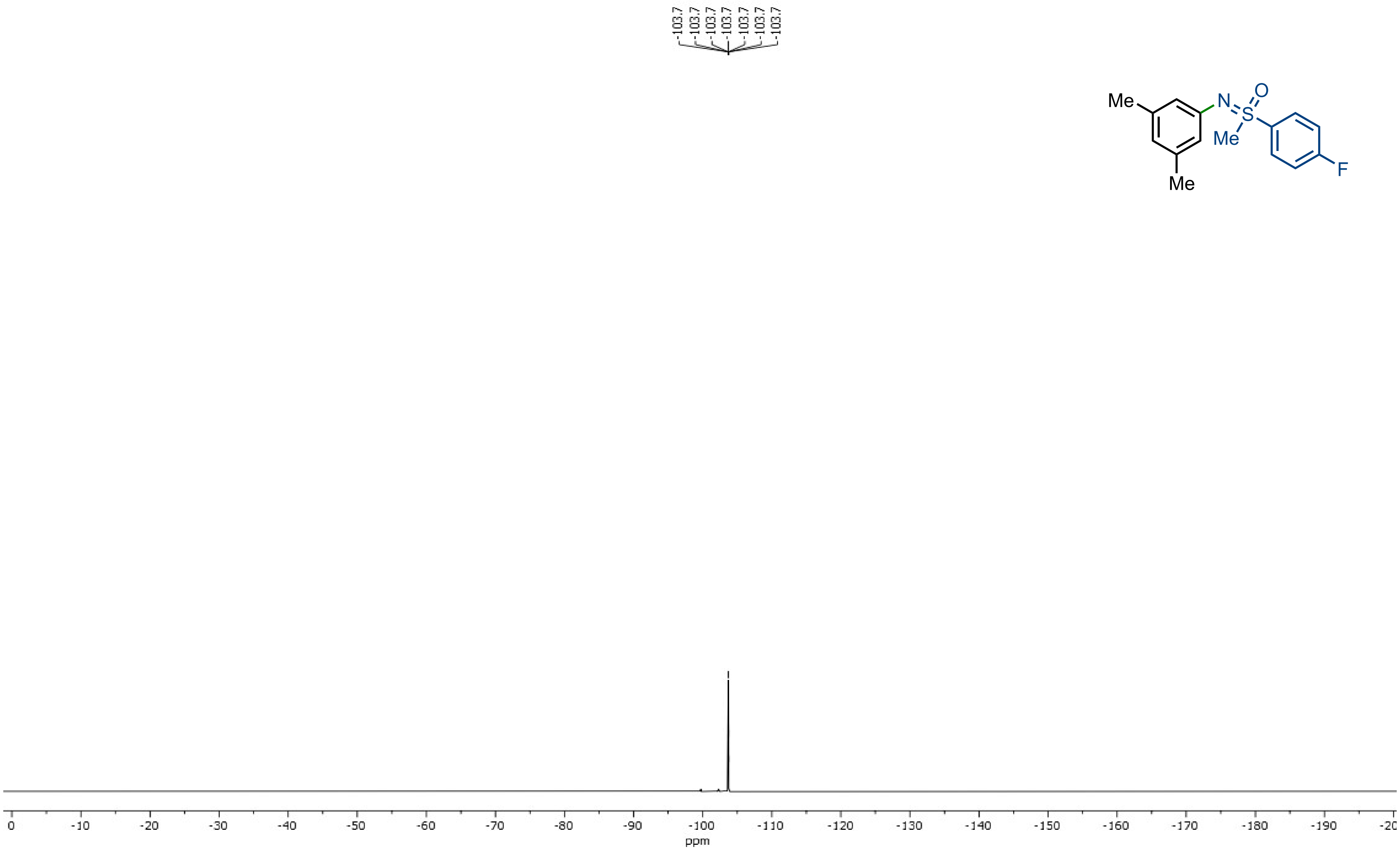

^{1}H NMR of celecoxib analogue 19

CD_2Cl_2, 298 K

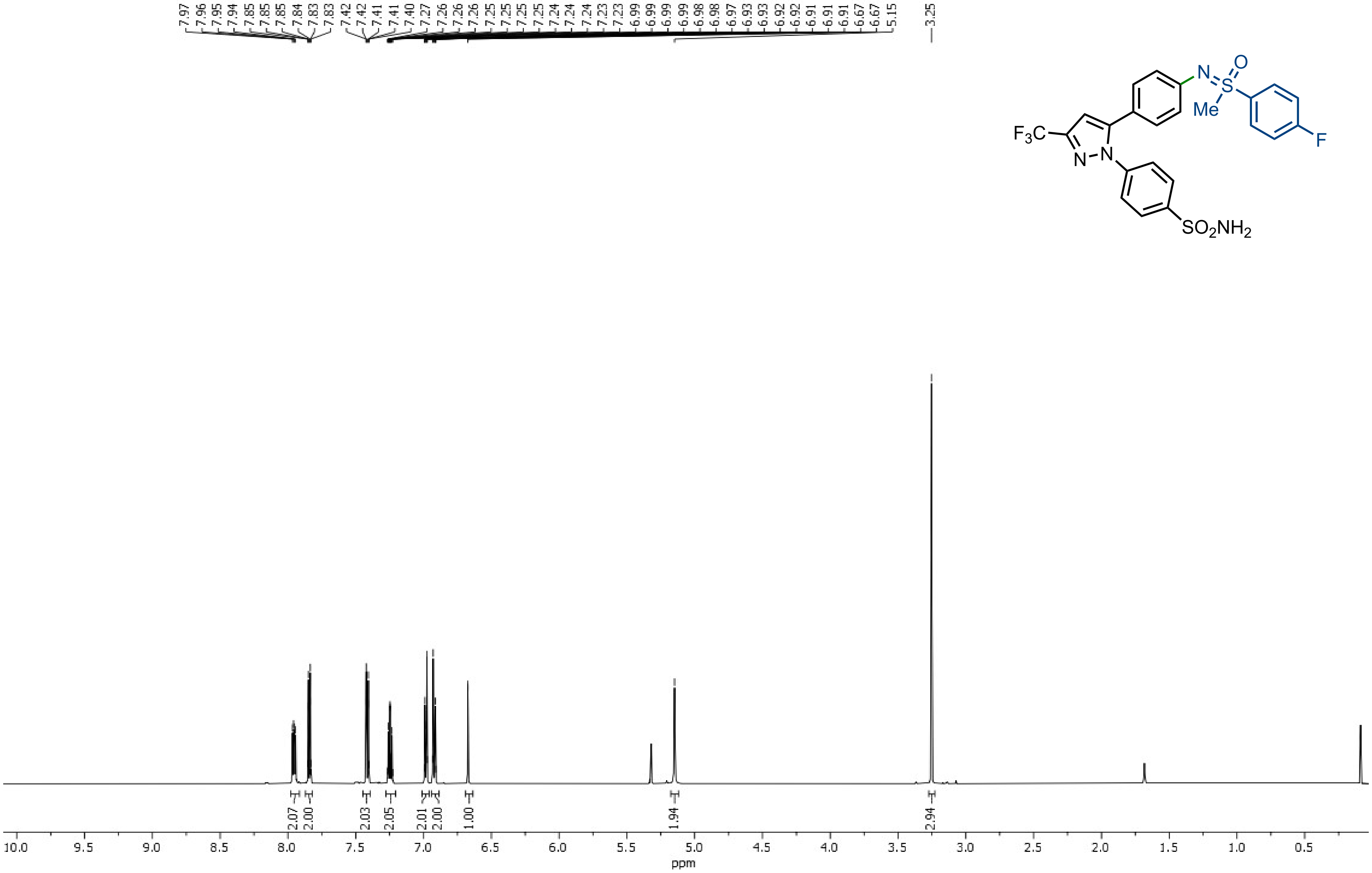

^{13}C NMR of celecoxib analogue 19

CD_2Cl_2, 298 K

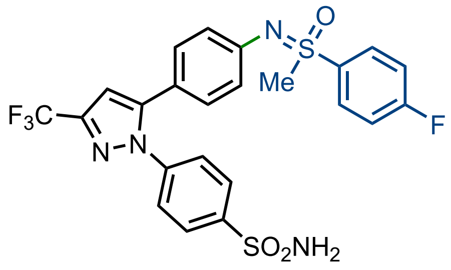

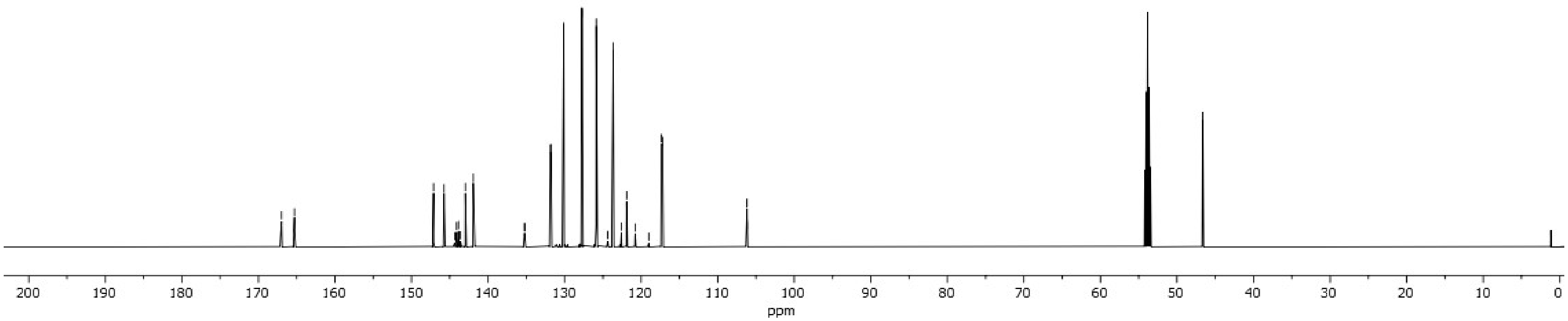

^{19}F NMR of celecoxib analogue 19

CD_2Cl_2, 298 K

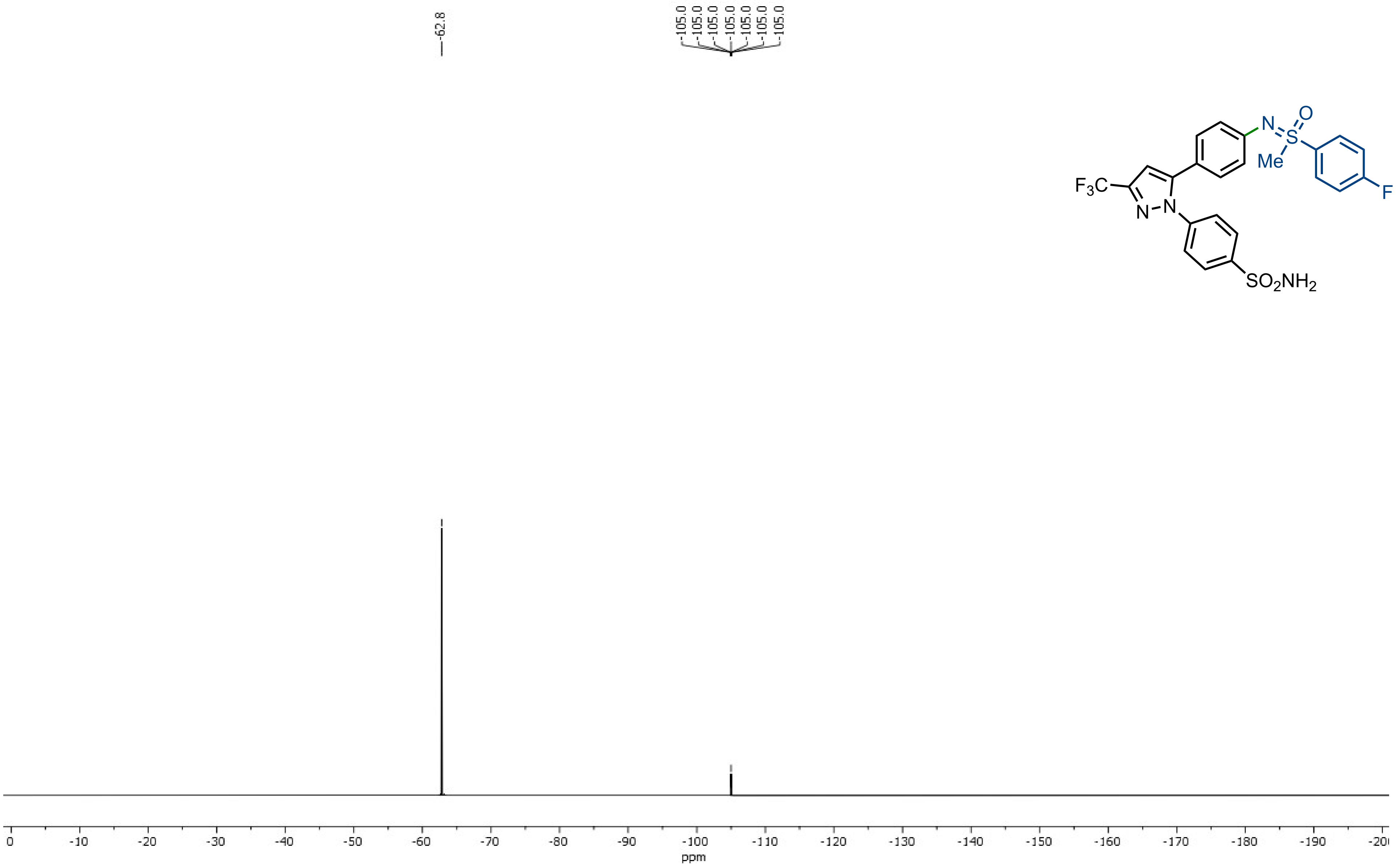

^{1}H NMR of triclosan derivative 20

CDCl$_3$, 298 K

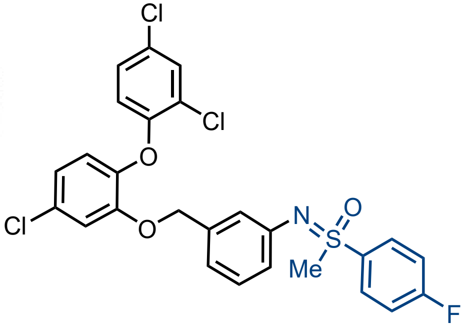

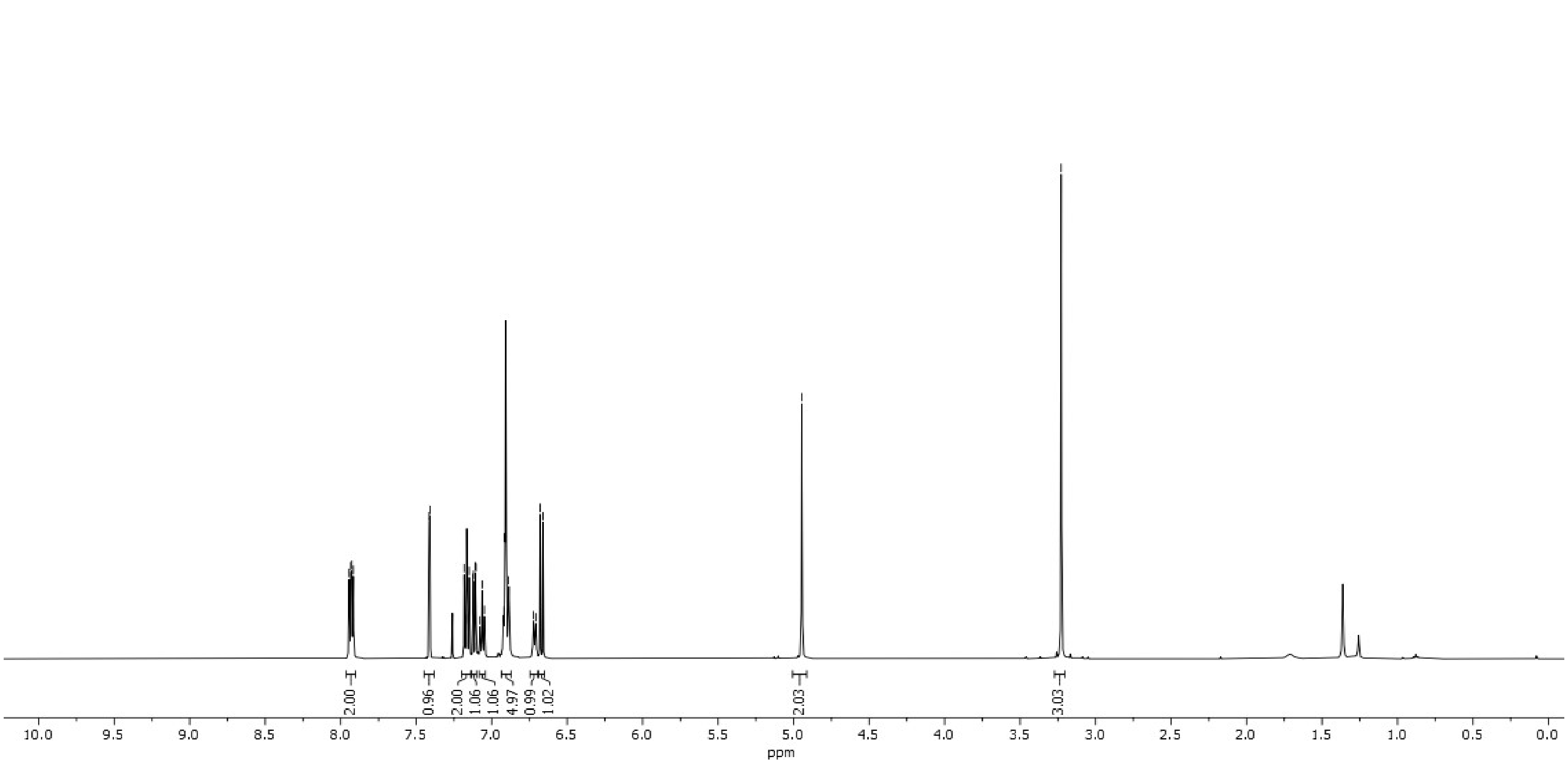

^{13}C NMR of triclosan derivative 20

$CDCl_3$, 298 K

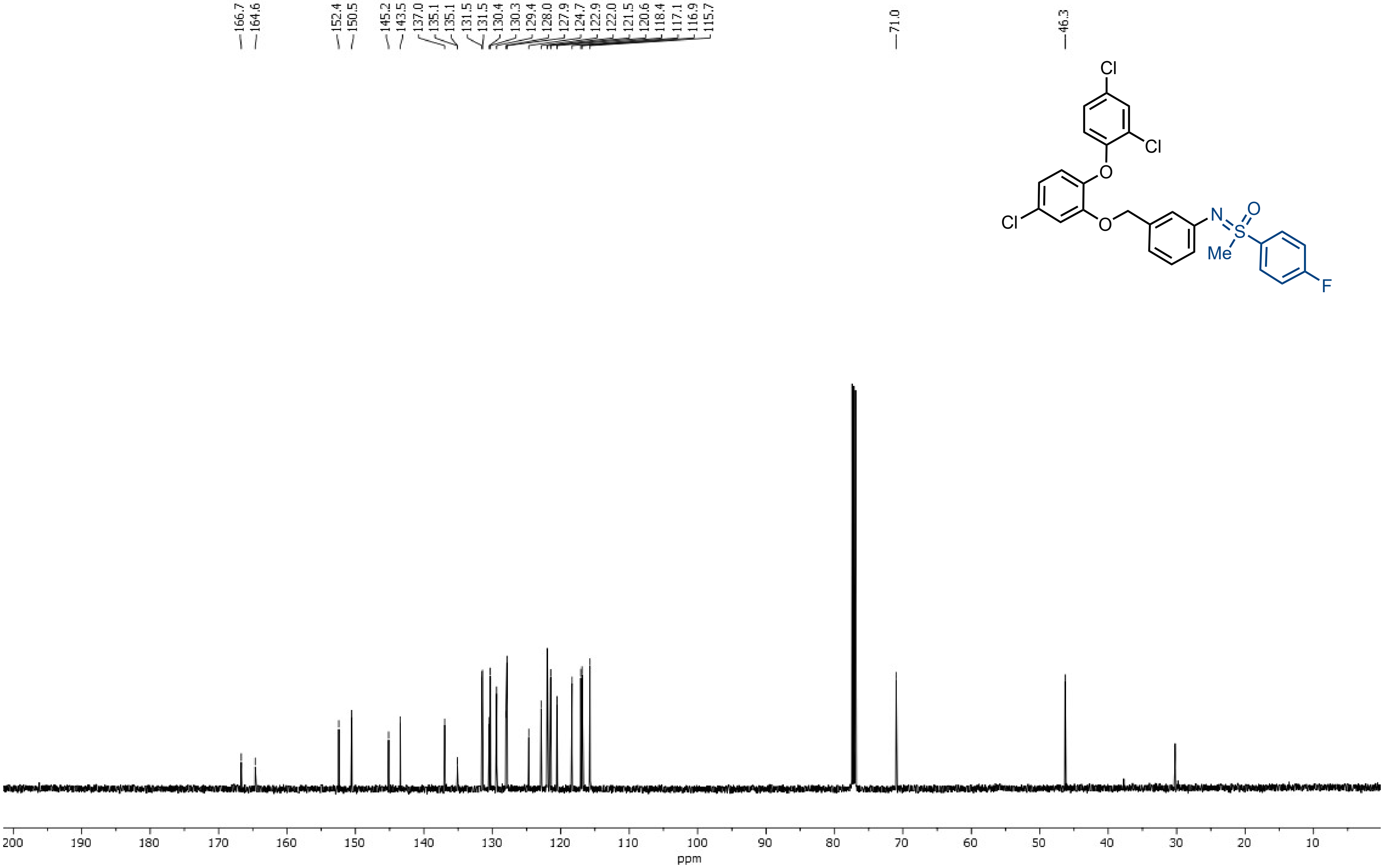

^{19}F NMR of triclosan derivative 20

$CDCl_3$, 298 K

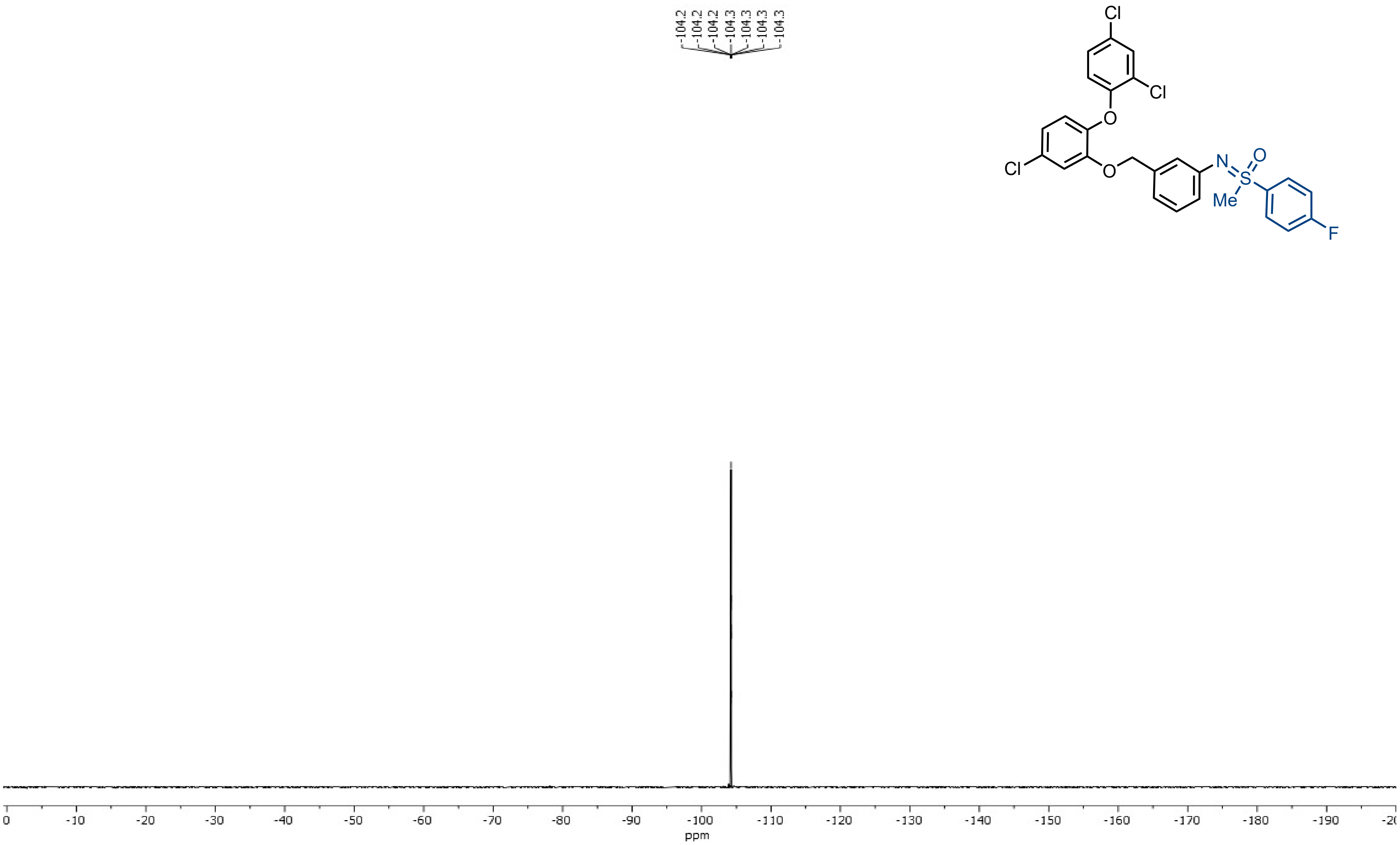

^{1}H NMR of (*R*)-methyl(phenyl)((4-(trifluoromethyl)phenyl)imino)-λ^{6}-sulfanone (21)

$CDCl_3$, 298 K

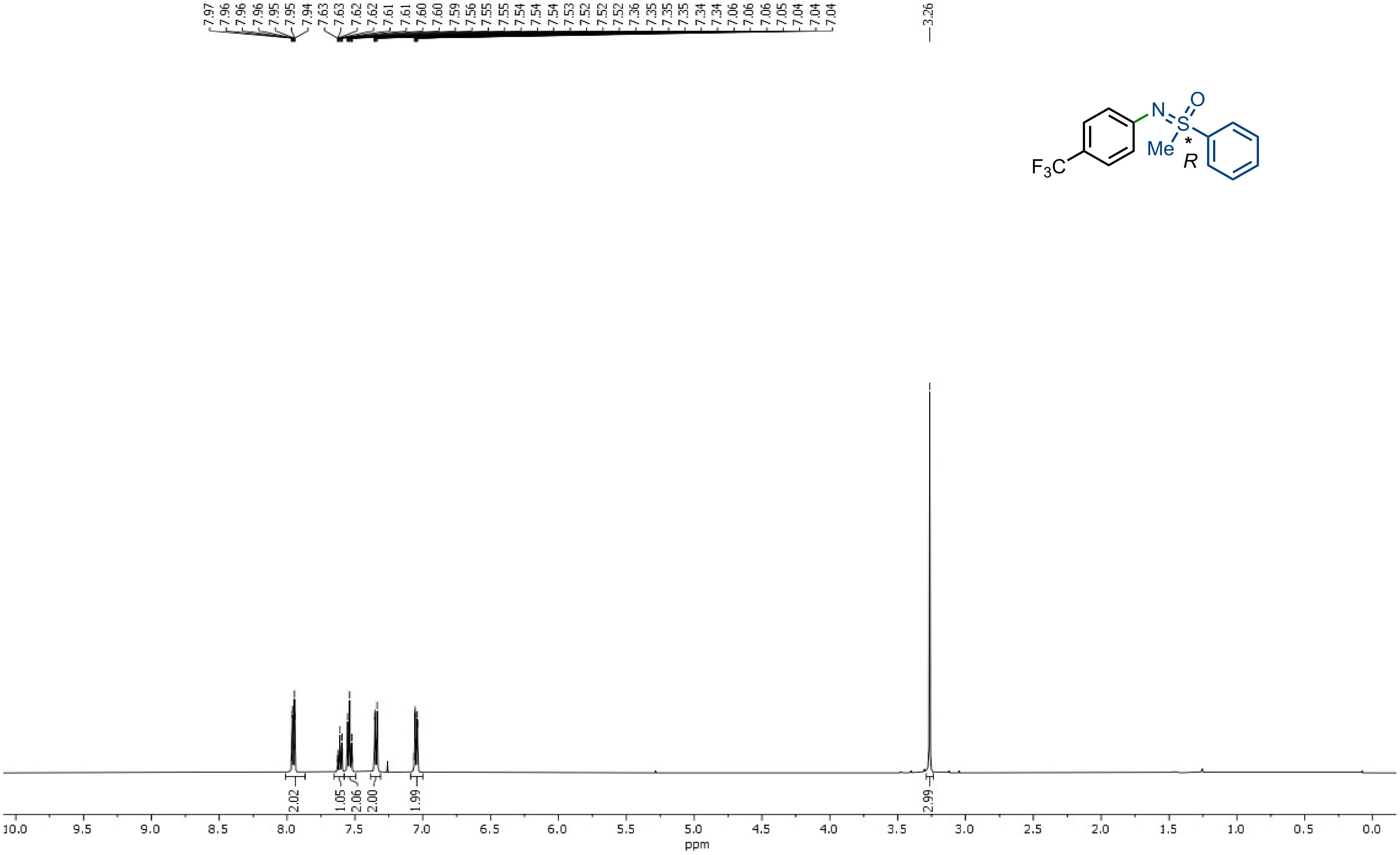

^{13}C NMR of (*R*)-methyl(phenyl)((4-(trifluoromethyl)phenyl)imino)-λ^6-sulfanone (21)

$CDCl_3$, 298 K

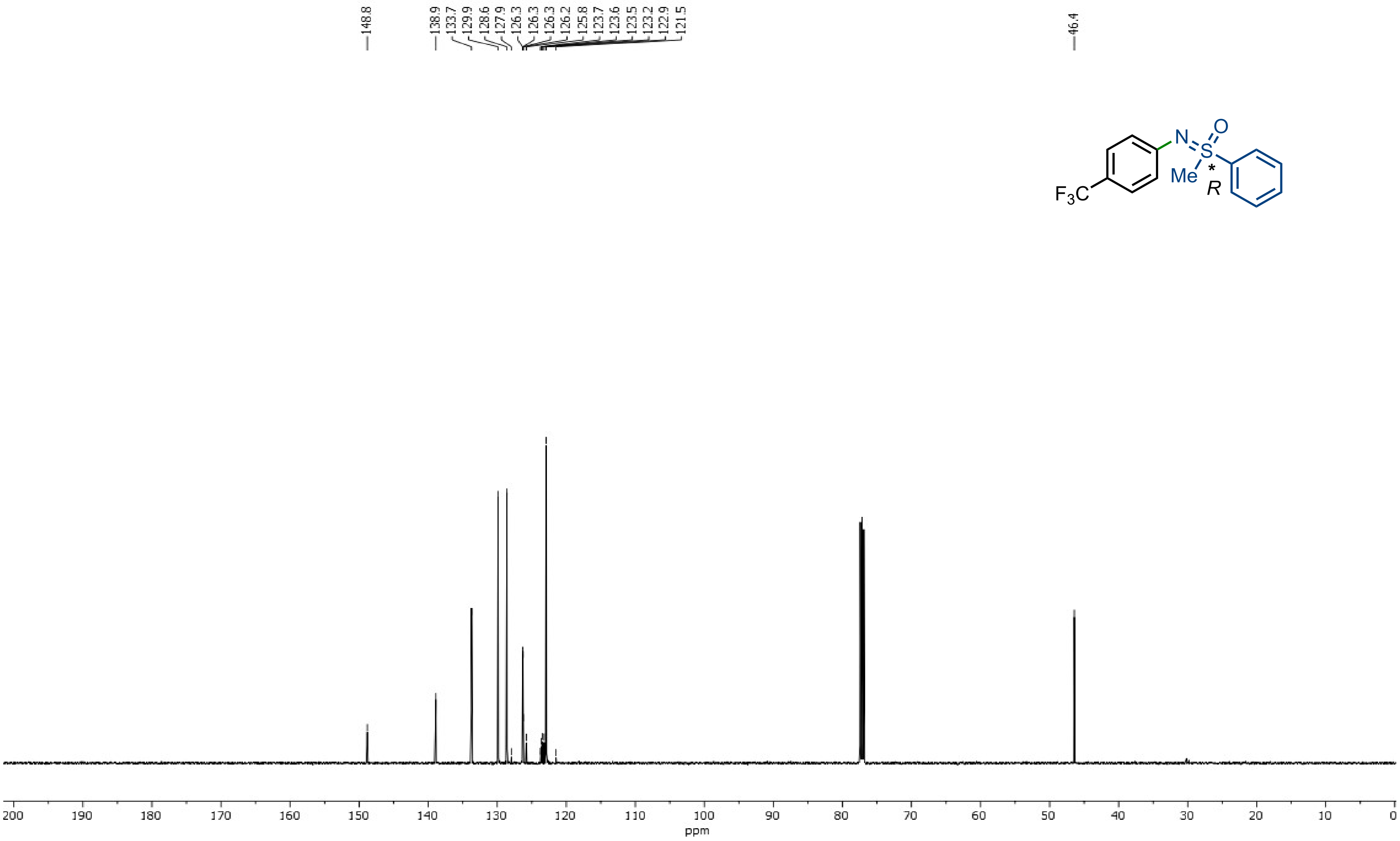

^{19}F NMR of (*R*)-methyl(phenyl)((4-(trifluoromethyl)phenyl)imino)-λ[6]-sulfanone (21)

CDCl3, 298 K

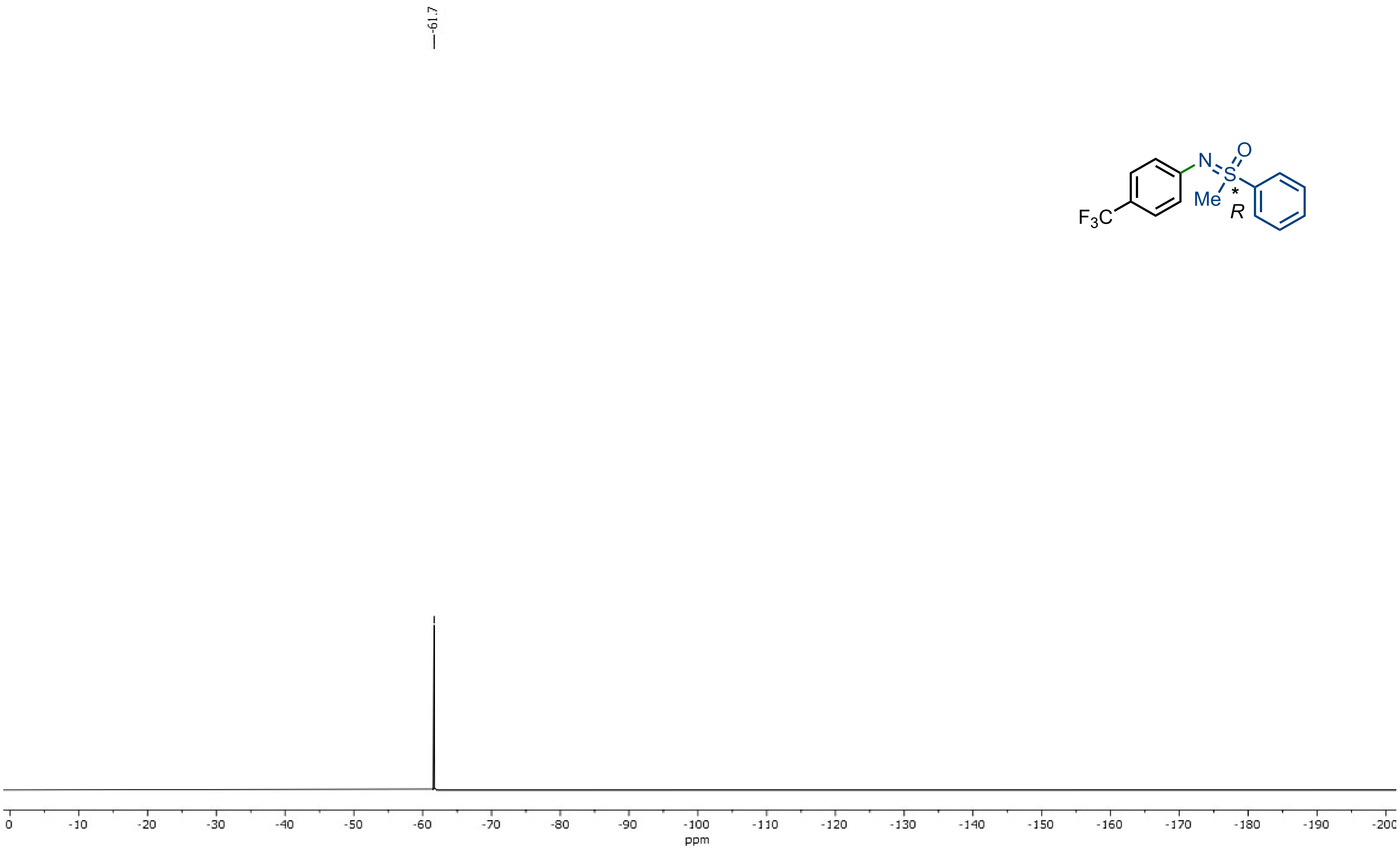

^{1}H NMR of (*S*)-methyl(phenyl)((4-(trifluoromethyl)phenyl)imino)-λ^6-sulfanone (22)

$CDCl_3$, 298 K

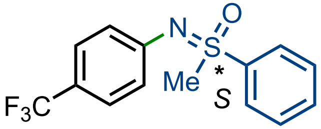

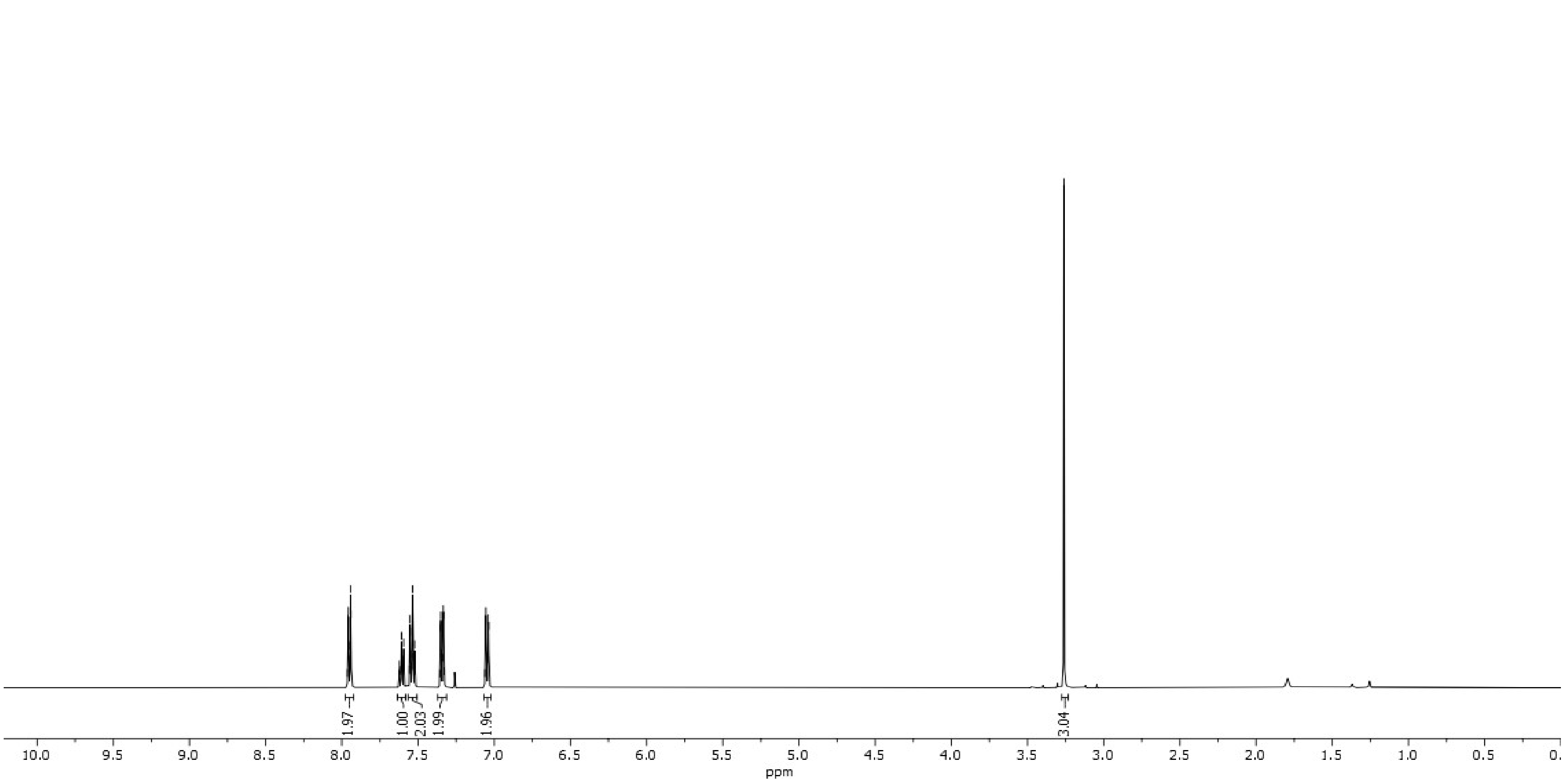

^{13}C NMR of (*S*)-methyl(phenyl)((4-(trifluoromethyl)phenyl)imino)-λ^6-sulfanone (22)

$CDCl_3$, 298 K

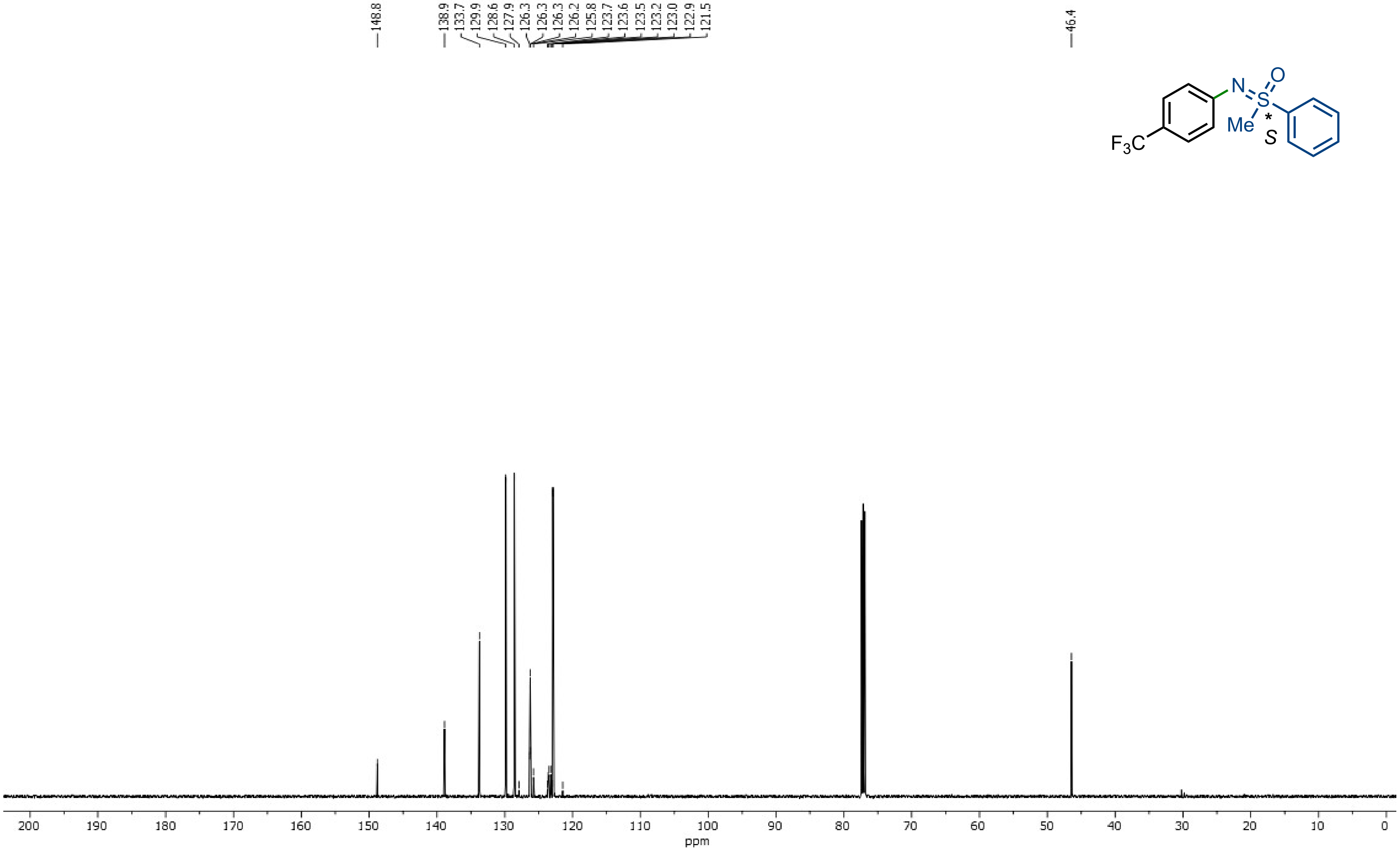

^{19}F NMR of (*S*)-methyl(phenyl)((4-(trifluoromethyl)phenyl)imino)-λ^6-sulfanone (22)

$CDCl_3$, 298 K

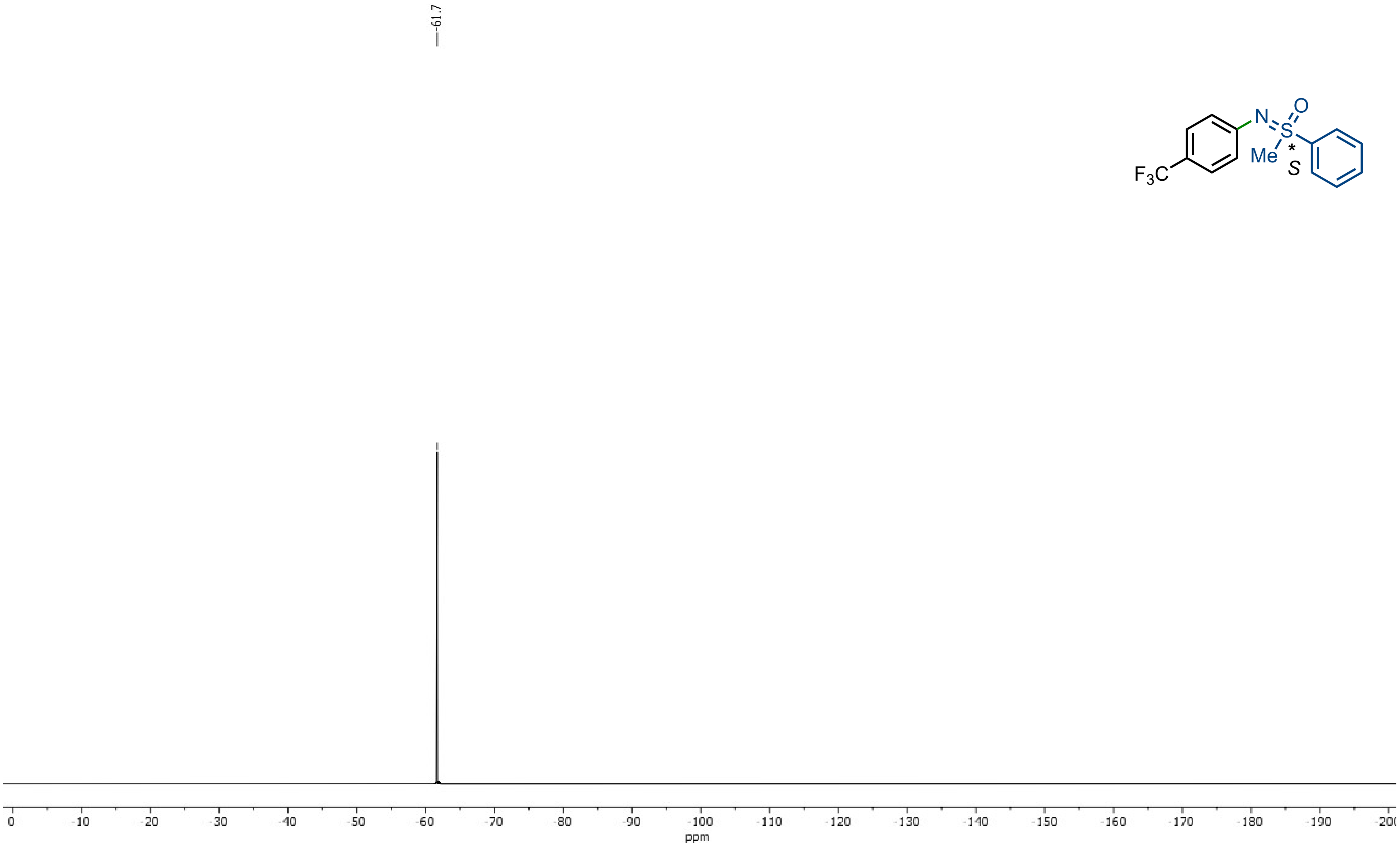

^{1}H NMR of ethyl(phenyl)((4-(trifluoromethyl)phenyl)imino)-λ^6-sulfanone (23)

$CDCl_3$, 298 K

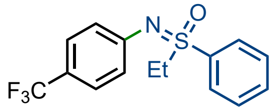

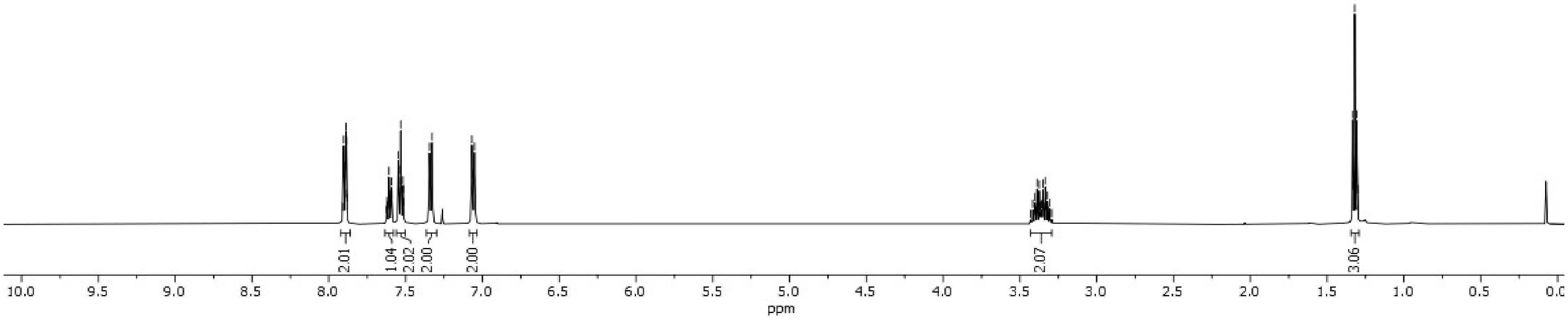

^{13}C NMR of ethyl(phenyl)((4-(trifluoromethyl)phenyl)imino)-λ^{6}-sulfanone (23)

$CDCl_3$, 298 K

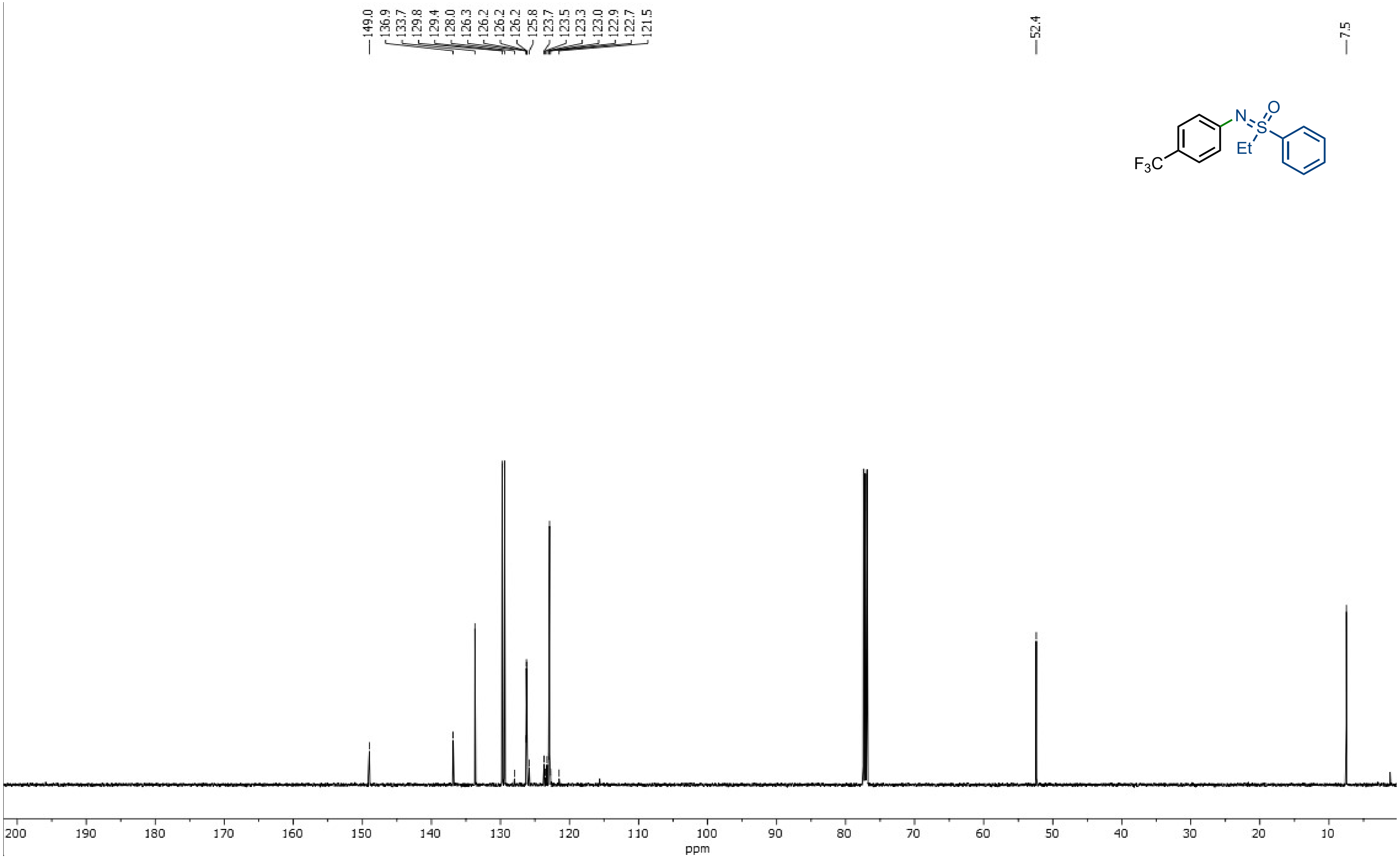

^{19}F NMR of ethyl(phenyl)((4-(trifluoromethyl)phenyl)imino)-λ^6-sulfanone (23)

$CDCl_3$, 298 K

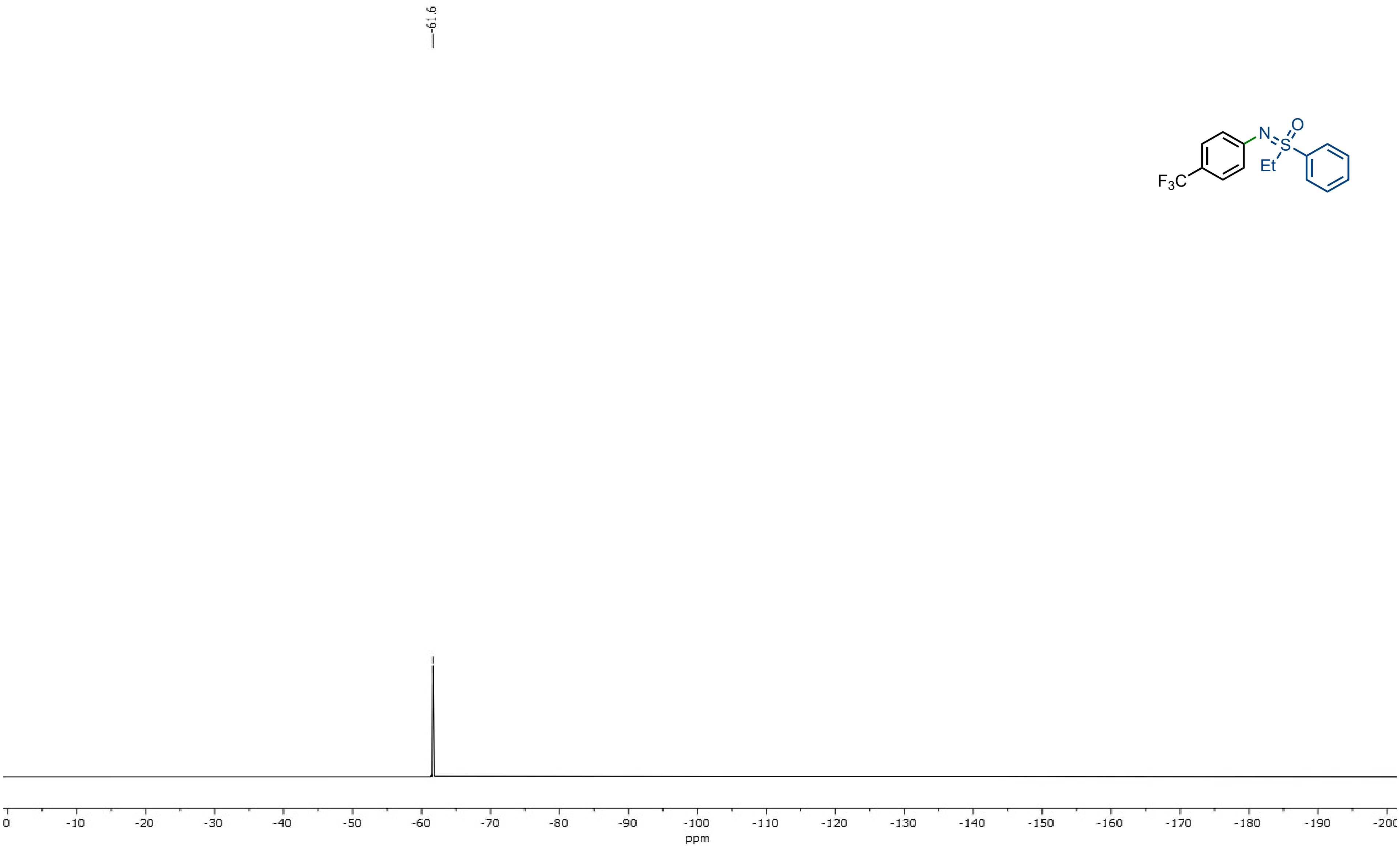

^{1}H NMR of (4-chlorophenyl)(methyl)((4-(trifluoromethyl)phenyl)imino)-λ^6-sulfanone (24)

$CDCl_3$, 298 K

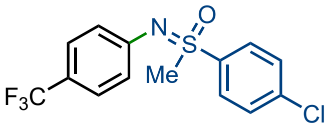

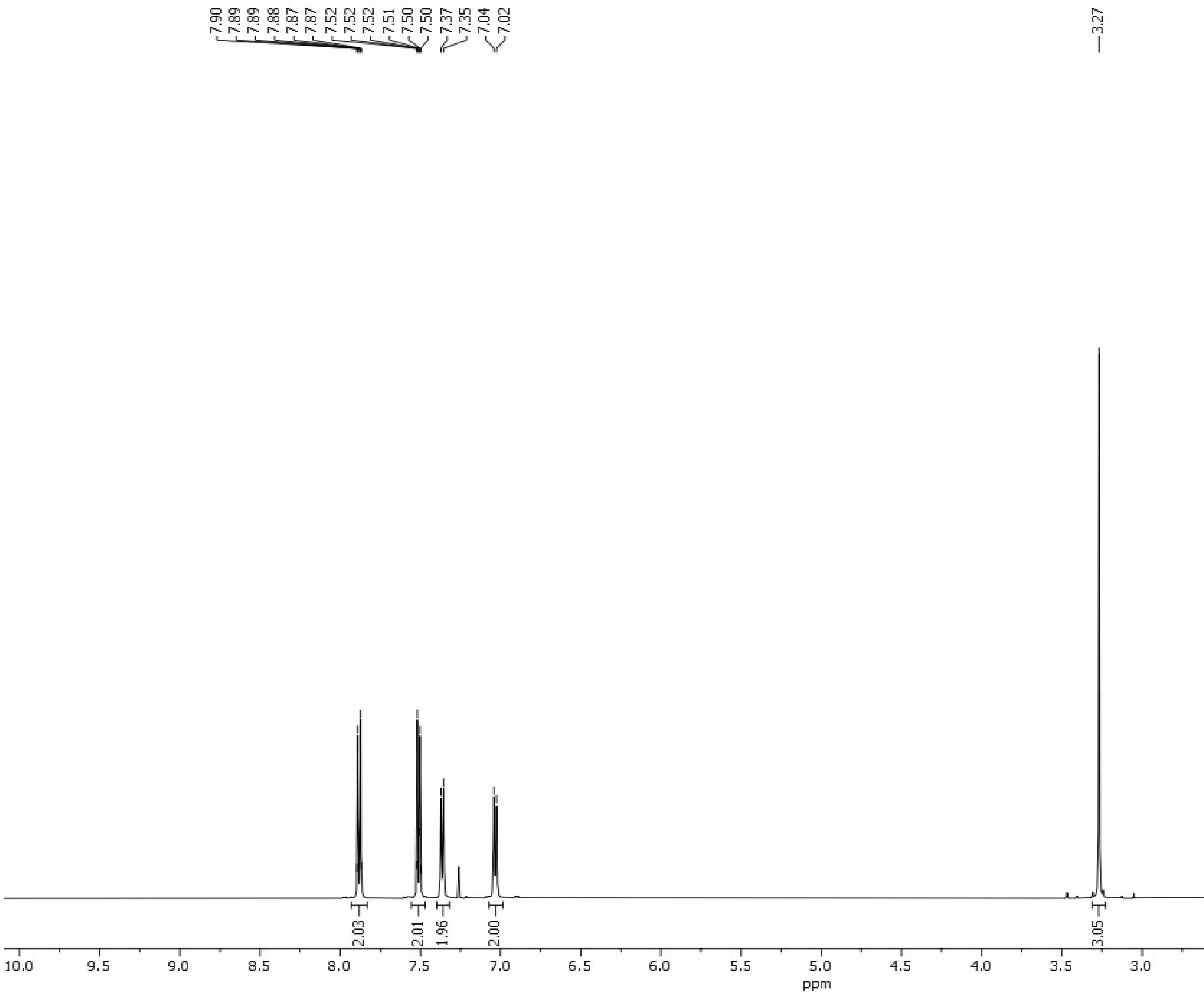

^{13}C NMR of (4-chlorophenyl)(methyl)((4-(trifluoromethyl)phenyl)imino)-λ^6-sulfanone (24)

CDCl$_3$, 298 K

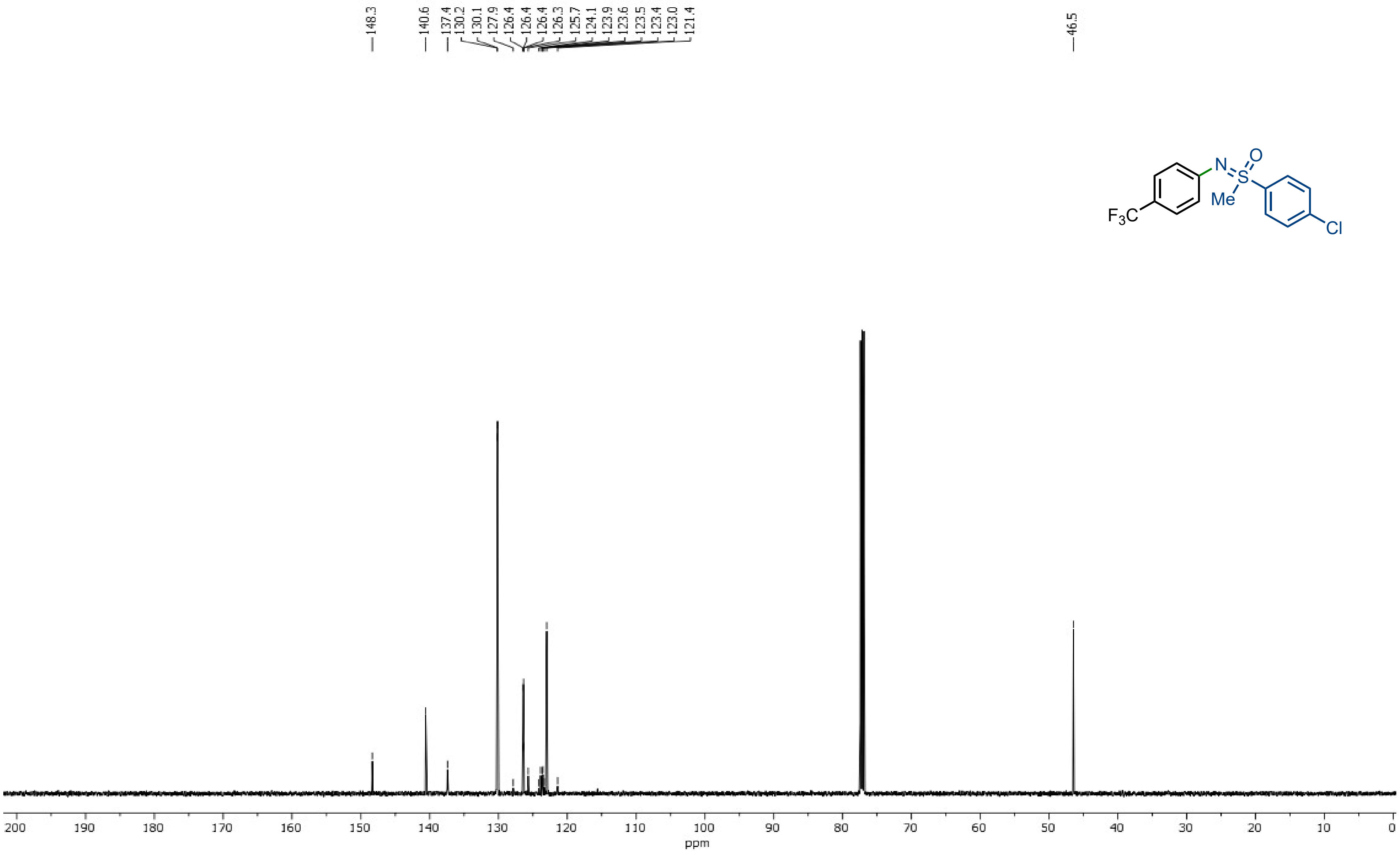

^{19}F NMR of (4-chlorophenyl)(methyl)((4-(trifluoromethyl)phenyl)imino)-λ[6]-sulfanone (24)

$CDCl_3$, 298 K

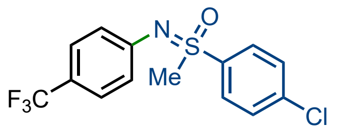

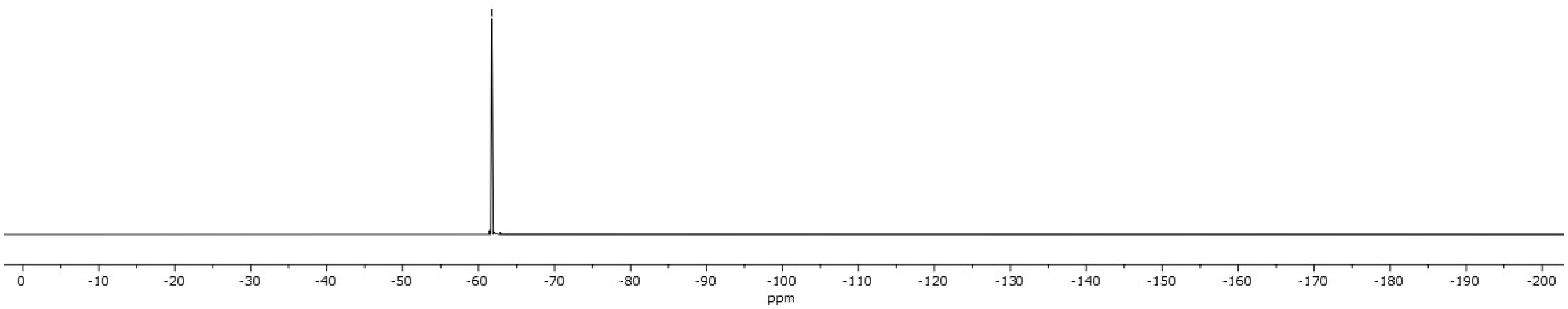

^{1}H NMR of (3,5-dichlorophenyl)(methyl)((4-(trifluoromethyl)phenyl)imino)-λ^6-sulfanone (25)

$CDCl_3$, 298 K

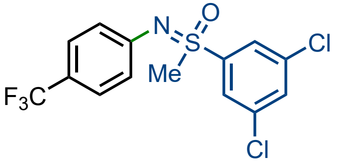

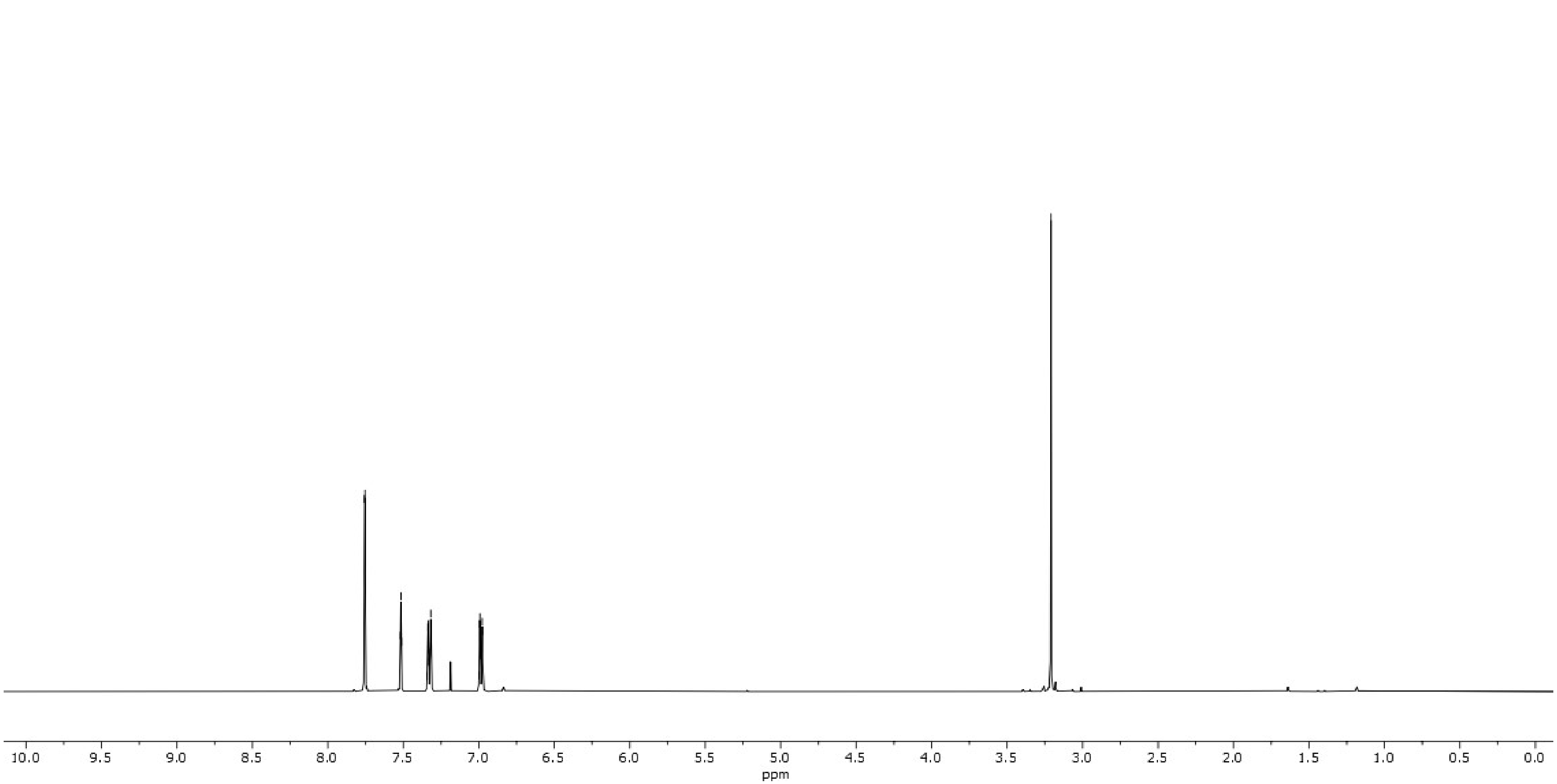

^{13}C NMR of (3,5-dichlorophenyl)(methyl)((4-(trifluoromethyl)phenyl)imino)-λ^{6}-sulfanone (25)

$CDCl_3$, 298 K

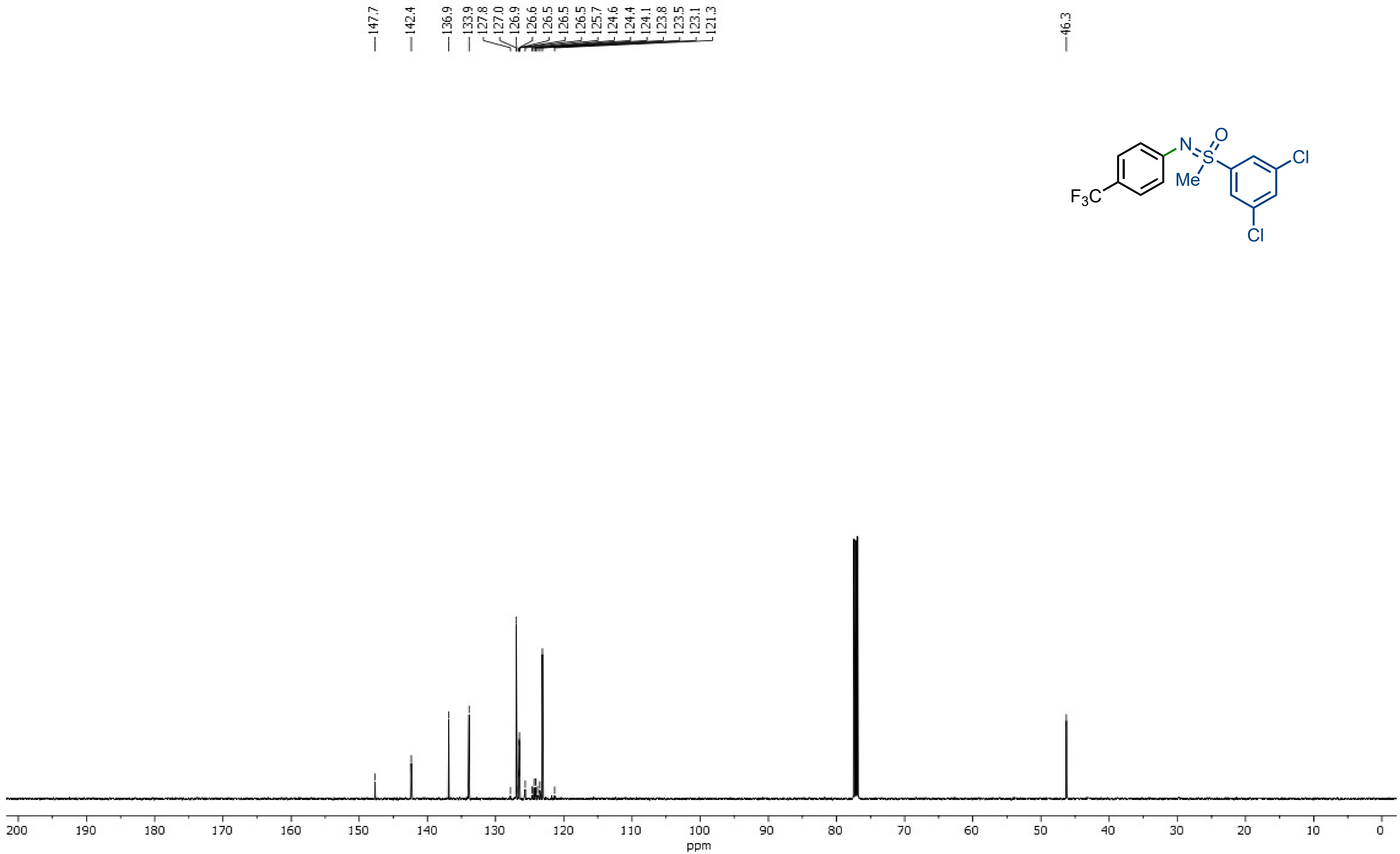

^{19}F NMR of (3,5-dichlorophenyl)(methyl)((4-(trifluoromethyl)phenyl)imino)-λ^{6}-sulfanone (25)

$CDCl_3$, 298 K

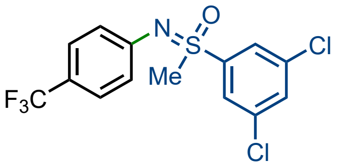

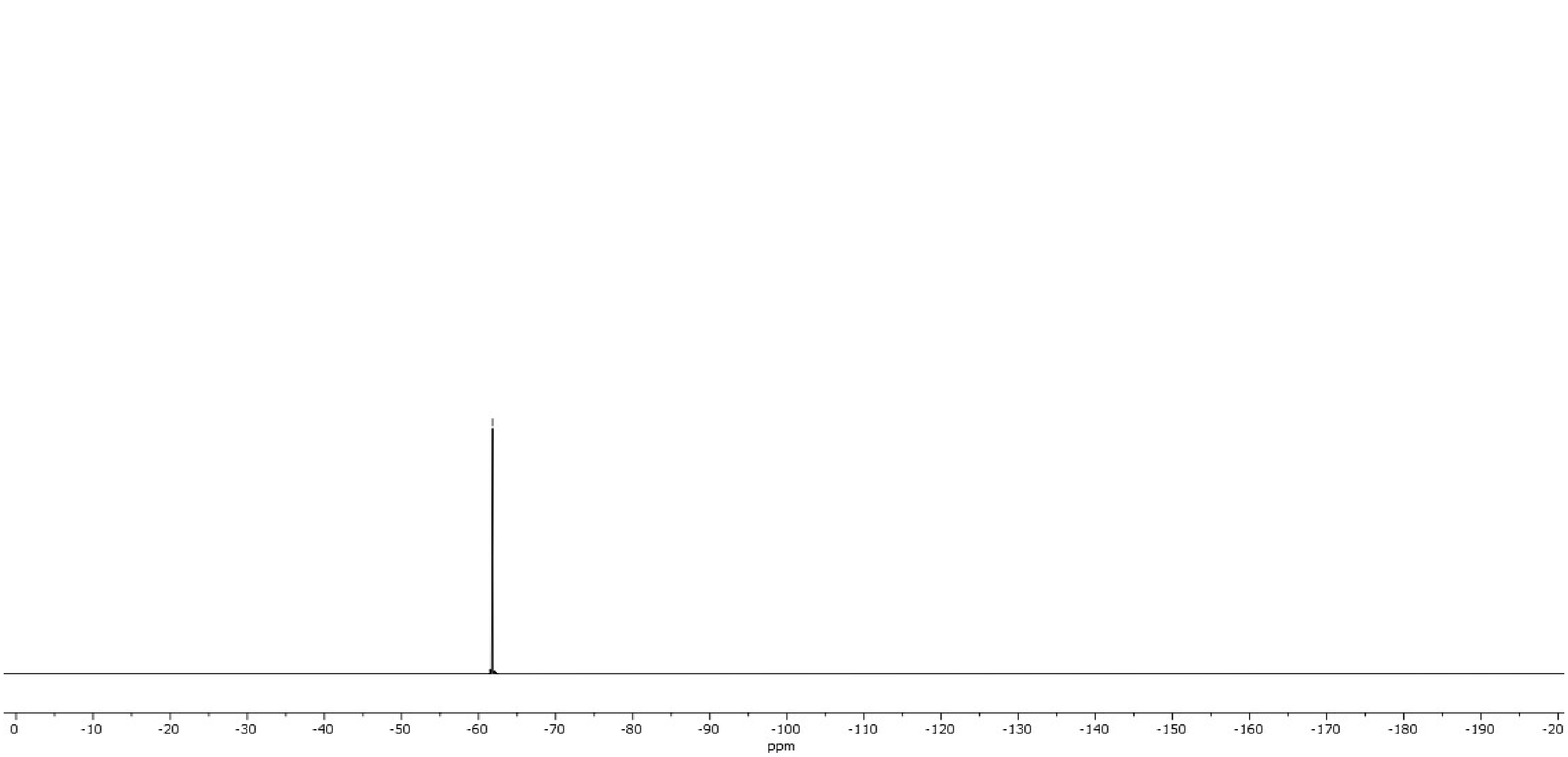

^{1}H NMR of (3-methylphenyl)(methyl)((4-(trifluoromethyl)phenyl)imino)-λ[6]-sulfanone (26)

CDCl$_3$, 298 K

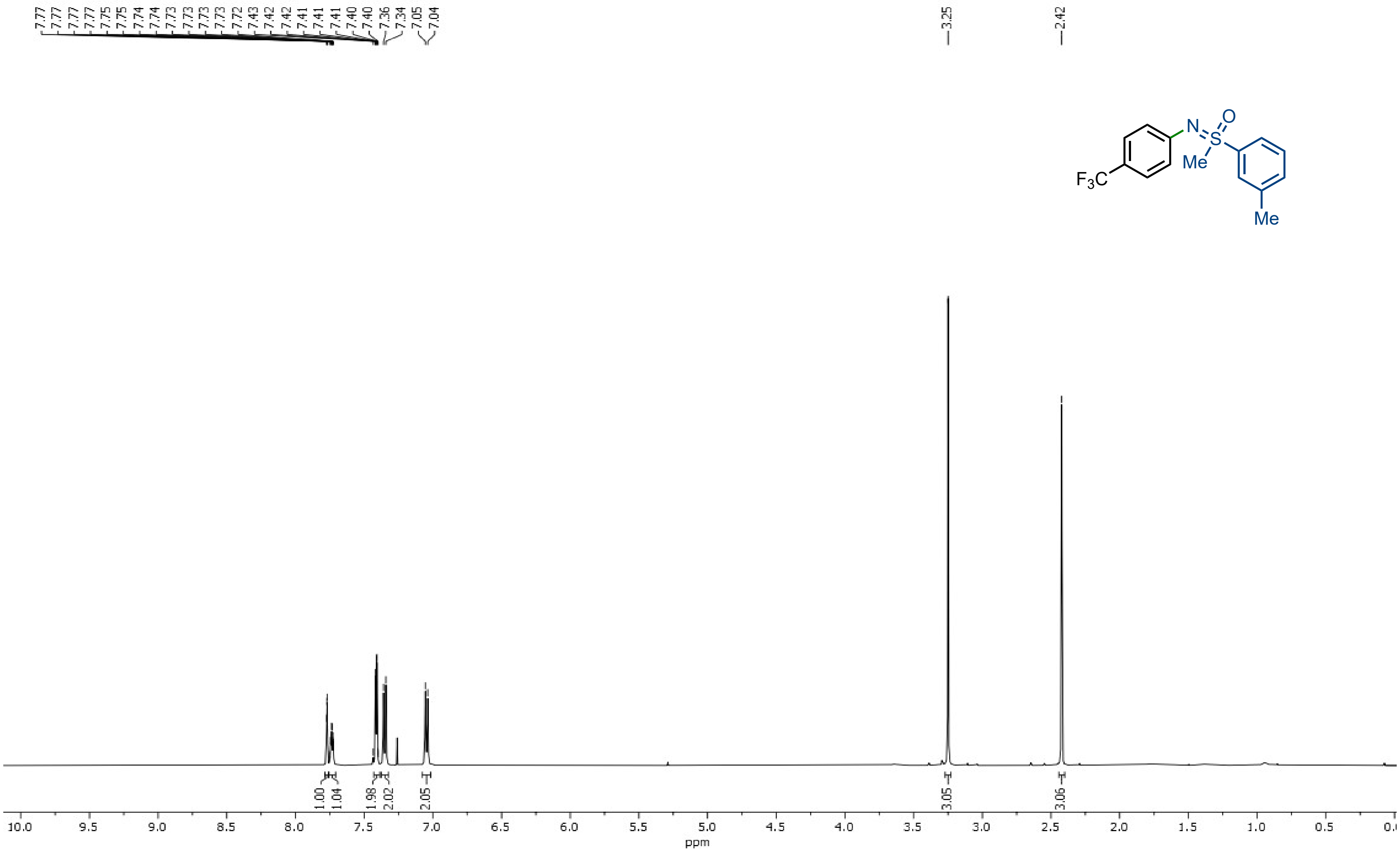

^{13}C NMR of (3-methylphenyl)(methyl)((4-(trifluoromethyl)phenyl)imino)-λ^6-sulfanone (26)

$CDCl_3$, 298 K

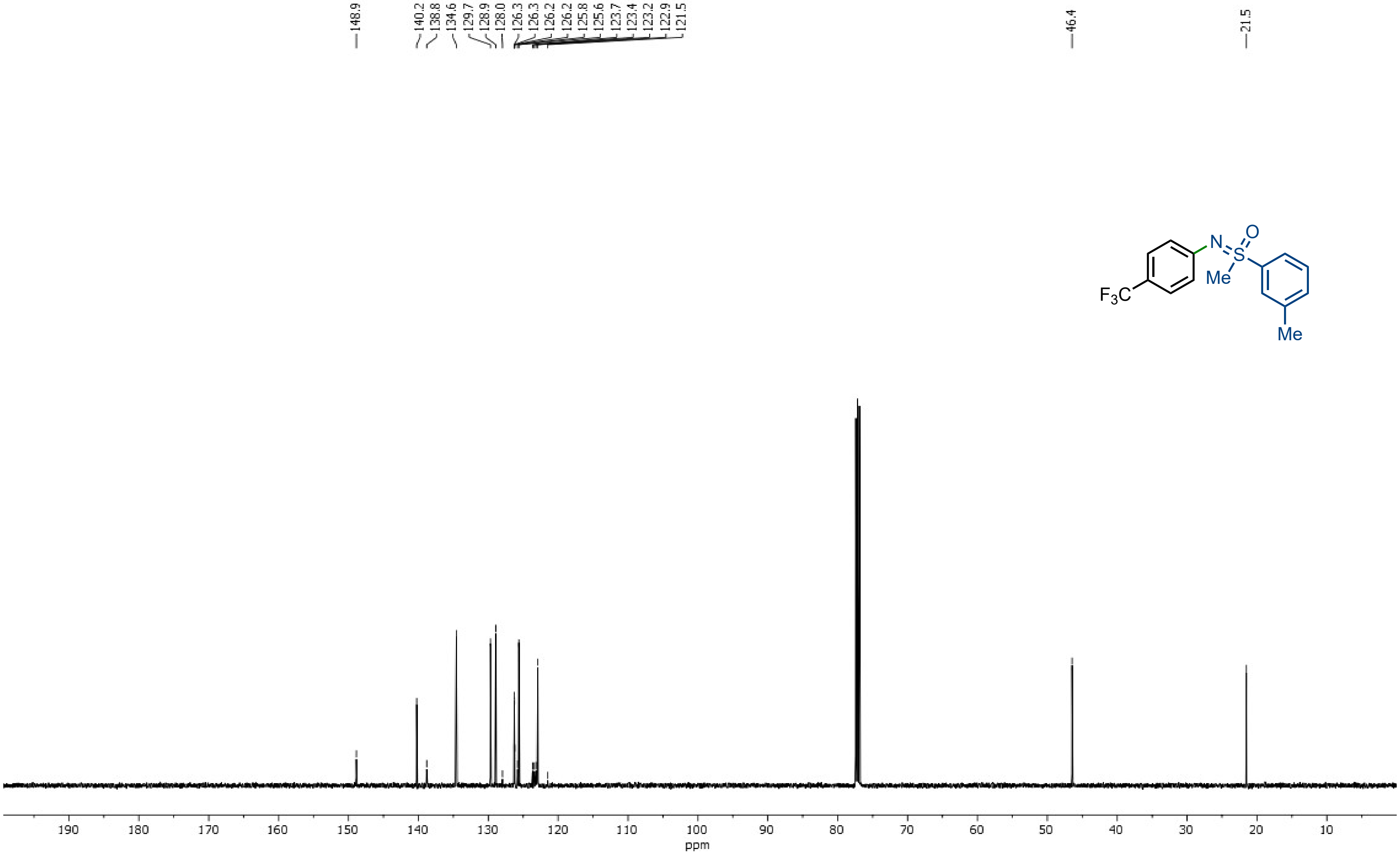

^{19}F NMR of (3-methylphenyl)(methyl)((4-(trifluoromethyl)phenyl)imino)-λ^6-sulfanone (26)

$CDCl_3$, 298 K

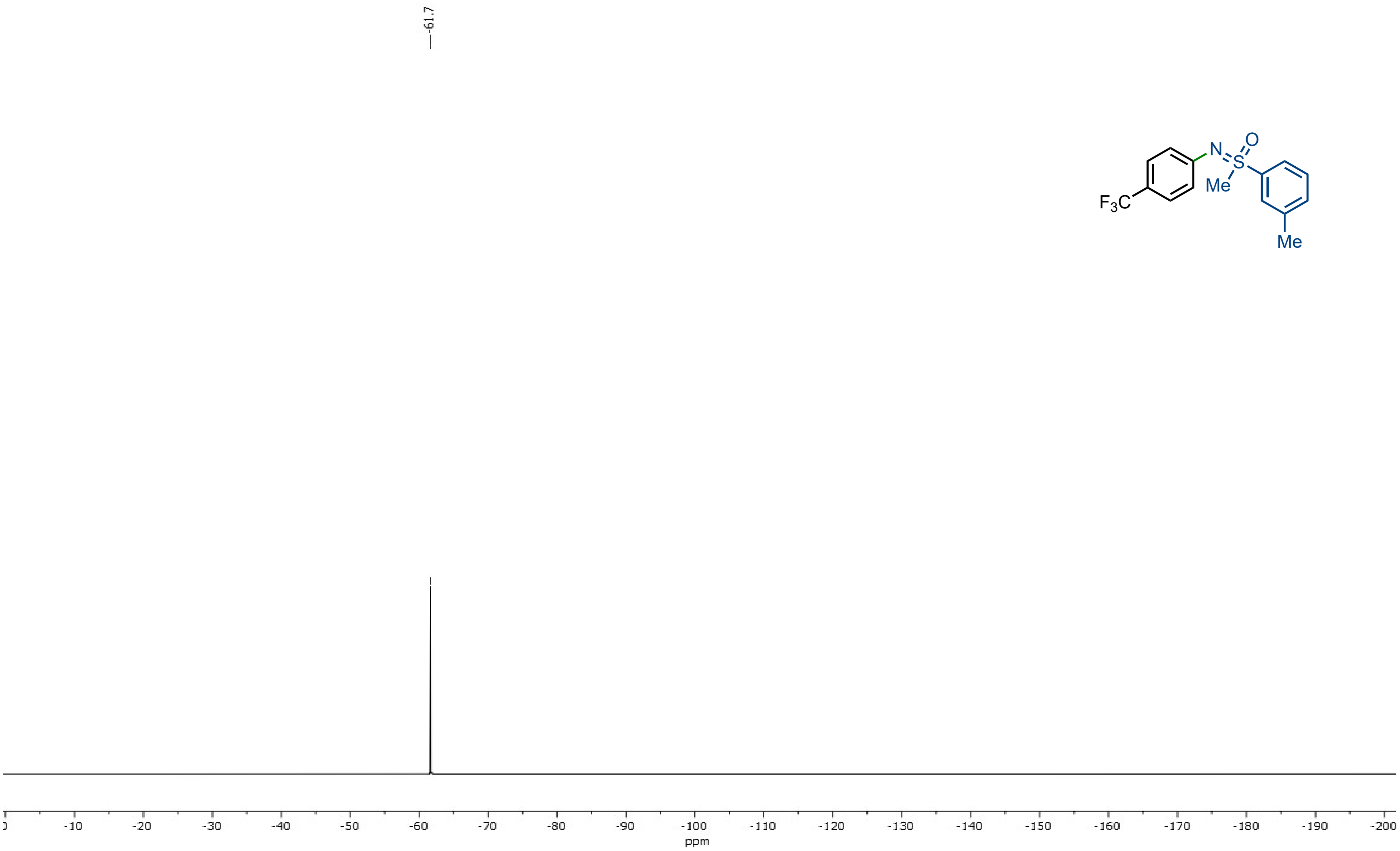

^{1}H NMR of (4-cyanophenyl)(methyl)((4-(trifluoromethyl)phenyl)imino)-λ^{6}-sulfanone (27)

CDCl$_3$, 298 K

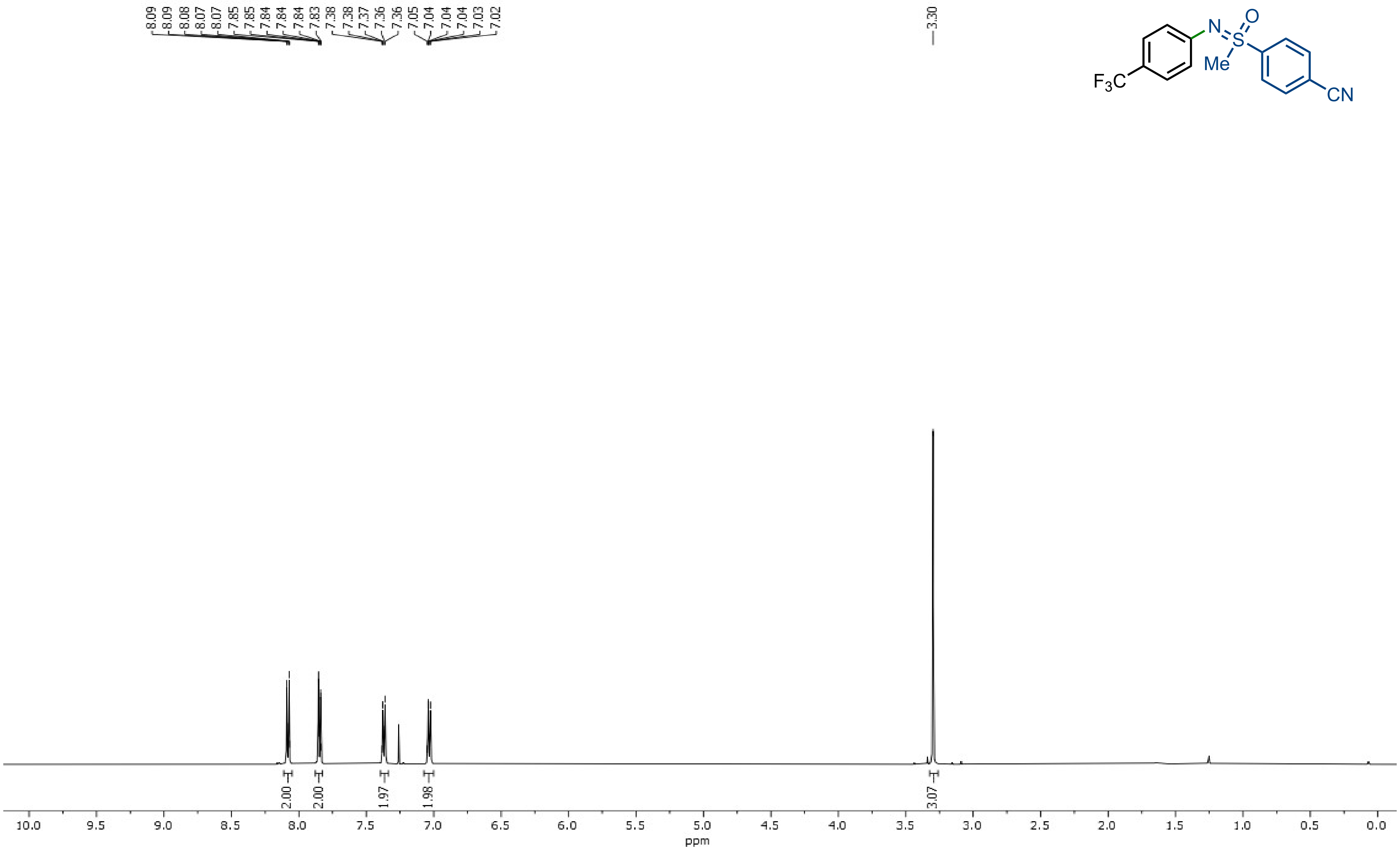

^{13}C NMR of (4-cyanophenyl)(methyl)((4-(trifluoromethyl)phenyl)imino)-λ^{6}-sulfanone (27)

$CDCl_3$, 298 K

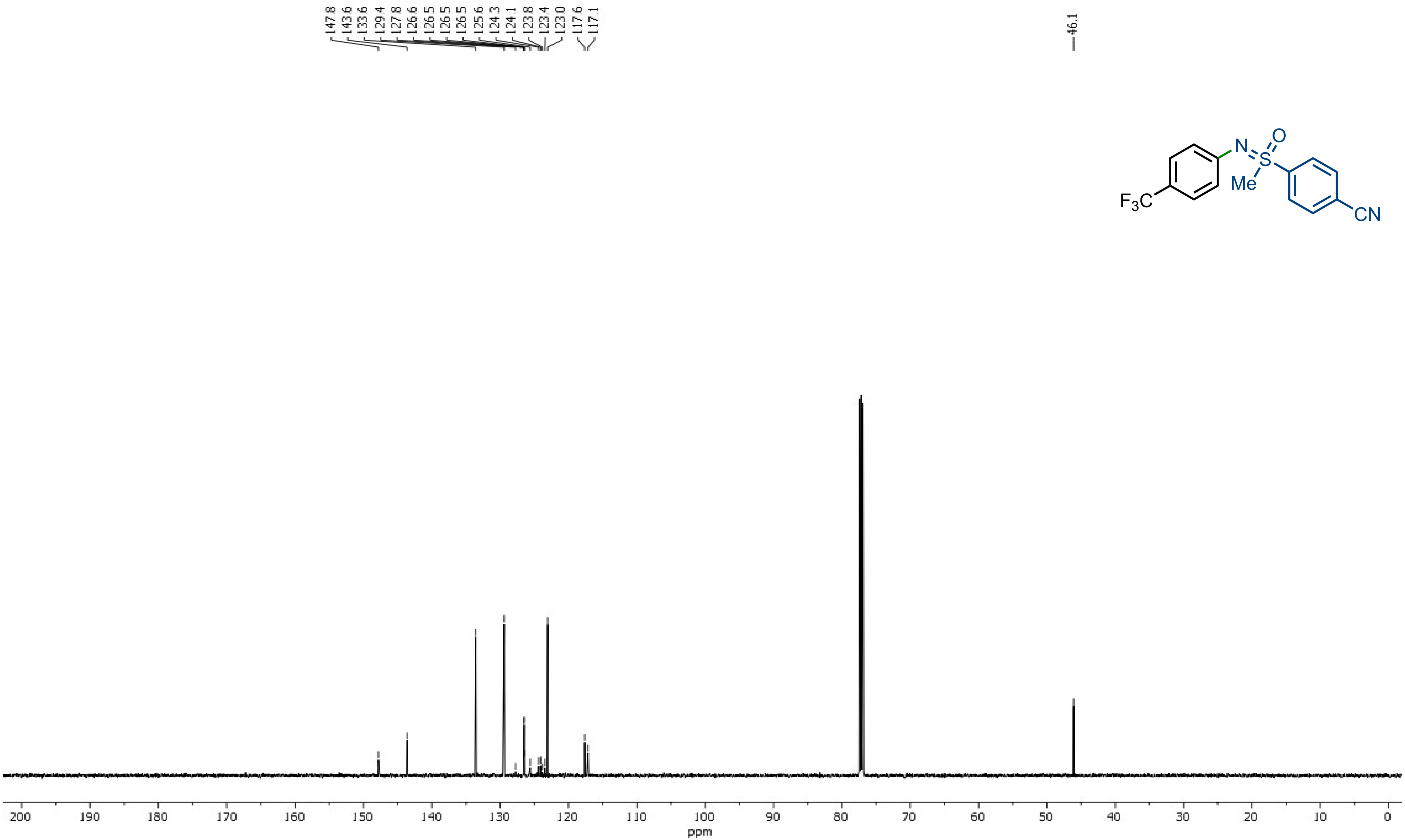

^{19}F NMR of (4-cyanophenyl)(methyl)((4-(trifluoromethyl)phenyl)imino)-λ^6-sulfanone (27)

$CDCl_3$, 298 K

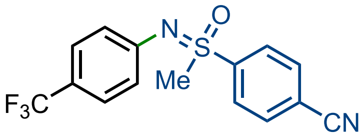

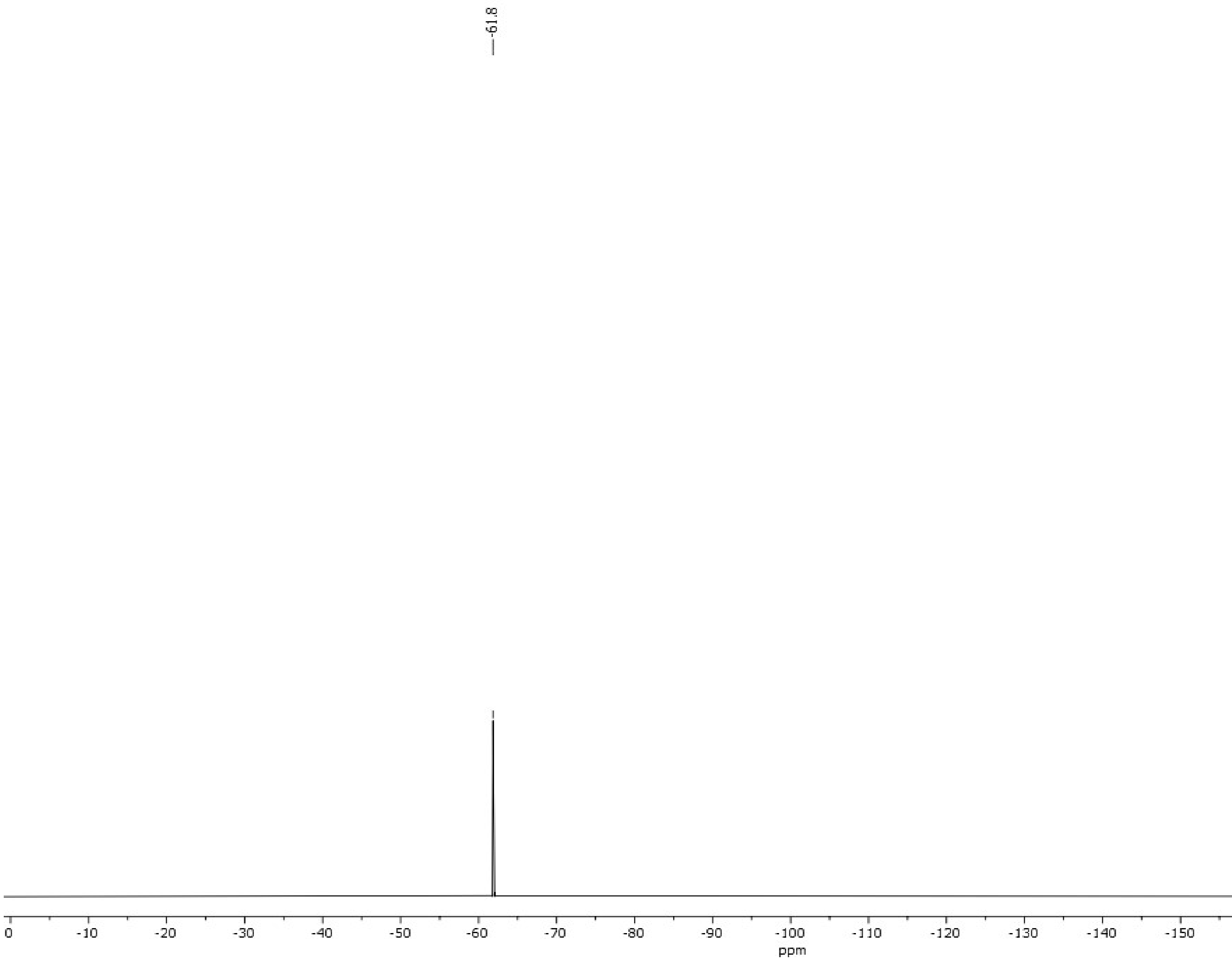

^{1}H NMR of (2-bromophenyl)(methyl)((4-(trifluoromethyl)phenyl)imino)-λ^6-sulfanone (28)

$CDCl_3$, 298 K

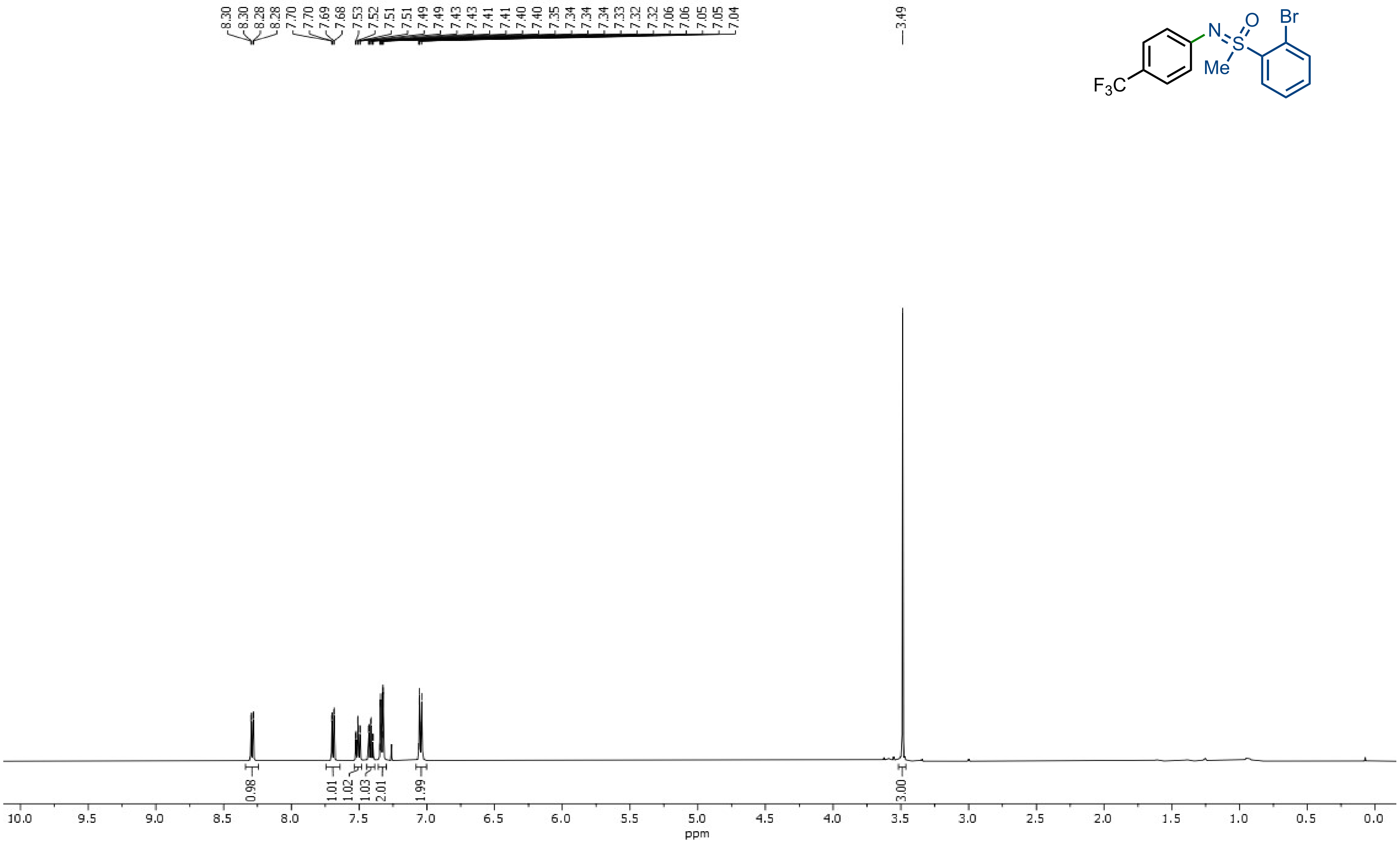

^{13}C NMR of (2-bromophenyl)(methyl)((4-(trifluoromethyl)phenyl)imino)-λ^6-sulfanone (28)

$CDCl_3$, 298 K

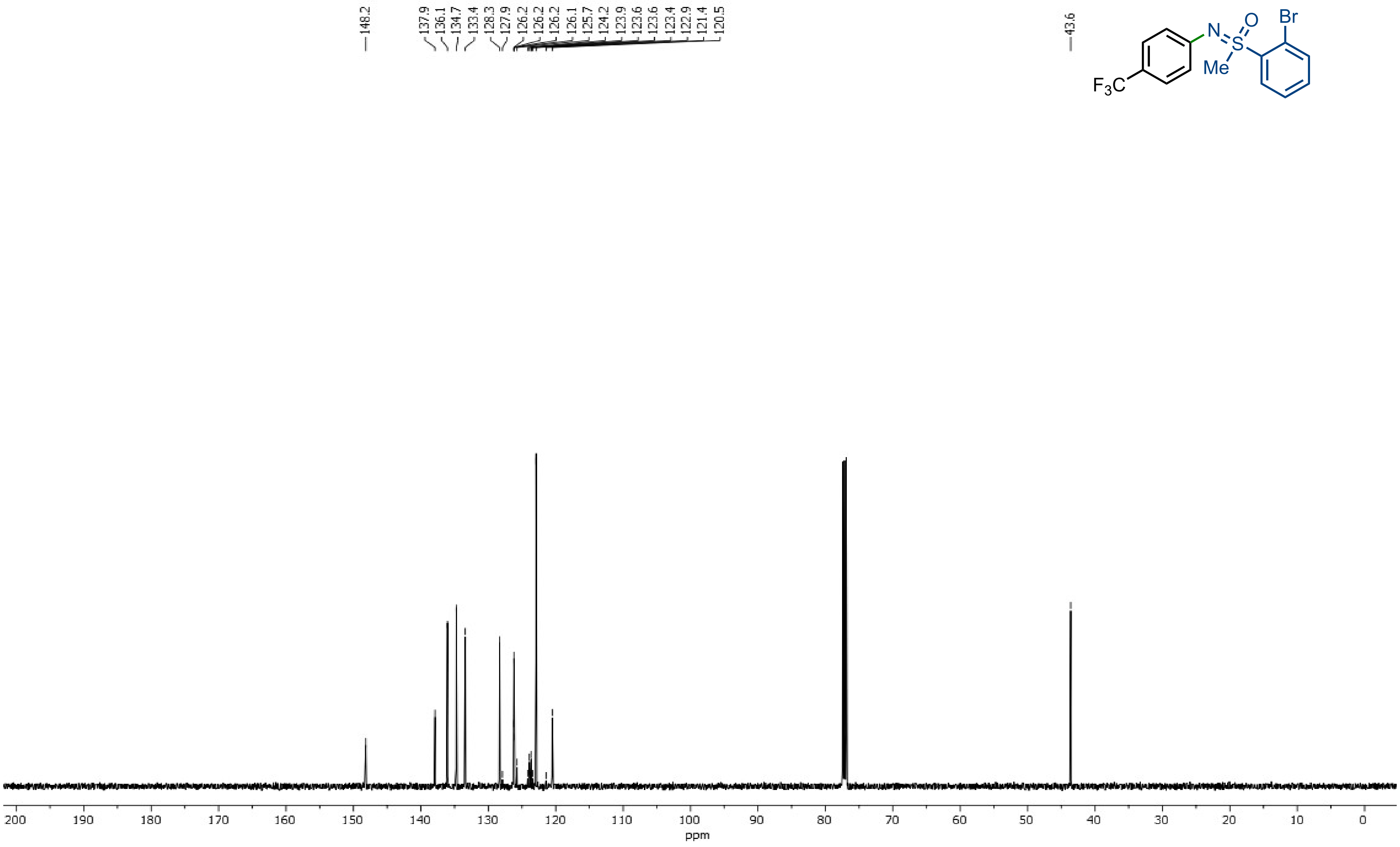

^{19}F NMR of (2-bromophenyl)(methyl)((4-(trifluoromethyl)phenyl)imino)-λ^{6}-sulfanone (28)

$CDCl_3$, 298 K

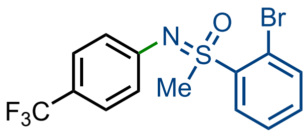

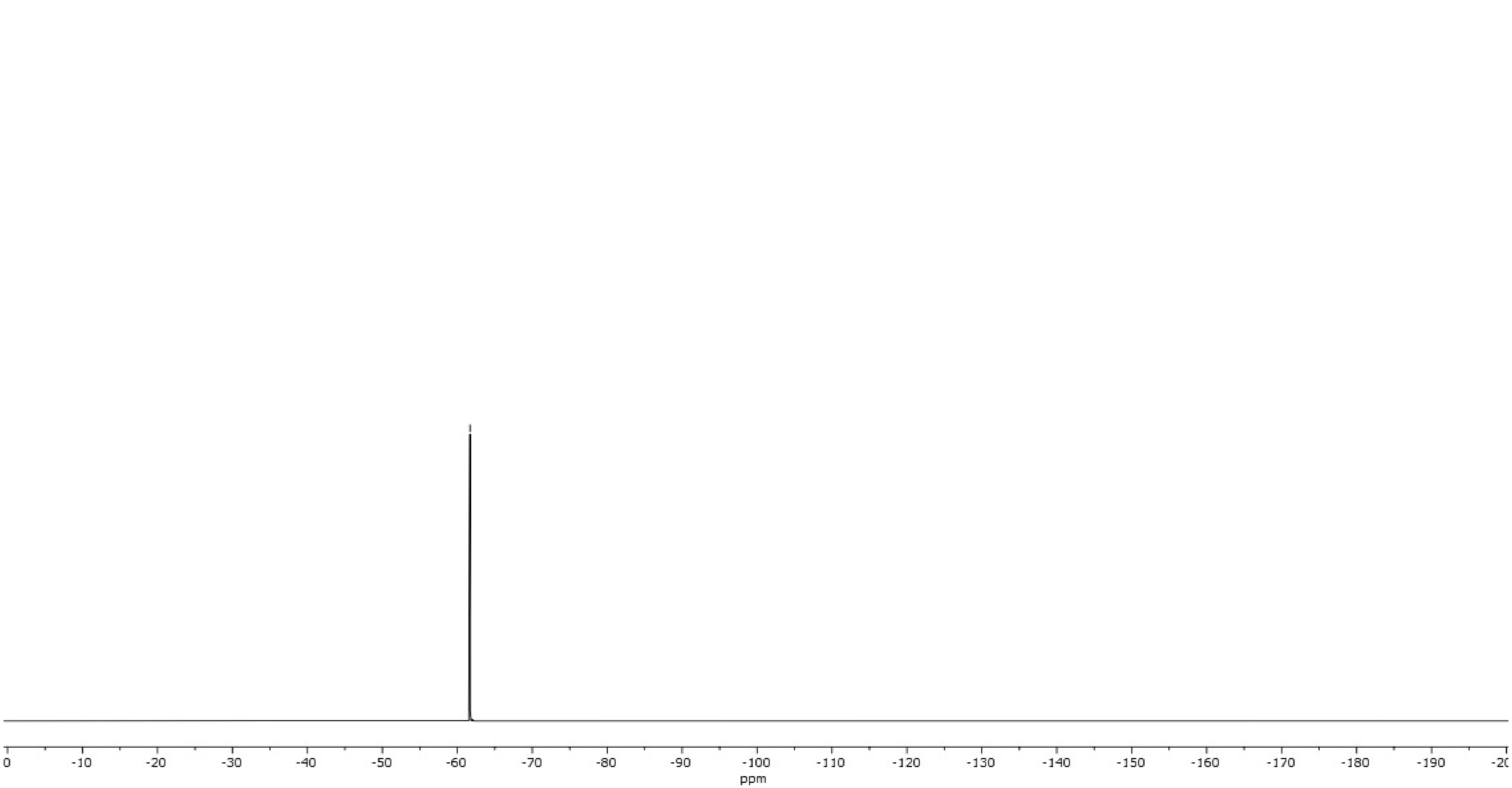

^{1}H NMR of (4-methoxyphenyl)(methyl)((4-(trifluoromethyl)phenyl)imino)-λ^{6}-sulfanone (29)

$CDCl_3$, 298 K

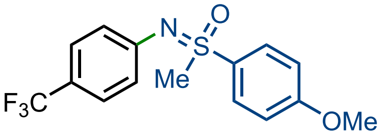

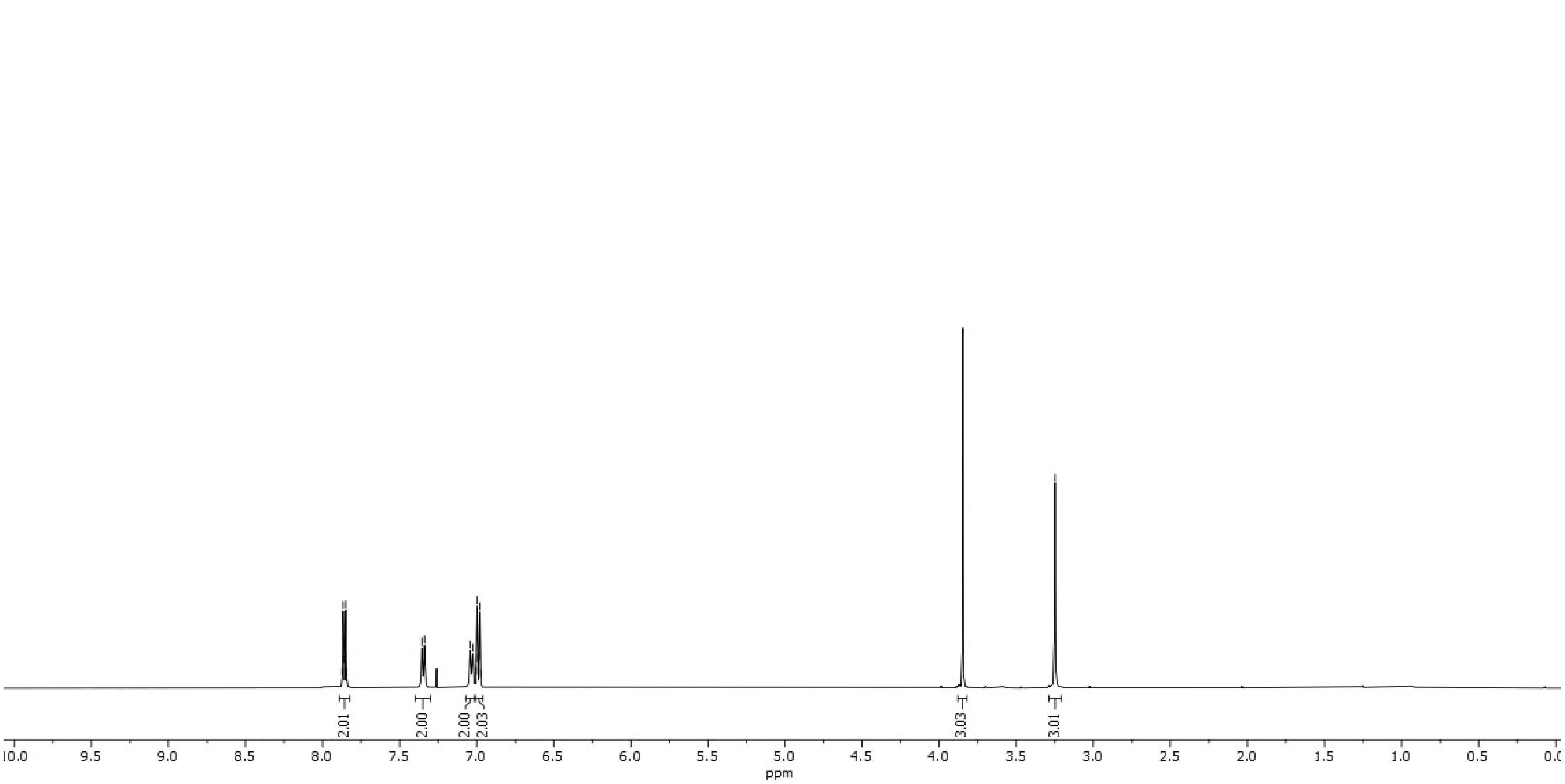

^{13}C NMR of (4-methoxyphenyl)(methyl)((4-(trifluoromethyl)phenyl)imino)-λ^{6}-sulfanone (29)

CDCl$_3$, 298 K

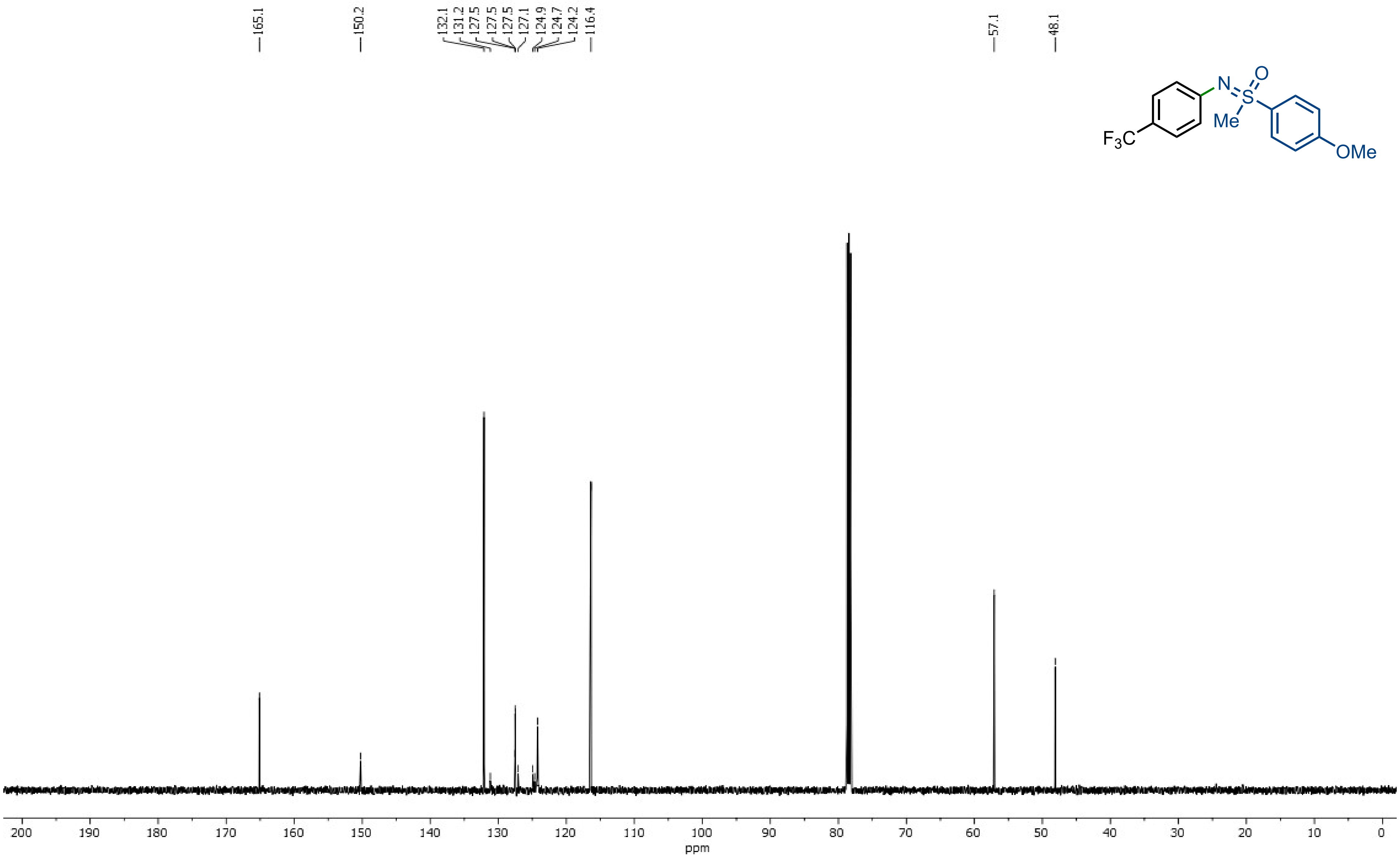

^{19}F NMR of (4-methoxyphenyl)(methyl)((4-(trifluoromethyl)phenyl)imino)-λ^{6}-sulfanone (29)

$CDCl_3$, 298 K

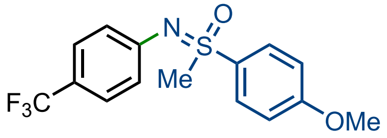

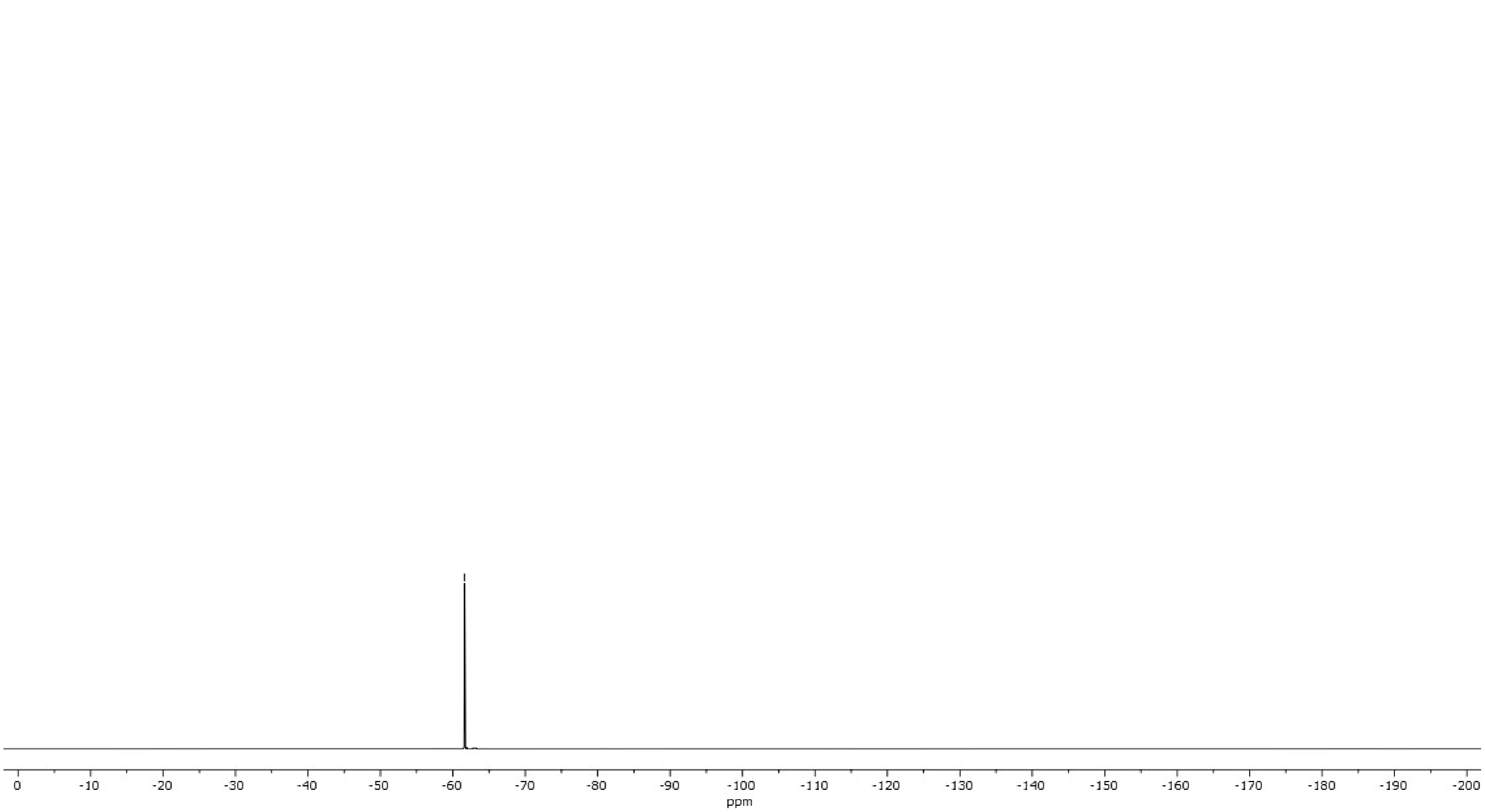

^{1}H NMR of (4-trifluoromethoxyphenyl)(methyl)((4-(trifluoromethyl)phenyl)imino)-λ^6-sulfanone (30)

$CDCl_3$, 298 K

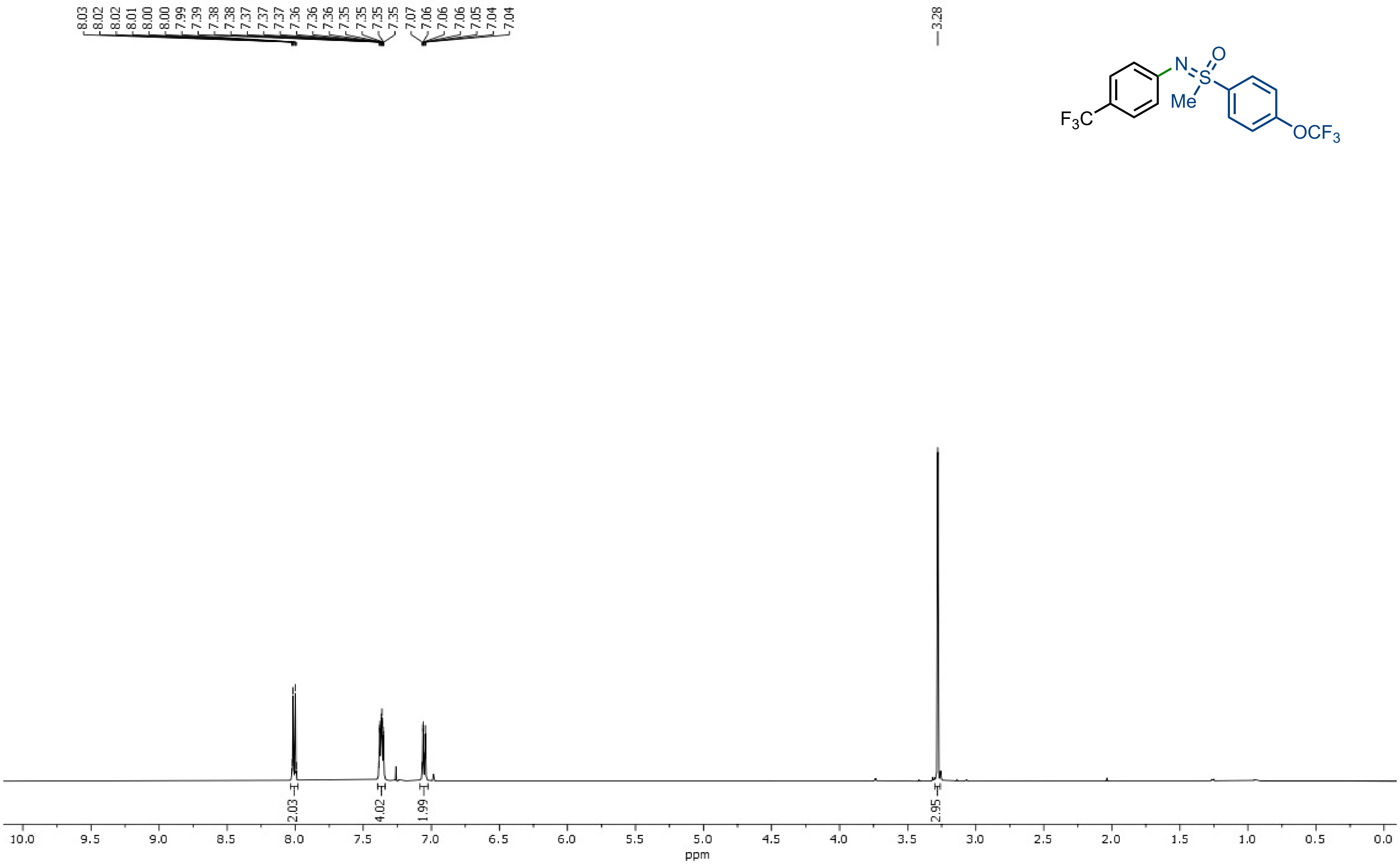

^{13}C NMR of (4-trifluoromethoxyphenyl)(methyl)((4-(trifluoromethyl)phenyl)imino)-λ^6-sulfanone (30)

CDCl$_3$, 298 K

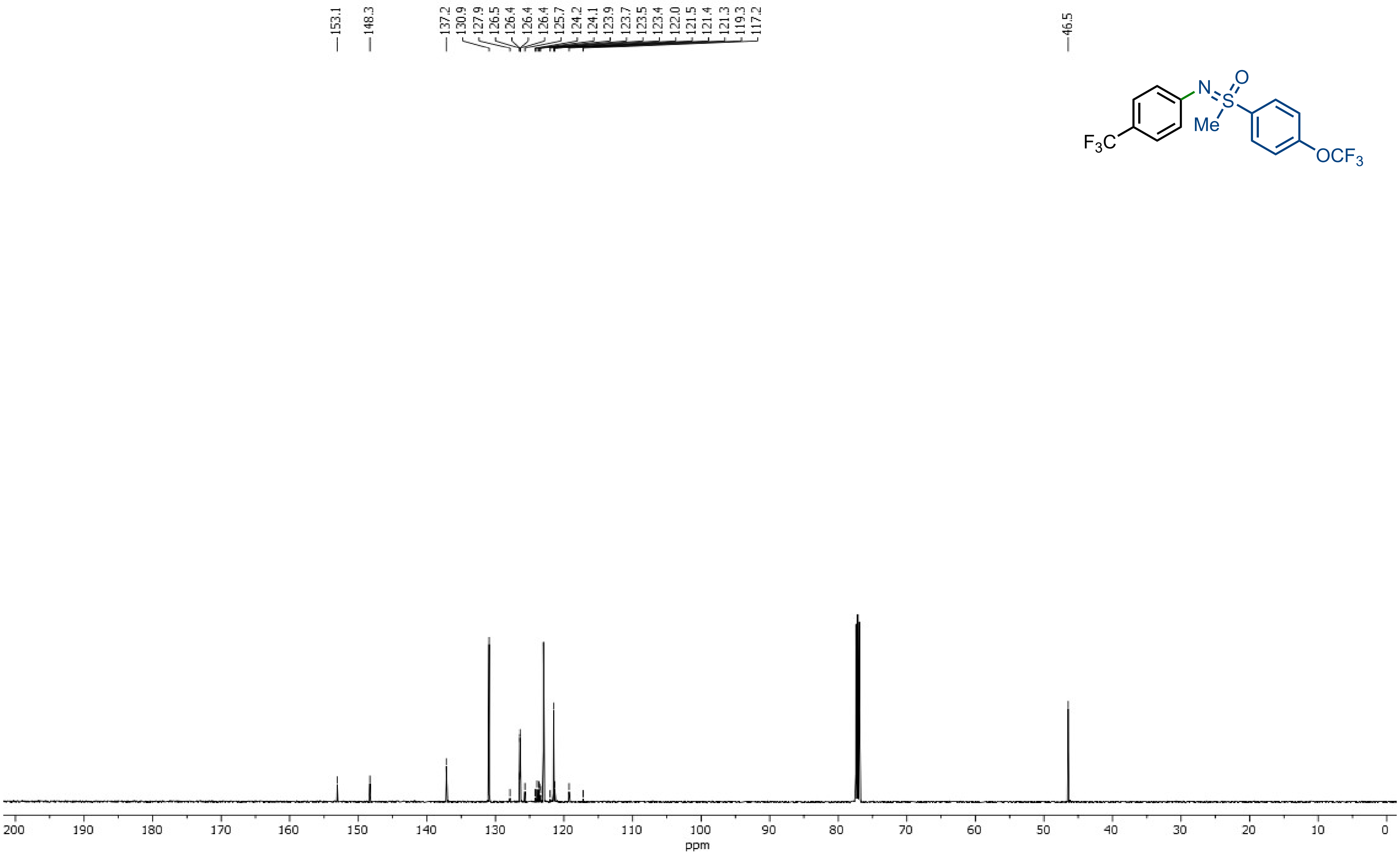

^{19}F NMR of (4-trifluoromethoxyphenyl)(methyl)((4-(trifluoromethyl)phenyl)imino)-λ^6-sulfanone (30)

$CDCl_3$, 298 K

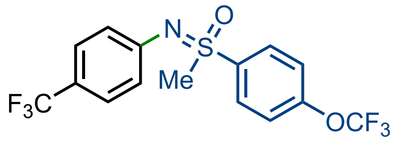

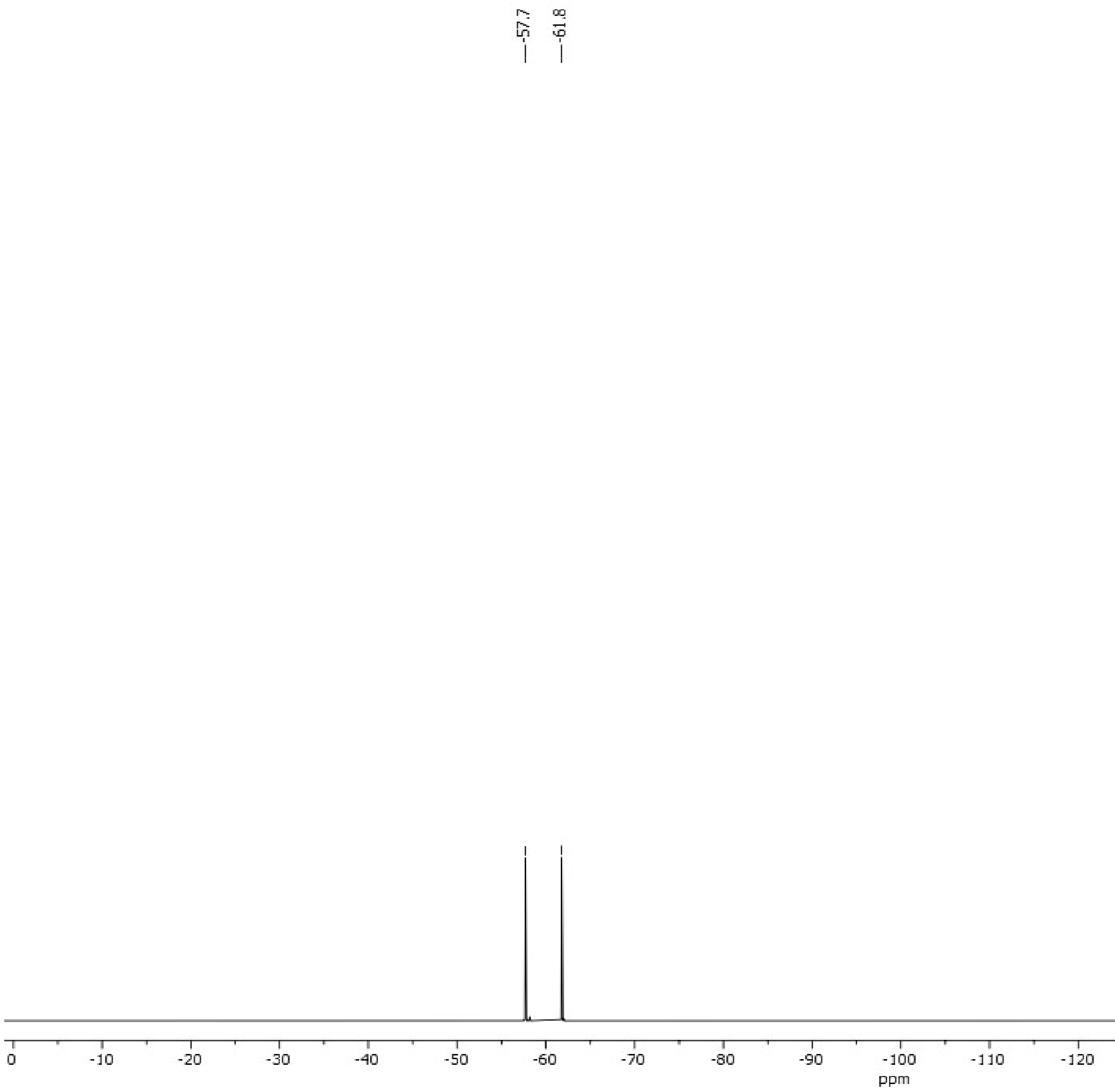

^{1}H NMR of dimethyl((4-(trifluoromethyl)phenyl)imino)-λ^6-sulfanone (31)

$CDCl_3$, 298 K

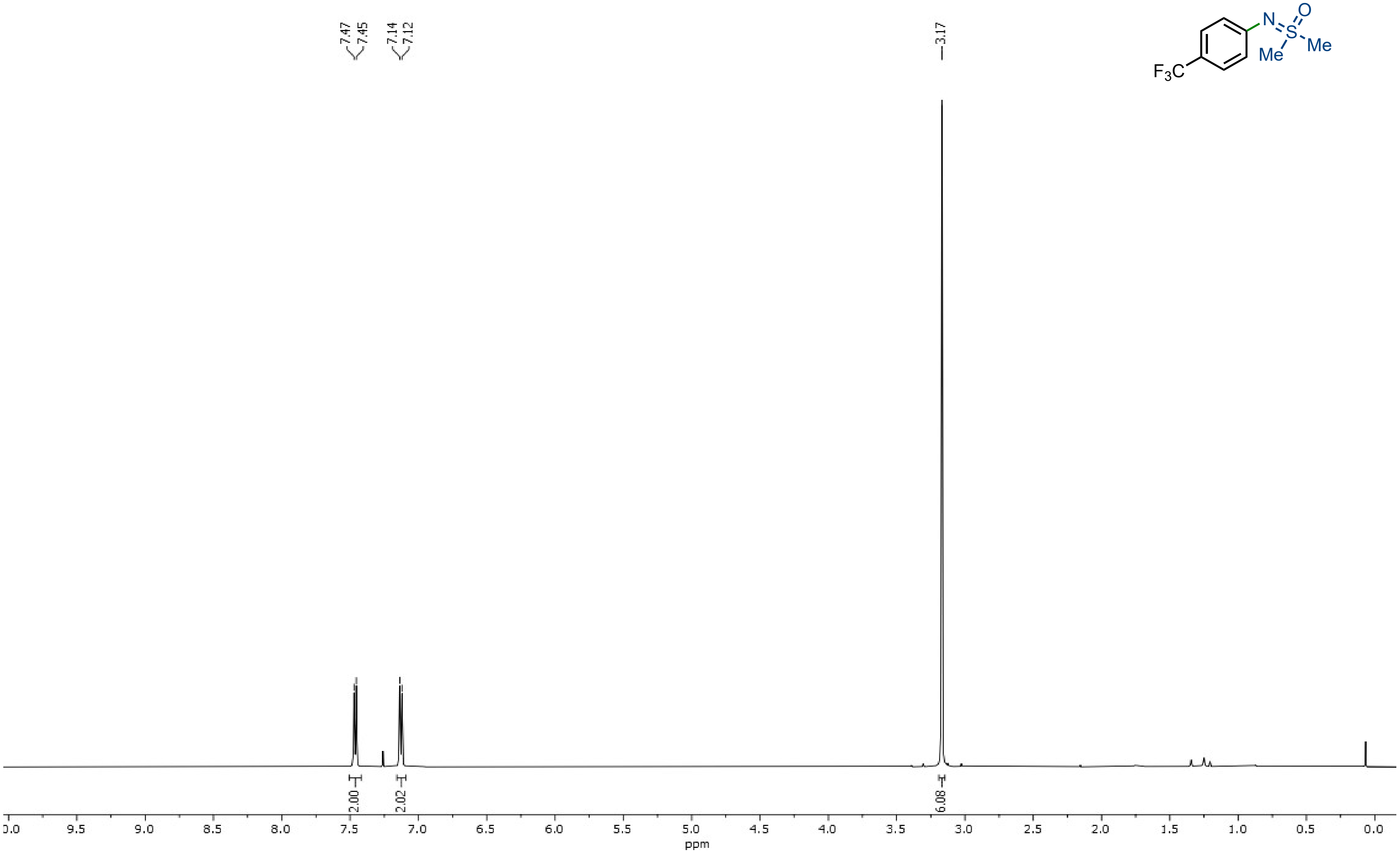

^{13}C NMR of dimethyl((4-(trifluoromethyl)phenyl)imino)-λ^6-sulfanone (31)

$CDCl_3$, 298 K

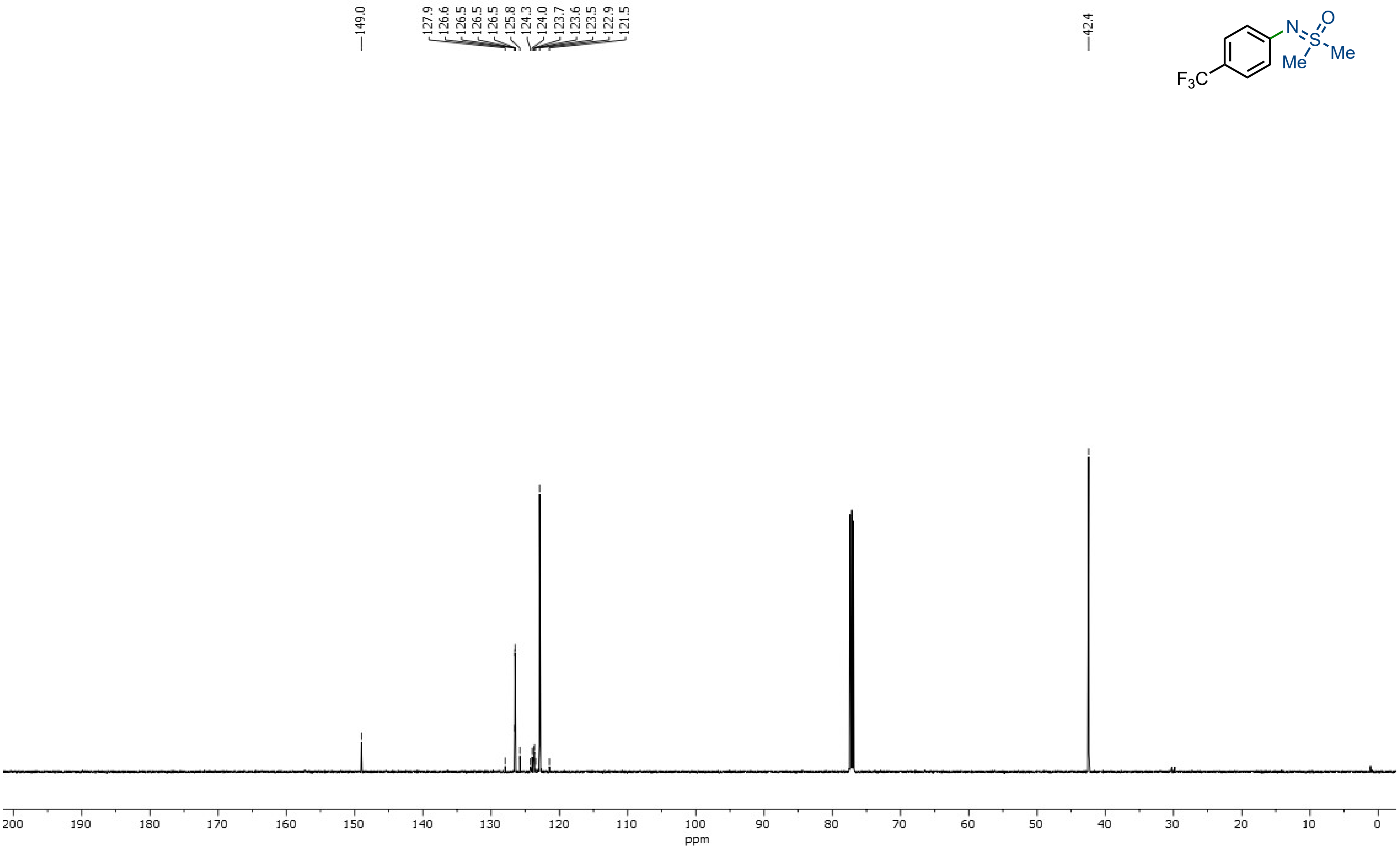

^{19}F NMR of dimethyl((4-(trifluoromethyl)phenyl)imino)-λ^{6}-sulfanone (31)

$CDCl_3$, 298 K

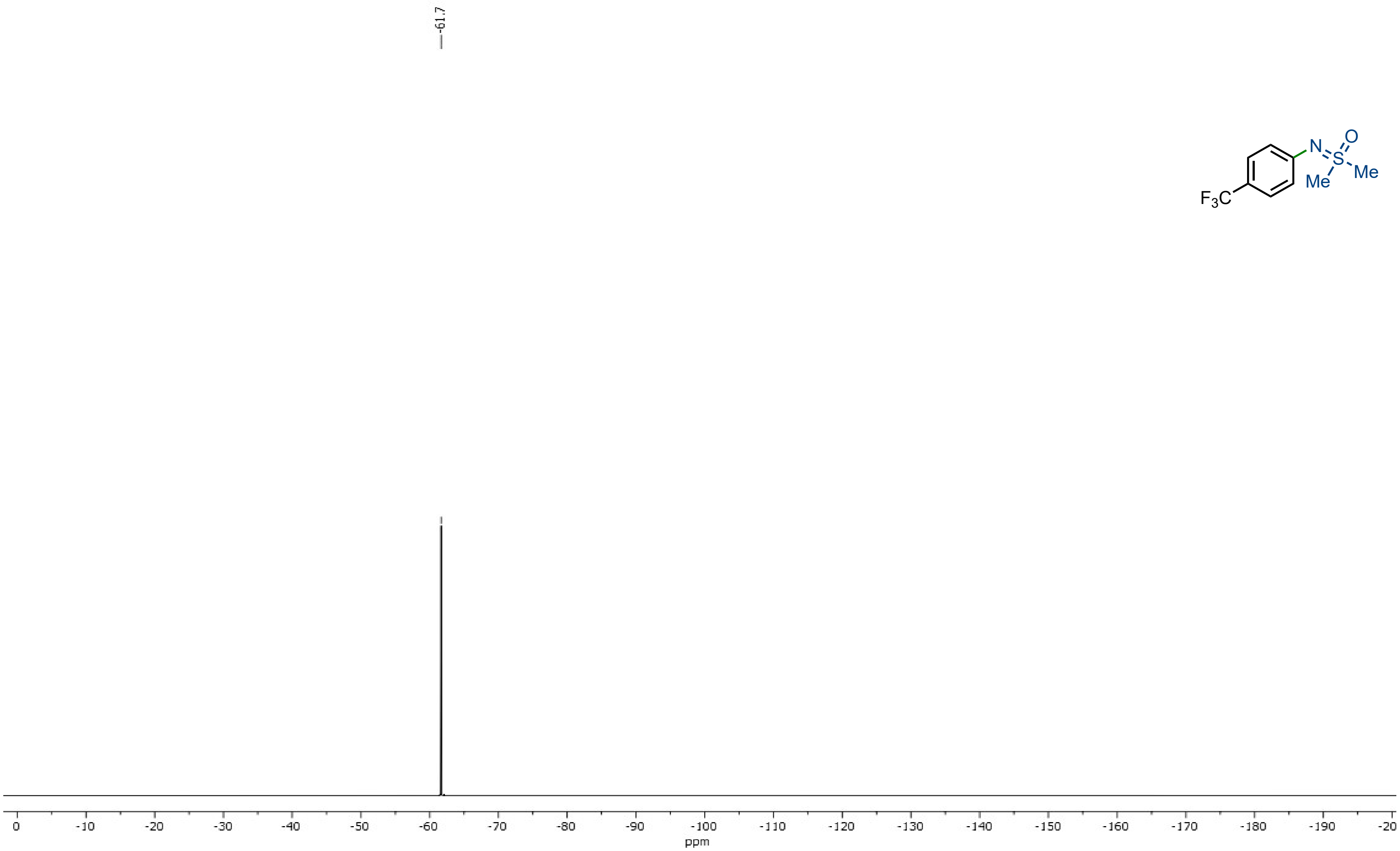

^{1}H NMR of diphenyl((4-(trifluoromethyl)phenyl)imino)-λ^6-sulfanone (32)

$CDCl_3$, 298 K

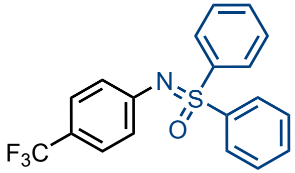

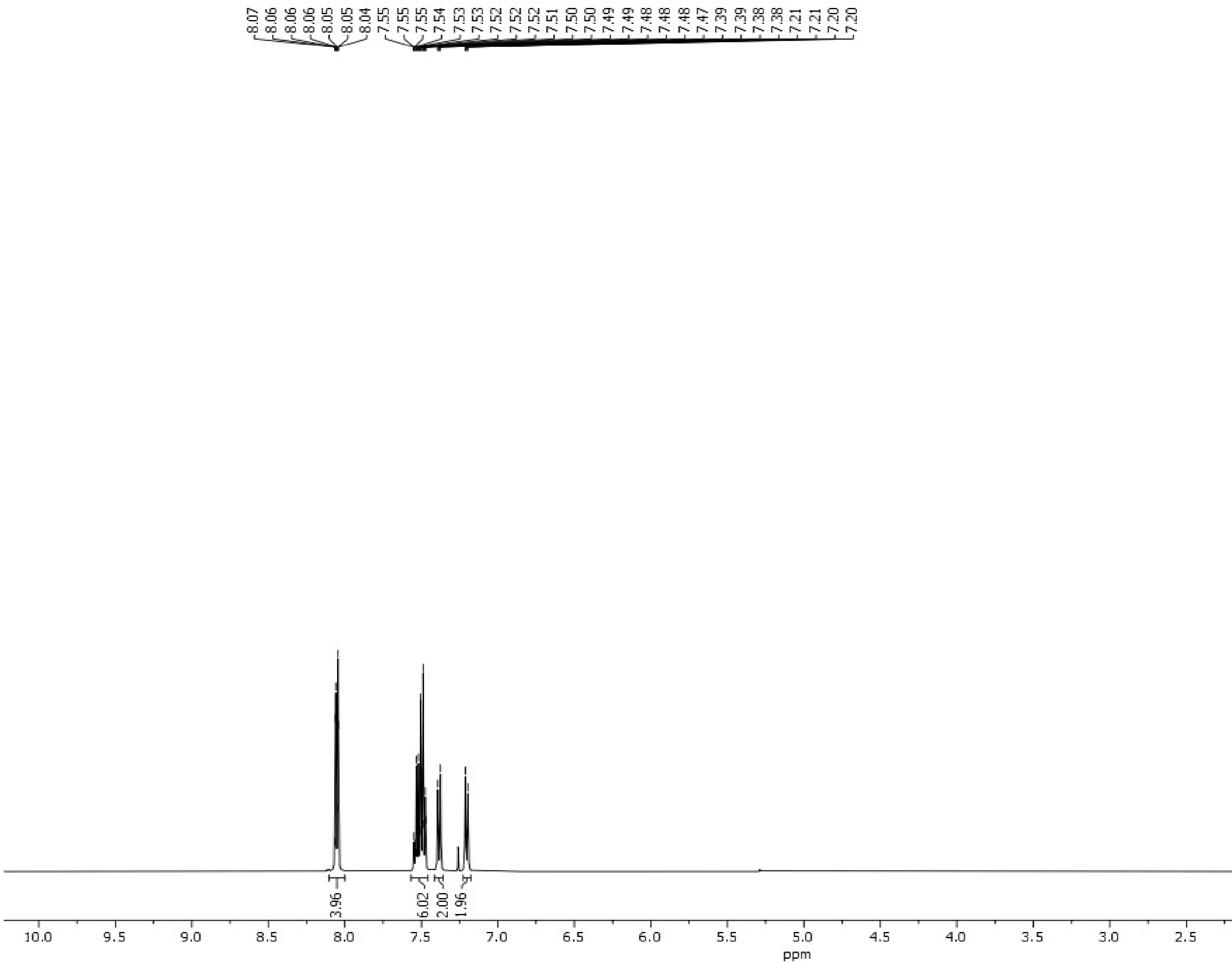

^{13}C NMR of diphenyl((4-(trifluoromethyl)phenyl)imino)-λ^6-sulfanone (32)

$CDCl_3$, 298 K

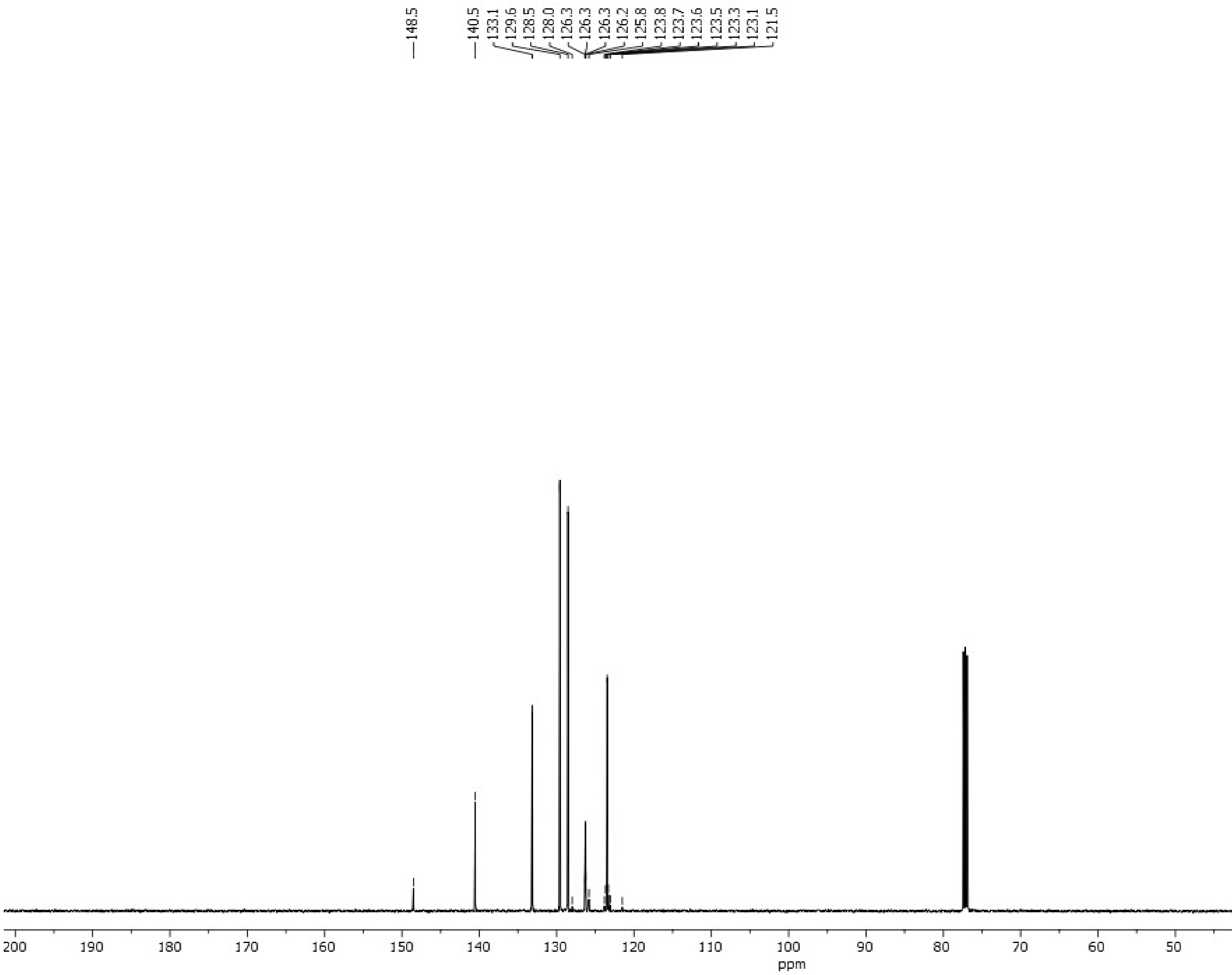

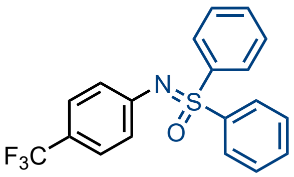

^{19}F NMR of diphenyl((4-(trifluoromethyl)phenyl)imino)-λ^6-sulfanone (32)

$CDCl_3$, 298 K

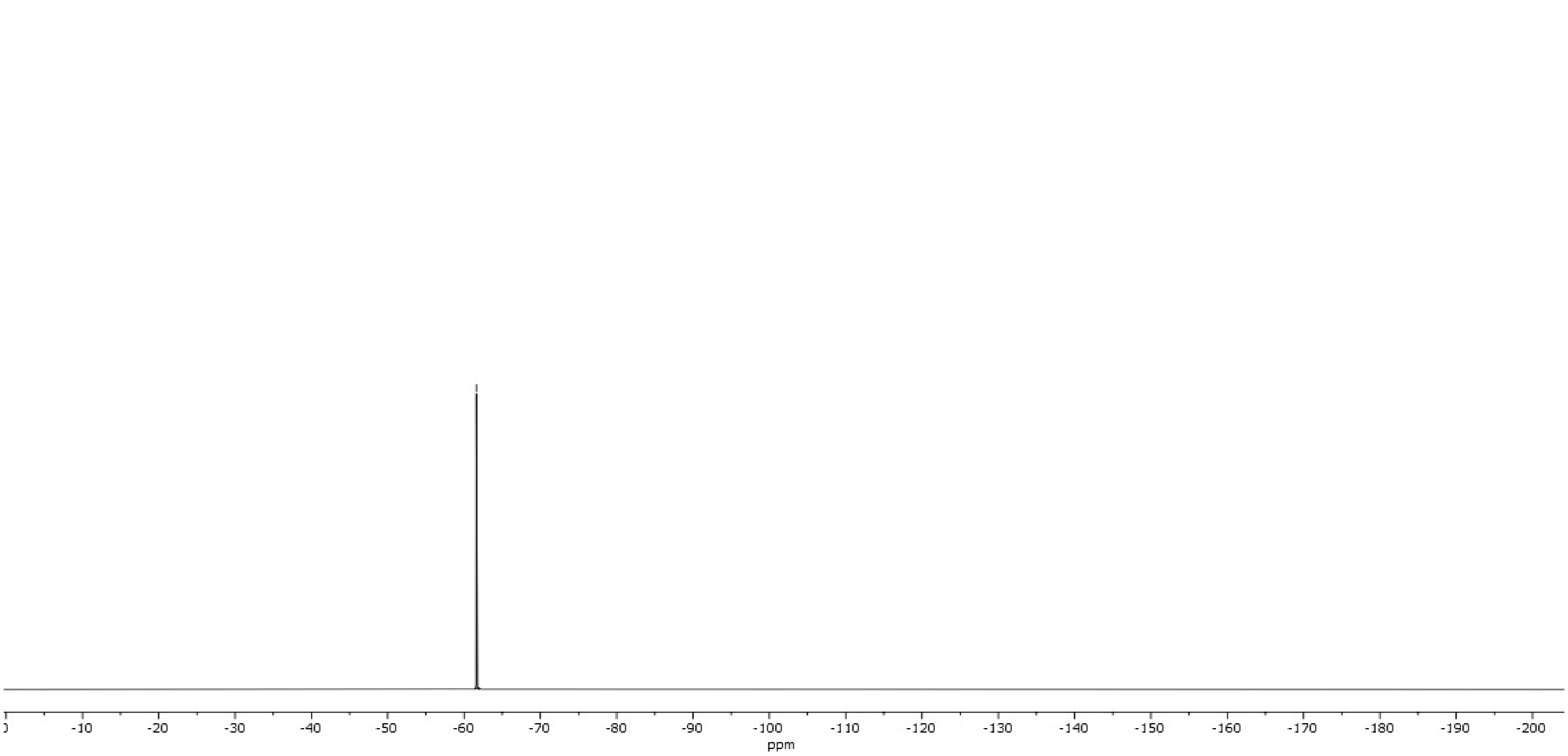

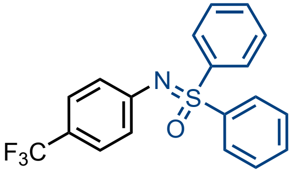

^{1}H NMR of phenyl-4-*d* 4-methylbenzenesulfonate (34)

CD_2Cl_2, 298 K

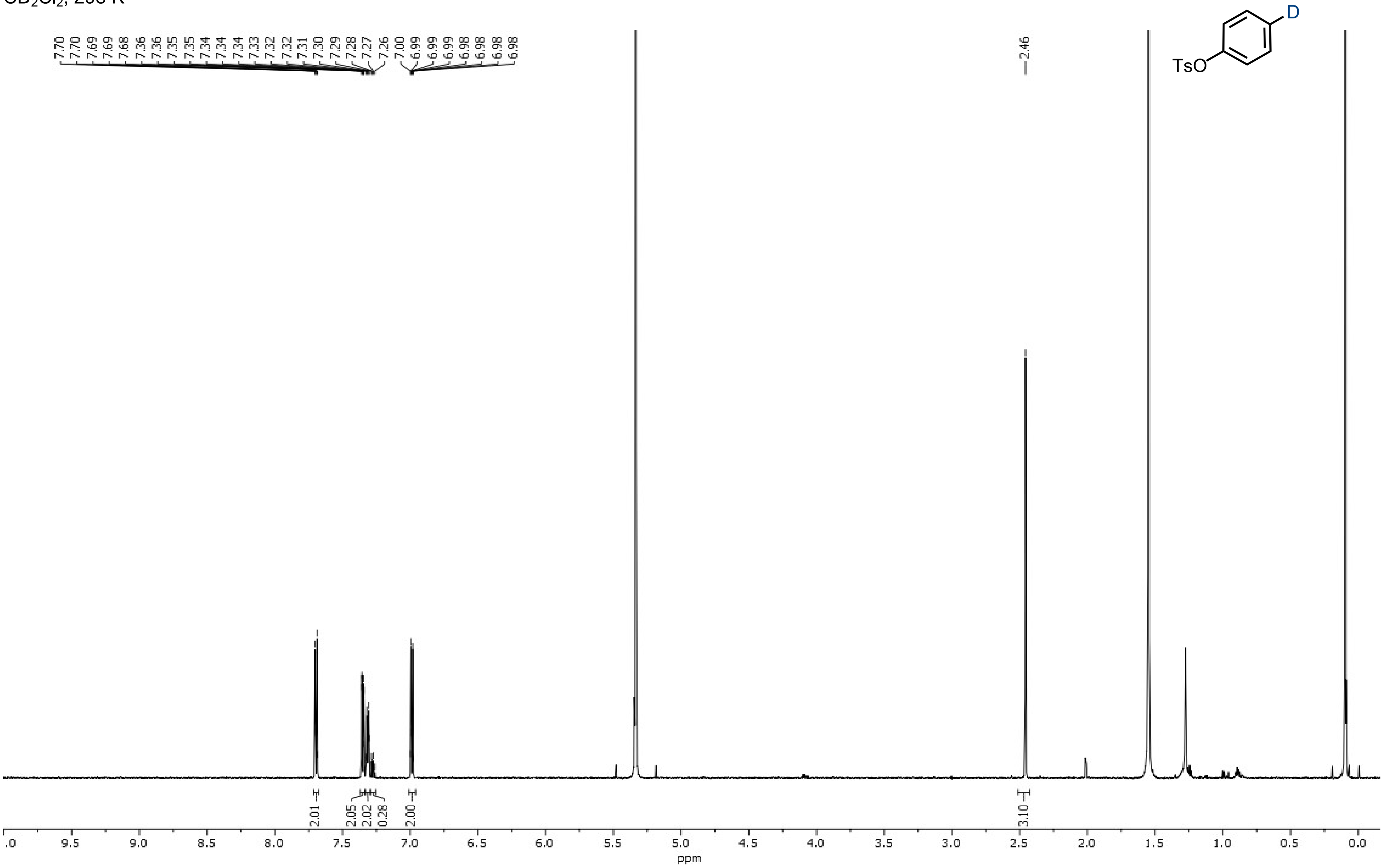

D NMR of phenyl-4-*d* 4-methylbenzenesulfonate (34)

CH_2Cl_2, 298 K

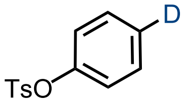

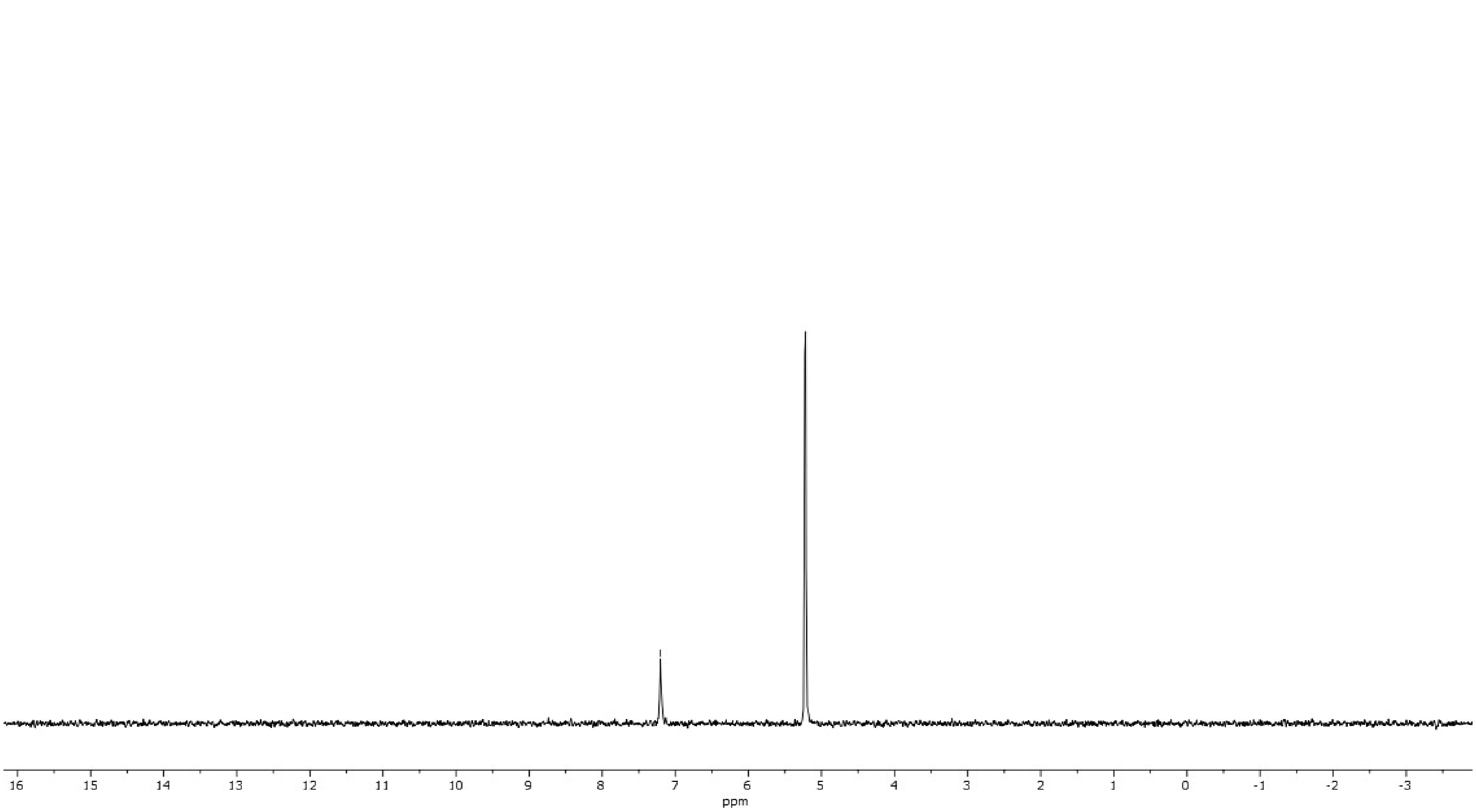

^{13}C NMR of phenyl-4-*d* 4-methylbenzenesulfonate (34)

CD_2Cl_2, 298 K

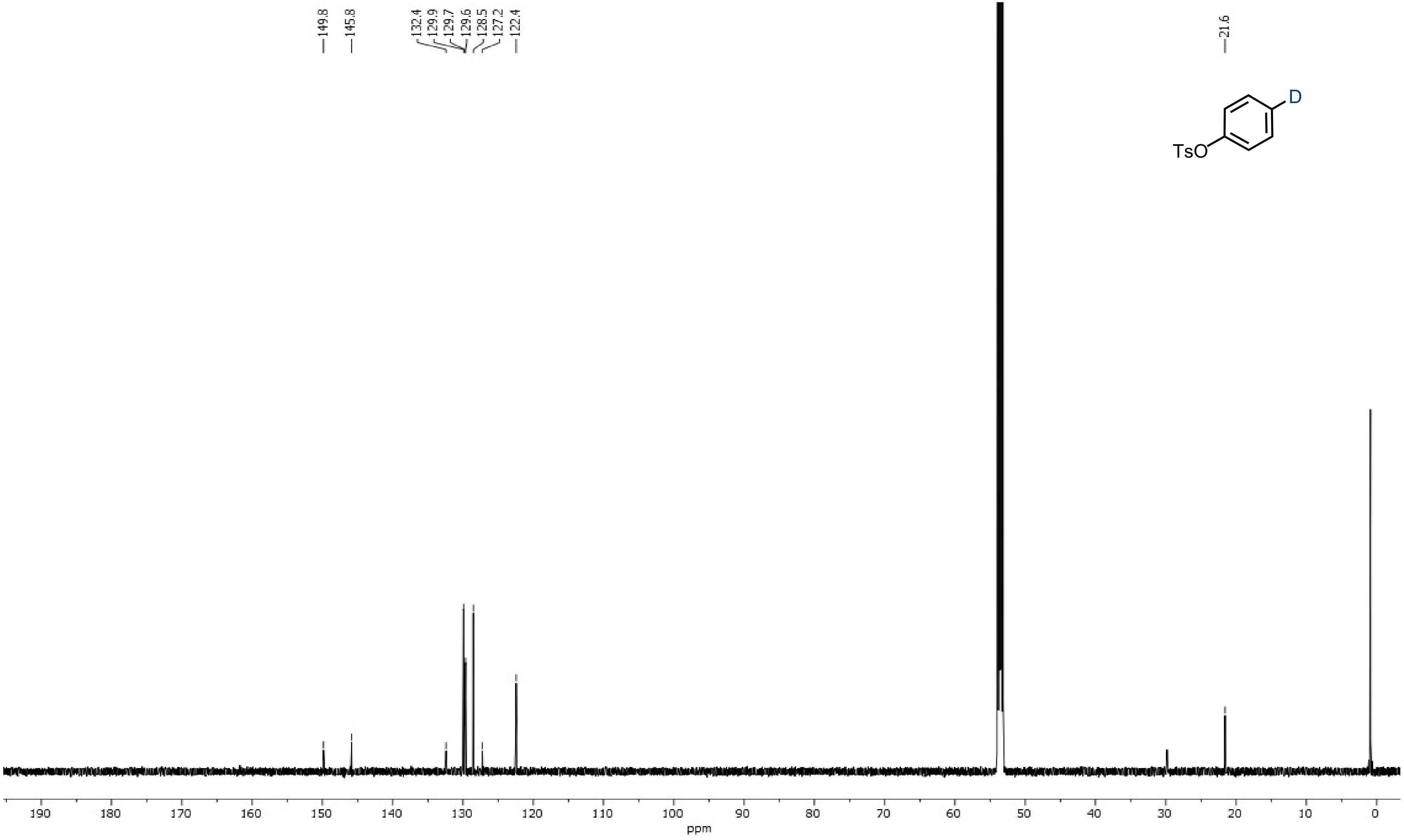

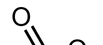

^{1}H NMR of 2-(4-fluorophenyl)benzo[*d*]isothiazol-3(2*H*)-one 1,1-dioxide (36)

$CDCl_3$, 298 K

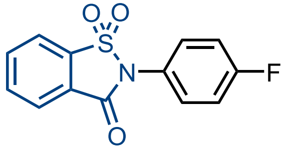

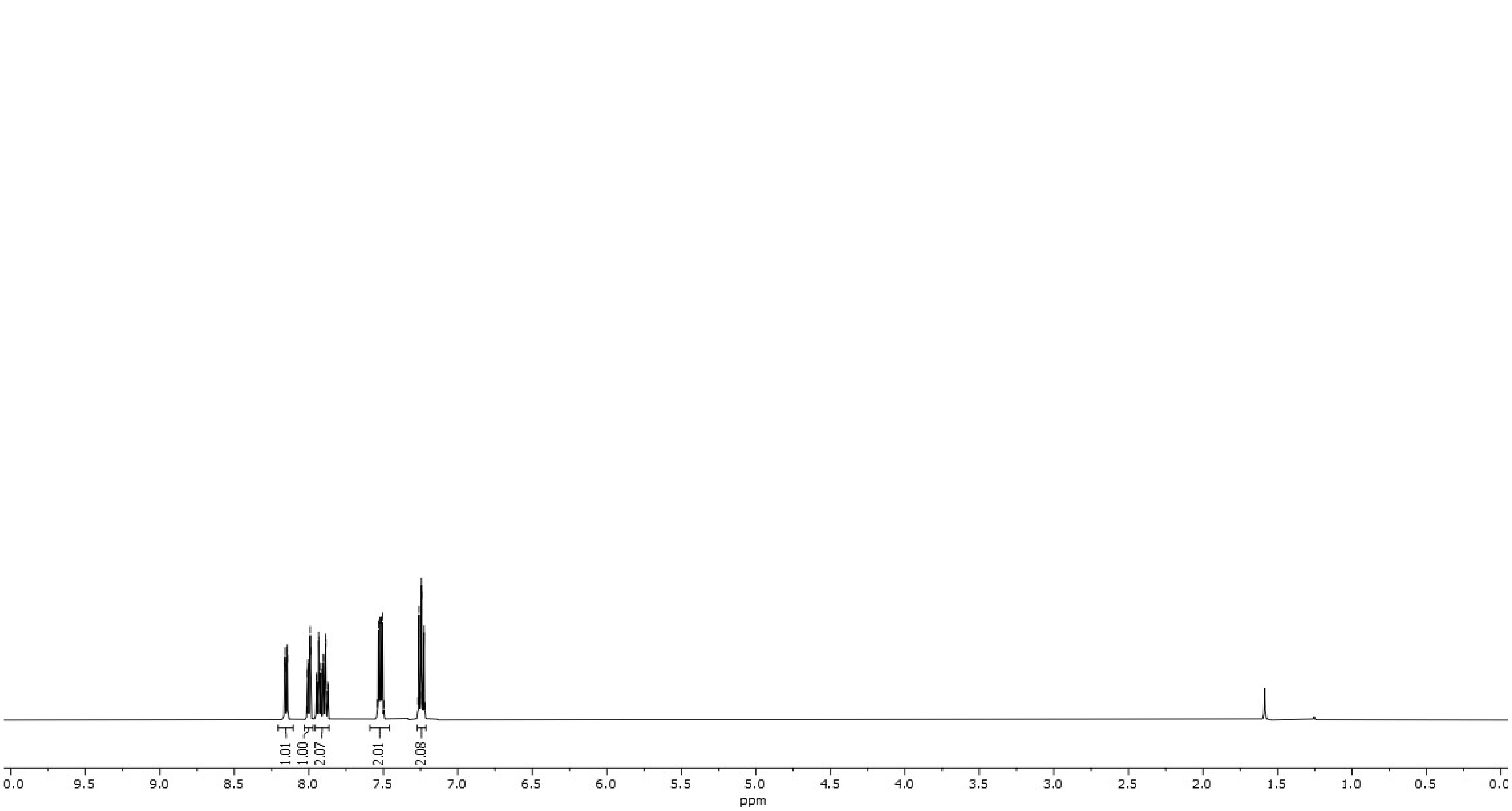

^{13}C NMR of 2-(4-fluorophenyl)benzo[*d*]isothiazol-3(2*H*)-one 1,1-dioxide (36)

$CDCl_3$, 298 K

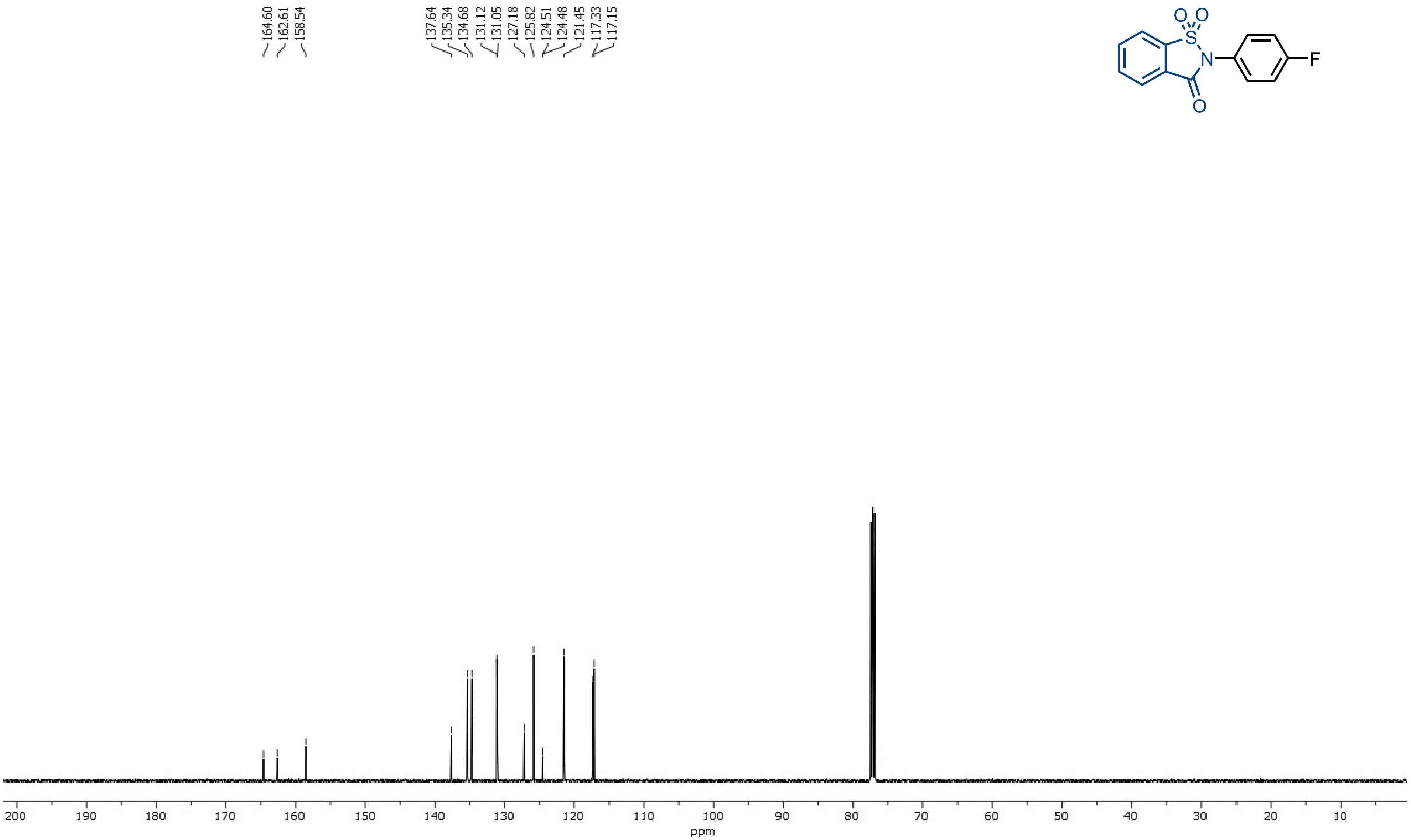

^{19}F NMR of 2-(4-fluorophenyl)benzo[*d*]isothiazol-3(2*H*)-one 1,1-dioxide (36)

$CDCl_3$, 298 K

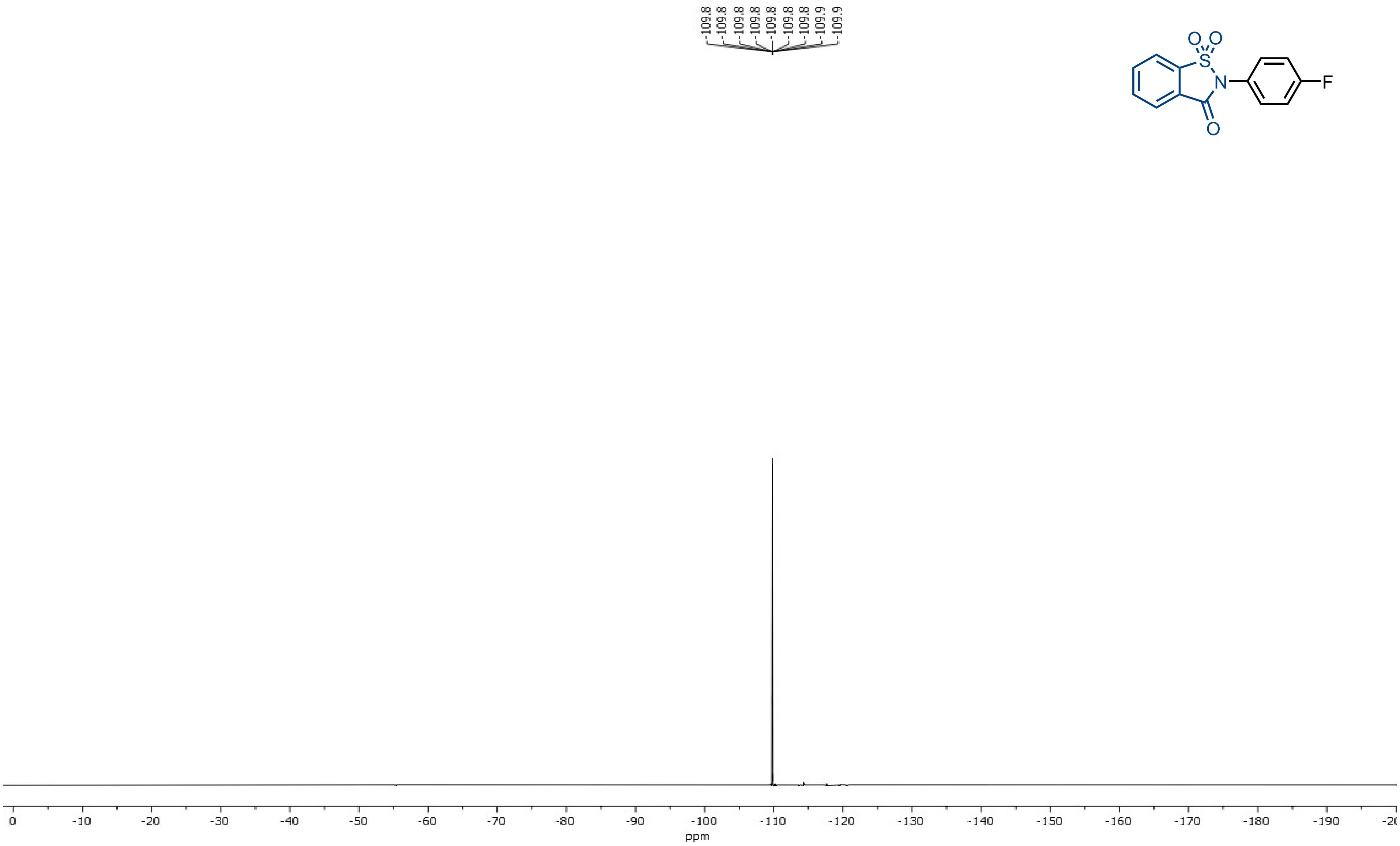

^{1}H NMR of phenyl 3-methoxybenzoate (S1)

$CDCl_3$, 298 K

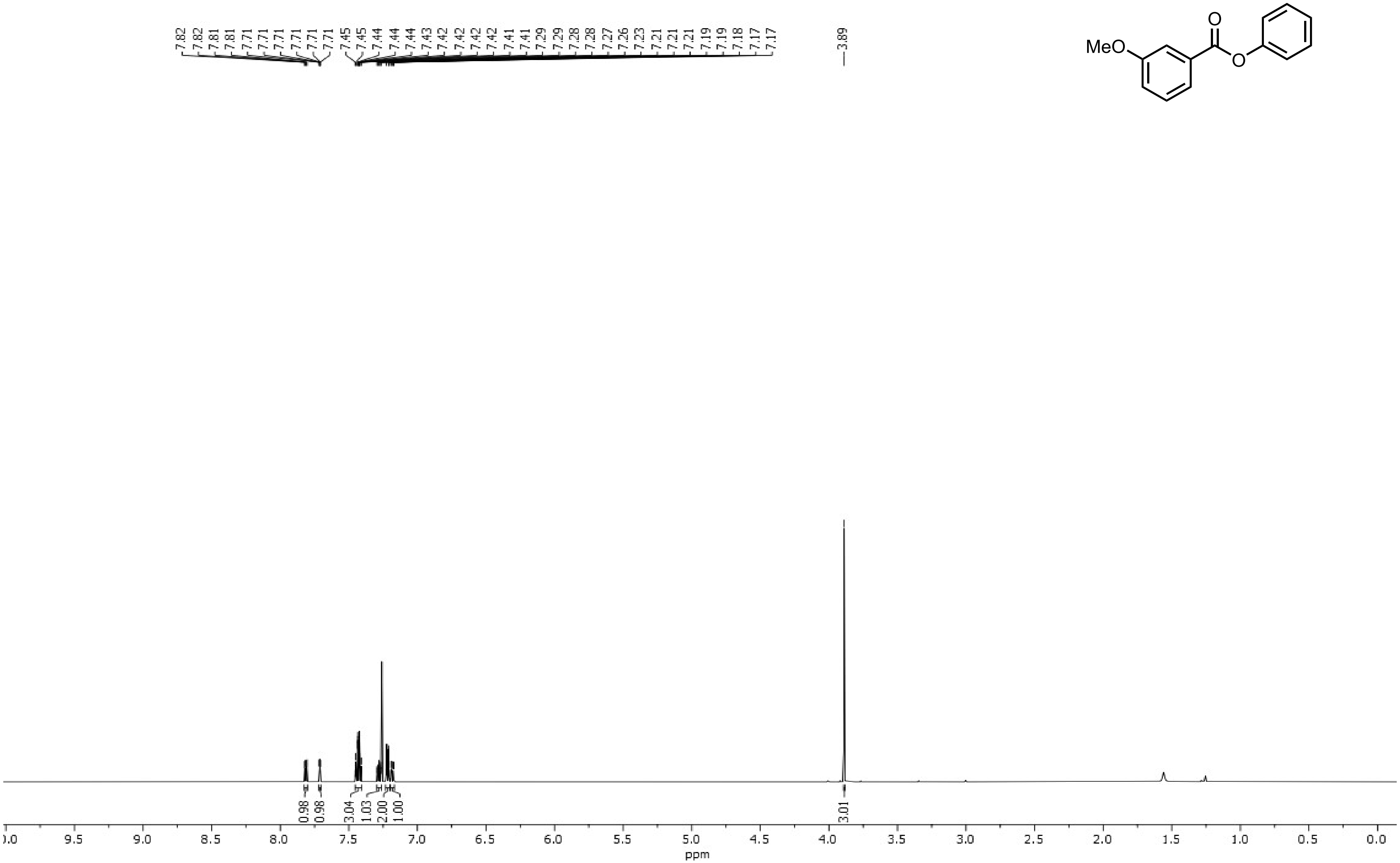

^{13}C NMR of phenyl 3-methoxybenzoate (S1)

$CDCl_3$, 298 K

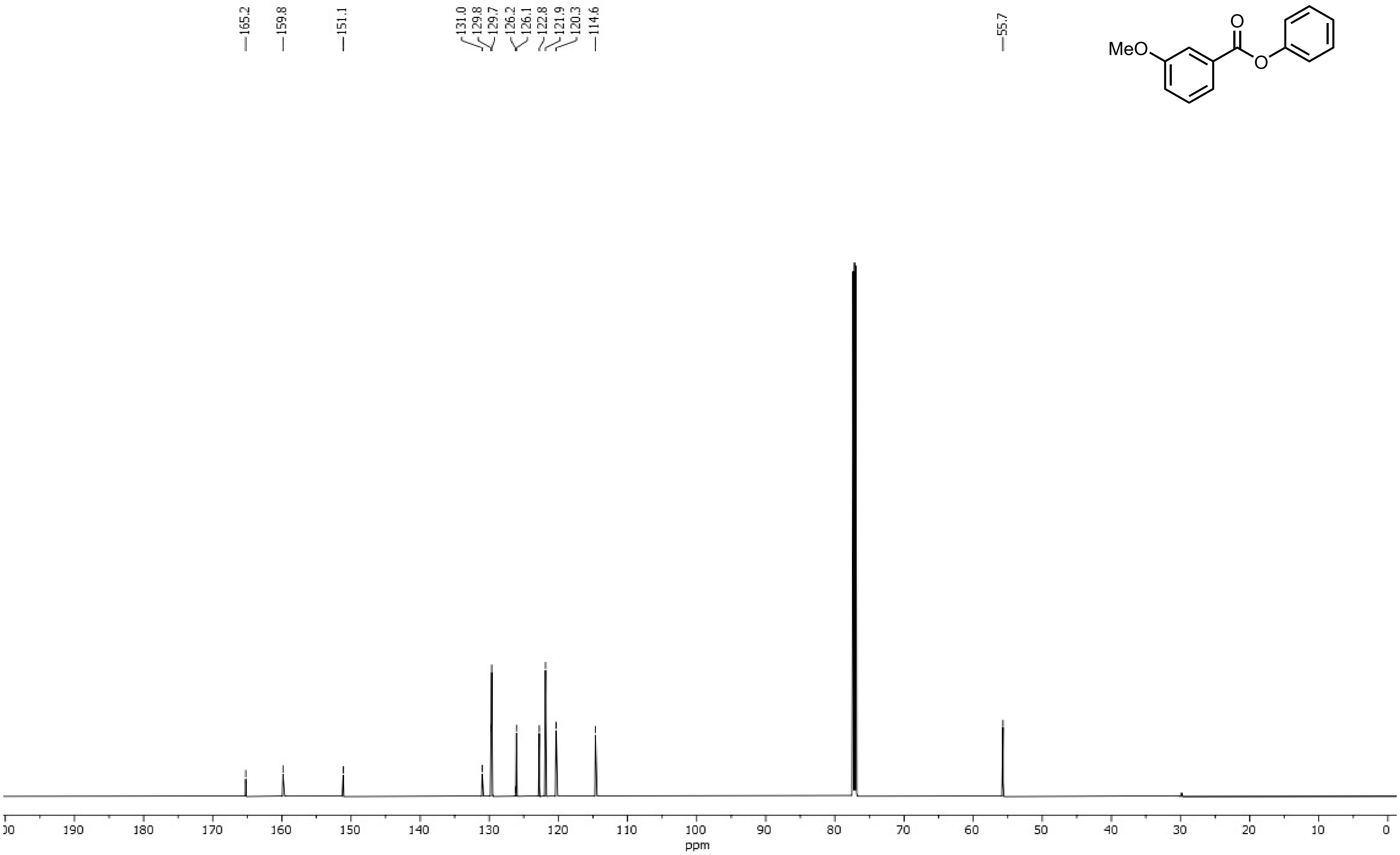

^{1}H NMR of 3-methoxy-1,1'-biphenyl (S2)

$CDCl_3$, 298 K

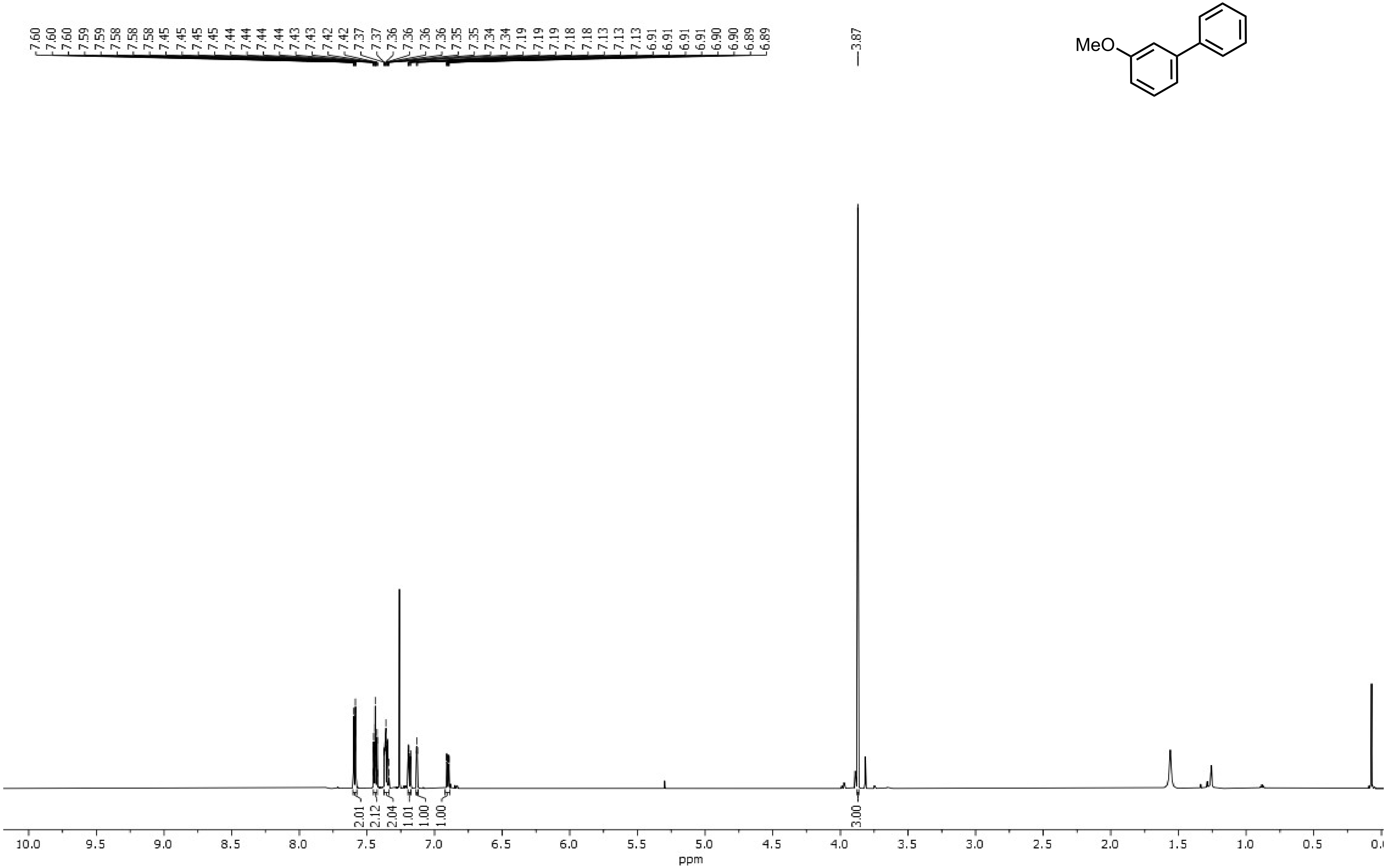

^{13}C NMR of 3-methoxy-1,1'-biphenyl (S2)

$CDCl_3$, 298 K

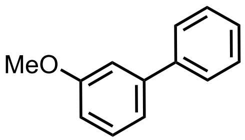

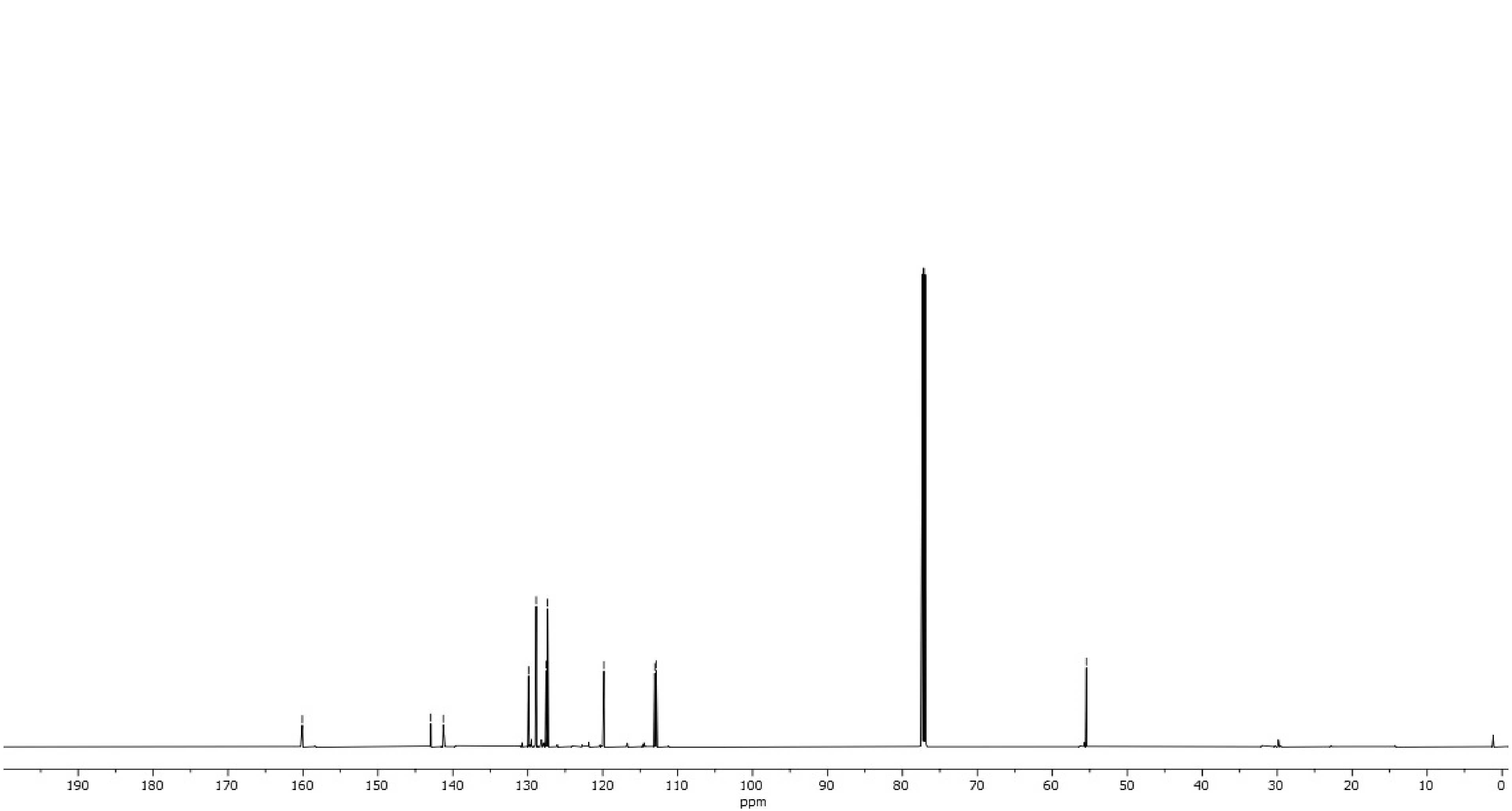

^{1}H NMR of (4-fluorophenyl)(methyl)(phenylimino)-λ^6-sulfanone (S3)

$CDCl_3$, 298 K

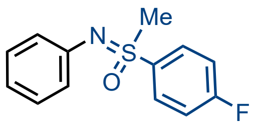

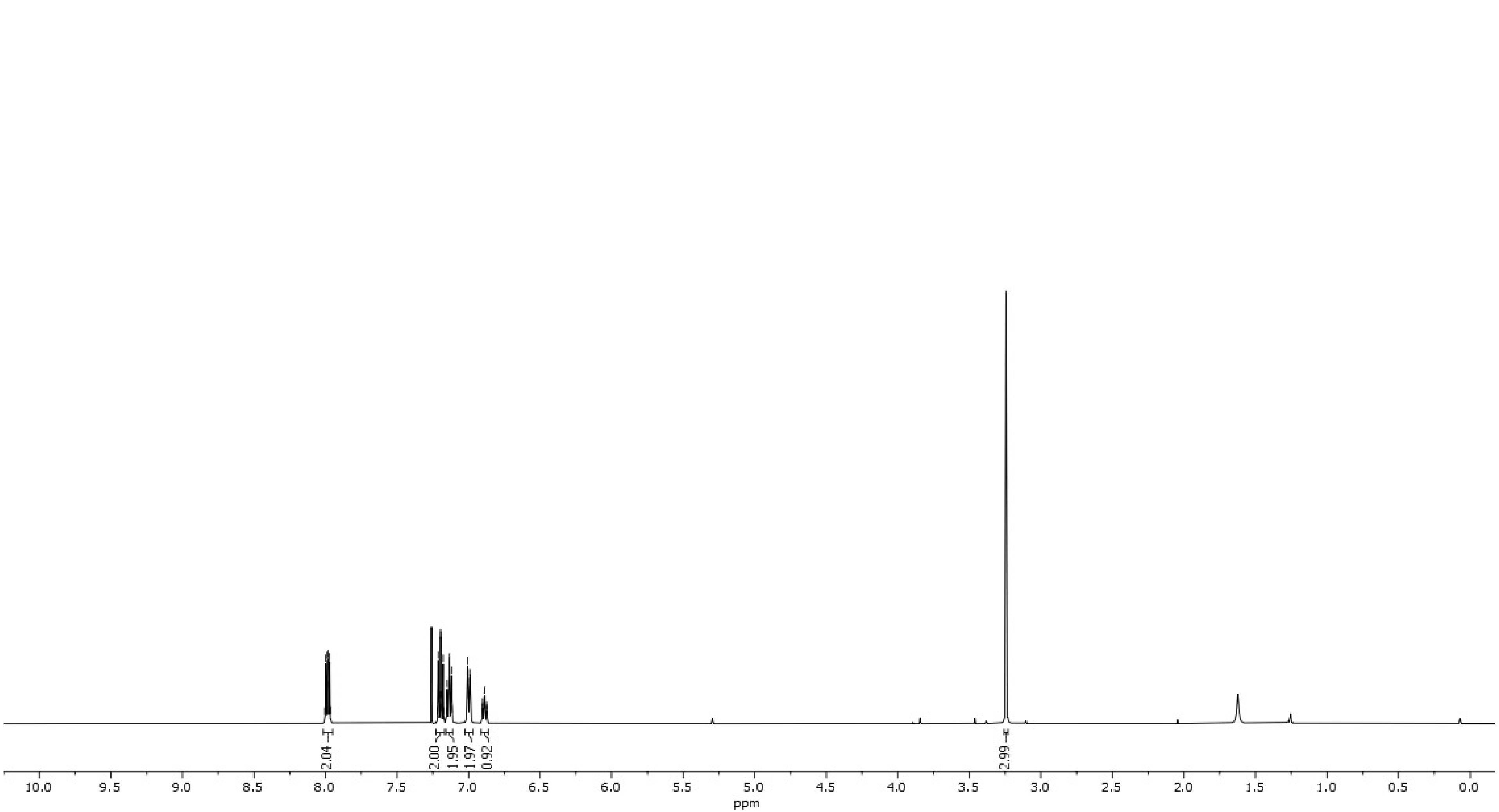

^{13}C NMR of (4-fluorophenyl)(methyl)(phenylimino)-λ^6-sulfanone (S3)

$CDCl_3$, 298 K

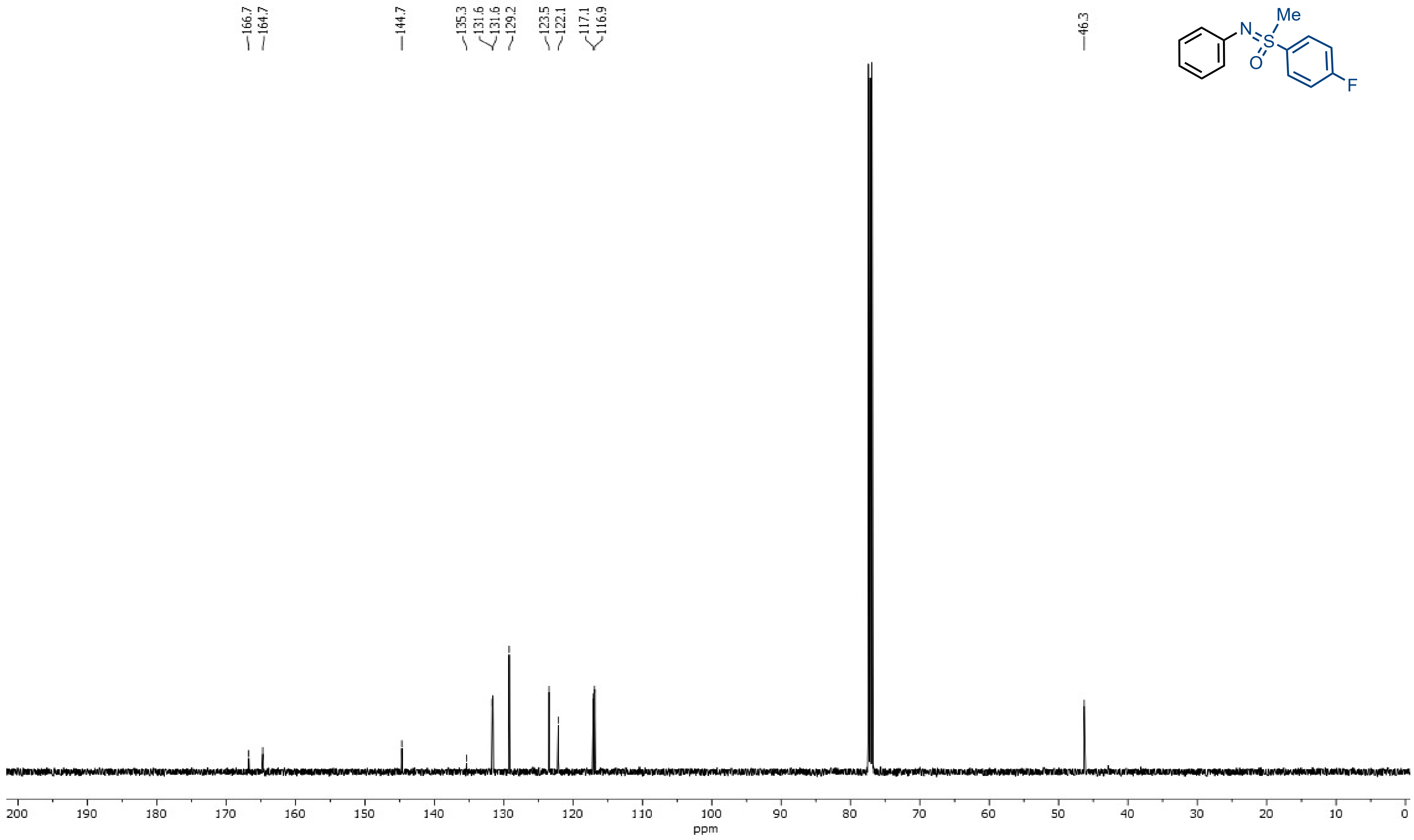

^{19}F NMR of (4-fluorophenyl)(methyl)(phenylimino)-λ^6-sulfanone (S3)

$CDCl_3$, 298 K

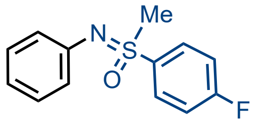

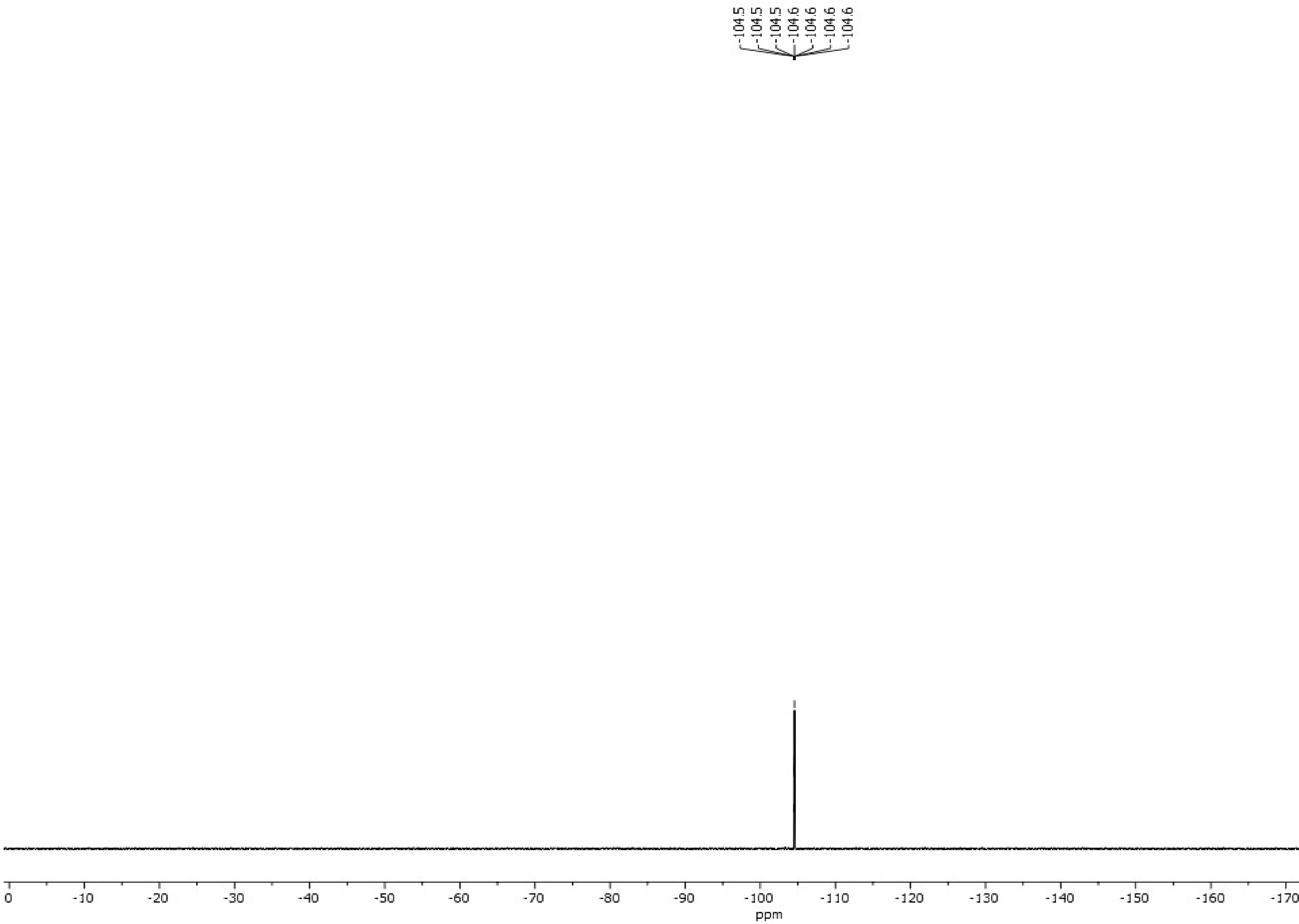

^{1}H NMR of 6*H*-benzo[c]chromen-6-one (S4)

CDCl$_3$, 298 K

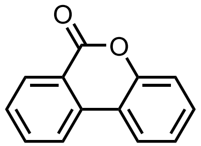

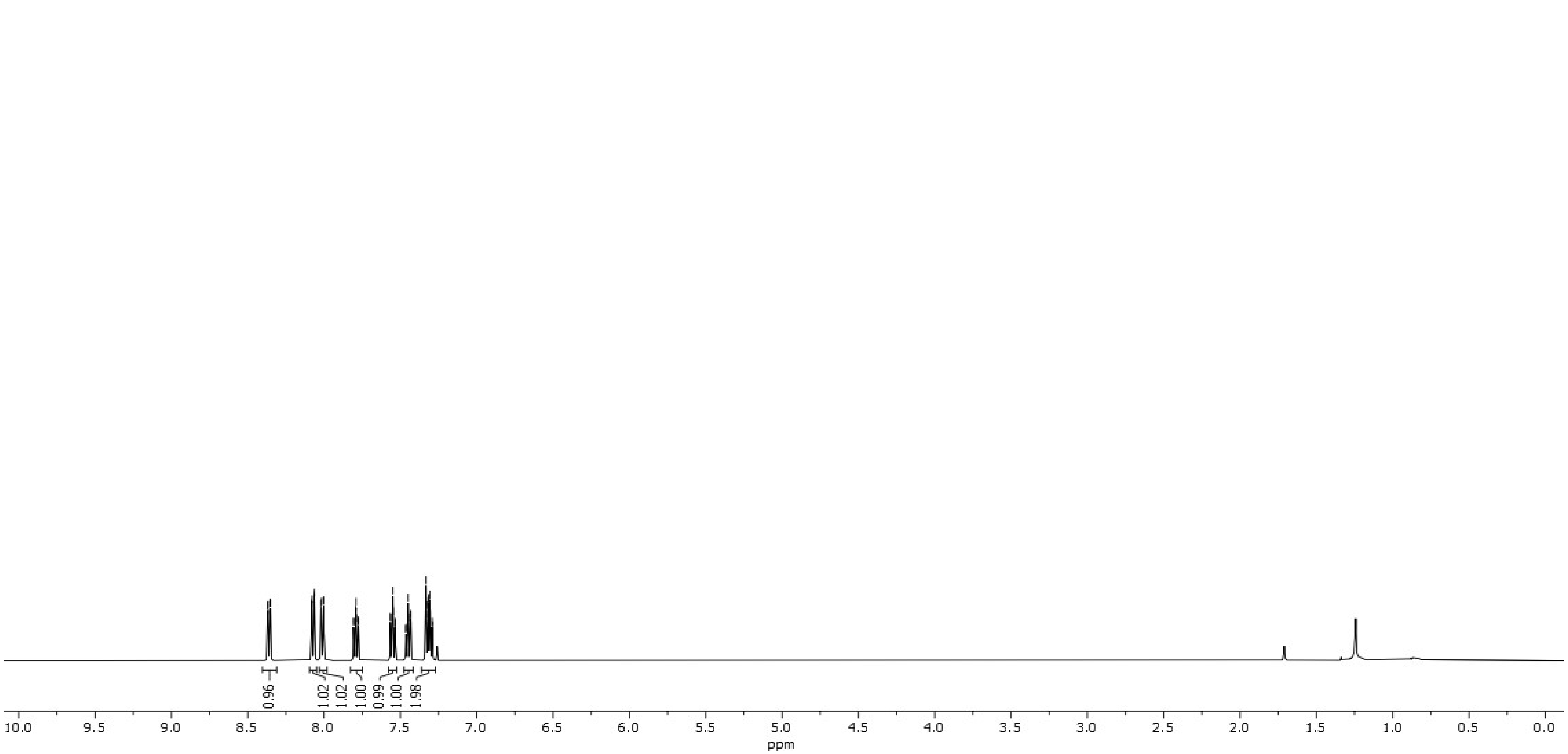

^{13}C NMR of 6*H*-benzo[c]chromen-6-one (S4)

$CDCl_3$, 298 K

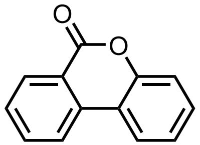

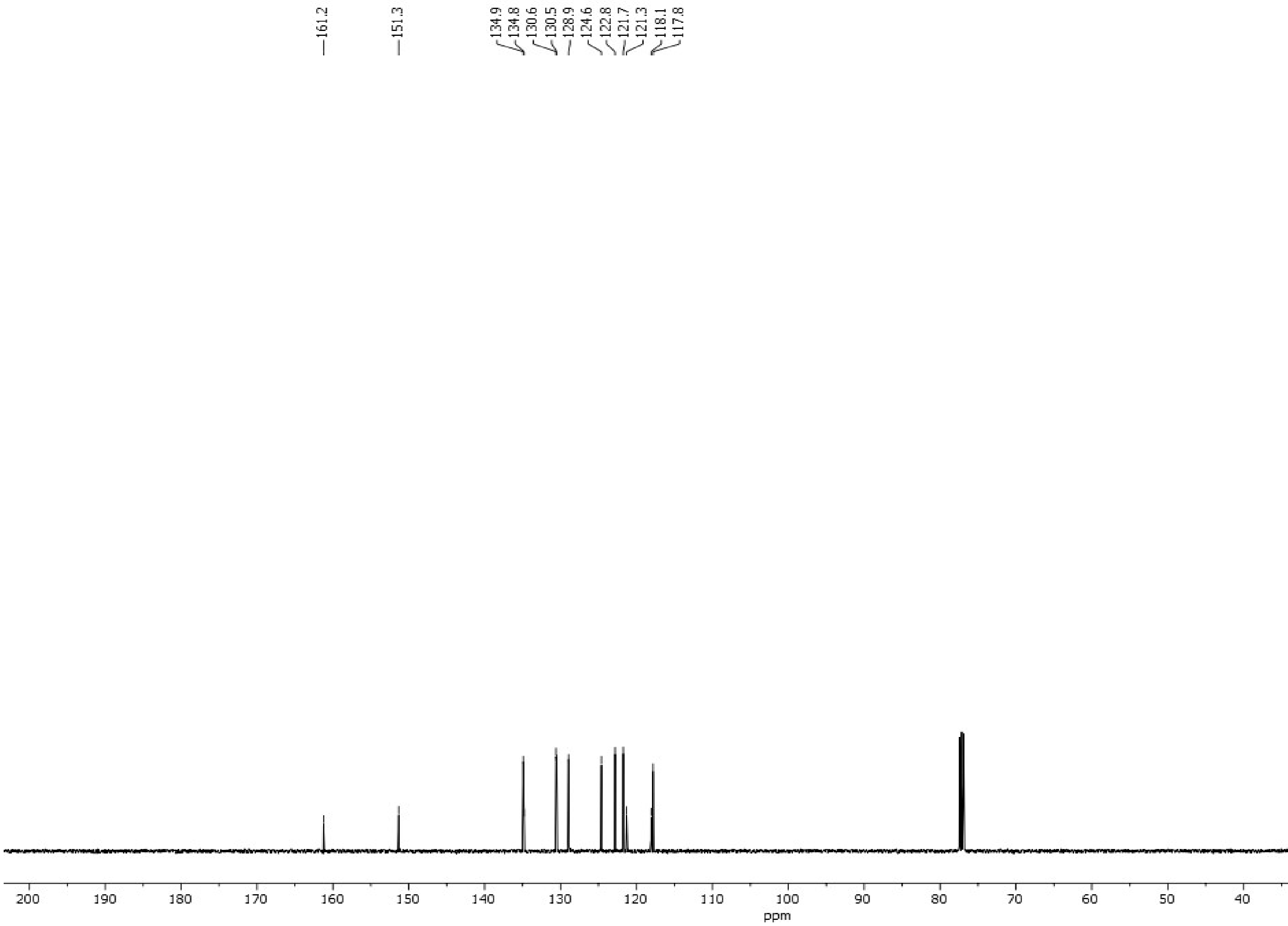

Supplement: SC-013-D2SC05442F-s001 [file SC-013-D2SC05442F-s001.pdf]
